# Evaluating a community engagement model for malaria elimination in Haiti: lessons from the community health council project (2019–2021)

**DOI:** 10.1186/s12936-023-04471-z

**Published:** 2023-02-09

**Authors:** Kevin Bardosh, Luccene Desir, Lorence Jean, Sarah Yoss, Brianna Poovey, Andrew Nute, Madsen Valerie Beau de Rochars, Marc-Aurèle Telfort, Fabiola Benoit, Ginette Chery, Marie Carmelle Charlotin, Gregory S. Noland

**Affiliations:** 1 School of Public Health, University of Washington, Seattle, WA USA; 2 The Carter Center, Atlanta, GA USA; 3 Ministère de la Sante Publique et de la Population, Jeremie and Port-au-Prince, Haiti; 4 Department of Health Services Research, Management and Policy, College of Public Health and Health Professions, University of Florida, Gainesville, FL USA

**Keywords:** Malaria elimination, Haiti, Community engagement, Participation, Vector control

## Abstract

**Background:**

Community engagement (CE) plays a critical role in malaria control and elimination. CE approaches vary substantially, with more participatory programmes requiring higher levels of adaptive management. This study evaluates the effectiveness of a volunteer-based CE programme developed in Haiti in 2018. The approach was based on local leaders organizing and implementing monthly anti-malaria activities in their communities, and was implemented as part of Malaria Zero Consortium activities.

**Methods:**

This programme evaluation draws on quantitative and qualitative data collected from 23 Community Health Councils (CHCs) over a two-year period (2019–2021) in Grand’Anse department, a malaria hotspot region in Haiti.

**Results:**

Monthly monitoring data showed that 100% of the 23 CHCs remained functional over the two-year period, with an average of 0.90 monthly meetings held with an 85% attendance rate. A high degree of transparency and diversity in membership helped create strong planning and involvement from members. CHCs conducted an average of 1.6 community-based activities per month, directly engaging an average of 123 people per month. High levels of fluctuation in monthly activities were indicative of local ownership and self-organization. This included school and church sensitization, environmental sanitation campaigns, mass education, support for case referrals and community mobilization during mass drug administration (MDA) and indoor residual spraying (IRS) campaigns. Members drew on the tradition of *konbit* (mutual self-help), local histories of health and development campaigns and a lexicon of “solidarity” in difficult times as they negotiated their agency as community volunteers. Small incentives played both symbolic and supportive roles. Some level of politicization was viewed as inevitable, even beneficial. Rumours about financial and political profiteering of CHC volunteers took time to dispel while the tendency towards vertical planning in malaria control created conditions that excluded CHCs from some activities. This generated resentment from members who felt sidelined by the government malaria programme.

**Conclusion:**

The CHC model was effective in promoting group solidarity and community-based anti-malaria activities over a two-year period in Haiti. With the end of the Malaria Zero Consortium in early 2021, there is now an opportunity to better integrate this programme into the primary healthcare system, evaluate the impact of the CHCs on malaria epidemiology, and promote the greater integration of CHCs with active surveillance and response activities.

**Supplementary Information:**

The online version contains supplementary material available at 10.1186/s12936-023-04471-z.

## Background

Hispaniola (Haiti and the Dominican Republic) is the last remaining malaria-endemic island in the Caribbean [1]. In 2020, more than 22,000 cases of malaria were reported, with Haiti accounting for 96% (MSPP, unpublished data). The Haitian Ministry of Public Health and Population (MSPP) outlined a new national malaria elimination plan in 2010 [2]. In 2015, a group of external partners together with MSPP in Haiti and the Ministry of Health in the Dominican Republic formed the Malaria Zero Consortium with support from the Bill & Melinda Gates Foundation (BMGF). Malaria Zero’s efforts were largely focused in Southwestern Haiti, particularly the high burden area of Grand’Anse department. Historically, this department has been a major source of malaria, accounting for 55% of all cases in Haiti in 2020 (MSPP, unpublished data). The consortium implemented a package of malaria elimination activities in Grand’Anse, including enhanced surveillance [3], community case management [4], targeted mass drug administration (MDA) and indoor residual spraying (IRS) campaigns [5] and community engagement.

Community engagement (CE) is now widely seen as a critical component for the success of malaria control and elimination [6–8]. Kaneko [9] argued for the necessity of scaling up community involvement from “simple participation” to “community direction”, where community members play a central role in planning and directing malaria diagnosis, treatment, education, and/or mass interventions instead of being passive social mobilizers, which is often the case with externally funded health projects. This is sometimes called community-directed interventions (CDIs) [10], an approach based on the principles of primary healthcare articulated in the Alma Ata Declaration of the 1970s but of increasing global policy relevance today [11].

Broadly speaking, community engagement (CE) is the process of engaging those who are affected by a particular problem in the process of solving and mitigating that problem [12]. There is a spectrum of different types of community engagement, from simple outreach and consultation to collaboration and shared leadership [12, 13]. According to McClosky et al. [13], *“Meaningful community participation extends beyond physical involvement to include generation of ideas, contributions to decision making, and sharing of responsibility”*.

Despite the increased emphasis on CE in malaria programmes, there continues to be uncertainty about the best models and approaches as well as evaluation methods [6–8, 13, 14]. Some of the major challenges relate to difficulties with individual and group motivation, trust in malaria services, incentives, political interference, capacity building, and integration with the health system [15]. Power and control, at different levels of decision-making and implementation, are also considered an important but difficult to define contextual issue [12]. CE is increasingly discussed as a local, ongoing and iterative process in need of constant feedback and change [16]; but there is a major tension between this vision and the existing biomedical and administrative norms that guide health organizations and funders [17].

To guide the evolution of CE approaches, the effectiveness of a novel community engagement model using Community Health Councils (CHCs) to assist with the control and elimination of malaria in Haiti was evaluated in this study. Implemented as part of the Malaria Zero Consortium activities, CHCs are voluntary membership organizations that promote community-based education and activities for malaria and general public health. This approach was developed based on insights during formative research in Haiti [18], and aimed to ensure local ownership, adaptability, and integration with the primary healthcare system. In this paper, we present data on the implementation of the CHC programme based on two years (June 2019 to May 2021) of monitoring and research data, and consider the implications for malaria elimination efforts in Haiti.

## Methods

### Study location

The CHC programme was launched in September 2018 in Grand’Anse department, which comprises 12 communes and is home to approximately 550,000 people in total. The department is located in the most southwestern point of Haiti, a full day’s drive from Port-au-Prince. Although Jeremie (the department capital) is connected to the national highway, many roads are in poor condition, making local community access a challenge. Towns and hamlets are more densely settled along the road network, with some remote mountain and coastal communities accessible only by foot or motorbike. Hurricane Matthew directly hit Grand’Anse in 2016, destroying tens of thousands of homes and interrupting and ultimately delaying Malaria Zero Consortium activities in the area.

Grand’Anse has the highest malaria burden in Haiti, accounting for roughly 50% of all cases in the country since 2016 (MSPP, unpublished data). Epidemiological data from the department (2016–2021) shows an annual average of 7,352 malaria cases (from 43,366 tests), 6,993 treated patients and 7 deaths reported per year (MSPP, unpublished data). The malaria transmission season generally peaks from November to January, following the primary annual rains from September to December.

**Fig. 1:**
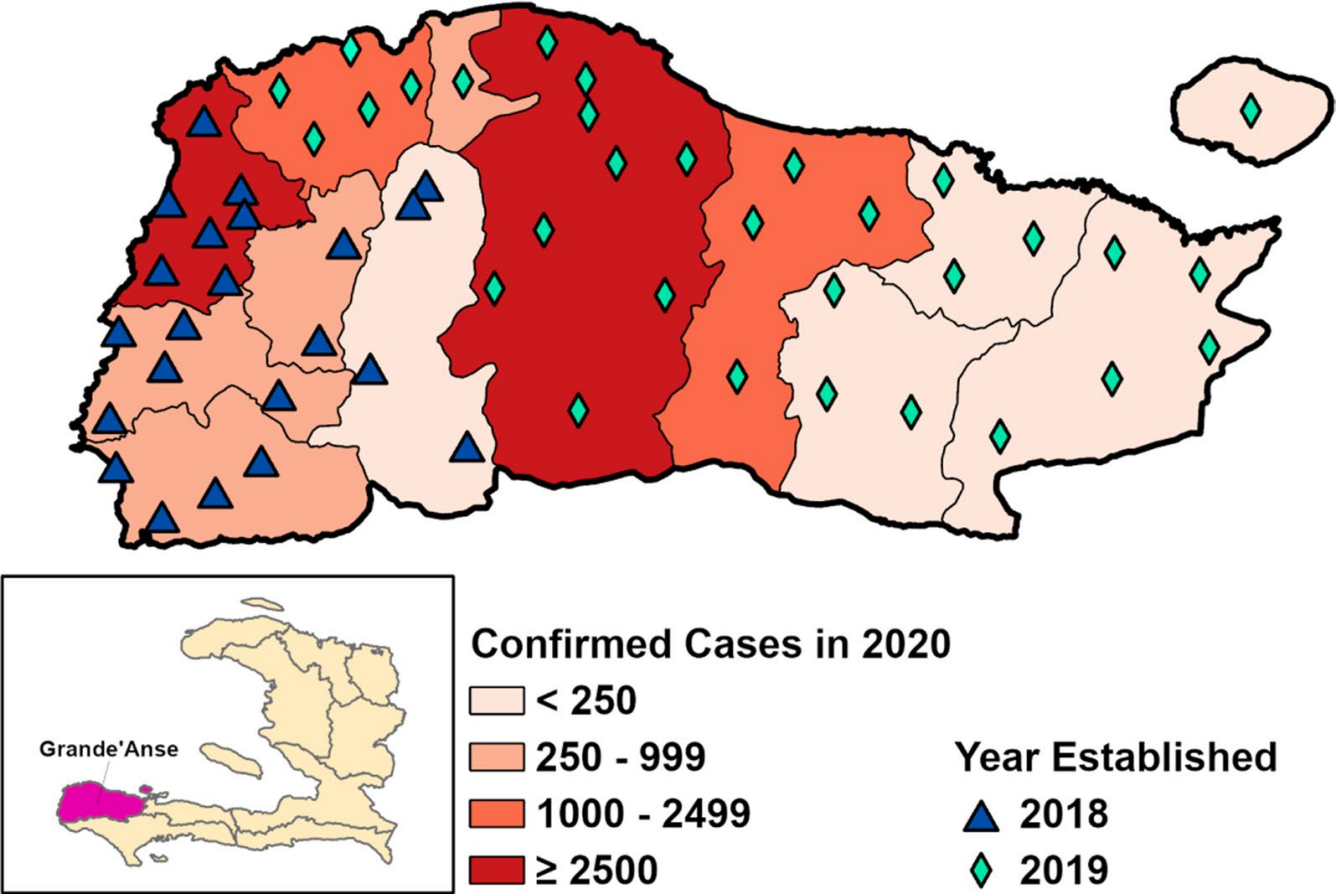
Study location, with 2020 malaria incidence data. The location of the original 23 CHCs included in this study are marked with a triangle, while 31 additional CHCs launched in 2019, and excluded from this study, are represented by a diamond. Note that some locations may have more than one CHC

Five communes (out of 12) were selected for an initial pilot phase that began in 2018. A total of 23 CHC groups were established, at the sub-commune level, covering an estimated 172,190 people (See Table 1 and Fig. 1). The selection of these five communes took into consideration malaria prevalence, but also road access, since these five communes share a common road west from the capital of Grand’Anse department, the city of Jeremie. Malaria Zero implemented mass drug administration (MDA) in 12 defined operational units (OUs) of Anse d’Hainault, Dame Marie, Les Irois, and Moron communes in the fall of 2018. Indoor Residual Spraying (IRS) was implemented in the same OUs except for one in Moron, where long-lasting insecticidal nets (LLIN) were distributed instead due to technical and logistical feasibility in this remote area. IRS treatment was repeated in 2019 in the same areas that received IRS in 2018. The MDA planned in all five communes for early 2020 were cancelled due to several cases of severe side effects after MDA in a neighboring commune as well as the COVID-19 pandemic. In December 2019, the programme expanded to all other communes of the department; however, this paper focuses exclusively on the data from the initial group of CHCs.

**Table 1:** Community Health Councils (CHCs) and total population, by commune

Commune	Number of CHCs	Total population
Anse d’Hainault	6	40,143
Dame Marie	6	42,731
Chambellan	4	29,179
Les Irois	3	25,777
Moron	3	34,360
Total	23	172,190

### Programme details

The CHC model was informed by a 4-week formative research period organized in mid-2018 in two communes of the department (Moron and Roseaux). A team of 10 field researchers conducted a total of 254 individual interviews and 252 group discussions on (1) local understandings of fever; (2) malaria treatment-seeking behaviour; (3) community trust and confidence in social institutions and networks; (4) sources and channels of health communication; (5) community perceptions of the malaria programme; and (6) community views on the possibility of malaria elimination. From this, a conceptual working document, *Malaria Zero Community Engagement Plan* [18], was finalized in mid-2018. Establishing the CHCs involved organizing an original training workshop for each CHC in 2018, followed by training workshops on community mobilization and malaria in 2019. A CHC Implementation Manual was finalized in 2020, as was a *Monitoring, Evaluation and Learning Handbook* (2020), both of which are available in English and French in the Additional file 1.

Selection of CHC members took place during meetings in mid-2018, organized by the research team in collaboration with MSPP. In each sub-commune (each CHC operates in one sub-commune), MSPP organized a meeting with 40–50 people that included: (1) local authorities (mayors, l'Assemblée de la Section Communale (ASEC) and the Conseil d'Administration de la Section Communale (CASEC), justice departments, local governors); (2) representatives of community-based organizations (farmers, youth, women, teachers, business leaders); and (3) community leaders (pastors, priest, Polyvalent Community Health Agents (ASCPs), traditional healers). An effort was made to ensure that people came from different geographical areas of the sub-commune. Guidelines were provided to all participants about the selection process, management of the CHCs and the types of activities to be conducted. This emphasized the exclusive voluntary nature of these groups. At the meeting, a list of names of potential members was generated and, in an open and transparent process, 9–13 people were selected with the aim of ensuring a broad representation of professional backgrounds, gender, age, socioeconomic status and residence location. Subsequently, meetings were held with each CHC for training on health promotion and malaria transmission, prevention and control,. A board of directors was created through an open vote for a coordinator/president, secretary/treasurer and two delegates/advisors. Roles and responsibilities for each role were clearly defined and distributed to all members. The board was responsible for organizing meetings, liaising with MSPP, preparing monthly reports of activities, mobilizing members, keeping track of resources and drafting plans. The original goal of the CHC programme was to have each group organize one planning meeting and four community-based activities per month. Clean-up equipment (including wheelbarrows, shovels, boots) were provided to each CHC to facilitate and encourage environmental sanitation activities.

The research team also conducted training activities with the malaria programme staff in Grand’Anse and commune-level MSPP health staff, both of who were trained and organized to provide routine and ongoing monthly support to the CHCs in leadership, planning, implementation and monitoring.

### Evaluation methods

This paper is based on 24-months of data collected from June 2019 to May 2021 including monthly monitoring data, two rounds of qualitative interviewing and observational notes gathered during training and monitoring visits.

CHC activity data were collected by the research team on a monthly basis, from the secretariats of each CHC who collated data using paper forms. These data were communicated to research staff either through telephone or in-person monitoring visits. An online database was established using open source electronic data collection software [19]. Data were then cleaned in Python 3 and analysed in R Statistical Software and Microsoft Excel for descriptive analysis.

Two rounds of qualitative research were also conducted. These were organized opportunistically to coincide with training and support activities provided to the CHCs. The first was in June 2019 and consisted of four in-depth qualitative interviews with MSPP staff and four focus group discussions (FGDs) with all CHC group leaders from three of the five communes: Les Irois, Anse d’Hainault and Dame-Marie. These communes were selected based on logistical constraints at the time. The second round of qualitative interviewing included semi-structured interviews with one representative of each of the 23 CHCs in June-July 2020. We asked each group to nominal one of the 3 leadership members for the interview. Due to the COVID-19 pandemic, two-thirds of these interviews were conducted remotely by telephone and one-third in-person. Most interviews lasted one hour while FGDs lasted approximately two hours. The interview and FGD guides for both rounds of qualitative research are provided in the Additional file 2. All qualitative data were recorded using hand-held voice recorders, and were subsequently transcribed and translated from Haitian Creole to French for analysis, which was done manually using Microsoft Word and Excel. A code list was developed and refined to guide inductive thematic analysis.

Lastly, participant observations were done by research staff during trainings and monitoring visits in a variety of reports. This included reports from the MDA and IRS campaigns in 2018–2020. During the two-year period, twice-monthly group management teleconferences were conducted to discuss emergent challenges and debriefed training, field monitoring and implementation. Notes from these meetings were included in the analysis.

### Ethical approval

Ethical approval was sought from Emory University’s Institutional Review Board (IRB), and a research ethics exemption was obtained. This was sufficient for Haiti’s Comité National de Bioéthique, who recommended against a formal application.

## Results

### CHC meetings, governance and planning

**Fig. 2:**
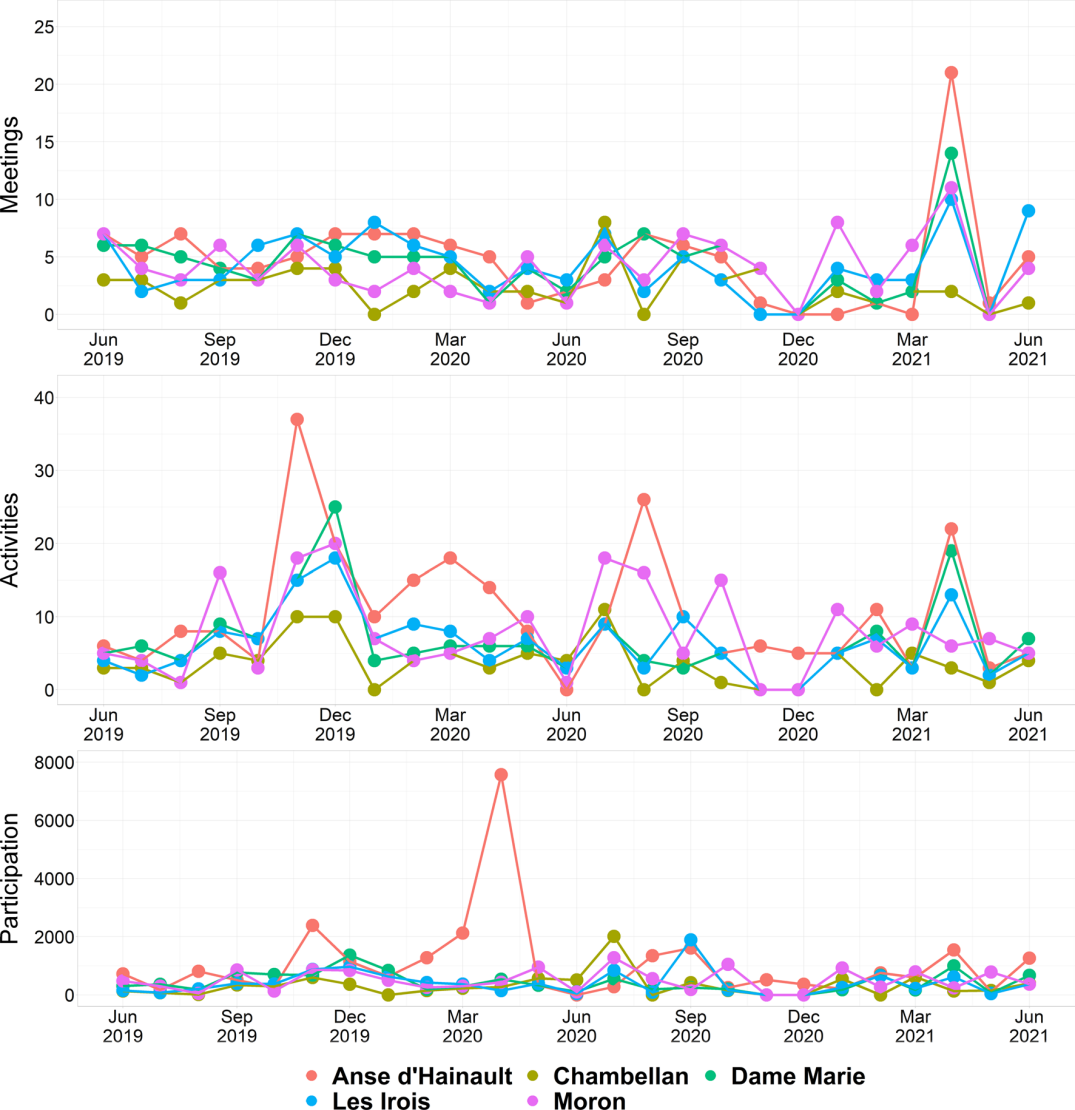
Monthly CHC performance data, average by commune, including: (1) Group planning meetings (top), (2) community activities (center, described in the next section) and (3) number of community members engaged (bottom)

The 23 CHCs conducted 498 group meetings over the 24-month period (June 2019-May 2021), with an average of 0.90 meetings/month per CHC (Table 2). This was close to the programme goal of 1 group meeting per month. No group was found to cease functioning over the project period. Meeting frequency was highest in Les Irois (1.15/month) and lowest in Dame Marie (0.76/month). Overall, we found substantial variation in meeting frequency per month and attendance over time, with a high level of monthly fluctuation shown by most groups (Fig. 2). Nine (39%) CHCs held more than one group meeting per month on average. The lowest preforming group was found in Anse d’Hainault (0.46 meetings/month) while the highest was in Les Irois (1.8/month).

**Table 2:** CHC meetings and attendance (June 2019-May 2021)

Commune	Total population	Number of CHCs	Total # CHC meetings	Average number of meetings per month	Average meeting attendance (%)
Anse d'Hainault	40,143	6	117	0.81	94
Chambellan	42,731	3	60	0.83	82
Dame Marie	29,179	6	110	0.76	79
Les Irois	25,777	4	107	1.15	82
Moron	34,360	4	104	1.08	89
Total	172,190	23	498	0.90	85

Qualitative data found that CHC activities were strongly influenced by governance processes, especially the original approach taken in the selection of members as well as a set of roles and responsibilities in management. CHC members strongly linked the transparent membership selection process, which ensured a diversity of community representation, to the effectiveness of CHC activities and their sustained functioning. Group members remained very positive about membership composition more than two years into the CHC programme. This was contrasted it to the more top-down and opaque selection process used in many other development and health projects in Haiti. Some leadership changes were required in 4 groups during the study period, due primarily to dropout rates.

The average attendance rate at monthly meetings was 85% across the 5 communes (Table 2), and few members left the groups during the study period. The commune with the lowest average attendance, in Dame Marie, had an attendance average of 71%. Qualitative data suggested that groups in more urban areas (which includes Dame Marie) had less attendance and meetings compared to those in peri-urban and rural areas.

CHCs planned activities from month-to-month, rather than taking a more long-term view with clear schedules and agreed agendas. Some additional planning did take place for special events and holidays but, on the whole, there was a great deal of fluidity to the ways groups self-organized. This was reflected in the fluctuation of meetings and activities (Fig. 2).

CHCs adapted the implementation guidelines in ways that made sense to them and aligned with shared group priorities. Most CHCs created their own oaths, mission statements and songs, and many also formed WhatsApp or Short Message Service (SMS) chat groups. These efforts emerged spontaneously. Group decision-making was described in terms of consensus generation. Common challenges involved the distance some members had to travel to attend meetings, the reimbursement of small expenses used by individual members and a lack of protocols and guidance in planning. While most CHCs appeared to have strong leadership teams, a number expressed problems with coordinators trying to dominate their groups. Members attempted to rotate the areas where activities were performed, and expressed frustration at the fact that their coordinators tried to focus solely on geographical areas favorable to them. CHC members emphasized the need for the “separation of power” and emphasized the power dynamic of group membership. Some recommended sub-dividing CHCs into smaller groups given that some sub-communes involved large geographical distances without travel reimbursements.,

### Frequency and type of community-based anti-malaria activities

Each CHC conducted an average of 1.6 community-based activities involving 123 community members per month, lower than the original goal of 4 community-based activities/month (Tables 3 and 4).

**Table 3:** CHC community-based activities

Commune	Number of CHCs	Average number of activities per month/CHC	Environmental sanitation activities	Awareness-raising activities	Other activities	Total number of community-based activities
Anse d’Hainault	6	1.82	115	150	34	262
Chambellan	3	1.26	41	60	10	91
Dame Marie	6	1.15	83	105	27	166
Les Irois	4	1.65	80	87	15	158
Moron	4	2.07	110	113	33	199
Total	23	1.59	429	515	119	876

**Table 4:** Number of people reached by CHCs

Commune	Population	Number of CHCs	Total # of community-based activities	Average number of community-based activities by CHC per month	Total number of reported individuals reached during activities	Average number of people reached by CHC per month
Anse d’Hainault	40,143	6	262	1.8	26,789	186
Chambellan	29,179	3	91	1.3	7,972	111
Dame Marie	42,731	6	166	1.2	10,607	74
Les Irois	25,777	4	158	1.6	10,192	106
Moron	34,360	4	199	2.1	12,518	130
Total	172,189	23	876	1.6	68,078	123

As with the monthly meetings, we found substantial variation in frequency, activity type and number of people reached, with a high level of monthly fluctuation shown by most groups (Fig. 2). CHCs in Anse d’Hainault and Moron communes organized more activities per month compared to Chambellan, Dame Marie and Les Irois. The most active group was in Anse d’Hainault (2.8 activities/month on average), which reported conducting 4 or more activities per month for 46% (11/24 months) of the reporting period. These differences were discussed in terms of leadership and motivation.

Community-based interventions consisted of malaria awareness-raising activities (n = 515), environmental sanitation activities (n = 429), and a series of “other” activities (n = 119). Awareness-raising activities included: education about malaria prevention, public meetings at schools and churches, public education, awareness-raising about MDA and/or IRS campaigns, education about fever-seeking behaviour and promoting malaria test and treat strategy. Environmental sanitation activities included: doing environmental improvement, sensitizing community members on environmental sanitation, and organizing and participating in community-based environmental sanitation campaign.

**Fig. 3:**
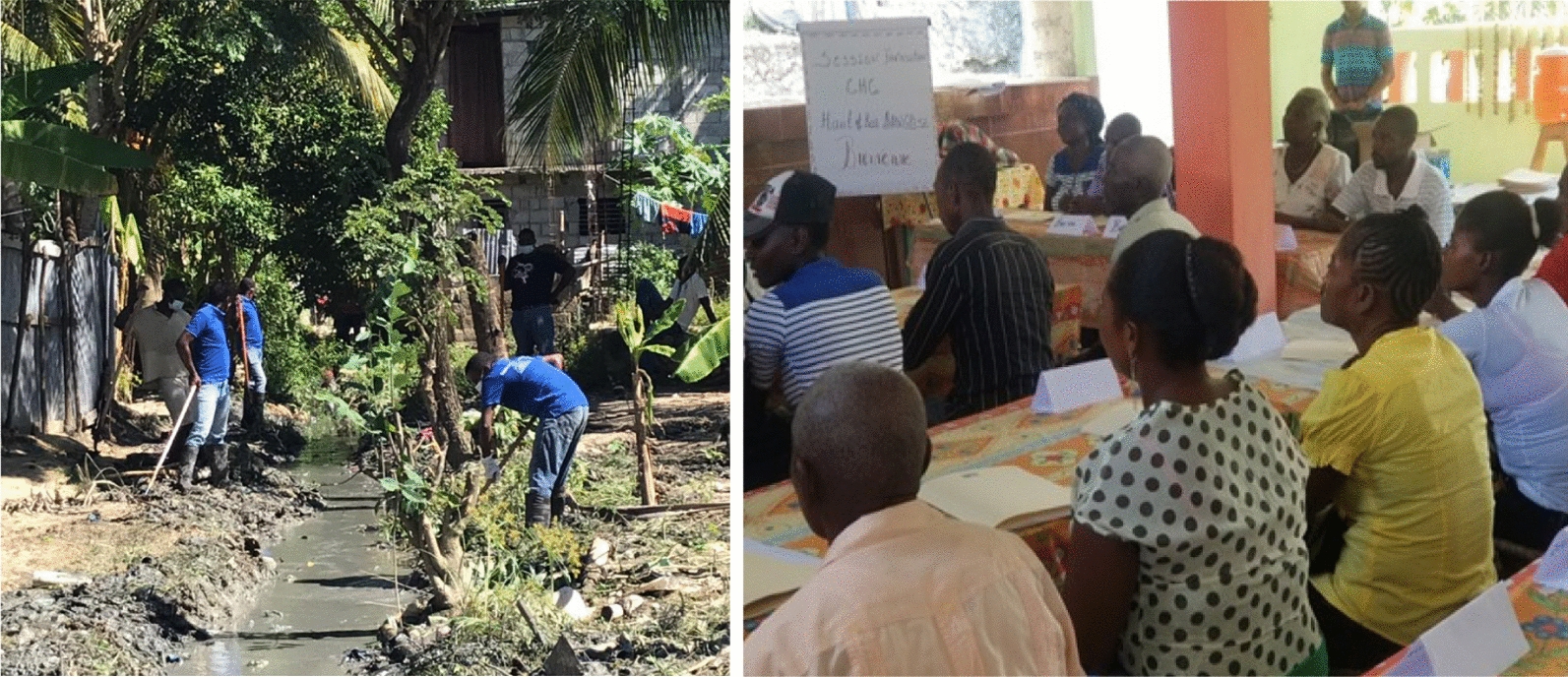
Environmental sanitation activities (left) and Group meeting (right)

Most environmental sanitation activities focused on garbage clean-up and stagnant water sources. Garbage clean-up typically concentrated on plastic objects, empty pots, coconuts, shells and tires, while efforts to address stagnant water sources involved cleaning and draining canals along roads and homes (See Fig. 3). Interviews and observations with CHC members (as per the guidance of entomologists from MSPP) showed that these activities were infrequently targeted to *Anopheles* breeding sites and that the campaigns likely over-emphasized general clean-up instead of mosquito control. Larger campaigns were often organized to correspond to festivals or holidays.

CHCs also conducted education activities. In our interviews, members especially stressed the importance of school and church meetings and sensitization. In schools, children were encouraged to discuss malaria information with their families once they returned home. Churches were seen as very influential venues to spread information and mobilize support for early diagnosis and treatment, given the high level of trust with church leaders. As with environmental sanitation campaigns, CHCs also organized educational outreach during festivals and holidays, and MDA and IRS campaigns organized by the Ministry of Health (MSPP). Megaphones provided to CHC were used for sensitization at water kiosks, bus stops, public squares, markets, and street corners as well as and during door-to-door education.

CHCs emphasized their ability to “change behaviors” and that this awareness-raising capacity was something that MSPP could leverage in their anti-malaria outreach activities. However, members requested more training on behavior change techniques; many found it very challenging and time-consuming. While CHCs believed that they had increased knowledge of malaria and helped reduce malaria, it was not clear if their activities targeted local malaria hotspots or areas with active cases. Interviews in 2020 suggested that some CHC members had provided direct support to people with malaria symptoms including advising and supporting them to seek treatment and sensitizing pharmacies, mobile drug vendors, herbalists and traditional doctors (*hougan*) in malaria testing and treatment. However this was not systematically documented by the CHCs.

### Negotiating volunteerism and project inputs

The volunteer-based nature of the CHCs required careful negotiation to avoid misperceptions and demotivation. In our qualitative data and field visits, group members frequently mentioned a sense of mission, feeling valued, being useful, and various statements of solidarity (“working for and with the population”). On the other hand, members consistently mentioned the lack of small incentives and remuneration as a major barrier to CHC participation, the fact that members are “busy people” and that, while they are willing to volunteer, “life is expensive and this inhibits people to participate in the CHCs.”

The original aim of the programme was to avoid direct cash incentives and to emphasize the “volunteer-based” nature of the programme. In our 2019 interviews, and during routine monitoring, CHC members emphasized the need for small financial and non-financial incentives and that these would make a big difference to their overall motivation and effectiveness. This included the need for regular trainings and larger group meetings with MSPP:

*“We have had training but we must understand that people must have continuous training because reporting work is difficult for our level. We're not used to it.”* CHC member, interview, 2020.

Members also mentioned the need for more education materials, as well as community training material, small incentives to help with group meetings, T-shirts, and cleanup materials. After an initial trial period in 2018, a flexible incentive of $100 USD(10,000 HTG) was provided to each CHC every two months to assist with the group meetings. This was clearly understood to be an incentive for drinks/snacks during meetings to help organize their work and was reduced to $100 USD every two months in early 2019. After approval by MSPP, T-shirts with the MSPP logo were provided, and all members believed this would increase the visibility and legitimacy of their work. Clean-up materials (boots, rakes, pickaxes, wheelbarrows, shovels, and machetes) were given to each group. In 2019, nearly a dozen motorcycles were provided to MSPP to help with MSPP supervision and support.

While interviews in 2019 found a continued expectation of greater financial incentives and resources, these reduced in 2020. All CHCs reported that they had community activity plans that had not been realized due to a lack of funds: for water, drinks, food, and local alcohol (rum was requested especially in remote areas by community volunteers involved in environmental cleanup). Members repeatedly highlighted that they have used their own resources to attend meetings and, in some cases, to organize community outreach. They understood that MSPP staff have access to travel and salary funds, and do not understand why greater resources are not provided to them for travel and community outreach. Members questioned the meaning and nature of “volunteering”, in this regard. CHC members also mentioned that “volunteers” in other health programmes (e.g., vaccination) still receive a small personal stipend to cover their transport and food at a minimum, albeit these programmes operate only for a few days on an annual basis.

*“Volunteering does not mean spending your entire life and even your savings. We do not have money. If you are organizing an activity you may need water. You have to take it into account.”* CHC member, interview, 2020.

Inputs also contributed to demotivation, as shown by our effort to establish an electronic data reporting system. Before 2019, MSPP staff would physically visit each CHC on a monthly basis to collect paper Monitoring & Evaluation (M&E) forms. To improve efficiency and timeliness of reporting, we distributed in December 2019, smart-phones that enabled online or offline data collection followed. A few months after the launch, we found a number of challenges with the phone system: coordinators keeping the phones, lost and broken phones, difficulties recharging the phones, and a general lack of internet connection. In mid-2020, programme staff transitioned to calling each CHC focal person to collect monthly M&E data by voice call. These monthly calls also became an opportunity for programme staff to build relationships with CHC members; the regular contact allowed CHC members to debrief about monthly activities and allowed staff to encourage members in their work.

### Politicization and community mobilization

Group outreach activities had to negotiate political and social dimensions, described by CHC members as “politicization.” On the one hand, some CHC members were labeled to be opportunists: “they will sneak into every NGO opportunity and try and take advantage of things” (CHC member, interview, 2019). On the other hand, political profiteering was also seen as an accepted, somewhat beneficial, aspect of the CHCs since members could leverage the group for their political ends. Balancing this was important and discussions about politicization reduced significantly in 2020, and appeared to have decreased with time.

CHC members saw their role as facilitating and mobilizing other community members to engage in malaria awareness and environmental sanitation rather than conducting the field activities themselves. CHC members “recruited” people in their social network, namely relatives or neighbours. Community members, in turn, were suspicious that the CHCs were receiving large salaries or remunerations: "*Moun lajan yo*” (interpretation: They are using people for their benefit). Many stressed that volunteering, “does not exist in poor countries [like Haiti]” and that people with links to a development and health programme always find some way to benefit personally. This generated community suspicion about CHCs membership being ‘voluntary.’ An often-repeated example involved the widespread perception that previous bed net distributions in 2017 had lead to misappropriations whereby volunteers had used their positions to sell the nets in the local market. The original emphasis on transparency with selecting members, transparently reporting CHC finances by the secretariat, and holding meetings in each sub-commune with a wide variety of stakeholders on this issue helped to address these concerns.

The politicization of CHCs appeared to be much stronger in urban centers, especially in Dame Marie (with the lowest average meeting and community activity rates), and involved the spreading of rumours about CHC members being paid for their work and tied to specific political parties.

### Relationship with the vertical malaria programme and Ministry of Health

Our qualitative interviews found challenges with the way malaria programme partners and the Ministry of Health (MSPP) engaged with the Community Health Councils (CHCs). Contrary to original plans, CHCs were not involved in planning for the first targeted MDA and IRS campaign in 2018. CHCs were involved in social mobilization and community education as part of this campaign, but the process of recruiting community members to assist the campaign was primarily done by local health staff rather than CHCs. In some cases, the coordinator of the CHC was involved in the selection but without involving the other CHC members. Health staff and CHC coordinators often appointed family members instead of transparently picking members. This created resentment and anger from many CHC members.

*“The coordinator delegated his sister to participate in MDA activities instead of choosing a committee member. Committee members were frustrated. They make choices based on their political affiliation, allies and friends.”* CHC member, interview, 2019.

It was also unclear to CHC members why some communes and sub-communes were targeted for MDA and IRS while others were not targeted. The targeted MDA/IRS campaign in 2018, for example, targeted 4 of the 5 communes with CHCs based on risk models but the rationale for selection was not sufficiently explained to, or understood by, CHCs or communities across the communes ahead of time. This furthered rumours of favoritism in the distribution of activities and their benefits.

These recruitment and communication challenges were improved slightly in the 2019 IRS campaign but not fully addressed.

Interviews in 2019 and 2020 showed that CHC members strongly felt that health staff from MSPP were not providing sufficient support and integrating with their activities. Groups involved MSPP in different ways; in some cases, nurses and community health workers were part of the CHC membership while in others they would participate occasionally in activities and as observers during meetings. This perceived lack of involvement by MSPP was confusing for CHC members and contributed to a sense of demotivation.

CHCs believed that MSPP should actively promote the groups, integrate them in their routine activities such as larval surveillance and vector control outreach, organize collaborative education activities during large events and holidays (including World Malaria Day on April 25th of each year) and integrate members in malaria case investigation. They stressed the need for higher-level committees that could convene different government departments to address larger vector control problems, specifically involving roadwork and public infrastructure including garbage collection.

CHC members also highlighted the need to better link their work to that of health workers. A number expressed frustration that when they would recommend people to seek malaria tests, the tests would be unavailable. Stockouts were not communicated to the groups, and CHCs felt that there should be better overall communication between local health officers and the CHCs. CHCs also noted the lack of linkage with the vector control division and asked for larvicides and the ability to monitor larval habitats. In particular, CHCs felt “overwhelmed” by large stagnant water sources and requested more support to address them.

Interviews in 2019 and 2020 also found that many members did not feel sufficiently trained on malaria. Some interviews raised questions about the knowledge of CHC members; for example, they may not know the name of the mosquito that transmits malaria or the name of the pathogen and some were confused about how malaria elimination can occur in the absence of eliminating mosquitoes (a common belief found in the formative research).

## Discussion

Our data from 23 Community Health Councils (CHC) showed that the CHC approach could be implemented and maintained in Haiti, despite programmatic and contextual challenges. No CHC stopped functioning, the programme achieved a high-level of local ownership, and planning, implementation and monitoring took place with relatively nominal external support and oversight. The CHCs helped build local capacity for malaria activities in settings where formal health staff and outreach is low and minimal, and increased the number of routine malaria interventions at the sub-commune level, especially education at schools, churches and community venues and environmental sanitation activities.

CHCs were initially developed under the Malaria Zero Consortium grant, which ended in early 2021. At this time, 71 CHCs were operating in Grand’Anse and Sud departments, covering nearly one million people in total. Although this study only focused on data from the original 23 CHCs, lessons learn here are applicable to the wider CHC project and to other similar initiatives in other countries.

As discussed above, CHC members emphasized four main factors in helping to motivate and sustain their groups: (1) the diverse composition of group members; (2) the different types of inputs and incentives; (3) the fostering of a shared sense of purpose that relied on existing social networks; and (4) trust relationships and supervision from MSPP.

CHC activities were strongly influenced by the original approach taken in the selection of members and management guidelines provided by programme staff. This helped establish an ethos of transparency and fairness. The fluctuation in monthly meetings and activities reflected this local ownership; groups coordinated and engaged in activities around their life schedules rather than prescribed routines. This was likely influenced by the example of primary healthcare community groups in the 1970–80 s, the long-standing agricultural tradition of “*Konbit”* and a culture of volunteerism that has long been a feature of the Haitian Catholic church [20].

The study showed that urban centers, such as Dame Marie, presented greater problems negotiating group interests due to the more politicized social landscape. Rural areas struggled more with negotiating where to conduct activities given the larger geographical distances between group members.

While groups showed high levels of endogenous motivation, external incentives were a fundamental and expected part of the project, as they are with most community-based health activities [7, 21]. This involved more than just financial reimbursements but also symbolic and supportive inputs. This included the use of physical inputs for environmental cleanup, money to facilitate group meetings, and t-shirts, which was felt to increase the legitimacy of field activities and the sense of group solidarity. Initially project staff resisted the idea of direct financial input. This was due to concerns that it would politicize the CHCs and would not be ‘sustainable’ when the project was to transition to MSPP and no longer have any support outside the government health system. However, after a few months in 2018 (prior to the data collection period), and after feedback from CHC members, the project team began to provide financial support to facilitate group meetings. Regular contact and encouragement to groups, conducted at least once a month by phone, and reinforced through physical visits and workshops, helped maintain feelings of support and built relationships between the project team and CHCs. These strategies (financial support and regular contact) proved vital to sustaining interest in the CHCs during the difficult period of the COVID-19 pandemic and the ongoing socio-political crises in Haiti, both of which prevented transport by the project team to the area for periods of time.

CHCs also voiced that a *stronger* partnership with the health sector was a vital part of sustaining group motivation. Members stressed the need for more direction in vector control, case management and outreach during the targeted MDA and IRS campaigns. At a minimum, this study shows that CHCs in Grand’Anse should be used as a platform for organizing future vertical anti-malaria interventions, such as MDA, IRS and bed net distribution. They also emphasized their desire to be ‘more than just a project’ by formally integrating within the MSPP primary healthcare system. Current efforts in Haiti have institutionalized networks of community health workers (ASCPs), and some work with CHCs. The Haitian health system, however, continues to struggle with basic service delivery, infrastructure and resources, challenges that are rooted in unstable financing and ineffective management [22]. Ongoing political and economic crises add ever-greater difficulties including with the morale of staff and external partners while also contributed to some setbacks with malaria control and elimination goals [1–3].

This interest was not limited to malaria but also involved a desire to expand CHC activities beyond malaria to other local health issues (e.g. childhood immunizations, maternal health, chronic disease). As noted by [8], this is an important strategy to address a paradox in malaria community engagement programmes: as elimination succeeds, cases reduce and group motivation becomes harder to sustain.

This evaluation study suggests a few ways to strengthen the effectiveness of CHC activities against malaria. A 2018 prospective case–control study in Grand’Anse, Haiti, found that location of residence, especially remoteness and inaccessibility (including seasonal fishing camps), was the most important factor in malaria positivity and that malaria cases cluster in locations of 2 to 10 km^2^ in size [4]. So far, the geographical focus for CHC activities have not been sufficiently oriented based on detailed epidemiological or risk data at the sub-commune or neighborhood level, although it has been informed by macro-level burden assessments. With more flexible financing and decentralized coordination [23], CHCs could participate in outbreak community mobilization as well as orientate their activities to communities with recent outbreaks.

Qualitative research on malaria in Haiti, including in Grand’Anse, has shown that local people tend to have a low level of detailed knowledge about malaria and associate it with “dirty environments” such as swamps and trash, as well as general poverty, and equate the existence of all types of mosquitoes (which are ubiquitous) with general malaria risk [24]. Malaria is conflated with fevers in general, with any type of fever being viewed as a form of malaria. There is an important role for CHCs to play in increasing basic malaria knowledge at the community level; but, as this study has shown, CHCs members themselves appear to lack knowledge about malaria itself and so would need better training before conducting more effective community education.

Because of this link between malaria and “dirty environments”, CHC activities have focused heavily on environmental sanitation (“*assainissement*”). In fact, CHC members frequently associated the failure of the state’s malaria control policy with the lack of canal maintenance, and believe that success can only be achieved through large-scale changes in infrastructure, governance and environment that mediate the distribution of stagnant water bodies. It is unlikely that current efforts by the CHCs are having a substantial impact on mosquito populations [25]; a more focused, consistent and integrated approach, led by MSPP and targeted to specific high-risk areas and specific sources of mosquito breeding – perhaps as part of a rapid response outreach approach to respond to active malaria cases – would help improve these activities. Mosquito control in Haiti also has beneficial efforts for *Aedes*-borne viral diseases (Zika, Chikungunya, Dengue) and *Culex*-transmitted lymphatic filariasis that is also targeted for elimination, albeit these different mosquitoes do have different vector dynamics (feeding and breeding behavior) that need to be considered [25]. Lessons can be drawn from a related recent community engagement pilot study in Northern Haiti, with community vector control groups, known as “mosquito police” [26].

MSPP has shifted the goal of malaria elimination in Haiti from 2020 [1] to 2025 in its revised National Strategic Plan for Malaria Elimination. When asked about the feasibility of malaria elimination in Grand’Anse, people have a wide variety of viewpoints. One consistent theme is that if MSPP and external partners want to achieve this goal, greater investment in local community action, such as CHC activities, are essential.

### Study limitations

There are a number of limitations to this study. First, the study relied on monthly self-reported quantitative activity data reported verbally by each CHC secretariat. To minimize false and error reporting, the study attempted to randomly triangulate reported data by contacting more than one CHC member. The study team also conducted site visits roughly every 3–6 months, where CHC members were convened to discuss activities and progress. The fluctuation in monthly reporting data provides confidence that reporting was accurate. Although the study attempted to categorize activities, there may have been overlap between activity types. Likewise, the number of people reached may be an overestimate as the same person may have been reached in more than one activity. Second, qualitative data collection was conducted by a programme staff member directly involved in implementing the CHC programme. Although this may have led to some bias in participant responses, CHC members were willing to discuss their challenges in a frank and honest way. Research efforts to ensure anonymity likely helped reduce reticence to discuss sensitive internal conflicts within each CHC. Third, the analysis does not allow for inferences about the effectiveness of CHC activities on malaria incidence or improved early diagnosis, treatment or prevention, as baseline and intervention data were not collected. Although originally planned, the project team was not able to organize the collection of these data, largely due to the COVID-19 pandemic. Further work is needed to evaluate the impact of the CHC approach on malaria epidemiology in Haiti.

## Supplementary Information

**Additional file 1. **CHC implementation manual English and French.

**Additional file 2. **CHC M&E handbook English and French.

## Data Availability

All data is reported in the manuscript. Datasets are available upon request.
