# Supplementary material for: Evaluating a community engagement model for malaria elimination in Haiti: lessons from the community health council project (2019–2021)
Source: Malar J. 2023 Feb 9;22:47. doi: 10.1186/s12936-023-04471-z (PMC9910254; doi:10.1186/s12936-023-04471-z)
Supplement: Supplementary file 1 — Additional file 1. CHC implementation manual English and French. [file 12936_2023_4471_MOESM1_ESM.zip › New folder/CHC Implementation Manual_english.pdf]

2020

Community Health Councils (CHC): An Implementation Manual for Community Engagement and Malaria Elimination in Haiti

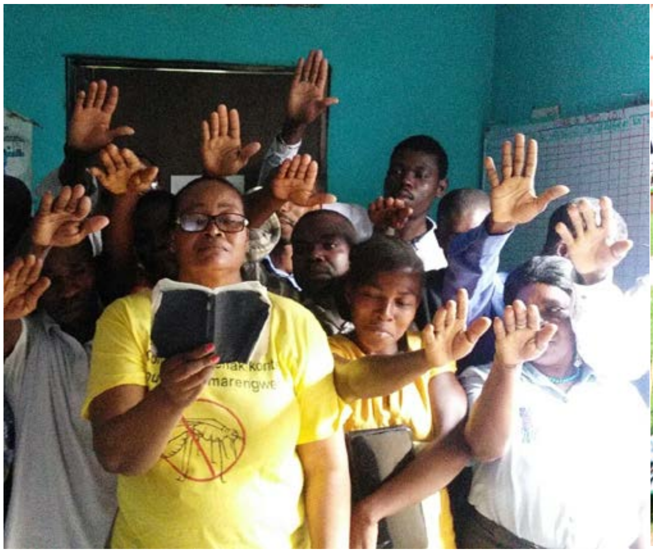

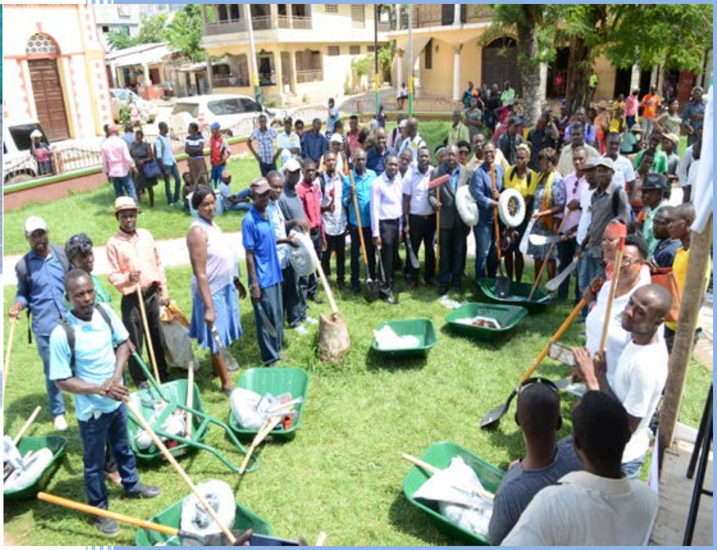

This is a step-by-step manual for members of community health councils (CHCs), staff from the Ministère de la Santé Publique et de la Population (MSPP), and other implementing partners in Grand Anse, Haiti. It provides detailed guidance for how CHC members should establish and run the day-to-day activities of their community health councils, and how MSPP staff should support them in doing so. These two elements are critical for strengthening community health and eliminating malaria from Haiti.

Suggested citation:
Bardosh, K., Jean, L., Desir, L., Yoss, S., Nu, S., Poovey, B., Blount, S., and Noland, G. (2020). *Community Health Councils (CHC): An Implementation Manual for Community Engagement and Malaria Elimination in Haiti*. The Carter Center: Atlanta.

Table of Contents

How to use this manual **1**
CHAPTER 1: The Community Health Council Approach **2**
1.1. What is a Community Health Council? **2**
1.2. Why are CHCs important for malaria elimination? **3**
1.3. CHC activities and scope of work **4**
1.4. Support and assistance provided to CHCs **5**
1.5. Sequence of steps in establishing a CHC **5**
CHAPTER 2: Roles and responsibilities of CHC members **6**
2.1. Membership of CHCs **6**
2.2. Composition of the CHC **7**
2.3. Interaction of CHCs with community members and groups **8**
2.4. Interaction of CHCs with MSPP **8**
CHAPTER 3: Developing Situational Awareness about Malaria **10**
Activity 1: Develop a vision for your CHC **11**
Activity 2: Clarify outstanding questions CHC members may have on malaria . **13**
Activity 3: Generate a community map of malaria risk factors **15**
Activity 4: Discuss strengths and weaknesses of malaria control interventions ... **17**
CHAPTER 4: Developing a community action plan **19**
4.1. Planning your routine interventions **19**
4.2. Creating a rapid malaria outbreak response plan **28**
4.3. Creating a material and resource plan **32**
CHAPTER 5: Implementing the community action plan **36**
5.1. Start small and then expand **36**
5.2. Create a regular communication channel with MSPP **36**
5.3. Be creative! Incorporate Haitian arts, music and culture **36**
5.4. School activities **36**
5.5. Environmental cleanup **37**
5.6. Follow five principles to engage all sectors of society **37**
CHAPTER 6: Establishing neighborhood CHC groups **38**
6.1. Select CHC volunteer leaders in each neighborhood **38**
6.2. Organize a network of neighborhood CHC volunteers **39**
6.3. Officially launching a neighborhood CHC group **41**
6.4 Very active neighborhood CHC volunteer groups **41**
6.5. Implementing interventions outside areas with CHC groups **42**
6.6. Working with MSPP and other medical staff at the neighborhood level **42**
6.7. Initiating the rapid response plan with CHCs volunteer groups **42**
CHAPTER 7: Supervision and Learning **44**
7.1. Prizes and evaluation **44**
7.2. Program supervision and reporting **44**
7.3. Community feedback research activities **45**
7.4. Malaria data from MSPP **45**
7.5. Learning and adapting **45**
7.6. Adapting your CHC beyond *malaria* to other health problems **45**
Annex **46**

How to use this manual

This manual is a step-by-step guide to establish and run community health councils (CHCs) in Haiti.

It includes guidance for CHC members, Ministère de la Santé Publique (MSPP) staff and other implementing partners.

The manual should be distributed to CHC members as well as all MSPP staff involved in supporting the program.

A series of workshops and trainings should be organized by MSPP and implementing partner staff with CHC members in order to introduce the various sections of the manual, ensure the components of the program are fully understood, and to facilitate the various group activities described in the manual.

The manual includes 7 Chapters.

Chapter 1 introduces the concept of the CHC.

Chapter 2 outlines the roles and responsibilities of CHC members.

Chapter 3 outlines four activities CHCs should do to strengthen their situational awareness about malaria.

Chapter 4 provides step-by-step instructions for developing CHC Action Plans, including routine interventions, rapid response plans, and a material and resource plan.

Chapter 5 discusses a number of key considerations in implementing the CHC Action Plans.

Chapter 6 provides step-by-step instructions for establishing neighborhood CHC groups as a part of implementing the CHC Action Plan.

Chapter 7 outlines important components of supervision and learning that are needed to ensure successful implementation and functioning of CHCs.

The Annex provides the program organogram and a series of report templates that should be filled out by CHC members as they go through the manual and complete the 11 Activities that are provided. An Activity Checklist is also provided.

CHAPTER 1: The Community Health Council Approach

1.1. What is a Community Health Council?

A community health council (CHC) is a voluntary organization run by local residents to act on behalf of its area by promoting health and positive social change. CHC members focus on carrying out education and behavior change campaigns, assisting with community disease surveillance activities, helping to strengthen links between the community and medical professionals, and the mobilization of local residents in disease prevention in their neighborhoods and whole community. These forms of community councils were common in Haiti in the 1970s and 80s, especially in rural areas. The CHC model outlined in this manual aims to revive past community participatory involvement in local health issues. CHCs are organizations comprised of local people who care about their community and want to make it a better place to live. We believe that this model of community empowerment has a role to play in strengthening primary healthcare in Haiti today, and in the fight against malaria (and other infectious diseases) in the country.

The primary goal of the CHC program is to empower individuals, families and communities in Haiti to strengthen local action for health and social change in ways that directly assist in the elimination of malaria.

The specific four objectives of the CHC program are to:

1. Strengthen the well-being and health of communities;
2. Strengthen community capacity and competency for planning and engagement in health issues;
3. Bring local people together to plan and engage in the elimination of malaria;
4. Provide a community platform for the vector-borne disease control and elimination programs of the (MSPP) in Haiti.

The initial establishment of CHCs have a special emphasis on malaria prevention and control activities. This is not an exclusive focus. Once CHCs are functional and have carried out activities for 6-12 months, we encourage them to define other health priorities that they may want to work on in their community and to use the tools, guidance, and protocols outlined in this manual to expand their efforts to address these other important problems. Guidance on how to do so is outlined in the final chapter of this manual.

The CHC approach was established by The Carter Center, which leads community engagement for the Malaria Zero alliance[1], to support MSPP's malaria elimination efforts

[1] Partners include the Ministry of Public Health and Population of Haiti, the Ministry of Public Health and

in Haiti. The conceptual model for CHCs was developed with assistance from colleagues at the University of Amsterdam (see: Pool, de Vries and Bardosh (2018) *Malaria Zero: Community Engagement Plan*) and refined through formative research (see: Druetz et al. (2018). "Wherever doctors cannot reach, the sunshine can": overcoming potential barriers to malaria elimination interventions in Haiti. *Malaria Journal* 17(1), 1-11). This manual takes into account learning from this work as well as an initial pilot phase of the CHCs in Grand Anse Department.

1.2. Why are CHCs important for malaria elimination?

Malaria is caused by a parasite and spread by mosquitoes. The disease is widespread in Grande Anse department. It is also a disease that MSPP and other international partners have targeted for elimination. Haiti and the Dominican Republic are the only remaining countries with malaria transmission in the Caribbean. To reach the goal of malaria elimination, and to protect communities and future generations from the threat of malaria in Haiti, MSPP and implementing partners cannot work in isolation from local communities and community-based organizations. The whole community must be engaged in the fight.

The CHC program provides an avenue for community members and leaders to organize and empower local citizens to become involved in the battle against malaria and to work in collaboration with MSPP.

According to the National Strategic Plan (2016-2022) of MSPP, the battle against malaria has six pillars; many will require direct involvement of the CHCs:

1. **Strengthening the management of malaria cases:** the signs and symptoms of malaria are similar to many other illnesses and diseases, and many people do not seek care at health facilities until it is too late. This leads to higher chance of spread in the community and also mortality from the disease. CHCs can help support MSPP health facilities, private clinics, and community health agents (especially in rural areas) in their efforts to diagnose and treat malaria.

2. **Developing a distribution and management system for diagnostics and treatments.** This is important to ensure that supplies are available throughout the country.

Social Assistance of the Dominican Republic, the U.S. Centers for Disease Control and Prevention, the CDC Foundation, the Pan American Health Organization, The Carter Center, the Clinton Health Access Initiative, the London School of Hygiene & Tropical Medicine, and Tulane University School of Public Health and Tropical Medicine

3. **Strengthening epidemiological surveillance.** Tracking malaria cases is very important in order to know where the disease is spreading and whether anti-malaria activities are working.

4. **Strengthening vector control interventions.** An important part of malaria control has to do with the control of mosquitoes and environmental sanitation, which includes the reduction of stagnant water sources with a high density of mosquitoes.

5. **Strengthening the national health system.** Fighting malaria will require developing and strengthening the ability for individuals, families, community groups, health staff, and local governments to take action. CHCs are tasked with mobilizing citizens to address barriers and challenges, and to help find local solutions that strengthen the health system to fight malaria. Sometimes MSPP will organize specific malaria interventions; this may include mass distribution of bed-nets, anti-malaria testing, anti-malaria drugs, the spraying of houses with chemicals, and the use of chemicals in stagnant water sources. These will only occur in areas of the highest malaria transmission and will depend on external funding. The CHCs should assist with their implementation.

6. **Strengthening IEC (information, education, and communication) within the community.** Raising awareness about malaria signs and symptoms, treatment, and prevention are absolutely essential to promoting individual and household behaviors and practices that fight malaria and other diseases.

It is fundamental that CHC members have a strong understanding of malaria as they plan and carry out activities associated with the six pillars of malaria control. All CHC members should familiarize themselves with the **Malaria Education Material** provided by MSPP. This material will be provided separately from this manual.

1.3. CHC activities and scope of work

<u>The Community Health Council will:</u>

1. Work with MSPP to take local ownership of malaria elimination activities.
2. Represent in the community the interest (voice) of the Ministry of Health and implementing partners during their scheduled community meetings.
3. Organize and realize a meeting of the CHC members once per month.
4. Rapidly respond to any new malaria cases in the community.
5. Organize and realize at least 4 community interventions per month, including sensitization and prevention activities, so as to increase awareness about malaria in the community.

6. By sensitizing the community, increase demand for malaria treatment by the local population at both health facility and community levels, as well as increase adherence to treatment dosage and follow-up.
7. Assist in developing strategies and messages relevant for malaria elimination in each community.
8. Participate actively as messengers and organizers of the activities aligned with the malaria elimination program within the community.
9. Promote use of personal protection against mosquitoes.
10. Be available to help with all anti-malaria interventions organized by MSPP.
11. Report back on activities of the group on a monthly basis to the health communal level.

Once a CHC becomes very active, it can graduate to tackle problems beyond only malaria. Each CHC should make this decision in collaboration with MSPP and partners.

1.4. Support and assistance provided to CHCs

CHCs are voluntary organizations. MSPP and implementing partners do not provide salaries or per diems to CHC members. CHCs should be self-sustaining community groups that mobilize local citizens to improve population health based on a spirit of solidarity and compassion.

MSPP and implementing partners will provide support in establishing CHCs and in some aspects of their functioning and supervision. MSPP and implementing partners may provide some material support such as T-shirts, badges, educational material, planning tools and cleanup materials. There may be some support to travel to official meetings with MSPP but not for the daily running of the CHC. This will vary over time and will need to be discussed and agreed in advance with MSPP and implementing partners.

1.5. Sequence of steps in establishing a CHC

The steps involved in establishing and running a CHC include:

- Deciding membership and organizing roles and responsibilities
- Maintaining member participation
- Developing a shared vision for your CHC
- Conducting a health needs assessment
- Creating a volunteer workforce
- Developing community action plans
- Implementing community action plans
- Learning and adapting

These are described in the remaining chapters of this manual.

CHAPTER 2: Roles and responsibilities of CHC members

2.1. Membership of CHCs

The first step in selecting CHC members is for:

1) The MSPP departmental health office in agreement with local health officials to call a meeting with all sectors within the sub-commune or town, depending on where the CHC will be set up. Local authorities (mayors, CASEC, ASEC, justice department, local governor), representatives of local community-based organizations, natural leaders (pastors, priest, ASCPs (Polyvalent Community Health Agents), traditional healers) should be invited to the meeting. Roughly 40-50 people should participate, from different geographical areas of the sub-commune.
2) MSPP and partners leading the meeting should present the main objectives of the CHCs and the importance of selecting a wide range of stakeholders to be members.
3) They should then give a brief presentation on malaria and how community engagement can improve life conditions within their community.
4) Carefully, the attendees should be subdivided into different social groups considering a balance of gender, age, socio-economic status, and professional background.
5) The selection process should be done in an open and transparent way. The goal should be to select people that are interested and are committed. This should allow for the inclusion of people from different geographical areas and social groups, which should be included in the council without prejudice.
6) Training session should then be planned and conducted to provide them more knowledge on malaria and other diseases, and on how to ensure everyone living within the community can be engaged in the work to improve health and livelihoods.
7) Afterwards, environmental cleanup supplies should be delivered so the CHC can promote behavior change (if the resources exist to do so).

There should be between 9 and 13 CHC members who represent all sectors of society: farmers, women, religious leaders, youth, local organizations, teachers, health staff, business leaders, etc.

Each CHC member should:

- Be non-sectarian and non-political.
- Be selected by the communities so that CHCs represent a full cross section of the community without discrimination.
- Have very good reputations at the community level.
- Be able to work with a spirit of voluntarism and compassion.

- Be accepted by the people of the locality.
- Reside within the community.

It is very important that CHCs not be affiliated or associated with any political parties to ensure that they are not used for political purposes and are seen as neutral councils working for the benefit of all members of the community.

2.2. Composition of the CHC

The community council is composed of representatives coming across the communal section. Within the community council members, a board of directors of 4 people will be selected to lead and coordinate actions of the council for two years. After these two years, each CHC will select a different board of directors. This should involve a process of nomination and vote by each CHC members. The board of directors is composed of the following members:

- One Coordinator or President
- One Secretary
- Two delegates or advisors

Role of the Coordinator/President

- Chair the committee meetings and represent the committee in all activities;
- Convene routine and extra general meetings;
- Prepare the agendas;
- Prepare and present the annual reports of the activities after sanction of the members of the committee during the general assemblies;
- Regularly submit the committee's activity report to MSPP;
- Ensure the proper functioning of the committee;
- Ensure that action plans are achieved successfully;
- Ensure links between the committee and other sectors of the community;
- Attend any meetings organized by MSPP.

Role of the Secretary

- Assist the coordinator to draft meeting agendas;
- Write and keep the correspondences of the committee;
- Be responsible for recording the minutes of each meeting;
- Maintain the list and the files of the CHC members and community volunteers;
- Write the annual report in collaboration with the coordinator;
- Sign all official documents of the committee with the coordinator.

Role of the two Advisers/Delegates

- Encourage other members of the committee to participate in mobilization activities;
- Ensure the proper functioning of the committee, including during meetings and in community outreach activities;
- Promote the mission of the committee within the community;
- Facilitate the resolution of conflicts;
- Encourage other members to pursue the committee's goals.

Responsibilities of all members

Aside from the roles and responsibilities listed above, all members are expected to:

- Actively participate in all agreed upon CHC activities;
- Promote the mission of the committee within the community;
- Raise awareness about the CHC in the community;
- Attend CHC meetings;
- Participate in sub-committee meetings and activities as agreed upon.

2.3. Interaction of CHCs with community members and groups

CHCs will function as mobilizing agents in their communities and work at a sub-commune level. They will need to work in collaboration with a broad range of community volunteers and groups to implement anti-malaria and other health interventions. They will need collaborators and support from others!

For this reason, guidance is provided in this manual on how CHCs should go about establishing a CHC volunteer network, including guidance on how and when CHCs should establish *neighborhood CHCs* to implement interventions at a local level.

It may also be a good idea to invite community groups, community leaders and community members to CHC monthly meetings.

2.4. Interaction of CHCs with MSPP

CHCs will maintain regular contact with MSPP and other implementing partners. It is expected that CHCs:

- Communicate activity reports on a monthly basis to MSPP;
- Meet deadlines for other activities as agreed upon;

- Participate in monitoring activities organized by MSPP;
- Rapidly communicate any problems or changes in the management of the CHC to the MSPP team by phone.

MSPP with support from other implementing partners commit to:

- Support the CHCs with technical support and guidance;
- Provide skills training to support the functioning of the CHCs;
- Provide educational material for community sensitization and mobilization;
- Provide some clean-up materials for environmental sanitation interventions (when possible);
- Provide small incentives (when possible) including an annual prize for CHCs;
- Monitor and supervise the activities of the CHCs;
- Listen to and respond to concerns and challenges.

CHCs, MSPP, and other implementing partners will work in a collaborative and participatory way that is open to new ideas and willing to adapt to meet challenges and generate new solutions for the control and elimination of malaria and other public health problems in the community.

CHAPTER 3: Developing Situational Awareness about Malaria

In this chapter, we describe four exercises that CHCs should do in the **first month** of establishing their CHC. **These activities should be done during the same CHC planning meeting.** This will be a long CHC meeting, but it is important that Activities 1, 2, 3, and 4 are completed together. Make arrangements for this meeting and ask CHC members to be familiar with them ahead of time.

This work will help CHC members better define their values as a group and ensure that they have a unified understanding of malaria. This will be important as they develop specific plans for interventions in their communities.

It is important that MSPP and the board of directors read through the instructions for each activity and create a plan for doing these activities with the CHC members during their regular meetings.

The steps are as follows:

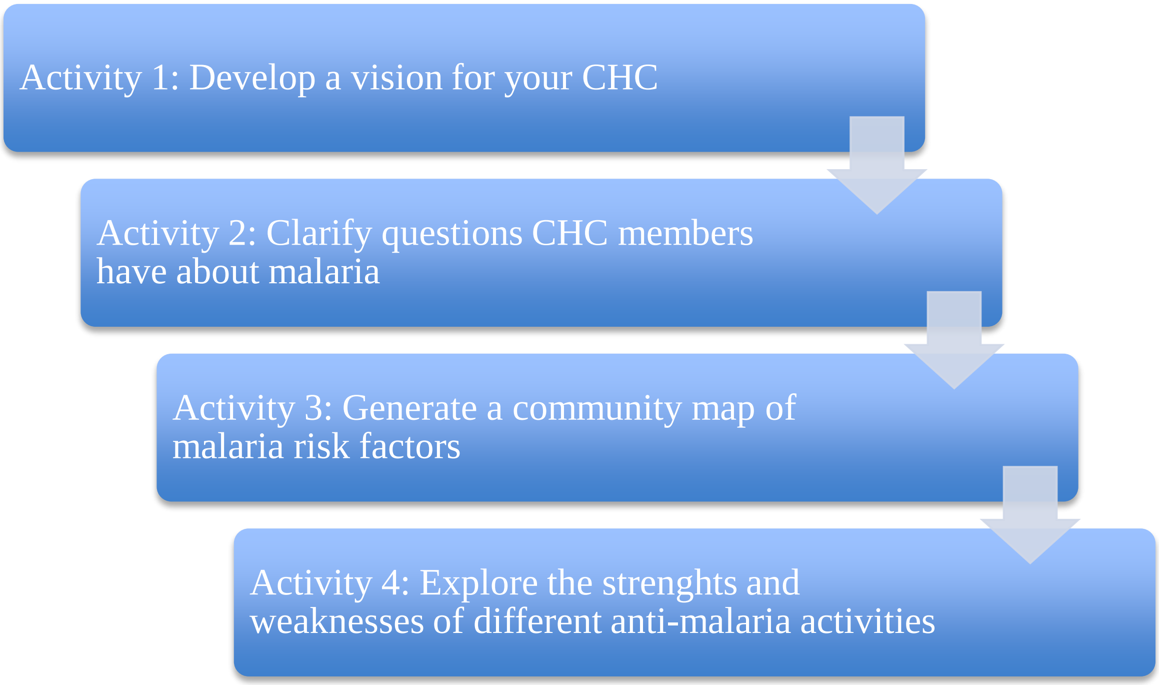

Activity 1: Develop a vision for your CHC

Once you have established your CHC, members should discuss the goals and vision they have for the council. To do this, the board of directors should facilitate an open discussion among the CHC members. This should follow the step-by-step guidelines described here in **Activity 1** below. In this activity, the CHC will develop a name and slogan for the CHC and agree on a code of conduct.

Activity 1: Developing a vision for your CHC

Objective: To generate a consensus and team spirit within the CHC and determine the location and date/time of future meetings.

Materials needed: Paper and pens.

Time required: 30 minutes

Activity description: The group will do Activity 1 during one of their routine monthly meetings. The CHC should:

1. Break into smaller sub-groups of 2-3 people each.
2. Each group should think about a name for the CHC and a slogan.
3. After 15 minutes, the group should reconvene, and each sub-group should share what they came up with. The whole group should then agree on a name for the CHC and a short slogan.
4. Afterwards, the group should discuss amongst themselves the expectations they have for the group. They should start with the following two questions: 1) What do they want to accomplish over the next year? And 2) How much time can each member dedicate to CHC activities each month?
5. One of the board of directors should then lead a discussion on the roles and responsibilities of CHC members.
6. The CHC should agree to a regular meeting day/time and location for CHC meetings.
7. During this discussion, they should read the roles and responsibilities listed in Chapter 2 for each member.
8. Afterwards, the CHC should develop a code of conduct that clearly articulates the values of the CHC and their working spirit.

Output: Once this activity is finished, the CHC should have an agreed upon: 1) name; 2) slogan; and 3) code of conduct. This should be recorded by the CHC Secretary and a copy of the agreed upon name, slogan, and code of conduct should be communicated to the MSPP focal person, following the format given in **Activity Report 1.**

Activity 2: Clarify outstanding questions CHC members may have on malaria

Malaria is a complicated disease and community members will have many questions regarding specific aspects of the disease during CHC interventions. It is very important that all CHC members have a strong comprehension of malaria transmission, diagnosis, treatment, and prevention as well as MSPP strategies to eliminate malaria.

For this reason, CHC members should hold an open discussion amongst themselves about specific questions they may have regarding malaria – things that remain unclear to them or areas where they would like more scientific information. To do this, each CHC should follow the instructions in **Activity 2.**

It is important that the facilitator remind the CHC members that no questions are bad questions. They should not feel embarrassed to ask any question. This is a time to clarify any misunderstandings or areas where members lack knowledge about malaria.

Activity 2: Develop a list of outstanding questions CHC members have regarding malaria

Objective: To develop a list of specific questions that CHC members have regarding malaria that they would like answered.

Materials needed: Paper, pen, and blackboard or flipchart.

Time required: 45 minutes

Activity description: The group will do **Activity 2** during one of their routine monthly meetings. The activity will be conducted in the following sequence:

1. CHC group members will be asked: "what questions do each of you have regarding malaria transmission, diagnosis, treatment, prevention, and elimination that you would like to know more about?"
2. The facilitator will allow each CHC member to individually write down any questions they have on a piece of paper. They should leave 5-10 minutes to do this.
3. Once everyone is done writing their questions down, the facilitator should ask the group to read their questions.
4. The facilitator should write down all of the questions mentioned by the CHC members on a blackboard or large flipcharts. All questions should be written down without interruption and members should not answer any questions at this stage. It is first important that all questions be written down before the group tries to answer some of the questions.
5. Once all of the questions are written down, the facilitator should go through the list of questions in order and ask if any members can answer them.
6. They should write down the answer to each question and should mark any questions that the CHC members cannot answer or areas where they feel that the answer is incomplete.
7. Before finishing, the facilitator should then ask the group if they have any other questions regarding malaria that they would like further information about. This should be added to the list.

Output: Once this activity is finished, the CHC should report back to MSPP with a list of questions and answers to these questions following the format of **Activity Report 2**. This will be used to help MSPP and partners design education material for the CHC.

Activity 3: Generate a community map of malaria risk factors

To control malaria, we need to be able to diagnose and treat people sick with the disease, and we also need to reduce contact between people and mosquitoes.

It is important that **CHCs understand the level of capacity their local health system has to fight malaria and the key environmental and socio-economic drivers that help spread the disease.** This will involve mapping hotspots for mosquitoes and visiting their local health clinics to ask about their capacity to diagnose and treat malaria cases.

This exercise will begin to establish a relationship between the CHCs and local health clinic staff, and it will also generate important information about the health system's capacity for malaria control and mosquito hotspots in the CHC territory. This information will be used to strengthen and improve malaria control by the CHCs.

To do this, the CHC members should follow instructions in **Activity 3.**

Activity 3: Mapping malaria risk factors in your area

Objective: To understand barriers to malaria control as well as the root causes of the barriers at the local health system level and to map geographical hotspots for mosquitoes in the community.

Materials needed: Paper, pen, and blackboard or flipchart. MSPP supplied list of malaria cases and clinics in the CHC territory.

Time required: 60-80 minutes

Activity description: There will be two stages (A + B) to this activity.

The first stage (A) will be for CHC members to draw a map of their territory on the poster board. This does not need to be perfect. It is a rough map, but it should be done with care since it will be used in the future. On the poster board, each CHC should:

1. Start by drawing sub-commune boundaries.
2. Second, draw important natural features, such as rivers, mountains and forests.
3. Third, draw major roads.
4. Fourth, note down all MSPP clinics, hospitals, and private clinics.

The *second stage* (B) will involve adding details specific to malaria. This involves:

1. Using the list of malaria cases supplied by MSPP, put a star at the rough location of each malaria case that has occurred in the last 10 years in your sub-commune. In some cases, the information will allow you to mark the specific locality of the household while in others only the name of the health clinic that diagnoses the case will be available.
2. Once you have marked all of the cases, you will now mark down important stagnant water sources or swampy areas that you believe are major breeding grounds for mosquitoes. Mark these areas on your map.
3. Once you are finished, discuss the risk factors for malaria in your area.

Output: Once this activity is finished, you should keep the copy of your map for future use. You should take a photo of the map and share this with your MSPP supervisor. You should also bring the map to CHC meetings in the future and use it when planning your activities. The poster itself is considered your **Activity Report 3.**

Activity 4: Discuss strengths and weaknesses of malaria control interventions

Before you develop your activity plans, it is very important to familiarize yourself with the various types of interventions that each CHC can implement in their community. For this reason, it is essential that each CHC member read through the list of interventions provided here and that the CHC discuss this list together as a group.

To do this, each CHC should spend 1-2 hours reading and discussing the options. **Activity 4** will not have any specific output but will form the foundation for your Community Action Plan that is described in the next chapter. It is not necessary to write anything down, but it is important to have a serious discussion about what activities CHC members think are the most important and feasible to do in the community.

1. Spread awareness and improve knowledge about malaria		
**Type of intervention**	**Strengths**	**Challenges**
1.1 House to house visits		
1.2. Community meetings		
1.3. Community event		
1.4. School program		
1.5. Community announcements		

2. Improve surveillance and treatment		
2.1. Support people with malaria symptoms to be diagnosed and treated early		
2.2. Work with and support health clinics and hospitals (public and private)		
2.3. Work with and support traditional healers		
2.4. Work with and support community health agents		

3. Mosquito Prevention		
3.1. Promote the use of bed-nets and window screens		
3.2. Identify and monitor important mosquito breeding sites		
3.3. Engage in environmental clean-up		

4. Building community solidarity		
3.4. Work with NGOs and health clubs		
3.5. Work with local government		
3.6. Work with MSPP		

CHAPTER 4: Developing a community action plan

In this chapter, we provide step-by-step guidance on how CHCs should organize health interventions at the community level. Careful planning with the CHC members and community volunteers will be required to ensure that these interventions are successful and are acceptable to local citizens. To accomplish this, each CHC should develop and follow a **"CHC Community Action Plan" (CAPs).** This chapter outlines how to do this.

The **CAP** will have three parts:

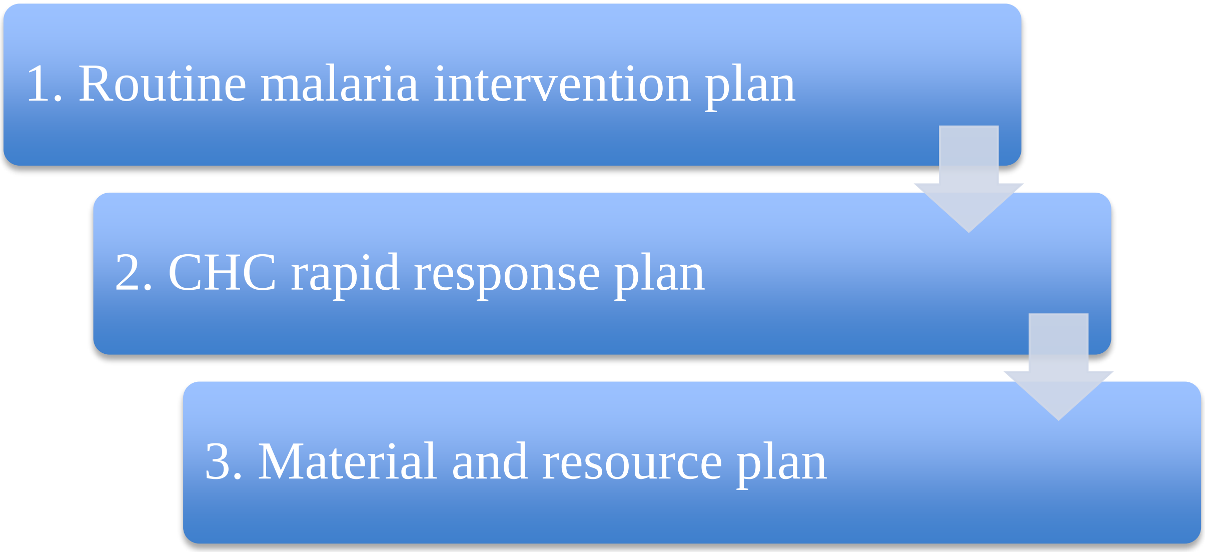

These are explained more below.

Each CHC should be creative in how they develop their Community Action Plan. They should adapt it to fit into the local community and to address concerns of citizens. CHCs should aim to generate support from different segments of the population.

Making the CAP into a reality and improving population health will require diligence, determination, and collective planning. This will need to be a team effort between the CHC, MSPP, and implementing partners. The CAP will need to be revisited and adapted on a 6-month basis.

4.1. Planning your routine interventions

Creating a plan for your routine interventions should be done together in the same CHC meeting. This will be a long CHC meeting, but it is important that Activities 5, 6, 7, 8, and 9 are completed together. Make arrangements for this meeting ahead of time and ask CHC members to read through the activities so that they are familiar with them before the meeting.

Steps involved in planning your routine interventions

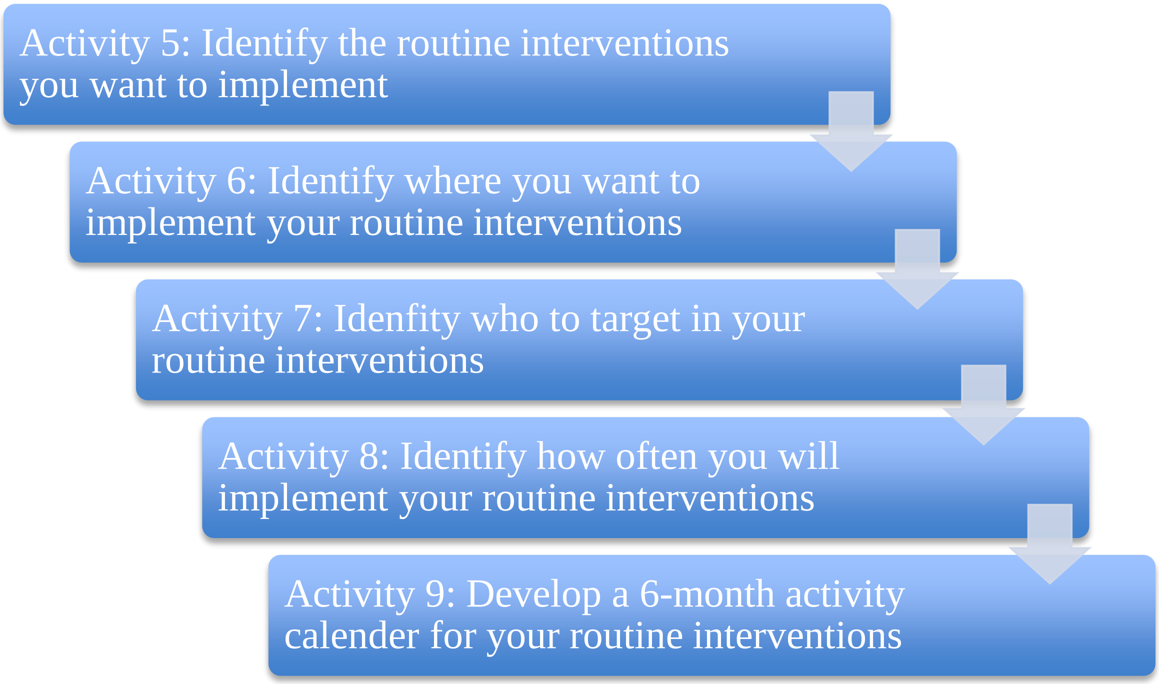

Activity 5: Identify the routine interventions you want to implement

The starting point for your CAP is to agree on a set of routine interventions your CHC will initiate in the community to raise awareness of malaria and promote prevention and control. These were listed above in Chapter 3. It is important that the CHC discuss the options for anti-malaria activities and collectively agree on a set of interventions. It is important to be very specific about what you envision doing; the more specific, the easier the planning and implementation will be.

In order to accomplish this, please do **Activity 5**.

Activity 5: Decide on your CHC anti-malaria interventions

Objective: To clearly define the interventions that the CHC plans to implement in their territory for the next 6 months.

Materials needed: Pen and paper.

Time required: 1-2 hours.

Activity description: This activity will form the beginning of the CAP. The CHC should do this activity during a CHC meeting following these steps:

1. The group should first make sure that everyone has read through the list of possible CHC interventions provided in **Activity 4** above (Chapter 3).
2. The CHC should then hold an open discussion. They should go through each of the possible interventions and discuss the pros and cons of each one.
3. They should then agree on a set number of interventions and fill out **Activity Form 5**. Remember that this can include as many interventions as the CHC would like – there is no limit other than the interest, time, and resources of the CHC.
4. Each CHC should consider forming sub-groups for some of the CHC interventions. This may assist with the planning process. This is not required but may help.

Output: Once this activity is finished, the CHC should have a list of specific CHC interventions they will implement in the community over the next 6 months. **Activity Form 5** should be filled out.

Once you have decided on your routine CHC interventions you will need to decide **where** you plan to implement these activities, **who** to target for the interventions and **how often**

you plan to do each one.

Activity 6: Identifying where to implement your routine interventions

Each CHC should prioritize neighborhoods with previous or current malaria cases for their routine anti-malaria interventions as well as neighborhoods that are considered "high risk" because of a high density of mosquitoes. **The decision of where to target your activities will need to be based on the list of malaria cases you have from MSPP.** To do this, please follow instructions for **Activity 6**.

Activity 6: Decide where to target your CHC anti-malaria interventions

Objective: To select 3-6 neighborhoods you want to target for anti-malaria activities for the next 6 months.

Materials needed: Pen and paper.

Time required: 30 minutes

Activity description: To conduct this activity, please:

1. Review the results of your Malaria Risk Factor Poster (Chapter 3).
2. Develop a list of 3-6 priority neighborhoods that are at high risk of malaria to organize their activities. This should be based on case reports given to you by MSPP. This list may also include some neighborhoods that are places of high visibility (with large population, transit hubs) where many people may hear the message. These areas should also be within reasonable travel distance to CHC members. Write these down on the **Activity Form 6.**
3. (However, it is also important to consider areas that may have malaria cases but do not report them. In effect, there may be silent malaria transmission areas. You should consider these as well.)

Output: Once this activity is finished, the CHC should have a list of 3-6 priority neighborhoods it will target for anti-malaria activities over the next 6 months. **Activity Form 6** should be filled out.

Activity 7: Who do you want to target for your routine interventions?

It is important to think about how to target your interventions to specific social groups. In some cases, you may want to target the whole community. In other cases, you may want to target specific social groups. Thinking about *who* you want to target is a helpful way to tailor your interventions. For example, this may include:

- School children and teachers
- Health workers
- Religious leaders
- Market sellers
- Youth organizations
- Community-based organizations
- Women's groups
- Income generation groups
- Local government
- Agricultural groups
- Cultural groups
- Households from remote areas

Activity 7: Decide who to target for your CHC anti-malaria interventions

Objective: Discuss who you would like to target for your anti-malaria interventions and why.

Materials needed: Pen and paper.

Time required: 30 minutes

Activity description: To conduct this activity, please:

1. Review the list of the CHC interventions you have decided to implement, according to **Activity 5**.
2. For each intervention, list any specific types of individuals you would like to target for these activities and why you think they should be targeted.
3. Write these down on the **Activity Form 7.**

Output: Once this activity is finished, the CHC should have a better appreciation of their target audience and social groups. **Activity Form 7** should be filled out.

Activity 8: How often will you implement your routine interventions?

It is important to consider how often you should organize specific routine interventions in each location. To make your planning and implementation easier, you should aim to implement the same interventions in each neighborhood. You should consider this to be a "package of interventions" that are done in every neighborhood you have selected.

Your planning must take into consideration the number of neighborhoods you have selected for your CHC interventions and the number of CHC interventions you have selected.

There are two ways to organize this:

Option 1: Each month, you select a different neighborhood to focus on. Here, you implement all of your selected interventions. Then the following month, you move to another neighborhood and so on.

For example, if you have selected 3 neighborhoods then you should aim to focus your interventions on 1 neighborhood per month. This means that you would visit all 3 neighborhoods over a 3-month period of time. Alternatively, if you selected 6 neighborhoods, then it will take you 6 months to visit all neighborhoods.

Option 2: Each month you select one type of intervention and implement it in all of the neighborhoods you have selected. Then the following month, you move to another type of intervention and so on.

For example, you have selected 3 neighborhoods and 4 different interventions: 1) house-to-house visits, 2) school program, 3) working with traditional medicine doctors and 4) environmental sanitation. In this case, the first month you would select one of these interventions (house-to-house education) and implement it in all 3 neighborhoods. Then the following month, you would select the second intervention (school program) and implement it in the 3 neighborhoods, and so on. In this case, it would take you 4 months to finish all of the interventions.

Activity 8: Decide how often you will implement your CHC anti-malaria interventions

Objective: Discuss how often you will implement your CHC anti-malaria interventions.

Materials needed: Pen and paper.

Time required: 30 minutes

Activity description: To conduct this activity, please:

4. Read through the description above in this section.
5. Clearly agree on how many neighborhoods you have selected for your interventions for the next 6 months and the number of different interventions you have selected.
6. Have a discussion with all CHC members about whether you want to follow Option 1 or Option 2 outlined above.
7. Based on this discussion, you may want to revise the total number of neighborhood and/or types of interventions your CHC has selected. This is okay and a normal part of the planning process. If so, note the change in your Activity Forms.
8. Write down your choice in the **Activity Form 8.**

Output: Once this activity is finished, the CHC will have improved its planning and organization and will be ready to develop an Activity Calendar, which is **Activity 9**. **Activity Form 8** should be filled out.

Activity 9: Develop a 6-month activity calendar for your CHC

You are now ready to complete your Activity Calendar! This will combine the brainstorming and decisions you have made in Activities 5-8 into a clear monthly calendar. Remember that each CHC should plan a minimum of five interventions per month, including the monthly CHC meeting. You will have two options on how to do this:

Option 1: Create a 6-month activity calendar with clearly defined dates and locations for each intervention. This should include 1 CHC meeting per month and a minimum of 4 interventions (you can certainly plan more than 4 per month, if you decide to – this is up to each CHC). The benefit of this option is that it allows you to have all of your plans decided ahead of time.

Option 2: Create a 6-month activity calendar, but with the dates defined on a month-to-month basis. In this option, you decide on the monthly date, time and location of your CHC meetings 6 month in advance, but you only plan your interventions the month before. You will need to agree on specific dates and times each month at your CHC meeting for the next month. The benefit of this option is that it allows you to have flexibility with the exact date and time of your plans.

The schedule should consider seasonal influences on malaria transmission. As the rainy season begins, the CHCs should alert people of the high season and promote preparedness activities to prevent widespread transmission

Activity 9 involves each CHC discussing these 2 options and choosing one. **There won't be an activity report for this activity. Rather, each CHC member will be provided with a monthly calendar to write the official calendar in**.

Scheduling CHC interventions should account for the:

- Day and time of the intervention.
- The location of the intervention
- The name of the intervention (type of intervention)
- The name of any CHC members that are responsible for leading activities in the field, if this is the case.

Each CHC member will be provided with a monthly calendar booklet to write the official calendar in. They should also communicate the information of each planned intervention with community members from each neighborhood in advance so that local community members and CHC volunteers are ready.

Each CHC should also write down important market days, festival days, holiday events, and days where malaria interventions are planned by MSPP and implementing partners (such as World Malaria Day) that are important to the community in their calendar booklet. They should aim to include some interventions during these important days.

It is very important that each CHC have a very clear plan for when they will implement their interventions. This will allow them to contact the appropriate CHC volunteers and plan any educational material or other necessities.

4.2. Creating a rapid malaria outbreak response plan

The **CAP** has three parts. So far, we have concentrated on routine interventions. But the CHC also needs to have a different plan – a plan for how to respond to cases of malaria or an epidemic of malaria in the community. This is called your **CHC rapid response plan.**

The CHC rapid response plan should be discussed at a CHC meeting.

Activity 10: Developing a CHC rapid response plan

The CHC rapid response plan is a set of interventions that the CHC has agreed to implement in neighborhoods where a malaria case, or many malaria cases, has been diagnosed. The rapid response plan will be initiated in collaboration with MSPP. It is a set of emergency activities to limit the spread of malaria in the community.

The plan should involve the following interventions:

- Spread awareness about malaria and increase knowledge in the local community.
- Assist with surveillance and treatment in the local community.
- Assist with mosquito prevention in the local community.
- Build community solidarity.
- Maintain long-term surveillance and support to monitor future malaria cases.

Depending on the circumstances and the discussion with MSPP, the CHC may need to take a very active engagement to ensure that people in the community with malaria-like symptoms are quickly diagnosed and, if positive for the disease, treated by health staff.

CHC members need to develop their own set of interventions that they plan to implement as part of the CHC Rapid Response Plan. Follow the instructions for **Activity 10** and agree on a CHC Rapid Response Plan.

This plan should cover:

- An intensive phase of activities over 2 weeks aimed at locating any additional malaria cases.
- An additional phase of activities over a 3-month period.
- A long-term phase of 1 year. This phase will involve incorporating this neighborhood to the target neighborhoods for your routine interventions.

Activity 10: Agree on a CHC Rapid Response Plan

Objective: Agree on a plan and set of CHC anti-malaria interventions to respond to active malaria cases.

Materials needed: Pen and paper.

Time required: 1 hour

Activity description: To conduct this activity, please:

1. Read through the description above in this section.
2. Review the list of CHC interventions in Chapter 3.
3. Discuss the benefits and challenges of each intervention in responding to a case of malaria.
4. Discuss which interventions should be prioritized for the intensive 2-week period.
5. Discuss which interventions should be prioritized for the 3-month period.
6. Use **Activity Form 10** to guide you.

Output: Once this activity is finished, the CHC will have a completed CHC rapid response plan. This should be written in **Activity Form 10** and should serve as the basis for any rapid response activities.

Activating and implementing the CHC rapid response plan

Once MSPP has communicated about a malaria case in your territory, you should activate your CHC rapid response plan:

Steps involved in activating the CHC rapid response plan

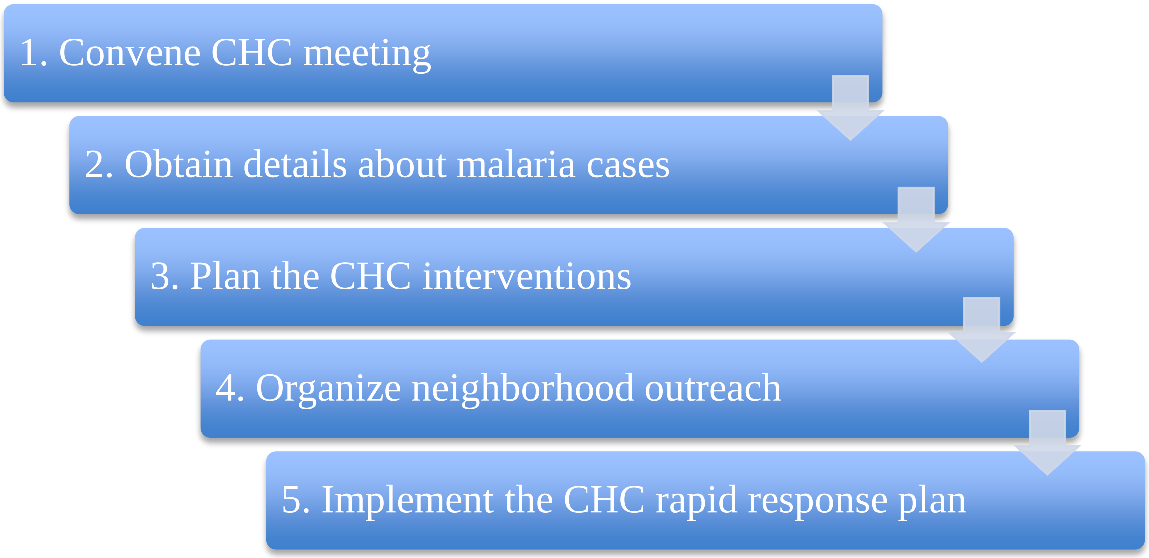

- **STEP 1: <u>Convene a CHC meeting as soon as possible.</u>** At this meeting, invite MSPP staff; organize the meeting in the community.

- **STEP 2: <u>Know the details of the malaria case.</u>** Before the meeting, find out the following information:
  - How many cases of malaria have recently been diagnosed in this area?
  - What are the details of the cases – their age, sex, occupation, and location of residence?
  - What is MSPP planning to do to address this malaria outbreak?

- **STEP 3: <u>Plan the CHC interventions at the meeting.</u>** Do this in collaboration with the local MSPP staff and community representatives and civil society groups

- **STEP 4: <u>Organize neighborhood outreach near the malaria case.</u>** The first intervention should always be a meeting at the neighborhood level where the malaria case lives with community members. At this meeting, the CHC should aim to mobilize the local population to engage in the planned CHC interventions. A local group of leaders and interested citizens should be organized. This group should be called the "Neighborhood CHC Rapid Support Team" or something similar to it.

- **STEP 5: Implement your CHC Rapid Response Plan,** working with the Neighborhood CHC Rapid Support Team. This will need to include deciding on the frequency of these activities and creating a work calendar. As with routine interventions, scheduling these rapid interventions should account for the:
  - Day and time of the intervention.
  - The location of the intervention
  - The name of the intervention (type of intervention)
  - The name of any CHC members that are responsible for leading activities in the field, if this is the case.

  When planning these activities, you should also consider where you plan to implement them, who you aim to target, and how often you plan to implement each intervention in the community, as discussed above for the routine interventions.

4.3. Creating a material and resource plan

The third and final part of your CHC Community Action Plan is a **Material and Resource Plan.** To do this, follow instructions for **Activity 11** and the steps below. This should be done at a CHC meeting.

CHCs are volunteer organizations. They depend on CHC members and volunteers to engage in the fight against malaria and to work to improve health in their communities. To achieve the goals of your CHC, it is important to discuss and plan for materials, supplies and equipment you may need.

Remember, MSPP and partners may not be able to meet your requests, or they may only be able to partially meet them. Discuss this with your MSPP focal person. Regardless, it is important plan in advance so that you may implement your interventions effectively.

Activity 11: Create a material and resource plan

Objective: Create a material and resource plan for CHC interventions.

Materials needed: Pen and paper.

Time required: 1-2 hour

Activity description: To conduct this activity, please:

1. Read through the 3 steps described below and follow the instructions.
2. Use **Activity Form 11** to guide you.

Output: Once this activity is finished, the CHC will have a completed a material and resources plan. This should be written in **Activity Form 11** and should be continuously reviewed and updated. A record should be kept.

Step 1: Requesting educational material

Educational material will be an important part of most of your CHC interventions. Working with your MSPP focal person, you should review the available malaria education material, as well as any other health promotion material that may be available for you to distribute (on other diseases, for example). Ask the MSPP focal person to bring a copy of each material and discuss how many of each you can receive every month.

**Educational material**	**Month 1**	**Month 2**	**Month 3**	**Total requested for 3 months**
Pamphlets:				
Fliers:				
Posters:				

Make a 3-month plan for supplies with your MSPP focal person so that you can ensure that you always have enough material. Use the format above.

Afterwards, and especially if there is a lack of available material, consider making and printing your own educational material. These do not need to be fancy. Discuss this with your MSPP focal person.

Step 2: Requesting supplies and materials

For your CHC to function you will need some basic supplies and materials. This may include three categories:

A. **<u>Planning supplies:</u>** books, paper, pens/pencils, posters, etc.
B. **<u>Implementation material:</u>** T-shirts, badges, school program kits, environmental clean-up kits, etc.
C. **<u>Special disease control equipment:</u>** malaria diagnostic tests, malaria drugs, etc.

<u>A. Planning supplies:</u>

These supplies should be used by the CHC to plan and organize interventions and meetings, following the instructions provided in this manual. Supplies will depend on the resources available from MSPP and implementing partners.

The following will be provided to the CHC:

- A book to be used as an attendance book
- A book to be used as a CHC volunteer network
- A yearly calendar booklet for planning all interventions
- Two poster boards.

Each 3 months, the MSPP focal person will supply (if possible) the CHC with:

- A book for each CHC member, with yearly calendar
- Two pens for each CHC member

Other resources that MSPP and implementing partners may include (depending in financial resources available):

- Airtime for each member
- An allowance for transport
- Drinks for meetings

<u>B. Implementation materials:</u>

Implementation materials are supportive materials that CHCs can use in conducting interventions in the community. These will need to be discussed with your MSPP focal person to see if resources are available. Nevertheless, it is important that you consider possible implementation material that you would like to obtain. These can include T-shirts, badges, school program kits, environmental clean-up kits, and other things. Discuss these with your MSPP focal person.

C. Special disease control supplies, material, and equipment:

Although CHC members will not use diagnostic treatments and chemicals, they may help local medical and public health staff take note of shortages or special needs, and act as advocates for their communities. In this regard, CHC members are encouraged to discuss important shortages and needs with their local medical colleagues and to discuss these requests with their MSPP focal person.

Any shortage or stock-out of malaria diagnostic tests or drugs in local clinics and hospitals should be communicated to the MSPP focal person as soon as possible. This may then be added to the requests to MSPP.

Step 3: Defining community resources and in-kind contributions

It is important that CHCs not rely exclusively on MSPP and partners for all supplies or resources needed to be effective in their community. Instead, CHC members should think about how they can draw on resources from existing community groups, government, and civil society.

For example, this could include clean-up equipment and staff from local government, schoolrooms for community gatherings, and food or other supplies from local NGOs. One important in-kind contribution is the use of a blackboard or flipchart for CHC meetings; this should be negotiated with a local school or other building. Community resources and in-kind contributions should be discussed for every intervention and negotiated with appropriate CHC volunteers and other community members.

CHAPTER 5: Implementing the community action plan

In this chapter, we briefly review some of the principles that will be important for your CHC to be successful as you implement your CAP.

5.1. Start small and then expand

It is very important that your CHC implement activities in a way that maintains motivation of its members. The CHC should not be overwhelmed, but it should concentrate on starting small, implementing successful activities, and then expanding. It should learn as it develops and use this learning to strengthen itself.

Each CHC needs to develop its own "style" of working.

It is important to focus on malaria cases and high-risk areas with a history of malaria cases.

5.2. Create a regular communication channel with MSPP

You should establish a strong and regular contact with your local MSPP clinical facility. This MSPP clinical facility should provide your CHC with monthly reports of malaria cases. If a clinic in your area does not report suspected, confirmed, and/or treated malaria cases, the CHC should discuss how to have them report these figures on a monthly basis with MSPP.

Each monthly meeting should start with an epidemiological update, provided by MSPP.

5.3. Be creative! Incorporate Haitian arts, music and culture

It is important that you encourage creativity in the ways you organize and implement interventions. Specifically, CHC members should be encouraged to take initiative, come up with new and exciting ideas, and mobilize communities in ways that draw on the unique strengths of Haitian culture and community ideas. This includes slogans, songs, dances, theater and drama, comedies, murals, poems and visual arts. Make things fun and exciting.

5.4. School activities

Organizing education and activities with school children, including engaging children in environmental cleanup and the spreading of malaria awareness and information, is an important opportunity. Each CHC should consider including school-based activities as

part of their routine and rapid response plan. Get school leaders and teachers involved and include them in the planning.

5.5. Environmental cleanup

Environmental cleanup is a big activity that requires a lot of work. It is important to consider the balance between this large amount of work and the benefit to anti-malaria efforts and broader community health. It is important to pick the most important mosquito breeding sites to focus on; otherwise the task is overwhelming.

It is important to realize that not all environmental cleanup is effective against the *Anopheles* mosquitoes that spread malaria. Other mosquito species are also found in the community but breed in different habitats compared to *Anopheles*. For example, *Aedes* mosquitoes spread Chikungunya, Zika and Dengue. *Culex* mosquitoes spread filariasis.

Each CHC should discuss their environmental cleanup activity plans with MSPP to make sure their efforts will be beneficial to the control of mosquitoes. They should consult the educational material provided that discusses the differences between *Anopheles*, *Aedes* and *Culex* mosquitoes and what this means for planning environmental cleanup.

5.6. Follow five principles to engage all sectors of society

The success of CHC interventions will depend on following the 5 principles outlined below to engage community members. CHC members should not act like experts; they should be **facilitators** that motivate community members to become involved. They should aim to **ignite enthusiasm and energy**. They should build trust and friendship with people. They should not be harsh or reprimanding. They should be truthful and not mislead people or create false expectations of support or benefits for participating. They should appeal to people's sense of citizenship and duty to one another.

Implementing the CHC CAP should be based on five principles:

1) Honesty: CHC members should act with integrity and always tell the truth.
2) Listening: CHC members should listen and show empathy to community concerns, ideas, and beliefs.
3) Partnership: CHC members should create strong partnerships between the CHC and community groups.
4) Transparency: CHC members should always provide the full picture to people and not hide anything.
5) Problem solving: CHC members should aim to work with community members to solve problems.

CHAPTER 6: Establishing neighborhood CHC groups

As mentioned throughout this manual, CHC members need to work in strong collaboration with community members, especially in malaria hotspots. This chapter describes how to do this.

Steps involved in establishing neighborhood CHC groups

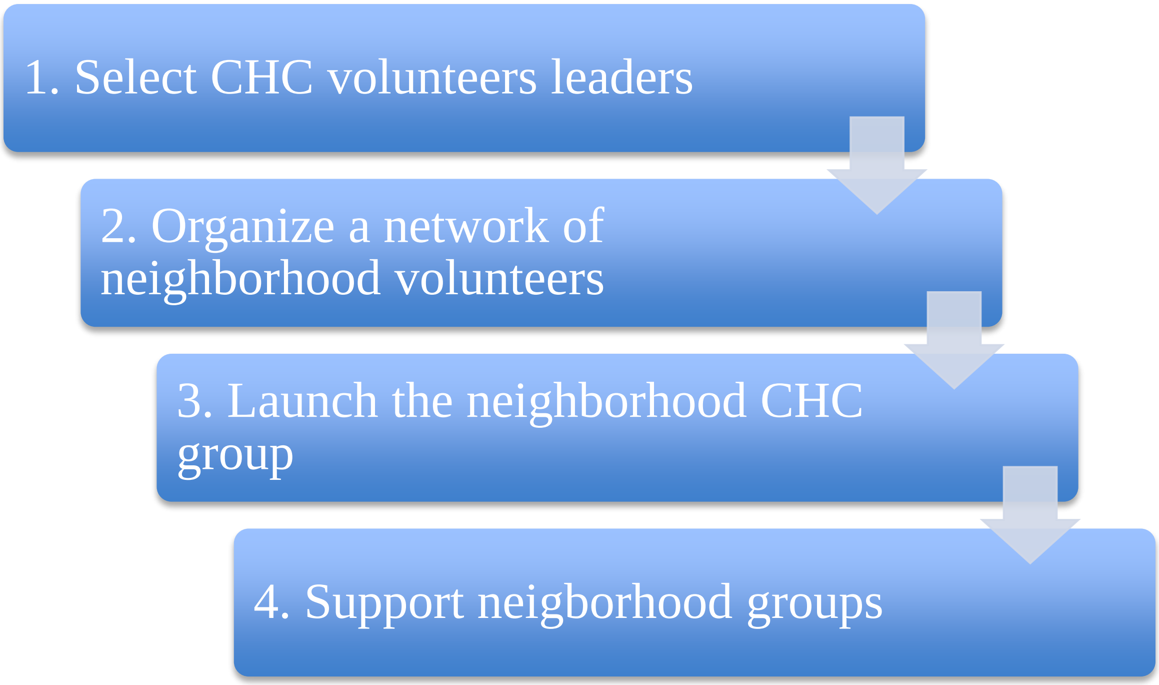

6.1. Select CHC volunteer leaders in each neighborhood

In the selected neighborhoods where your CHC will work, it is important that you establish a group of volunteers. This should consist of people who live in the neighborhood and are dedicated to work with the CHC in implementing interventions in their neighborhood. This group should be called a: **Community Health Council Volunteer Group** (or something similar).

Remember, the most important work of CHC members will be to mobilize and energize groups of CHC volunteers to become engaged in CHC interventions. This does not mean that CHC members will not work hard in the field; CHC members need to act as examples to CHC volunteers. The role of CHC members is to organize and motivate these individuals and community groups.

- **STEP 1: Select three (3) CHC Volunteer Leaders.** The first step in establishing a neighborhood CHC is to select 3 CHC Volunteers Leaders who act as informal

leaders and mobilizers for the group in their neighborhood. These 3 people should:

- Be non-sectarian and non-political.
- Be selected to represent a full cross section of the community without discrimination.
- Have very good reputations at the level of the community.
- Be able to work with a spirit of voluntarism and compassion.
- Be accepted by the people of the locality.
- Reside within the community.

6.2. Organize a network of neighborhood CHC volunteers

- **STEP 2: <u>Organize a network of local volunteers.</u>** Selection of the 3 CHC Volunteer Leaders should be determined before the official launch of each CHC volunteer group. CHC members should visit the 3 CHC Volunteer Champions and develop a list of people from the neighborhood to invite at the launch event for the neighborhood CHC volunteer group. They should aim to invite people from the following social and cultural institutions and organizations:
  - Schools
  - Churches
  - Youth organizations
  - Women's groups
  - Agricultural groups and cooperatives
  - Cultural groups
  - Music groups
  - Football groups
  - Clean-up groups
  - Health groups
  - Local government
  - Medical staff
  - Traditional healers

Membership will not be exclusive. Anyone can join the group. The more CHC volunteers in the neighborhood, the better! The CHC Volunteer Leaders will spread the news about the new neighborhood CHC during the next week. They will invite as many community members as they can to the first launch meeting.

If a community-based health group already exists in the neighborhood, efforts should be made to see if the goals of malaria control could be integrated with this existing group. Instead of creating a separate group, CHC members should see if it would be possible to work with the existing group. If the existing group is very specific to one health issue (such as women's health) then it may be better to create a separate group. In this case, it

is still important to have members of this health group active in the neighborhood CHC.

6.3. Officially launching a neighborhood CHC group

- **<u>STEP 3: Launch the neighborhood CHC group.</u>** The official launch of the neighborhood CHC volunteer group should be organized ahead of time to include activities that raise awareness about the CHC and about malaria. This could include:
    - A parade with music;
    - Announcements at churches; and
    - The involvement of school children and teachers.

At the launch event, CHC members along with MSPP representatives should introduce the CHC approach, discuss important facts about malaria and introduce the various interventions that the CHC plan to implement in the neighborhood.

CHC members should ensure that adequate numbers of health education material are available to distribute to all community members who attend the launch event and that extra copies are available for CHC volunteers to pass out during the parade.

A follow-up plan should be made with the CHC Volunteer Leaders in order to start the first official CHC intervention.

6.4 Very active neighborhood CHC volunteer groups

Most neighborhood CHC volunteer groups will not work independently but only when mobilized by CHC members. If community members want to self-organize and conduct their own anti-malaria interventions outside the plans of the CHC, the CHC should discuss this with the MSPP focal person. Only groups that have showed exceptional capability and dedication should be encouraged to self-form into a more active neighborhood CHC group. This should not be discouraged, but at the same time great care should be taken to supervise and manage these volunteer groups.

If CHC neighborhood volunteer groups want to work without the direct physical involvement of CHC members, a plan should be made with the CHC. In this case, neighborhood CHC groups should conduct intervention activities at a minimum of once every 1 or 2 weeks. Neighborhood CHC groups may also want to create sub-groups, such as a youth group and church group, that can work independently in the community on specific interventions.

In this case, each neighborhood CHC group will need some material and supplies to assist it with CHC interventions. These will include:

- A banner and two flags to raise awareness of meetings and to carry around the

community during interventions.

- One booklet to keep track of community members who participate and to list all formal members of the neighborhood CHC.
- Health education material.

In this case, a plan should be made with each neighborhood CHC on how often they will meet and on the different types of interventions they would like to initiate and carry out. To start the discussion, CHC members should present the list of CHC interventions to the neighborhood CHC and facilitate a discussion with them about the strengths and challenges of each type of intervention.

The interventions of the neighborhood CHC should not be viewed as separate than the interventions of the CHC. It is critical that CHC members are fully engaged with all neighborhood CHC interventions and participate regularly in their interventions.

6.5. Implementing interventions outside areas with CHC groups

Some activities may need to be initiated in areas without active neighborhood CHC groups. This may occur in large cities around important festivals and holidays. It may also occur in response to an active malaria case. If this is the case, CHC members should organize to have CHC volunteers from neighborhood CHC groups involved in malaria control activities in these other areas and a plan should be developed.

6.6. Working with MSPP and other medical staff at the neighborhood level

It is important that CHC members work in collaboration with MSPP and other medical staff in organizing and overseeing each neighborhood CHC group. Each neighborhood group should also directly work in collaboration with local MSPP staff, local private clinics, traditional healers, community health agents, and any community health groups that already exist. Working with these groups will be critical to improving malaria diagnosis and treatment.

6.7. Initiating the rapid response plan with CHCs volunteer groups

As soon as MSPP alerts the CHC about a possible or confirmed malaria case, CHC members should contact the neighborhood CHC group leaders at that location to begin the process of initiating the rapid malaria response plan. If there is currently no neighborhood CHC group in that specific neighborhood, the closet group or groups should be contacted and engaged. In this case, efforts should be made to rapidly create a

neighborhood group by engaging the closest members of the CHC volunteer network.

CHAPTER 7: Supervision and Learning

All successful public health programs keep detailed records and collect various types of information, including feedback from community members. **This is called monitoring.** It gives us an idea of how effective the interventions are in achieving their goals.

This information is very important for learning and adapting to the needs of the community. It can also help motivate people involved in implementing the CHC plans. Motivation and a sense of purpose are very important in achieving our results.

7.1. Prizes and evaluation

CHCs are voluntary groups. They do not receive salaries or per diems for their work. However, MSPP and implementation partners may want to consider (depending on financial resources) providing prizes to the top preforming CHCs. If decided, this should occur every 6 or 12 months to the top 3 CHCs in each Department. These prizes should consist of three levels: gold, silver and bronze.

In this case, the CHCs will be evaluated based on the following criteria by the supervisor team, MSPP and implementing partners:

- Number and quality of interventions
- Community acceptance
- Impact of interventions, including on malaria cases
- Timeliness of reporting and interaction with MSPP and partners.

The prizes will be distributed at meetings with all CHC members in the department. During this time, MSPP and partners will present on the current status of the CHC program and provide training to address specific issues and concerns. MSPP and implementing partners should consider covering the travel costs of CHC members if financial support is available.

7.2. Program supervision and reporting

The CHC program will be supervised by MSPP and TCC staff who will provide support to the CHCs. This will include a local MSPP focal person from your commune as well as a supervisor at the arrondissement level. The supervisors will assist with organizing and planning the CHC and will also collect monthly reports and all Activity Forms mentioned in this manual. The monthly reports will be communicated through the use of cellphones to the supervisor team. **Details are provided in a Monitoring & Evaluation (M&E) manual, a supplementary document that accompanies this implementation manual.**

7.3. Community feedback research activities

Some CHCs will be selected to be part of a community feedback system. This will involve special staff from MSPP and implementing partners visiting the CHC at regular intervals, every 3 months. During these visits, the community feedback team will work with the CHC to initiate discussions and interviews in the community, including with CHC volunteers, different community groups and individual households. This information will be used by program staff to improve the functioning of the CHC program. Some of this data will be shared with the CHCs. The community feedback team will explain more about these activities when they visit the CHC. Again, details are outlined in the M&E manual.

7.4. Malaria data from MSPP

MSPP collects routine information from health clinics that include the number of people tested for malaria, the number of positive cases and the number of treated cases. This information will be routinely shared from the MSPP focal person to each CHC. This data should be presented during the monthly CHC meetings in order to inform CHC members about malaria epidemiology in the Department and in the sub-commune.

7.5. Learning and adapting

After every 6 months, the MSPP focal person and the CHC will review the progress of the CAP and evaluate the successes and challenges that the CHC has encountered in implementing their plan. This should be used as an opportunity to adapt the CAPs.

During this meeting, the MSPP focal person and partners should present some monitoring and evaluation data and provide an update on the CHC program in general. CHC members should be allowed to ask questions about the program and to provide suggestions about how to improve its functioning.

A further chance to improve the CHC program will occur during routine CHC conference meeting (which should occur every 6 or 12 months); each CHC member should attend. This will depend on financial resources being available.

7.6. Adapting your CHC beyond *malaria* to other health problems

Once CHCs have implemented all aspects of this manual, they will have the opportunity to decide if they would like to expand their focus beyond just malaria to include other health issues in their community. Guidance about how to do this will be provided to selected CHCs in Year 2 of the program and once they have discussed this with their MSPP focal person.

Annex

1. Community Health Councils Haiti Program Organogram

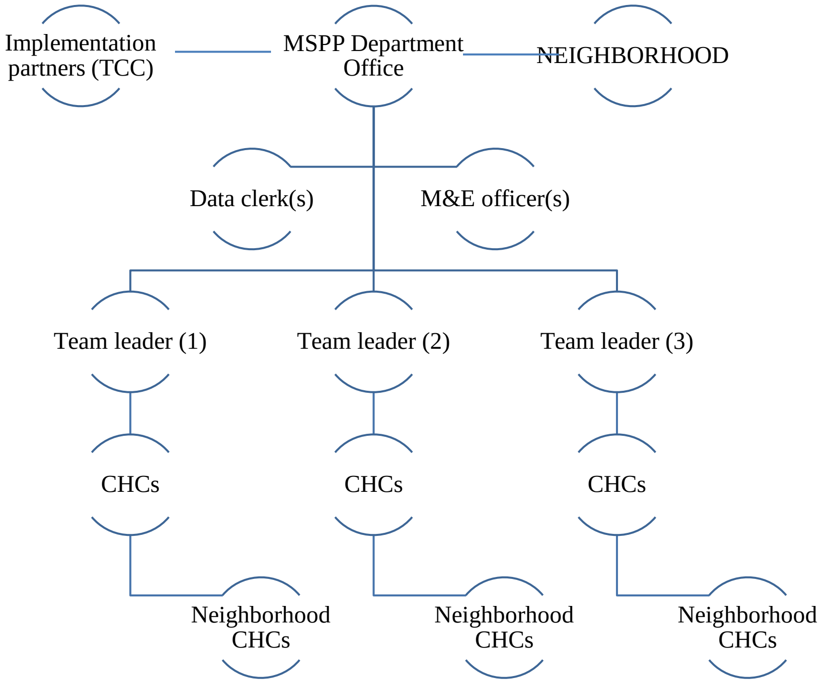

Description: The CHC program is to be organized through the MSPP Departmental Office in each Department with support from PNCM and implementation partners, including The Carter Center.

PNCM and implementation partners (The Carter Center): Responsible for technical support to MSPP including training, monitoring and evaluation, supervisor of staff, and addressing emerging problems.

MSPP Department Coordinator: Responsible for all technical and administrative aspects of the implementation of the program at field level. This will include mobilizing existing staff at the departmental and local level.

MSPP M&E officer: Based at the MSPP Departmental Office and responsible for following all M&E protocols as outlined in the M&E handbook and for ensuring robust M&E data and reporting.

MSPP data clerk: Based at the MSPP Departmental Office and responsible for assistance with all reporting aspects of the program and in day-to-day office assistance to the Coordinator and M&E officer.

Team leaders: Existing MSPP staff based at the commune level and report to the MSPP CHC Coordinator. Responsible for all day-to-day field supervision and support to CHCs, including providing technical support to CHCs and assistance with training.

CHC members: There should be between 9 and 13 CHC members who represent all sectors of society.

Neighborhood CHC volunteer groups: These should be organized in neighborhoods with active malaria cases or recent cases. Each group should have 3 CHC Volunteer Leaders who act as liaisons between the community and the CHCs.

2. Activity Report Checklist

Activity number	Title of Activity	Is Activity complete?	Has Activity Report been submitted?
Activity 1	Developing a vision of your CHC		
Activity 2	Mapping malaria risk factors in your area		
Activity 3	Develop a list of outstanding questions CHC members have regarding malaria		
Activity 4	Discuss strengths and weaknesses of malaria approaches	- Not applicable -	
Activity 5	Identify routine interventions		
Activity 6	Identify where you want to implement your interventions		
Activity 7	Identify who to target		
Activity 8	Identify how often to implement your activities		
Activity 9	Develop a 6-month activity calendar	CHCs should be given calendar booklets for this activity	
Activity 10	CHC Rapid response plan		
Activity 11	Material and supply plan		

**Activity Report 1: Developing a vision of your CHC**<br>**Date:**
Name of the CHC:<br><br><br><br>Slogan of the CHC:<br><br><br><br>Code of conduct of the CHC:

**Activity Report 2**<br>**Date:**	
**Questions**<br><br>Malaria transmission<br><br>Malaria diagnosis<br><br>Malaria treatment<br><br>Malaria prevention<br><br>Malaria elimination	**Answers provided by CHC members (if any):**

**Activity Report 3**<br>**Date:**
Photo of the poster with malaria risk factors

**Activity Report 5**<br>**Date:**	
**Possible interventions**	**Interventions chosen by the CHC**<br>(Write them down here, or mark with an X)
House to house visits	
Community meetings	
Community event	
School program	
Community announcements	
Support people with malaria symptoms to be diagnosed and treated	
Work with and support health clinics and hospitals (public and private)	
Work with and support traditional healers	
Work with and support community health agents	
Promote the use of bed-nets and window screens	
Identify and monitor important mosquito breeding sites	
Engage in environmental clean-up	
Work with NGOs and health clubs	
Work with local government	
Work with MSPP	

**Activity Report 6**<br>**Date:**	
**Name of neighborhood selected**	**Reason for selecting it**

**Activity Report 7**<br>**Date:**		
**Selected intervention**	**Social group you would like to target**	**Reason for targeting this social group**

**Activity Report 8**<br>**Date:**	
**Will the CHC decide on Option 1 or Option 2?**	
**Name of each neighborhood selected for the next 6 months**	**Name of each intervention selected for next 6 months**

Activity 10: CHC Rapid Response Plan
Date:

To implement your CHC Rapid Response Plan, you will need to decide what interventions you would implement to response to a malaria case and create a plan for the:

- Intensive phase of activities over 2 weeks aimed at locating any additional malaria cases.
- Additional phase of activities over 3 months.

Write this down here:

Interventions chosen for intensive phase of the rapid response activities	Interventions chosen for the 3-month phase of rapid response activities

Once this period of intervention is done, you will then incorporate this neighborhood to the target neighborhoods for your routine interventions for a period of one-year.

Activity 11: Material and Supply Plan
Date:

This plan will need to be updated on a regular basis.

		Requested (date)	***Approved? (Y/N)***	***Received (date)***
**1. Educational material**				
	Pamphlets			
	Fliers			
	Posters			
**2. Planning supplies**				
	Books			
	Poster boards			
	Pens			
**3. Implementation material (if available)**	T-shirts			
	Badges			
	School program kits			
	Clean up equipment			
**4. Other resources**				
**5. Special disease control equipment (requested by local MSPP)**				
**6. Community resources and contributions**				
