# Supplementary material for: Evaluating a community engagement model for malaria elimination in Haiti: lessons from the community health council project (2019–2021)
Source: Malar J. 2023 Feb 9;22:47. doi: 10.1186/s12936-023-04471-z (PMC9910254; doi:10.1186/s12936-023-04471-z)
Supplement: Supplementary file 1 — Additional file 1. CHC implementation manual English and French. [file 12936_2023_4471_MOESM1_ESM.zip › New folder/CHC Implementation Manual_French.pdf]

2020

Conseils de Santé Communautaire (CSC) : Manuel d’Exécution pour L’Engagement Communautaire et l’Elimination de la Malaria en Haïti

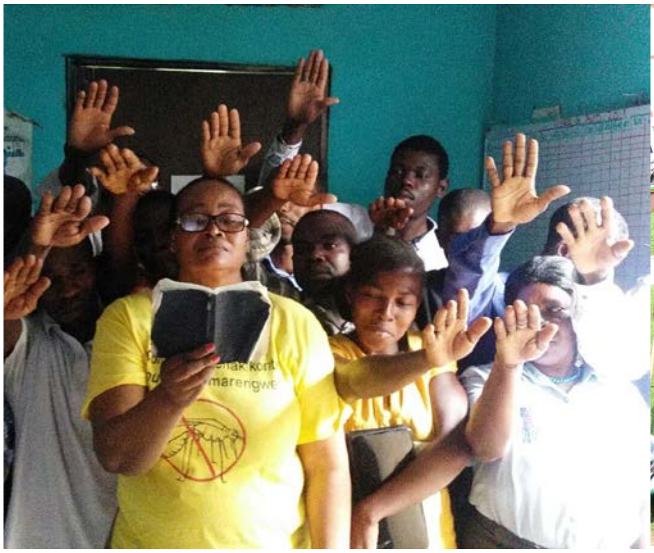

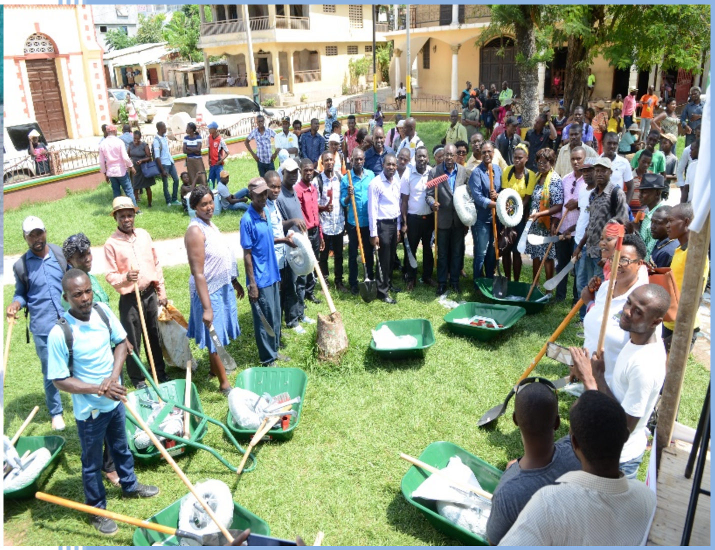

Ceci est un manuel destiné aux membres des Conseils de Santé Communautaire (CSC), le Ministère de la Santé Publique et de la Population (MSPP) et d’autres partenaires de mise en œuvre de la Grande Anse, Haïti, qui explique la procédure étape par étape. Il fournit des directives détaillées sur la façon dont les membres doivent établir et réaliser les activités quotidiennes de leurs conseils de santé communautaire et la façon dont le personnel du MSPP doit les appuyer ce faisant. Ces deux éléments sont d’une importance critique pour renforcer la santé communautaire et éliminer la malaria en Haïti.

Citation suggérée:
Bardosh, K., Jean, L., Desir, L., Yoss, S., Nu, S., Poovey, B., Blount, S., et Noland G. (2020). Community Health Councils (CSC): *An Implementation Manual for Community Engagement and Malaria Elimination in Haiti* (Manuel de Mise en Œuvre for l’Engagement Communautaire et l’Elimination de la Malaria en Haïti). The Carter Center: Atlanta.

Table des Matières

Comment Utiliser ce Manuel 1
CHAPITRE 1 : L'approche du Conseil de Santé Communautaire 2
1.1. Qu'est-ce qu'un Conseil de Santé Communautaire 2
1.2. Pourquoi les CSC sont-ils importants pour l'élimination de la malaria ? 3
1.3. Activités des CSC et portée de leur travail 5
1.4. Le soutien et l'assistance fourni aux CSC 5
1.5. Séquence des étapes pour l' établissement d'un CSC 6
CHAPITRE 2 : Rôles et responsabilités des membres des CSC. 7
2.1. Effectif des CSC. 7
2.2. Composition d' un CSC 8
2.3. Interaction des CSC avec les membres et groupes de la communauté 10
2.4. Interaction des CSC avec le MSPP 10
CHAPITRE 3 : Développer une connaissance situationnelle de la malaria. 12
Activité 1- Développer une vision pour votre CSC 13
Activité 2 : Etablir une liste des questions en suspens que les membres du CSC ont au sujet de la malaria 14
Activité 3 : Cartographier les facteurs de risque de la malaria dans la communauté 16
Activité 4 : Discuter des forces et faiblesses des interventions de lutte contre la malaria 18
CHAPITRE 4 : Elaborer un Plan d'Action Communautaire 20
4.1. Planification de vos interventions routinières 21
4.2. Elaborer un plan de réponse rapide à la malaria 29
4.3. Elaborer un plan pour les matériels et les ressources 33
CHAPITRE 5 : Exécuter le Plan d'Action Communautaire. 37
5.1. Commencer modestement et étendre 37
5.2. Créer une voir régulière de communication avec le MSPP 37
5.3. Faites preuve de créativité! Incorporer l'art, la musique et la culture haïtiennes 37
5.4. Activités dans les écoles 38
5.5. Nettoyage de l'environnement 38
5.6. Suivre les cinq principes pour engager tous les secteurs de la société 38
CHAPITRE 6 : Etablir des groupes CSC de quartier 40
6.1. Choisir des volontaires pour diriger les CSC dans chaque quartier 40
6.2. Organiser un réseau de volontaires de quartier pour les CSC 41
6.3. Lancement officiel du groupe CSC de quartier 42
6.4. Groupes de volontaires CSC de quartier très actifs 42
6.5. Réaliser des activités dans des zones où il n'y a pas de groupe CSC 43
6.6. Travailler avec le MSPP et autres professionnels de la médecine au niveau des quartiers 44
6.7. Initier le plan de réponse rapide avec le groupe de volontaires du CSC 44
CHAPTER 7: Supervision et Apprentissage 45
7.1 . Prix et évaluation 45
7.2. Supervision du programme et rapportage 45
7.3. Activités de recherche sur le feedback des communautés 46
7.4. Données du MSPP sur la malaria 46
7.5. Apprentissage et adaptation 46
7.6. Adapter votre CSC à d'autres problèmes de santé au-delà de la malaria 47
Annexe 48

Comment Utiliser ce Manuel

Ce manuel est un guide qui explique étape par étape la façon d'établir et d'opérer des Conseils de Santé Communautaire (CSC) en Haïti.

Il inclut des directives pour les membres des CSC, le personnel du MSPP et les autres partenaires d'exécution.

Ce manuel doit être distribué aux membres des CSC de même qu'au personnel du MSPP impliqué dans le soutien du programme.

Le manuel comprend sept (7) chapitres.

Chapitre 1 Introduit le Conseil de Santé Communautaire (CSC).

Chapitre 2 Définit les rôles et responsabilités des membres des CSC.

Chapitre 3 Décrit quatre (4) activités que les CSC doivent réaliser pour renforcer leur conscience situationnelle par rapport à la malaria.

Chapitre 4 Donne des instructions étape par étape pour élaborer les plans d'action des CSC, incluant les interventions routinières, les Plans de Réponse Rapide et un Plan pour les Matériels et des Ressources.

Chapitre 5 Discute d'un nombre de considérations essentielles pour la mise en œuvre des plans d'actions des CSC.

Chapitre 6 Donne des instructions étape par étape pour établir des groupes CSC de quartier pour la mise en œuvre du plan d'action des CSC.

Chapitre 7 Souligne les composantes majeures de supervision et d'apprentissage nécessaires à assurer la mise en œuvre et le fonctionnement réussi des CSC.

L'Annexe Fournit l'organigramme du programme et une série de modèles de rapport à remplir par les membres des CSC au fur et à mesure qu'ils examinent le manuel et finalisent les onze (11) activités prévues. Une liste de vérification des activités est aussi incluse.

CHAPITRE 1 : L'approche du Conseil de Santé Communautaire

1.1. Qu'est-ce qu'un Conseil de Santé Communautaire

Un Conseil de Santé Communautaire (CSC) est une Organisation Bénévole opérée par des résidents locaux en vue d'agir au nom de leur zone en faisant la promotion de la santé et des changements sociaux positifs. Les membres des CSC se focalisent sur la menée de campagnes d'éducation et de changement de comportement, aidant aux activités de surveillance des maladies communautaires, et renforçant les liens entre la communauté et les professionnels de la médecine, sans oublier la mobilisation des résidents de la localité pour la prévention des maladies dans leur quartier et dans la communauté en général. Ces types de Conseils Communautaires étaient communs en Haïti, en particulier dans les zones rurales, dans les années 70 et 80. Le modèle de CSC (décrit dans ce manuel) vise à relancer l'implication de participation communautaire dans les questions de santé locales qui existaient par le passé. Les CSC sont des organisations formées de personnes locales intéressées à leur communauté et qui veulent en faire des endroits meilleurs où vivre. Nous pensons que ce modèle de capacitation communautaire a un rôle à jouer dans le renforcement des soins de santé primaires en Haïti aujourd'hui, et dans lutte contre la malaria (et autres maladies infectieuses) à travers le pays.

Le but principal du programme CSC est d'autonomiser les personnes, les familles et les communautés d'Haïti en vue de renforcer les mesures locales de santé et de changement social de sorte à aider directement à l'élimination de la malaria.

Les quatre (4) objectifs spécifiques du programme CSC sont de :

1. Renforcer le bien-être et la santé des communautés ;

2. Renforcer la capacité et la compétence des communautés pour planifier et s'engager dans les problèmes de santé ;

3. Regrouper les résidents des localités en vue de planifier et de les engager dans l'élimination de la malaria ;

4. Fournir une plateforme communautaire pour le contrôle des maladies transmises par vecteur et les programmes d'élimination du Ministère de la Santé Publique et de la Population (MSPP) en Haïti.

L'établissement initial des CSC met un accent particulier sur les activités de prévention et de contrôle de la malaria. Ceci n'est pas un point de concentration exclusif. Une fois que les CSC

sont fonctionnels et ont réalisé des activités pendant seize (16) à douze (12) mois, nous les encourageons à définir d'autres priorités sanitaires sur lesquelles ils veulent travailler dans leur communauté, et à utiliser les outils, les directives, et les protocoles définis dans ce manuel pour augmenter leurs efforts en vue d'aborder ces autres problèmes importants. Des directives sur la façon de ce faire sont soulignées dans le dernier chapitre de ce manuel.
L'approche CSC a été établie par le Carter Center qui dirige l'engagement communautaire pour l'Alliance[1] Zéro Malaria en vue d'appuyer les efforts d'élimination de la malaria en Haïti. Le modèle conceptuel des CSC a été élaboré avec l'assistance des collègues de l'Université d'Amsterdam (voir : Pool, de Vries et Bardosh (2018) *Malaria Zero : Community Engagement Plan* – Zéro Malaria : Plan d'Engagement Communautaire) et raffiné par des recherches formatives (voir : Druetz et al. (2018). « Là où les médecins ne peuvent pas arriver, le soleil lui, peut » : surmonter les barrières éventuelles aux interventions d'élimination de la malaria en Haïti). *Malaria Journal*, 17 (1, 1-11). Ce manuel tient compte de ce qui a été appris à partir de ce travail de même qu'une phase pilote initiale des CSC dans le département de la Grand Anse.

1.2. Pourquoi les CSC sont-ils importants pour l'élimination de la malaria ?

La malaria est causée par un parasite et propagée par les moustiques. Cette maladie est très répandue dans le département de la Grand Anse. C'est aussi une maladie que le MSPP et d'autres partenaires internationaux ont ciblé pour élimination. Haïti et la République Dominicaine sont les seuls pays de la Caraïbes où la transmission de la malaria existe encore. Pour atteindre l'objectif de l'élimination de la malaria et protéger les communautés et les générations futures des menaces de la malaria en Haïti, le MSPP et les partenaires d'exécution ne peuvent pas travailler en dehors des communautés locales et des organisations communautaires. La communauté dans son intégralité doit être engagée dans la lutte.

Le programme CSC donne une ouverture aux membres et aux leaders communautaires en vue d'organiser et d'autonomiser les citoyens locaux à s'engager dans la lutte contre la malaria et à travailler en collaboration avec le MSPP.

Selon le Plan Stratégique National (2016-2022), cette lutte contre la malaria comporte six (6) piliers dont plusieurs nécessitent l'implication directe des CSC :

1. **Renforcer la prise en charge des cas de malaria :** les signes et symptômes de la malaria sont similaires à plusieurs autres fièvres et maladies et beaucoup de gens ne

[1] Les partenaires incluent le Ministère de la Santé Publique et de la Population, le Ministère de la Santé Publique et de l'Assistance Sociale de la République Dominicaine, les Centres de Contrôle et de Prévention des Maladies des Etats-Unis, la Fondation CDC, l'Organisation Panaméricaine de la Santé, le Carter Center, le Clinton Health Access Initiative, l'Ecole d'Hygiène et de Médecine Tropicale de Londres, et l'Ecole de Santé Publique et de Médecine Tropicale de l'Université de Tulane.

cherchent pas à trouver des soins dans les centres de santé jusqu'à ce qu'il soit trop tard. Ceci mène à de plus grandes possibilités de propagation de la maladie dans la communauté et de mortalité causée par la maladie. Les CSC peuvent aussi aider à appuyer les installations sanitaires du MSPP, les cliniques privées et les agences de santé communautaire (particulièrement dans les zones rurales) dans leurs efforts de diagnostic et de traitement de la malaria.

2. **Elaborer un système de distribution et de gestion pour les diagnostics et les traitements.** Ceci est important pour assurer que les matériels soient disponibles à travers tout le pays.

3. **Renforcer la surveillance épidémiologique.** Il est très important de localiser les cas de malaria pour connaître les endroits où se propage la maladie et savoir si les activités anti-malariales marchent bien.

4. **Renforcer les interventions de contrôle des vecteurs.** Une partie importante du contrôle de la malaria est le contrôle des moustiques et l'assainissement environnemental qui inclut la réduction des sources d'eau stagnante et de haute densité de moustiques.

5. **Renforcer le Système de Santé National.** La lutte contre la malaria exige le développement et le renforcement de la capacité d'agir des personnes, des familles, des regroupements communautaires, du personnel sanitaire et des gouvernements locaux. Les CSC sont responsables de mobiliser les citoyens pour identifier les barrières et les défis et aider à trouver des solutions locales qui renforcent le système de santé dans la lutte contre la malaria. Parfois, le MSPP organise des interventions spécifiques par rapport à la malaria, ceci peut inclure une distribution massive de moustiquaires, des tests anti-malaria, des médicaments contre la malaria, l'aspersion des maisons avec des produits chimiques et l'utilisation de produits chimiques dans les sources d'eau stagnante. Ceci se fera seulement dans les zones de transmission élevée de malaria et dépendra des financements externes. Les CSC doivent aider à leur mise en œuvre.

6. **Renforcer le IEC (Information, Education et Communication) dans la communauté.** Il est absolument essentiel de conscientiser les gens sur les signes et symptômes, le traitement et les moyens de prévenir la malaria en vue de promouvoir les comportements des personnes et des ménages et les pratiques de lutte contre la malaria et d'autres maladies.

Il est fondamental que les membres des CSC aient une bonne compréhension de la malaria lorsqu'ils planifient et effectuent les activités associées aux six (6) piliers de contrôle de la

malaria. Tous les membres des CSC doivent se familiariser avec le **matériel d'éducation sur la malaria** fourni par le MSPP. Ce matériel sera fourni séparément de ce manuel.

1.3. Activités des CSC et portée de leur travail

Le Conseil de Santé Communautaire doit :

1. Travailler avec le MSPP pour s'approprier les activités locales d'élimination de la malaria ;
2. Représenter les intérêts (la voix) du Ministère de la Santé et des partenaires d'exécution lors des réunions communautaires fixées ;
3. Organiser et tenir une réunion avec les membres des CSC une (1) fois par mois ;
4. Répondre rapidement à n'importe quel cas de malaria dans la communauté ;
5. Organiser et réaliser au moins quatre (4) interventions communautaires par mois, incluant des activités de sensibilisation et de prévention ;
6. Sensibiliser la communauté sur la nécessité de se rendre à une institution de santé au début d'une fièvre ;
7. Aider à élaborer des stratégies et des messages pertinents à l'élimination de la malaria dans chaque communauté ;
8. Participer activement comme messagers et organisateurs des activités en ligne avec le programme d'élimination de la malaria dans la communauté ;
9. Promouvoir le changement de comportement ;
10. Être disponible pour aider au niveau de toutes les interventions organisées par le MSPP contre la malaria ;
11. Rapporter les activités du groupe sur une base mensuelle au niveau sanitaire des communes.

Une fois qu'un CSC devient très actif, il peut aller jusqu'à se colleter aux problèmes au-delà de la malaria. Chaque CSC doit prendre cette décision en collaboration avec le MSPP et les partenaires.

1.4. Le soutien et l'assistance fourni aux CSC

Les CSC sont des organisations bénévoles. Le MSPP et les partenaires d'exécution ne donnent ni salaire ni *per diem* aux membres ou aux volontaires des CSC. Les CSC doivent être des groupes communautaires autonomes qui mobilisent les citoyens de la localité en vue d'améliorer la santé de la population à partir d'un esprit de solidarité et de compassion.

Le MSPP et les partenaires d'exécution apporteront leur soutien dans l'établissement des CSC et dans certains aspects de leur fonctionnement et de leur supervision. Le MSPP et les partenaires d'exécution peuvent fournir des matériels de soutien comme des t-shirts, des badges, des

matériels éducatifs, des outils de planification et des produits de nettoyage. Il y aura peut-être une aide pour voyager aux réunions officielles avec le MSPP, mais par pour le fonctionnement quotidien du CSC. Ceci peut changer dans le temps et devra être discuté et convenu à l'avance avec le MSPP et les partenaires d'exécution.

1.5. Séquence des étapes pour l' établissement d'un CSC

Les étapes comprises dans l'établissement et la gestion d'un CSC incluent:

- Décider de l'effectif et organiser les rôles et les responsabilités ;
- Maintenir la participation des membres ;
- Développer une vision partagée pour votre CSC ;
- Faire une évaluation des besoins sanitaires ;
- Créer une main-d'œuvre volontaire ;
- Mettre en œuvre des plans d'action communautaire ;
- Apprendre et s'adapter.

Ces points sont décrits dans les chapitres restants de ce manuel.

CHAPITRE 2 : Rôles et responsabilités des membres des CSC.

2.1. Effectif des CSC

La première étape dans le choix des membres d'un CSC est :

1) Le Bureau Départemental du MSPP en accord avec les autorités sanitaires locales doit organiser une réunion avec tous les secteurs des sections rurales ou de la ville, selon l'endroit où le CSC doit être établi. Les autorités locales (maires, CASCEC, ASEC, Département de la Justice, Gouvernement local), les représentants des organisations communautaires, les leaders naturels (pasteurs, prêtre, ASCP (Agent de Santé Communautaire Polyvalent), les guérisseurs traditionnels) doivent être invités à la réunion. Il faut la participation d'à peu près quarante (40) à cinquante (50) personnes de zones géographiques différentes des sections rurales.

2) Le MSPP et les partenaires qui animent la réunion doivent présenter les objectifs principaux des CSC et l'importance de choisir une grande gamme de parties prenantes pour être des membres.

3) Ils doivent alors faire une brève présentation sur la malaria et sur la façon dont l'engagement communautaire peut améliorer les conditions de vie dans leur communauté.

4) Il faut prendre le soin de sous-diviser les participants en différents groupes sociaux en tenant compte d'un équilibre entre les gens, de statut socio-économique et d'expérience professionnelle.

5) Le processus de sélection doit se faire de manière ouverte et transparente. Le but est de choisir des gens qui sont intéressés et engagés. Ceci doit tenir compte de l'inclusion de personnes de différentes zones géographiques et d'une variété de groupes sociaux qui doivent être représentés au sein du Conseil sans préjugés.

6) Il faut alors planifier et offrir des sessions de formation pour augmenter leurs connaissances sur la malaria et d'autres maladies et sur la façon d'assurer que tous ceux qui vivent dans la communauté soient engagés dans le travail d'amélioration de la santé et des moyens de survie.

7) Puis, des produits de nettoyage de l'environnement doivent être délivrés afin que le CSC puisse promouvoir le changement de comportement (s'il y a des ressources pour ce faire).

Il faut avoir entre neuf (9) à treize (13) membres d'un CSC qui représentent tous les secteurs de la société : paysans, femmes, leaders religieux, jeunes, organisations locales, enseignants, personnel sanitaire, secteur des affaires, etc.

Chaque Centre de Santé Communautaire (CSC) doit :

- Être non-sectaire et apolitique ;
- Être choisi de sorte que les CSC représentent un échantillon complet de la communauté sans discrimination ;
- Avoir une très bonne réputation au niveau de la communauté ;
- Pouvoir travailler dans un esprit de bénévolat et de compassion ;
- Être accepté par les gens de la communauté ;
- Résider dans la communauté.

Il est très important que les CSC ne soient ni affiliés ni associés à un quelconque parti politique afin qu'ils ne soient pas utilisés à des fins politiques et qu'ils soient vus comme des organisations neutres qui travaillent au bénéfice de tous les membres de la communauté.

2.2. Composition d' un CSC

Le Conseil Communautaire est composé de représentants qui viennent de toute la section communale. Au sein des membres du Conseil Communautaire sera choisi un (1) Conseil d'Administration de quatre (4) personnes pour gérer et coordonner les actions du Conseil pendant deux (2) années. Après ces deux (2) années, chaque CSC choisira un Conseil d'Administration différent. Ceci implique un processus de nomination et de vote par chacun des membres du CSC. Le Conseil d'Administration est composé des membres suivants :

- Un Coordonnateur ou un Président ;
- Un Secrétaire ;
- Deux (2) délégués ou conseillers.

Rôle du Coordonnateur ou Président

Le Coordonnateur ou Président doit :

- Présider les réunions du Comité et représenter le Comité dans toutes ses activités ;
- Convoquer des réunions routinières et extraordinaires ;
- Préparer les ordres du jour ;
- Préparer et soumettre les rapports annuels des activités après approbation des membres du Comité lors les Assemblées Générales ;
- Soumettre régulièrement le rapport des activités du Comité au MSPP ;

- Assurer le bon fonctionnement du Comité ;
- Assurer que les plans d'actions soient réalisés de manière réussie ;
- Assurer le lien entre le Comité et les autres secteurs de la communauté ;
- Assister à toutes les réunions organisées par le MSPP.

Rôle du Secrétaire

Le Secrétaire doit :

- Aider le Coordonnateur à rédiger l'ordre du jour des réunions ;
- Ecrire et tenir les correspondances du Comité ;
- Être responsable d'enregistrer les procès-verbaux de chaque réunion ;
- Maintenir la liste et les dossiers des membres et des volontaires communautaires du CSC ;
- Rédiger le rapport annuel en collaboration avec le Coordonnateur ;
- Signer tous les documents officiels du Comité avec le Coordonnateur.

Rôle des deux Conseillers délégués

Les délégués doivent :

- Encourager les autres membres du Comité à participer aux activités de mobilisation ;
- Assurer le bon fonctionnement du Comité lors des réunions et des activités de sensibilisation communautaire ;
- Promouvoir la mission du Comité au sein de la communauté ;
- Faciliter la résolution de conflits ;
- Encourager les autres membres à poursuivre les objectifs du Comité.

Responsabilités de tous les membres

En dehors des rôles et des responsabilités énumérés ci-dessus, il est anticipé que tous les membres doivent :

- Participer activement à toutes les activités convenues par le CSC ;
- Promouvoir la mission du Comité dans la communauté ;
- Sensibiliser la communauté sur le rôle du CSC ;
- Assister aux réunions du CSC ;
- Participer aux réunions des sous-comités et aux activités selon ce qui a été convenu et décidé.

2.3. Interaction des CSC avec les membres et groupes de la communauté

Les CSC fonctionneront comme agents de mobilisation dans leur communauté et travailleront au niveau des sections rurales. Ils devront travailler en collaboration avec une grande diversité de volontaires communautaires et de groupes afin de mettre en œuvre des interventions contre la malaria et autres problèmes de santé. Il leur faudra des collaborateurs et le soutien d'autres personnes !

Pour cette raison, des directives sont fournies dans ce manuel sur la façon dont les CSC doivent aborder l'établissement d'un réseau de volontaires CSC incluant des directives sur la façon et le moment où les CSC doivent établir des *CSC de quartier* pour exécuter les interventions au niveau local.

2.4. Interaction des CSC avec le MSPP

Les Conseils de Santé Communautaire garderont un contact régulier avec le MSPP et d'autres partenaires d'exécution. Il est attendu que les CSC :

- Transmettent des rapports mensuels sur leurs activités au MSPP ;
- Respectent les délais pour les autres activités qui ont été décidées et convenues ;
- Participent au monitoring des activités organisées par le MSPP ;
- Communiquent rapidement n'importe quel problème ou changement dans la gestion du CSC à l'équipe du MSPP par téléphone.

Le MSPP avec le soutien des autres partenaires d'exécution s'engage à :

- Assister le CSC en lui fournissant un soutien technique et des directives ;
- Fournir une formation en compétences à l'appui du fonctionnement des CSC ;
- Fournir des matériels pédagogiques pour la sensibilisation et la mobilisation communautaires ;
- Fournir des matériels de nettoyage pour les interventions d'assainissement de l'environnement (lorsque possible) ;
- Donner de petites incitations (lorsque possible) incluant un prix annuel pour les CSC ;
- Monitorer et superviser les activités des CSC ;
- Ecouter et répondre aux préoccupations et aux défis.

Les CSC, le MSPP et autres partenaires d'exécution travailleront de manière <u>collaborative et participative</u> et doivent être ouverts aux idées nouvelles et prêts à s'adapter pour relever les défis et trouver de nouvelles solutions pour le contrôle et l'élimination de la malaria et autres problèmes de santé publique dans la communauté.

CHAPITRE 3 : Développer une connaissance situationnelle de la malaria.

Dans ce chapitre, nous décrivons quatre (4) exercices que les CSC doivent faire le **premier mois** de l'établissement de leur CSC. **Ces activités doivent se faire durant la même réunion de planification du CSC.** Cette réunion du CSC sera très longue, mais il est important que les activités 1, 2, 3 et 4 se fassent ensemble. Faire les arrangements pour cette réunion à l'avance et demander aux membres du CSC de se familiariser avec ceux-ci bien avant.

Ce travail aidera les membres du CSC à mieux définir leurs valeurs en tant que groupe et fera en sorte qu'ils aient une compréhension unique de la malaria. Ceci sera important au fur et à mesure qu'ils élaborent des plans spécifiques pour les interventions dans leur communauté.

Il est important que le MSPP et le Conseil d'Administration lisent attentivement les instructions pour chacune des activités et élaborent un plan pour réaliser ces activités avec les membres du CSC lors de leurs réunions régulières.

Les étapes sont les suivantes :

Activité 1 : Trouver une vision pour votre CSC

Activité 2 : Clarifier les questions des membres des CSC sur la malaria

Activité 3 : Cartographier les facteurs de risque de malaria au niveau communautaire

Activité 4 : Explorer les forces et les faiblesses des différentes activités anti-malariales

Activité 1- Développer une vision pour votre CSC

Une fois votre CSC établi, les membres doivent discuter des objectifs et de la vision qu'ils ont pour le CSC. Pour ce faire, le Conseil d'Administration doit faciliter une discussion ouverte entre les membres du CSC. Ceci doit suivre étape par étape les directives décrites ici dans **l'Activité 1**. Dans cette activité, le CSC trouvera un nom et un slogan pour le CSC et conviendra d'un code de conduite.

Activité 1 : Trouver une vision pour votre CSC

Objectif : Arriver à un consensus et un esprit d'équipe au sein du CSC et déterminer le lieu, la date et l'heure des réunions futures.

Matériels nécessaires : Papier et plumes.

Temps requis : 30 minutes

Description de l'activité : Le groupe fera l'activité 1 lors d'une de leurs réunions mensuelles routinières. Le CSC doit :

1. Répartir en sous-groupes de deux (2) ou trois (3) personnes chaque ;
2. Chaque groupe doit penser à un nom et à un slogan pour le CSC ;
3. Au bout de quinze (15) minutes, le groupe doit se reformer et chaque sous-groupe doit partager ce qu'il a trouvé. A ce moment-là, tout le groupe doit convenir d'un nom et d'un bref slogan pour le CSC ;
4. Ensuite, le groupe doit discuter ensemble de leurs attentes pour le groupe. Ils doivent commencer par les deux (2) questions qui suivent : 1) que veulent-ils réaliser au cours de l'année prochaine ? et 2) combien de temps chaque membre peut-il dévouer aux activités du CSC chaque mois ?
5. Un (1) des membres du Conseil d'Administration doit alors mener une discussion sur les rôles et responsabilités des membres du CSC.
6. Le CSC doit convenir du jour, de l'heure et du lieu de rencontre pour les réunions régulières du CSC.
7. Durant cette discussion, ils doivent lire les rôles et responsabilités de chaque membre comme énuméré au Chapitre II.
8. Ensuite, le CSC doit élaborer un Code de Conduite qui exprime clairement les valeurs du CSC et son esprit de travail.

Résultat : Une fois cette activité terminée, le CSC doit avoir convenu d' : 1) un nom ; 2) un slogan ; et 3) un Code de Conduite. Ceci doit être enregistré par le Secrétaire du CSC et une (1) copie du nom, du slogan et du Code de Conduite convenu doit être communiquée au point focal du MSPP selon le format reflété au **Rapport d'Activité 1**.

Activité 2 : Etablir une liste des questions en suspens que les membres du CSC ont au sujet de la malaria

La malaria est une maladie compliquée et les membres de la communauté auront beaucoup de questions concernant les aspects spécifiques de la maladie lors des interventions du CSC. Il est très important que tous les membres du CSC aient une excellente compréhension de la transmission, du diagnostic, du traitement et de la prévention de la malaria de même que des stratégies du MSPP pour éliminer cette maladie.

Pour cette raison, les membres du CSC doivent avoir une discussion ouverte entre eux sur les questions spécifiques qu'ils peuvent avoir sur la malaria – les choses qui ne sont pas claires pour eux ou les domaines où ils aimeraient avoir des informations plus scientifiques. Pour ce faire, chaque CSC doit suivre les instructions données dans **l'Activité 2.**

Il est important que le facilitateur rappelle aux membres du CSC qu'aucune question n'est une mauvaise question. Ils ne doivent pas se sentir gênés de poser n'importe quelle question. C'est le moment de clarifier n'importe quel malentendu ou des points sur lesquels les membres manquent de connaissances sur la malaria.

Activité 2 : Etablir une liste des questions en suspens que les membres du CSC ont au sujet de la malaria

Objectif : Etablir une liste de questions spécifiques que les membres du CSC ont concernant la malaria et pour lesquelles ils veulent avoir une réponse.

Matériels pessaires : Papier et plume, tableau noir ou feuilles volantes.

Temps requis : 45 minutes

Description de l'activité : Le groupe fera l'activité 2 lors d'une de leur réunion mensuelle routinière. L'activité sera menée dans l'ordre qui suit :

1. On demandera aux membres du groupe CSC: « quelles questions chacun d'entre vous a concernant la transmission, le diagnostic, le traitement, la prévention et l'élimination de la malaria sur lesquelles vous aimeriez avoir plus de détails ? »
2. Le facilitateur permettra à chaque membre du CSC individuellement, de poser n'importe quelle question qu'il/elle aurait sur un morceau de papier. Il doit laisser cinq (5) à dix (10) minutes pour leur permettre de le faire.
3. Une fois qu'ils auront tous fini d'écrire leurs questions, le facilitateur demandera au groupe de lire leurs questions.
4. Le facilitateur écrira toutes les questions posées par les membres du CSC sur un tableau noir ou une feuille volante. Toutes les questions doivent être écrites sans interruption et les membres ne doivent pas répondre aux questions à ce moment-là. Il est très important que toutes les questions soient écrites avant que le groupe n'essaie de répondre à certaines de ces questions.
5. Une fois toutes les questions écrites, le facilitateur doit lire la liste des questions dans l'ordre et demander si un (1) des membres peut y répondre.
6. Ils doivent écrire la réponse à chaque question et souligner n'importe quelle question à laquelle les membres du CSC ne peuvent pas répondre ou les points sur lesquels ils trouvent que la réponse est incomplète.
7. Avant de terminer, le facilitateur doit demander aux groupes s'ils ont d'autres questions concernant la malaria sur lesquelles ils aimeraient avoir plus d'informations. Ceci doit être ajouté à la liste.

Résultat : Une fois cette activité terminée, le CSC doit en faire le rapport au MSPP avec une liste des questions et réponses à ces questions suivant le format du **Rapport d'Activité 2**. Ceci sera utilisé pour aider MSPP et ses partenaires à concevoir des matériels pédagogiques pour le CSC.

Activité 3 : Cartographier les facteurs de risque de la malaria dans la communauté

Pour contrôler la malaria, il faut pouvoir diagnostiquer et traiter les personnes qui souffrent de la maladie tout en réduisant le contact entre les personnes et les moustiques.

Il est important que les **CSC comprennent le niveau de capacité de leur système de santé local dans la lutte contre la malaria et les principaux facteurs environnementaux et socio-économiques qui aident à propager la maladie.** Ceci implique la cartographie des points chauds où il y a des moustiques et des visites aux centres de santé locaux pour chercher à connaître leur capacité de diagnostiquer et de traiter les cas de malaria.

Cet exercice commencera à établir une relation entre les CSC et le personnel des centres de santé locaux tout en générant des informations importantes sur la capacité du système sanitaire pour contrôler la malaria et les foyers de moustiques dans le territoire du CSC. Ces informations seront utilisées pour renforcer et améliorer le contrôle de la malaria par les CSC.

Pour y arriver, les membres des CSC doivent suivre les instructions reflétées dans **l'Activité 3.**

Activité 3 : Cartographier les facteurs de risque de malaria dans votre zone

Objectif : Comprendre les obstacles au contrôle de la malaria au niveau du système de santé local et cartographier la géographie des gîtes de moustiques dans la communauté.

Matériels nécessaires : Papier et plume, tableau noir ou feuilles volantes. Liste des cas de malaria et des centres de santé dans le territoire du CSC fournie par le MSPP.

Temps requis : 60 à 80 minutes.

Description de l'activité : Cette activité comportera deux (2) étapes (A + B).
La première étape (A) consistera à dessiner une carte de leur territoire sur la fiche par les membres des CSC. Cette carte n'a pas besoin d'être parfaite mais plutôt rudimentaire, elle doit se faire avec soin, vu qu'elle sera utilisée à l'avenir. Sur la fiche, chaque CSC doit :

1. Commencer par dessiner les bornes des sections rurales ;
2. En deuxième lieu, dessiner les caractéristiques naturelles importantes comme les rivières, les montagnes et les forêts ;
3. En troisième lieu, dessiner les routes majeures ;
4. En quatrième lieu, noter où se trouvent tous les centres de santé, hôpitaux du MSPP et les cliniques privées.

La deuxième étape (B) consistera à ajouter des détails spécifiques à la malaria. Ceci implique :

1. A partir de la liste des cas de malaria fournie par le MSPP, mettre une étoile sur la localisation approximative de chaque cas de malaria qui s'est produit au cours des dix (10) dernières années dans la section rurale. Dans certains cas, ces informations permettront de marquer la localisation spécifique du ménage alors que dans d'autres, seulement le nom du centre de santé qui a diagnostiqué le cas sera disponible ;
2. Une fois que vous aurez marqué tous les cas, vous allez maintenant marquer toutes les grandes sources d'eau stagnante ou zones marécageuses que vous pensez être des gîtes majeurs de moustiques. Marquez ces endroits sur votre carte.
3. Une fois que vous aurez terminé, discuter des facteurs de risque de malaria dans votre zone.

Résultat : Une fois cette activité terminée, il faut garder une copie de votre carte pour utilisation future. Vous devez prendre une photo de la carte et la partager avec votre superviseur du MSPP. A l'avenir, apporter la carte aux réunions de CSC et l'utiliser dans la planification de vos activités. L'affiche elle-même est considérée comme votre **Rapport d'Activité 3**.

Activité 4 : Discuter des forces et faiblesses des interventions de lutte contre la malaria

Avant d'élaborer vos plans d'activité, il est très important de vous familiariser avec les divers types d'intervention que chaque CSC peut mettre en œuvre dans sa communauté. Pour cette raison, il est essentiel que chaque membre d'un CSC lise attentivement la liste d'interventions fournie dans la présente et que le CSC discute de cette liste ensemble en groupe.

Pour ce faire, chaque CSC doit passer une (1) à deux (2) heures à lire et à discuter des options. **L'Activité 4** n'aura aucun résultat spécifique mais constituera la base de votre Plan d'Action Communautaire décrit dans le prochain chapitre. Il n'est pas nécessaire d'écrire quoi que ce soit, cependant, il est important d'avoir une discussion sérieuse sur les activités qui, de l'avis des membres du CSC sont les plus importantes et faisables à réaliser dans la communauté.

1. **Propager la sensibilisation et améliorer les connaissances sur la malaria**		
**Type d'intervention**	**Forces**	**Défis**
1.1 Visites domiciliaires		
1.2 Réunions communautaires		
1.3 Evènements communautaires		
1.4 Programmes scolaires		
1.5 Annonces communautaires		

2. **Améliorer la surveillance et le traitement**		
2.1 Assister les personnes qui présentent des symptômes de malaria à diagnostiquer et à traiter		
2.2 Travailler avec et soutenir les centres de santé et les hôpitaux (publics et privés)		
2.3 Travailler avec et soutenir les guérisseurs traditionnels		
2.4 Travailler avec et soutenir les agents de santé communautaire		

3. **Prévention des moustiques**		
3.1 Promouvoir l'utilisation de moustiquaires ou de fenêtres en tulle		
3.2 Identifier et monitorer les gîtes majeurs de moustiques		
3.3 S'engager dans le nettoyage de l'environnement		

4. **Etablir une solidarité communautaire**		
3.4 Travailler avec les ONG et les clubs de santé		
3.5 Travailler avec les gouvernements locaux		
3.6 Travailler avec le MSPP		

CHAPITRE 4 : Elaborer un Plan d'Action Communautaire

Dans ce chapitre, nous donnons des directives étape par étape sur la façon dont les CSC doivent organiser les interventions sanitaires au niveau communautaire. Une planification soigneuse avec les membres des CSC et les volontaires communautaires sera nécessaire pour assurer la réussite de ces interventions de manière acceptable aux citoyens locaux. Pour y arriver, chaque CSC doit élaborer et respecter un **Plan d'Action Communautaire du CSC (PAC).** Ce chapitre explique comment le faire.

Le Plan d'Action Communautaire (PAC) comportera trois (3) parties, ci-après détaillées :

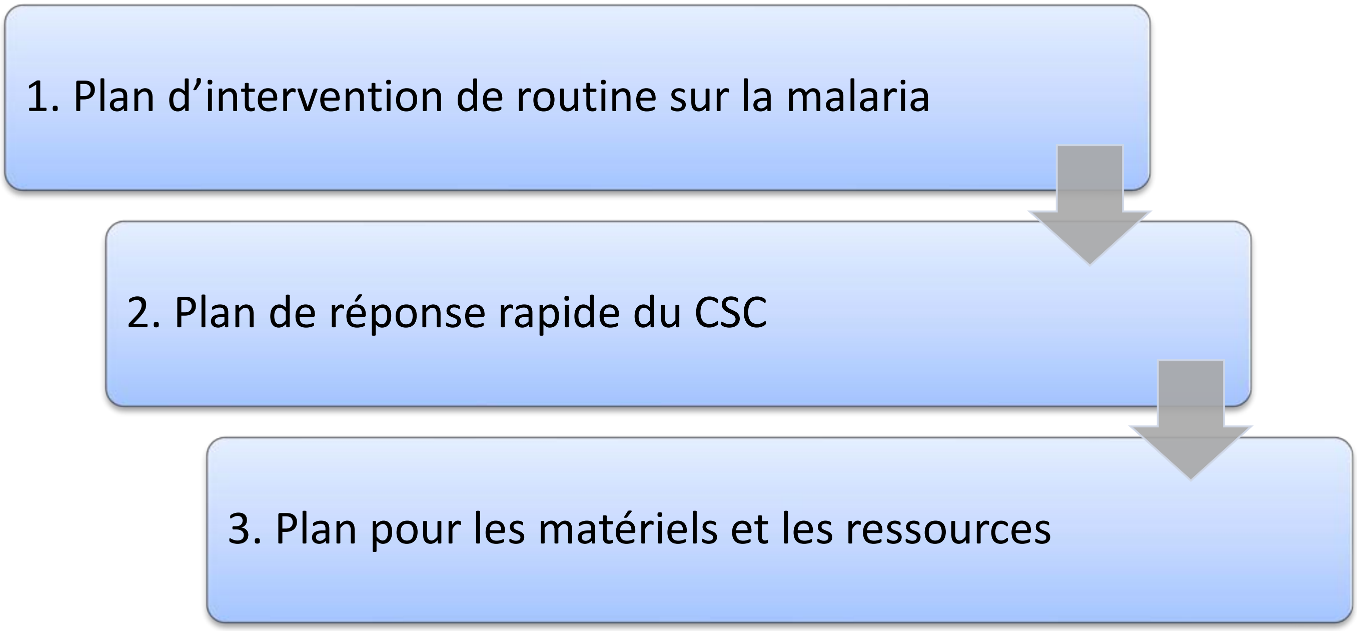

Ces parties sont expliquées dessous en plus de détails.

Chaque CSC doit faire preuve de créativité dans la façon d'élaborer son Plan d'Action Communautaire. Il doit l'adapter aux besoins de la communauté locale et aborder les préoccupations des citoyens. Les CSC doivent viser à obtenir le soutien des différents segments de la population.

Faire d'un Plan d'Action Communautaire une réalité et améliorer la santé de la population exige diligence, détermination et planification collective. Ceci doit être un effort d'équipe. Le Plan d'Action Communautaire (PAC) doit être révisé et adapté tous les six (6) mois.

4.1. Planification de vos interventions routinières

L’établissement d’un plan pour vos interventions routinières doit se faire en même temps dans la même réunion du CSC. Cette réunion sera longue mais il est important que les Activités 5, 6, 7, 7, 8 et 9 soient réalisées ensemble. Organiser cette réunion à l’avance et demander aux membres du CSC de lire toutes les activités pour se familiariser à l’avance avec celles-ci.

Etapes à suivre dans la planification de vos interventions routinières

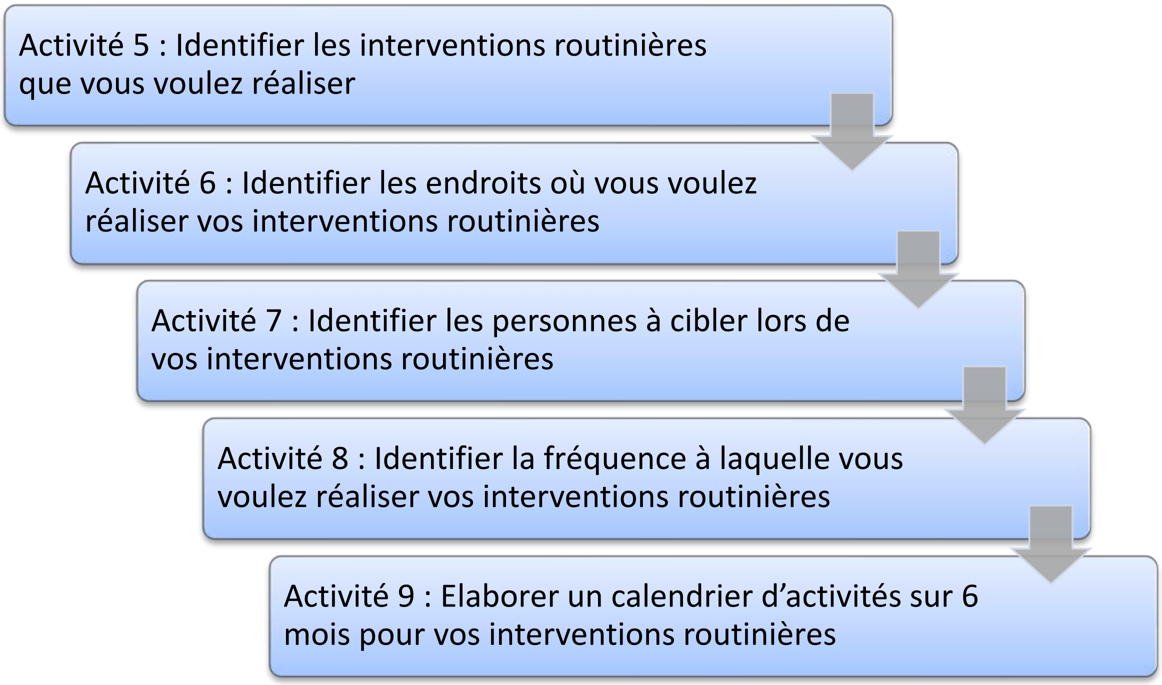

Activité 5 : Identifier les interventions routinières que vous voulez mettre en œuvre

Le point de départ de votre Plan d'Action Communautaire (PAC) est de convenir d'une série d'interventions routinières qui seront initiées dans la communauté par votre CSC pour sensibiliser les gens sur la malaria et promouvoir la prévention et le contrôle. Ces choses ont été énumérées au Chapitre III ci-dessus. Il est important que le CSC discute des options pour les activités anti-malariales et conviennent ensemble d'une série d'interventions. Il est nécessaire d'être très spécifique sur ce que vous envisagez de faire, plus c'est spécifique, plus c'est facile de planifier et d'exécuter.

Pour y arriver, veuillez réaliser l'**Activité 5**.

Activité 5 : Décider des activités anti-malariales de votre CSC

Objectif : Définir clairement les interventions planifiées par le CSC pour exécution dans leur territoire au cours des six (6) prochains mois.

Matériels nécessaires : Papier et plume.

Temps requis : Une (1) à deux (2) heures.

Description de l'activité : Cette activité constituera le début du Plan d'Action Communautaire (PAC). Le CSC doit réaliser cette activité dans le cadre d'une réunion du CSC en passant par les étapes suivantes :

1. Le groupe doit d'abord s'assurer que tout le monde ait lu la liste des interventions possibles du CSC prévues dans **l'activité 4** ci-dessus (Chapitre III).
2. Le CSC doit alors avoir une discussion ouverte. Ils doivent examiner chacune des interventions possibles et discuter des pour et des contre de chacune d'elles.
3. Ensuite, ils doivent convenir d'un nombre d'interventions fixe et remplir le **Formulaire d'Activité 5.** N'oubliez pas que ceci peut inclure autant d'intervenions que le CSC aimerait réaliser – il n'y a aucune limite autre que l'intérêt, le temps et les ressources du CSC.
4. Chaque CSC doit envisager la formation de sous-groupes pour certaines de ses interventions. Ceci peut aider le processus de planification bien que ce ne soit pas exigé mais peut quand même aider.

Résultat : Une fois cette activité terminée, le CSC devrait avoir une liste des interventions spécifiques qu'il exécutera dans la communauté au cours des six (6) prochains mois. Le **Formulaire d'Activité 5** doit être rempli.

Une fois que vous aurez décidé des interventions routinières de votre CSC, il vous faudra décider **où** vous avez l'intention d'exécuter ces activités, **qui** cibler pour ces interventions et **avec quelle fréquence** vous envisagez d'exécuter chacune de ces interventions.

Activité 6: Identifier les endroits pour la mise en œuvre de vos interventions routinières

Chaque CSC doit prioriser les quartiers où il y a eu des cas de malaria par le passé ou présentement pour leurs interventions routinières pour contrer la malaria de même que les quartiers qui sont considérés à « haut risque » à cause de la grande densité des moustiques. **La décision sur l'endroit où vous allez cibler vos activités doit être basée sur la liste des cas de malaria fournie par le MSPP.** Pour y arriver, veuillez suivre les instructions pour l'**Activité 6.**

Activité 6 : Décider des endroits à cibler pour vos activités anti-malariales

Objectif : Choisir trois (3) à six (6) quartiers que vous désirez cibler pour les activités anti-malariales des six (6) prochains mois.

Matériels nécessaires : Papier et plume.

Temps requis : 30 minutes

Description de l'activité : Pour réaliser cette activité, veuillez :

1. Revoir les résultats de votre affiche de facteurs de risque de malaria (Chapitre 3).
2. Etablir une liste de trois (3) à six (6) quartiers prioritaires qui ont un haut risque de malaria pour organiser les activités. Ceci doit être basé sur les cas rapportés par le MSPP. Cette liste peut aussi inclure certains quartiers qui ont une haute visibilité (large population, points de transit) où beaucoup de personnes peuvent entendre le message. Ces endroits doivent aussi être à une distance raisonnable des membres du CSC. Veuillez les noter sur le **Formulaire d'Activité 6**.

Résultat : Une fois cette activité terminée, le CSC devrait avoir une liste de trois (3) à six (6) quartiers prioritaires à cibler pour les activités anti-malariales au cours des six (6) prochains mois. Le **Formulaire d'Activité 6** doit être rempli.

Activité 7: Qui voulez-vous cibler pour vos interventions routinières?

Il est important de réfléchir sur la façon de cibler les interventions vers des groupes sociaux spécifiques. Dans certains cas, il serait bon de cibler toute la communauté. Dans d'autre cas, il faudra peut-être cibler des groupes sociaux spécifiques. Un moyen utile pour préparer vos interventions serait de réfléchir sur *qui* vous voulez cibler. Par exemple : ceux-ci pourraient inclure :

- Ecoliers et enseignants ;
- Agents de santé ;
- Leaders religieux ;
- Marchands/marchandes ;
- Organisations de jeunes ;
- Organisations communautaires ;
- Organisations de femmes ;
- Des groupes de génération de revenus ;
- Gouvernements locaux ;
- Regroupements agricoles ;
- Groupes culturels ;
- Ménages dans les endroits retirés.

Activité 7 : Décider qui cibler pour vos interventions anti-malariales

Objectif : Discuter des personnes que vous aimeriez cibler pour vos interventions anti-malariales et pourquoi.

Matériels nécessaires : Papier et plume

Temps requis : 30 minutes

Description de l'activité : Pour réaliser cette activité, veuillez :

1. Revoir la liste des interventions CSC que vous avez décidé d'exécuter selon **l'Activité 5**.
2. Pour chacune des informations, énumérez les types de personnes spécifiques que vous voulez cibler pour ces activités et la raison pour laquelle vous pensez qu'elles doivent être ciblées.
3. Les noter dans le **Formulaire d'Activité 7**.

Résultat : Une fois cette activité terminée, le CSC devrait avoir une meilleure appréciation de leur public et des groupes sociaux ciblés. Le **Formulaire d'Activité 7** doit être rempli.

Activité 8: Avec quelle fréquence mettrez-vous en œuvre vos activités routinières?

Il est important de considérer la fréquence avec laquelle vous devez organiser des interventions routinières spécifiques dans chaque endroit. Pour faciliter votre planification et votre exécution, vous devez viser à exécuter les mêmes interventions dans chaque quartier. Il faut considérer que ceci est un « package » d'interventions à exécuter dans chacun des quartiers que vous avez choisi.

Votre planification doit tenir compte du nombre de quartiers que vous avez choisi pour vos interventions CSC et le nombre d'interventions que vous avez choisi.

Il y a deux (2) façons d'organiser ces choses :

Option 1 : Chaque mois vous choisissez un quartier différent sur lequel vous concentrer. Là, vous exécuterez toutes les interventions que vous avez choisi. Le mois suivant, vous passez à un autre quartier, et ainsi de suite.

Par exemple, si vous avez choisi trois (3) quartiers, vous devez viser à concentrer vos interventions sur un (1) quartier par mois. Ceci veut dire que vous visiterez les trois (3) quartiers sur une période de trois (3) mois. D'un autre côté, si vous avez choisi six (6) quartiers, il vous faudra six (6) mois pour les visiter tous.

Option2 2 : Chaque mois vous allez choisir un (1) type d'intervention et l'exécuter dans tous les quartiers que vous avez choisi. Le mois suivant, vous passez à un autre type d'intervention et ainsi de suite.

Par exemple, vous avez choisi trois (3) quartiers et quatre (4) interventions différentes : 1) visites domiciliaires, 2) programme scolaire, 3) travail avec les pratiquants de médecine traditionnelle, et 4) assainissement environnemental. Dans ce cas, le premier mois, vous choisissez une (1) de ces interventions (éducation domiciliaire) et vous l'exécutez dans les trois (3) quartiers. Puis le mois suivant, vous choisissez la deuxième intervention (programme scolaire) et vous l'exécutez dans les trois (3) quartiers et ainsi de suite. Dans ce cas, il vous faudra quatre (4) mois pour réaliser toutes les interventions.

Activité 8 : Décider avec quelle fréquence vous allez exécuter vos interventions anti-malariales

Objectif : Discuter de la fréquence à laquelle vous allez exécuter les interventions anti-malariales du CSC.

Matériels nécessaires : Papier, plume

Temps requis : 30 minutes

Description de l'activité : Pour réaliser cette activité, veuillez :

1. Lire la description qui précède dans cette section.
2. Convenez clairement du nombre de quartiers que vous avez choisi pour vos interventions au cours des six (6) prochains mois et le nombre d'interventions différentes que vous avez choisi.
3. Avoir une discussion avec tous les membres du CSC à savoir si vous désirez suivre l'option 1 ou l'option 2 décrite ci-dessus.
4. Sur la base de cette discussion, vous voudrez peut-être réviser ou modifier le nombre total de quartiers et/ou de types d'intervention choisi par votre CSC. Ceci est correct et constitue une partie normale du processus de planification. Si tel est le cas, noter ce changement sur vos formulaires d'activités.
5. Ecrivez votre choix sur le **Formulaire d'Activité 8**.

Résultat : Une fois cette activité terminée, le CSC aura amélioré sa planification et son organisation et sera prêt à élaborer un Calendrier d'Activités qui est **l'Activité 9**. Le **Formulaire d'Activité 8** doit être rempli.

Activité 9: Elaborer un calendrier d’activités de 6 mois pour votre CSC

Vous êtes maintenant prêts à finaliser votre calendrier d’activités !

Ceci sera une combinaison du brainstorming et des décisions que vous avez pris dans les activités 5 à 8 pour préparer un calendrier mensuel clair. Rappelez-vous que chaque CSC doit planifier un minimum de cinq (5) interventions par mois incluant la réunion mensuelle du CSC. Vous aurez alors deux (2) options sur la façon de ce faire :

Option 1 : établir un calendrier d’activités de six (6) mois avec des dates et des endroits clairement définis pour chaque intervention. Ceci doit inclure une réunion mensuelle du CSC et un minimum de quatre (4) interventions (vous pouvez certainement planifier plus de quatre (4) interventions par mois si vous le décidez – ceci dépend de chaque CSC). Le bénéfice de cette option est qu’elle vous permet d’avoir décidé de tous vos plans à l’avance.

Option 2 : établir un calendrier d’activités de six (6) mois comportant les dates définies sur une base mensuelle. Dans cette option, vous décidez de la date, de l’heure et de l’endroit de vos réunions de CSC chaque mois, ce, six (6) mois à l’avance, mais vous planifiez vos interventions seulement le mois précédent. Il vous faudra convenir des dates et des heures spécifiques chaque mois lors de votre réunion de CSC pour le mois à venir. Le bénéfice de cette option est qu’elle vous permet d’avoir une flexibilité par rapport à la date et à l’heure exactes de vos plans.

Activité 9 implique que chaque CSC fasse une discussion sur ces deux (2) options et en choisissent une (1). **Il n'y a pas de rapport d'activité pour cette activité. Plutôt, chaque membre du CSC recevra un livret dans lequel écrire le calendrier officiel du mois.**

La programmation des interventions du CSC doit tenir compte du :

- Jour et heure de l’intervention ;
- Lieu de l’intervention ;
- Nom de l’intervention (type d’intervention) ;
- Nom de n’importe lequel des membres du CSC responsable de mener les activités sur le terrain si tel est le cas.

Chaque membre du CSC recevra un livret dans lequel écrire le calendrier officiel du mois. Ils doivent aussi transmettre les informations de chaque intervention aux membres de la communauté de chaque quartier à l’avance pour permettre aux membres de la communauté locale et aux membres du CSC de se tenir prêts.

Chaque CSC doit aussi noter dans leur calendrier les jours de marché importants, les jours de fêtes champêtres et les évènements qui se tiennent les jours fériés qui ont une importance

pour la communauté. Ils doivent aussi essayer d'inclure certaines interventions lors de ces jours importants.

Il est essentiel que chaque CSC ait un plan très clair sur le moment où ils exécuteront leurs interventions. Ceci leur permettra de contacter les volontaires CSC qu'il faut et de planifier les matériels pédagogiques ou autres choses nécessaires.

4.2. Elaborer un plan de réponse rapide à la malaria

Le Plan d'Action Communautaire (PAC) comporte trois (3) parties. A date, nous nous sommes concentrés sur les interventions routinières. Mais, le CSC doit aussi avoir un plan différent – un plan sur la façon de répondre aux cas de malaria ou à une épidémie de malaria dans la communauté. Ceci s'appelle **Plan de Réponse Rapide du CSC.**

Le Plan de Réponse Rapide du CSC doit être discuté lors d'une réunion du CSC.

Activité 10: Elaborer un plan de réponse rapide du CSC

Le Plan de Réponse Rapide du CSC est une série d'interventions que le CSC a convenu d'exécuter dans les quartiers où il y a eu un (1) ou plusieurs cas de malaria diagnostiqué(s). Le Plan de Réponse Rapide sera initié en collaboration avec le MSPP. Il s'agit d'une série d'activités d'urgence pour limiter la propagation de la malaria dans la communauté.

Le Plan doit inclure les interventions suivantes :

- Propager la sensibilisation sur la malaria et augmenter les connaissances de la communauté locale ;
- Aider à la surveillance et au traitement dans la communauté locale ;
- Aider à la prévention des moustiques dans la communauté locale ;
- Renforcer la solidarité communautaire ;
- Maintenir la surveillance et le soutien à long-terme pour monitorer les cas futurs de malaria.

Selon les circonstances et les discussions avec le MSPP, il se peut que le CSC s'engage activement à assurer que les membres de la communauté qui ont des symptômes similaires à la malaria soient rapidement diagnostiqués, et, s'ils sont positifs, traités par le personnel de santé.

Les membres du CSC doivent élaborer leur propre série d'interventions qu'ils ont l'intention d'exécuter dans le cadre de leur Plan de Réponse Rapide CSC. Veuillez suivre les instructions de **l'Activité 10** et convenir d'un Plan de Réponse Rapide du CSC.

Ce Plan doit couvrir :

- Une phase d'activités intensives sur une période de deux (2) semaines dans le but de localiser d'autres cas de malaria ;
- Une phase d'activités de plus sur une période de trois (3) mois ;
- Une phase à long-terme d'un (1) an. Cette phase comprend l'incorporation du quartier pour cibler d'autres quartiers pour les interventions routinières.

Activité 10 : Convenir d'un Plan de Réponse Rapide du CSC

Objectif : Convenir d'un plan et d'une série d'interventions anti-malariales pour répondre aux cas actifs de malaria.

Matériels nécessaires : Papier et plume

Temps requis : 1 heure

Description de l'activité : Pour mener cette activité, veuillez :

1. Lire attentivement la description qui précède dans cette section.
2. Réviser la liste des interventions du CSC au Chapitre III,
3. Discuter des bénéfices et des défis de chaque intervention lorsque vous répondez à un cas de malaria.
4. Discuter des interventions à prioriser pour la période intensive de deux (2) semaines.
5. Discuter des interventions à prioriser pour la période de trois (3) mois.
6. Utiliser le **Formulaire d'Activité 10** pour vous guider.

Résultat : Une fois cette activité terminée, le CSC aura un Plan de Réponse Rapide complet. Ceci doit être reflété sur le **Formulaire d'Activité 10** et servir de base pour n'importe quelles activités de réponse rapide.

Activation et mise en œuvre du plan de réponse rapide du CSC

Une fois que le MSPP vous aura communiqué la présence d'un cas de malaria dans votre territoire, vous devez activer le Plan de Réponse Rapide du CSC :

Etapes nécessaires à l'activation du Plan de Réponse Rapide du CSC

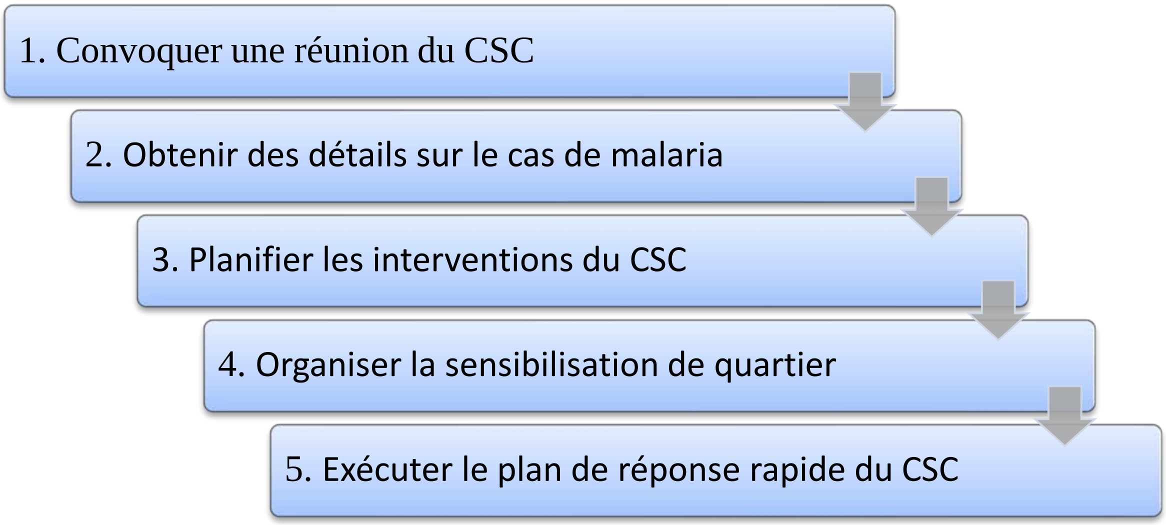

- **ETAPE 1 : <u>Convoquer une réunion du CSC aussi vite que possible.</u>** Inviter le personnel du MSPP à cette réunion ; organiser la réunion dans la communauté.

- **ETAPE 2 : <u>Connaître tous les détails du cas de malaria.</u>** Avant la réunion, chercher à avoir les informations suivantes :

  - Combien de cas de malaria ont été diagnostiqués récemment dans cette zone ?
  - Quels sont les détails des cas – âge, sexe, occupation et lieu de résidence ?
  - Que planifie le MSPP pour aborder cette flambée de malaria ?

- **ETAPE 3 : <u>Planifier les interventions du CSC pendant la réunion.</u>** Faire ceci en collaboration avec le personnel local du MSPP, les représentants de la communauté et les groupes de la société civile.

- **ETAPE 4 : <u>Organiser une sensibilisation dans les quartiers avoisinant les cas de malaria.</u>** La première intervention doit toujours être une réunion au niveau du quartier où la personne atteinte de malaria vit avec des membres de la communauté. A cette réunion, le CSC doit essayer de mobiliser la population locale à s'engager dans les interventions envisagées par le CSC. Un groupe de leaders locaux et de citoyens concernés doit être organisé. Ce groupe doit être appelé « Equipe d'Assistance Rapide de Quartier » ou quelque chose de ce genre.

- **ETAPE 5 : <u>Exécuter le Plan de Réponse Rapide du CSC.</u>** En travaillant avec l'Equipe d'Assistance Rapide de Quartier » il sera nécessaire de décider de la fréquence de ces activités et d'établir un calendrier de travail. Comme pour les interventions routinières, la programmation de ces interventions rapides doit tenir compte de ce qui suit :
    - Jour et heure de l'intervention ;
    - Lieu de l'intervention ;
    - Nom de l'intervention (type d'intervention) ;
    - Nom de n'importe quels membres du CSC responsables de mener les activités sur le terrain, le cas échéant.

En planifiant ces activités, vous devez aussi considérer l'endroit où vous planifiez de les exécuter, ceux que vous voulez cibler, et la fréquence à laquelle vous voulez exécuter chaque intervention dans la communauté, comme discuté ci-dessus pour les interventions routinières.

4.3. Elaborer un plan pour les matériels et les ressources

La troisième et dernière partie de votre Plan d'Action Communautaire CSC est un **<u>Plan pour les Matériels et les Ressources</u>**. Pour y arriver, veuillez suivre les instructions de **<u>l'Activité 11</u>** et les étapes ci-dessous. Ceci doit être fait à une réunion du CSC.

Les CSC sont des organisations bénévoles, ils dépendent de leurs membres et de leurs volontaires pour s'engager dans la lutte contre la malaria et travailler à l'amélioration de la santé dans leurs communautés. Pour atteindre les objectifs de votre CSC, il est important de discuter et de planifier ce qu'il vous faut comme matériels, fournitures et équipements.

<u>Rappelez-vous que le MSPP et les partenaires pourraient ne pas pouvoir satisfaire vos demandes ou qu'ils pourraient seulement le faire partiellement.</u> Discuter de ceci avec votre point focal du MSPP. Quel que soit le cas, il est important de planifier à l'avance pour vous permettre d'exécuter effectivement vos interventions.

Activité 11 : Elaborer un Plan pour les Matériels et les Ressources

Objectif : Elaborer un Plan pour les Matériels et les Ressources qu'il faut pour les interventions du CSC.

Matériels nécessaires : Papier et plume

Temps requis : 1 à 2 heures

Description de l'activité : Pour mener cette activité, veuillez :

1. Lire les trois (3) étapes ci-après décrites, et suivre les instructions.
2. Utiliser le **Formulaire d'Activité 11** pour vous guider.

Résultat : Une fois cette activité terminée, le CSC aura un Plan complet pour les Matériels et les Ressources. Ceci doit être reflété dans le **Formulaire d'Activité 11** et être continuellement révisé et actualisé. Ceci doit être consigné par écrit.

Etape 1: Demande de matériels pédagogiques

Les matériels pédagogiques constituent une partie importante pour la plupart des interventions du CSC. Travaillant avec votre point focal du MSPP, vous devez réviser le matériel pédagogique disponible concernant la malaria, de même que n'importe quel autre matériel de promotion de la santé que vous pourriez avoir pour distribution (par exemple pour d'autres maladies). Demander au point focal du MSPP d'apporter une (1) copie de chaque matériel et discuter de la quantité que vous pouvez recevoir chaque mois.

Matériel pédagogique	Mois 1	Mois 2	Mois 3	Total requis pour trois (3) mois
Brochures				
Flyers				
Affiches				

Faire un plan de trois (3) mois pour les fournitures qu'il vous faut avec votre point focal du MSPP pour vous assurer que vous ayez toujours suffisamment de matériel. Utiliser le format ci-dessus.

Ensuite, et particulièrement s'il y a un manque de matériels disponibles, penser à préparer et à imprimer vos propres matériels pédagogiques. Il n'est pas nécessaire qu'ils soient sophistiqués. En discuter avec votre point focal du MSPP.

Etape 2: Demande de fournitures et de matériels

Pour que votre CSC fonctionne, il vous faudra des fournitures et des matériels de base. Ceci inclut trois (3) catégories :

A. **<u>Fournitures de planification :</u>** livres, papiers, plumes/crayons, affiches.
B. **<u>Matériels d'exécution :</u>** t-shirts, badges, kits scolaires, kits de nettoyage de l'environnement, etc.
C. **<u>Equipements spéciaux pour contrôler la maladie :</u>** tests de diagnostic de la malaria, médicaments, etc.

A. <u>Planification des fournitures :</u>

Ces fournitures doivent être utilisées par les CSC pour planifier et organiser les interventions et les réunions en suivant les instructions fournies dans ce manuel. Les fournitures dépendront des ressources disponibles du MSPP et des partenaires d'exécution.

Le CSC recevra les fournitures suivantes :

- Un registre de présence ;
- Un livre à utiliser pour refléter le réseau des volontaires ;
- Un livret de calendrier mensuel pour planifier les interventions ;
- Deux (2) tableaux d'affichage.

Tous les trois (3) mois, le point focal du MSPP fournira au CSC (si possible) :

- Un livre pour chaque membre du CSC comportant un calendrier annuel ;
- Deux (2) plumes pour chaque membre du CSC.

D'autres ressources que le MSPP et les partenaires d'exécution peuvent fournir incluent (selon les ressources financières disponibles) :

- Un temps d'antenne pour chaque membre ;
- Des frais de transport ;
- Des boissons pour les réunions.

B. <u>Matériels d'exécution :</u>

Les matériels d'exécution sont des matériels de soutien que les CSC aimeraient utiliser pour faire leurs interventions dans la communauté. Il faudra en discuter avec votre point focal du MSPP pour voir si ces ressources sont disponibles. Cependant, il est important de considérer les matériels d'exécution que vous désirez obtenir, et qui peuvent inclure des t-shirts, des badges, des kits scolaires, des kits de nettoyage de l'environnement et d'autres. En discuter avec votre point focal du MSPP.

C. **Fournitures, matériels, et équipements spéciaux pour le contrôle de la maladie :**

Bien que les membres du CSC n'utilisent pas les matériels de diagnostic, de traitement et des médicaments, ils peuvent aider le personnel local médical et de santé publique à noter les raretés ou les besoins spéciaux et faire un plaidoyer pour leurs communautés. En ce sens, les membres du CSC sont encouragés à discuter des raretés et des besoins importants avec leurs collègues sanitaires locaux et de discuter de ces demandes avec leur point focal du MSPP.

N'importe quelle rareté ou rupture de stock de tests de diagnostic de la malaria ou de médicaments dans les cliniques et les hôpitaux locaux doit être communiqué au point focal du MSPP dès que possible. Ceci peut être ajouté aux demandes faites au MSPP.

Etape 3: Définir les ressources de la communauté et les contributions en nature

Il est important que les CSC ne dépendent pas exclusivement du MSPP et des partenaires pour toutes les fournitures et toutes les ressources nécessaires pour être effectifs dans leurs communautés. Les membres des CSC doivent plutôt réfléchir sur la façon dont ils peuvent obtenir des ressources des groupes communautaires existants, du gouvernement et de la société civile.

Par exemple, ceci pourrait inclure des équipements de nettoyage et du personnel du gouvernement local, des salles de classe pour les rencontres communautaires, de la nourriture et autres produits des ONG locales. Une contribution importante en nature est l'utilisation d'un tableau ou de feuilles volantes pour les réunions du CSC ; ceci peut être négocié avec une école ou un autre bâtiment local. Les ressources communautaires et les contributions en nature doivent être discutées pour chaque intervention et négociées avec les volontaires appropriés du CSC et les autres membres de la communauté.

CHAPITRE 5 : Exécuter le Plan d'Action Communautaire.

Dans ce chapitre, nous réviserons succinctement certains principes importants essentiels à la réussite de votre CSC, au fur et à mesure que vous exécuterez votre Plan d'Action Communautaire.

5.1. Commencer modestement et étendre

Il est très important que votre CSC exécute les activités de sorte à maintenir la motivation de ses membres. Le CSC ne doit pas être débordé, mais se concentrer à commencer sur une petite échelle en exécutant des activités réussies et les augmenter par la suite. Il doit apprendre au fur et à mesure et utiliser cet apprentissage pour se renforcer.

Il faut que chaque CSC développe son propre « style » de travail.

Il est important de se concentrer sur les cas de malaria et les zones à haut risque qui ont un historique de malaria.

5.2. Créer une voir régulière de communication avec le MSPP

Vous devez établir un contact serré et régulier avec le bureau du MSPP. Le bureau du MSPP devrait fournir à votre CSC des rapports mensuels sur les cas de malaria. Si une clinique dans votre zone ne rapporte pas des cas de malaria soupçonnés, confirmés et/ou traités, le CSC (en accord avec le MSPP), doit discuter sur la manière de les porter à rapporter ces chiffres au MSPP sur une base mensuelle.

Chaque réunion mensuelle doit commencer avec une mise à jour épidémiologique fournie par le MSPP.

5.3. Faites preuve de créativité! Incorporer l'art, la musique et la culture haïtiennes

Il est important d'encourager la créativité dans la façon de vous organiser et d'exécuter vos interventions. Plus spécifiquement, les membres du CSC doivent être encouragés à prendre des initiatives, à trouver des idées nouvelles et intéressantes et à mobiliser les communautés de sorte à puiser dans les forces uniques de la culture haïtienne et des idées de la communauté. Ceci inclut des slogans, des chansons, des danses, des pièces de théâtre et des drames, des comédies, des peintures murales, des poèmes et des arts visuels. Faire en sorte que les choses soient amusantes et intéressantes.

5.4. Activités dans les écoles

Une bonne possibilité serait d'organiser des séances d'éducation et des activités avec les écoliers en les engageant par exemple dans le nettoyage de l'environnement, la divulgation de la sensibilisation et les informations sur la malaria. Chaque CSC devrait inclure des activités dans les écoles dans leur routine et leur Plan de Réponse Rapide. Impliquer les dirigeants et les professeurs d'écoles et les inclure dans la planification.

5.5. Nettoyage de l'environnement

Le nettoyage de l'environnement est une activité importante qui demande beaucoup de travail. Il faut considérer l'équilibre entre cette grande quantité de travail et les bénéfices pour les efforts contre la malaria et la santé communautaire en général. Il est important de choisir les gîtes les plus importants et se concentrer là-dessus autrement, la tâche accablante.

Il faut absolument se rendre compte que le nettoyage de l'environnement n'est pas tout à fait efficace contre les moustiques anophèles qui propagent la malaria. On trouve aussi d'autres espèces de moustiques dans les communautés qui se reproduisent dans des habitats différents en comparaison avec les *Anophèles.* Par exemple, les moustiques *Aedes* propagent la Chikungunya, le Zika, et la Dengue. Les moustiques *Culex* propagent la filariose lymphatique.

Chaque CSC doit discuter de leur plan de nettoyage de l'environnement avec le MSPP pour s'assurer que leurs efforts soient bénéfiques pour le contrôle des moustiques. Ils doivent consulter le matériel pédagogique fourni qui parle des différences entre les *Anophèles*, les *Aedes* et les *Culex* et ce que cela implique pour la planification du nettoyage de l'environnement.

5.6. Suivre les cinq principes pour engager tous les secteurs de la société

La réussite des interventions du CSC dépendra des cinq (5) principes soulignés ci-après pour engager les membres de la communauté. Les membres du CSC ne doivent pas agir comme des experts, ils doivent plutôt être des **facilitateurs** qui motivent les membres de la communauté à s'engager. Ils doivent viser à **déclencher l'enthousiasme et l'énergie.** Ils doivent établir la confiance et former des amitiés avec la population. Il ne faut pas qu'ils soient sévères ou qu'ils s'adonnent à des reproches. Ils doivent être sincères et ne pas induire les gens en erreur pour créer de fausses attentes d'aide ou de bénéfices pour leur participation. Ils doivent faire appel au sens civique des gens et à leur devoir les uns envers les autres.

L'exécution du Plan d'Action Communautaire du CSC doit être basée sur cinq (5) principes :

1) **Honnêteté :** les membres des CSC doivent agir avec intégrité et toujours dire la vérité.
2) **Ecoute :** les membres des CSC doivent écouter et faire montre d'empathie pour les préoccupations, les idées et les croyances de la communauté.
3) **Partenariat :** les membres des CSC doivent créer des partenariats fermes entre les CSC et les groupes communautaires.
4) **Transparence :** les membres des CSC doivent toujours donner une image claire à la population et ne rien leur cacher.
5) **Résolution de problème :** les membres des CSC doivent faire de leur mieux pour travailler avec les membres de la communauté pour résoudre les problèmes.

CHAPITRE 6 : Etablir des groupes CSC de quartier

Comme mentionné à travers ce manuel, les membres des CSC doivent travailler en étroite collaboration avec les membres de la communauté, en particulier dans les points chauds de la malaria.

Etapes à suivre pour l'établissement des groupes de CSC dans les quartiers

1. Choisir les volontaires pour diriger le CSC
2. Organiser un réseau de volontaires de quartier
3. Lancer le groupe de CSC de quartier
4. Soutenir les groupes de quartier

6.1. Choisir des volontaires pour diriger les CSC dans chaque quartier

Dans les quartiers choisis où votre CSC doit travailler, il est important d'établir un groupe de volontaires composé de résidents du quartier et dévoués au travail du CSC en réalisant des interventions dans leur quartier. Ce groupe doit être appelé **Groupe de Volontaires du Conseil de Santé Communautaire** (ou quelque chose de semblable)

Rappelez-vous que le travail le plus important des membres du CSC sera de mobiliser et de stimuler les groupes de volontaires du CSC à s'engager dans les interventions du CSC. Ce qui ne veut pas dire que les membres du CSC ne travailleront pas dur sur le terrain, les membres du CSC doivent tracer l'exemple pour les volontaires du CSC. Le rôle des membres du CSC est d'organiser et de motiver ces personnes et ces groupes communautaires.

- **<u>ETAPE 1 : choisir trois (3) volontaires pour être les leaders.</u>** La première étape dans l'établissement d'un CSC de quartier est de choisir 3 leaders volontaires pour agir en qualité de leaders et mobilisateurs informels du groupe dans leur quartier. Ces 3 personnes doivent :
    - Être non-sectaires et apolitiques ;
    - Être choisies pour représenter une section tout à fait transversale de la communauté sans discrimination ;
    - Avoir une très bonne réputation au niveau de la communauté ;
    - Pouvoir travailler dans un esprit de volontariat et de compassion ;
    - Être acceptées par les gens de la localité ;
    - Résider dans la communauté.

6.2. Organiser un réseau de volontaires de quartier pour les CSC

- **<u>ETAPE 2 : organiser un réseau de volontaires locaux.</u>** Le choix des trois (3) leaders volontaires du CSC doit être déterminé avant le lancement officiel de chaque groupe de volontaires CSC. Les membres du CSC doivent visiter les trois (3) champions volontaires du CSC et établir une liste de personnes du quartier à inviter à l'évènement de lancement du groupe de volontaires du CSC du quartier. Ils doivent faire en sorte d'inviter des personnes, des organisations et institutions sociales et culturelles suivantes :
    - Ecoles ;
    - Eglises ;
    - Organisations de jeunes ;
    - Groupes de femmes ;
    - Groupes et coopératives agricoles ;
    - Groupes culturels ;
    - Groupes musicaux ;
    - Clubs de football ;
    - Groupes de nettoyage ;
    - Groupes de santé ;
    - Gouvernements locaux ;
    - Personnel médical;
    - Guérisseurs traditionnels.

La qualité de membre ne doit pas être exclusive. Il faut se rappeler que tout le monde peut se joindre au groupe. Plus il y a de volontaires CSC dans le quartier, mieux ça vaut ! Les leaders volontaires du CSC propageront la nouvelle du nouveau CSC de quartier au cours de la prochaine semaine. Ils inviteront autant de membres de la communauté que possible à la première réunion de lancement.

Si un groupe de santé communautaire existe déjà dans le quartier, des efforts doivent être déployés pour voir si les objectifs de contrôle de la malaria peuvent être intégrés dans ce groupe qui existe déjà. Au lieu de créer un groupe à part, les membres du CSC doivent envisager la possibilité de travailler avec le groupe existant. Si le groupe existant est très spécifique par rapport à un problème de santé (comme la santé de la femme), il serait peut-être mieux de créer un groupe à part. Dans ce cas, il est toujours important d'obtenir que ce groupe de santé soit actif dans le CSC du quartier.

6.3. Lancement officiel du groupe CSC de quartier

- **ETAPE 3 : lancer le groupe CSC de quartier.** Le lancement officiel du groupe de volontaires du CSC du quartier doit être organisé à l'avance pour inclure des activités qui sensibilisent sur le rôle du CSC et de la malaria. Ceci pourrait inclure :
    - Une parade avec de la musique ;
    - Des annonces dans les églises ; et
    - L'implication des élèves d'école et des enseignants.

Lors du lancement, les membres du CSC et les représentants du MSPP doivent introduire l'approche du CSC, discuter des faits importants de la malaria et présenter les diverses interventions que le CSC a l'intention de réaliser dans le quartier.

Les membres du CSC doivent faire en sorte qu'il y ait une quantité adéquate de matériels éducatifs disponibles pour distribution à tous les membres de la communauté qui assistent à l'évènement et que des copies supplémentaires soient disponibles pour permettre aux volontaires du CSC de les distribuer pendant la parade.

Un plan de suivi doit être fait avec les leaders volontaires du CSC afin de commencer la première intervention officielle du CSC.

6.4. Groupes de volontaires CSC de quartier très actifs

La plupart des groupes de volontaires de quartier du CSC ne travailleront pas de manière indépendante mais lorsqu'ils sont mobilisés par les membres du CSC. Si les membres de la communauté veulent s'organiser eux-mêmes et réaliser leurs propres interventions anti-malariales en-dehors des plans du CSC, le CSC doit en discuter avec le point focal du MSPP. Seuls les groupes qui ont démontré une capacité et un dévouement exceptionnel doivent être encouragés à s'auto-former en groupe de CSC de quartier plus actif. Ceci ne doit pas être découragé, cependant, il faut faire très attention de superviser et de gérer ces groupes de volontaires.

Si les groupes de volontaires de quartier du CSC veulent travailler sans l'implication physique directe des membres du CSC, il faut établir un plan avec le CSC. Dans ce cas, les groupes de quartier du CSC doivent faire des activités d'intervention une (1) fois toutes les une (1) à deux (2) semaines au minimum. Les groupes de quartier du CSC peuvent vouloir créer des sous-groupes comme des groupes de jeunes et des groupes d'église qui peuvent travailler de manière indépendante dans la communauté sur des interventions spécifiques.

Dans ce cas, chaque groupe de quartier du CSC aura besoin de matériels et de fournitures pour les aider dans leurs interventions, incluant :

- Une bannière et deux (2) drapeaux pour sensibiliser sur les réunions et à parader lors des interventions communautaires ;
- Un livret pour inscrire les membres de la communauté qui participent et tenir une liste de tous les membres formels du CSC de quartier ;
- Du matériel d'éducation sur la santé.

Dans ce cas, un plan doit être élaboré avec chaque CSC de quartier sur la fréquence à laquelle ils se rencontreront et sur les différents types d'intervention qu'ils veulent initier et effectuer. Pour entamer la discussion, les membres du CSC doivent présenter la liste des interventions au CSC de quartier et faciliter une discussion avec eux sur les forces et les défis de chaque type d'intervention.

Les interventions du CSC de quartier ne doivent pas être considérées comme étant séparées des interventions du CSC. Il est extrêmement important que les membres du CSC soient pleinement engagés dans toutes les interventions du CSC de quartier et participent régulièrement à leurs interventions.

6.5. Réaliser des activités dans des zones où il n'y a pas de groupe CSC

Il se peut que certaines activités doivent être initiées dans des endroits où il n'y a pas de groupes actifs de CSC de quartier. Ceci peut se produire dans les grandes villes lors de fêtes et de jours fériés majeurs. Il peut aussi se produire en réponse à un cas actif de malaria. Si tel est le cas, les membres du CSC doivent s'arranger pour que les volontaires de quartier et les groupes de CSC engagés dans les activités de contrôle de la malaria dans d'autres endroits soient présents et un plan élaboré.

6.6. Travailler avec le MSPP et autres professionnels de la médecine au niveau des quartiers

Il est important que les membres du CSC travaillent en collaboration avec le MSPP et d'autres professionnels de la médecine dans l'organisation et le contrôle de chaque groupe de CSC de quartier. Chaque groupe de quartier doit aussi travailler directement en collaboration avec le personnel local du MSPP, les cliniques privées locales, les guérisseurs traditionnels, les agents de santé communautaires, et n'importe quels autres groupes de santé communautaires qui existent déjà. Travailler avec ces groupes est extrêmement important pour améliorer le diagnostic et le traitement de la malaria.

6.7. Initier le plan de réponse rapide avec le groupe de volontaires du CSC

Une fois que le MSPP avertit le CSC d'un cas de malaria possible ou confirmé, les membres du CSC doivent contacter les leaders des groupes du CSC de quartier de cet endroit pour commencer le processus d'initiation du Plan de Réponse Rapide à la malaria. Au cas où il n'y aurait aucun groupe de CSC de quartier dans cet endroit spécifique, le ou les groupes le(s) plus proche(s) doivent être contactés et engagés. Dans ce cas, des efforts doivent être déployés pour créer rapidement un groupe de quartier en engageant les membres les plus proches du réseau de volontaires du CSC.

CHAPTER 7: Supervision et Apprentissage

Tous les programmes de santé publique réussis tiennent des registres détaillés et collectent plusieurs types d'informations incluant la réaction des membres de la communauté. **Ceci s'appelle monitoring,** et nous donne une idée de l'efficacité des interventions dans l'atteinte des objectifs.

Les informations sont cruciales pour apprendre et s'adapter aux besoins de la communauté. Elles peuvent aussi aider à motiver les personnes impliquées dans l'exécution des plans du CSC. La motivation et un sens de responsabilité sont très importants pour obtenir les résultats que l'on veut.

7.1. Prix et évaluation

Les CSC sont formés par des groupes de volontaires. Ils ne reçoivent ni salaire, ni *per diem* pour leur travail. Cependant, le MSPP et les partenaires d'exécution pourraient considérer (selon la disponibilité des ressources financières) d'offrir des prix aux CSC qui ont une excellente performance. Si cette décision est prise, ceci doit se faire tous les six (6) à douze (12) mois pour les trois (3) meilleurs CSC dans chaque département. Ces prix, doivent être de trois (3) catégories : or, argent et bronze.

Dans ce cas, le CSC sera évalué sur la base des critères suivants par l'équipe de supervision, le MSPP et les partenaires d'exécution :

- Nombre et qualité des interventions ;
- Acceptation communautaire ;
- Impact des interventions, incluant les cas de malaria ;
- Opportunité du rapportage et interaction avec le MSPP et les partenaires.

Les prix seront distribués aux réunions où tous les membres des CSC du département sont présents. A ce moment-là, le MSPP et les partenaires feront une présentation sur l'état actuel du programme CSC et fera une formation pour aborder les problèmes et les préoccupations spécifiques. Le MSPP et les partenaires d'exécution doivent envisager de fournir des frais de voyage aux membres des CSC si les moyens financiers sont disponibles.

7.2. Supervision du programme et rapportage

Le programme CSC sera supervisé par le personnel du MSPP et le personnel du TCC fournira une assistance aux CSC. Ceci doit inclure un point focal du MSPP de la commune de même qu'un superviseur au niveau de l'arrondissement. Les superviseurs aideront à l'organisation et à la planification du CSC et collecteront les rapports mensuels et tous les formulaires d'activités

mentionnés dans ce manuel. Les rapports mensuels seront communiqués à l'équipe de supervision par téléphone portable. **Les détails sont fournis dans un manuel de monitoring et d'évaluation (M&E), un document additionnel qui accompagne le manuel d'exécution.**

7.3. Activités de recherche sur le feedback des communautés

Certains CSC seront choisis pour faire partie d'un système de *feedback* communautaire. Ceci impliquera un personnel spécialisé du MSPP et des partenaires d'exécution qui visitent le CSC à des intervalles réguliers tous les trois (3) mois. Lors de ces visites, l'équipe de *feedback* communautaire travaillera avec le CSC pour initier des discussions et des entrevues dans la communauté avec les volontaires du CSC, différents groupes communautaires et des ménages individuels. Ces informations seront utilisées par le personnel du programme pour améliorer le fonctionnement du programme CSC. Certaines de ces données seront partagées avec le CSC. L'équipe de *feedback* communautaire expliquera ces activités en détail lors de leurs visites aux CSC. Pour rappel, ces détails sont soulignés dans le manuel M&E.

7.4. Données du MSPP sur la malaria

Le MSPP collecte des informations de routine des cliniques de santé incluant le nombre de personnes testées pour la malaria, le nombre de cas positifs et le nombre de cas traités. Ces informations seront routinièrement partagées avec chaque CSC par le point focal du MSPP. Ces données doivent être présentées lors des réunions mensuelles du CSC afin d'informer les membres des CSC sur l'épidémiologie de la malaria dans le département et les sections rurales.

7.5. Apprentissage et adaptation

Au bout de chaque six (6) mois, le MSPP et le CSC examineront les progrès du Plan d'Action Communautaire pour évaluer les réussites et les défis que le CSC a rencontré dans l'exécution de son plan. Ceci doit être considéré comme une opportunité d'adapter les Plans d'Action Communautaire.

A cette réunion, le point focal du MSPP et les partenaires doivent présenter certaines données de monitoring et d'évaluation et une actualisation du programme CSC en général. Les membres du CSC doivent pouvoir poser des questions sur le programme et faire des suggestions pour améliorer son fonctionnement.

En outre, une autre opportunité d'améliorer le programme de CSC sera soulevée lors des réunions de conférence routinière des CSC (ceci devrait se faire tous les six (6) à douze (12) mois) ; chaque membre du CSC doit y assister. Ceci dépendra des ressources financières disponibles.

7.6. Adapter votre CSC à d’autres problèmes de santé au-delà de la malaria

Une fois que les CSC auront exécuté tous les aspects de ce manuel, ils auront la possibilité de décider s’ils veulent étendre leur concentration au-delà de la malaria et inclure d’autres problèmes de santé qui existent dans leur communauté. Des directives sur la façon de ce faire seront fournies à certains CSC choisis dans la deuxième année du programme après discussion avec leur point focal du MSPP.

Annexe

1. Organigramme du Programme des Conseils de Santé Communautaire en Haïti

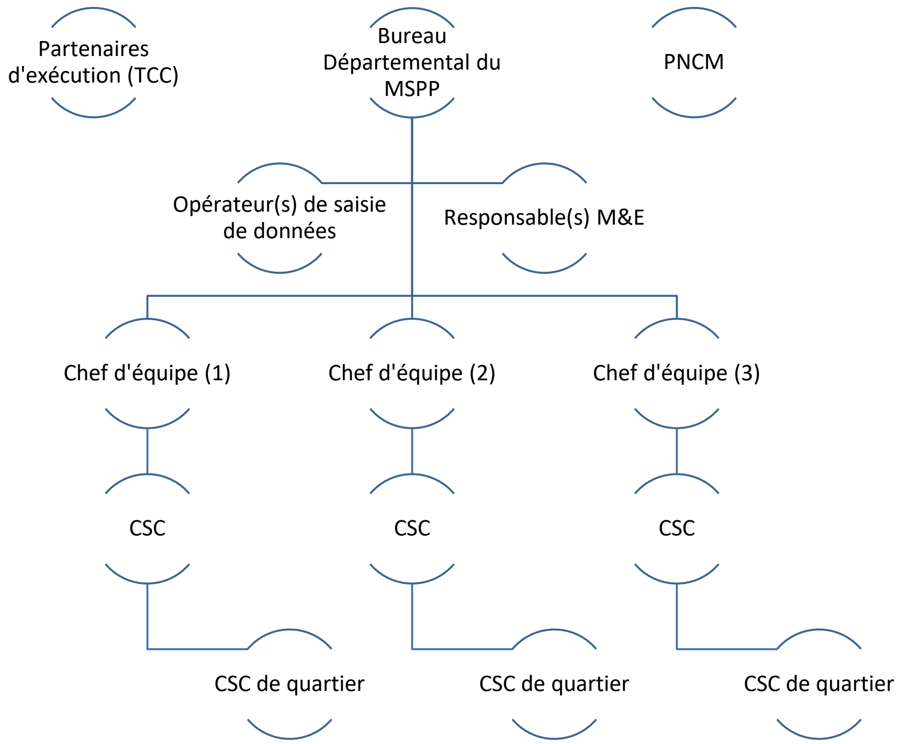

<u>Description :</u> le programme CSC doit être organisé à travers le Bureau Départemental du MSPP dans chaque département avec l'appui du PNCM et les partenaires d'exécution incluant le Carter Center.

PNCM et partenaires d'exécution (Carter Center) : responsables de fournir le soutien technique au MSPP, incluant la formation, le monitoring et l'évaluation, la supervision du personnel, et gérer les problèmes et préoccupations émergeants.

Coordonnateur Départemental du MSPP : responsable de tous les aspects techniques et administratifs de la mise en œuvre du programme sur le terrain. Ceci inclut la mobilisation du personnel existant au niveau départemental et local.

Responsable M&E du MSPP : basé au Bureau Départemental du MSPP et responsable de suivre tous les protocoles de monitoring et d'évaluation reflétés dans le manuel M&E et assuré des données et des reportages robustes concernant le monitoring et l'évaluation.

Opérateur de saisie de données du MSPP : basé au Bureau Départemental du MSPP et responsable d'aider à tous les aspects de rapportage du programme en fournissant une assistance

quotidienne du Bureau du Coordonnateur et de l’Officier responsable du monitoring et de l’évaluation.

Leaders d’équipe : personnels existants du MSPP au niveau communal et répondent au Coordonnateur du CSC du MSPP. Responsable de toute la supervision sur le terrain au jour le jour et du soutien aux CSC, dont la fourniture de soutien technique aux CSC et l’assistance à la formation.

Membres du CSC : il doit y avoir neuf (9) à treize (13) membres du CSC qui représentent tous les secteurs de la société.

Groupes de volontaires de quartier du CSC : ces groupes doivent être organisés dans des quartiers où il y a des cas de malaria actifs ou récents. Chaque groupe doit avoir trois (3) leaders volontaires de CSC pour servir de liaison entre la communauté et les CSC.

2. Checklist du Rapport d'Activité

**Numéro de l'Activité**	**Nom de l'Activité**	**L'activité est-elle réalisée?**	**Le rapport de l'activité a-t-il été soumis ?**
Activité 1	Développer une vision de votre CSC		
Activité 2	Cartographier les facteurs de risque de malaria dans votre zone		
Activité 3	Elaborer une liste de questions en suspens posées par les membres du CSC sur la malaria		
Activité 4	Discussion des forces et des faiblesses des approches à la malaria	- Sans objet -	
Activité 5	Identifier les interventions routinières		
Activité 6	Identifier les endroits où vous voulez réaliser vos interventions		
Activité 7	Identifier les personnes à cibler		
Activité 8	Identifier la fréquence de vos activités		
Activité 9	Elaborer un calendrier des activités sur une période de 6 mois	Les CSC devraient recevoir des livrets de calendrier pour cette activité	
Activité 10	Plan de réponse rapide du CSC		
Activité 11	Plan pour les matériels et les fournitures		

Rapport de l'Activité 1: Développer une vision de votre CSC
Date :

Nom du CSC :

Slogan du CSC :

Code de conduite du CSC

**Rapport de l’Activité 2**<br>**Date :**	
**Questions**<br><br><br><br>Transmission de la malaria<br><br><br><br><br><br>Diagnostic de la malaria<br><br><br><br><br><br>Traitement de la malaria<br><br><br><br><br><br>Prévention de la malaria<br><br><br><br><br><br>Elimination de la malaria	**Réponses données par les membres du CSC (le cas échéant)**

<table>
<tr><td>Rapport de l’Activité 3
Date :</td><td></td></tr>
<tr><td colspan="2"></td></tr>
<tr><td colspan="2">Photo de l’affiche avec les facteurs de risque de la malaria</td></tr>
</table>

**Rapport de l'Activité 5**<br>**Date :**	
**Interventions possibles**	**Interventions choisies par le CSC**<br>(les écrire ci-dessous, ou marquer avec un X)
Visites domiciliaires	
Réunions communautaires	
Evènement communautaire	
Programme scolaire	
Annonces communautaires	
Aider les personnes qui présentent des symptômes de malaria à diagnostiquer et à traiter	
Travailler avec et assister les cliniques et hôpitaux (publics et privés)	
Travailler avec et aider les guérisseurs traditionnels	
Travailler avec et aider les agents de santé communautaire	
Promouvoir l'utilisation de moustiquaires et de fenêtres en tulle	
Identifier et monitorer les gîtes importants de moustiques	
S'engager dans le nettoyage de l'environnement	
Travailler avec les ONG et les clubs de santé	
Travailler avec les gouvernements locaux	
Travailler avec le MSPP	

**Rapport de l’Activité 6**<br>**Date :**	
**Nom du quartier choisi**	**Raison expliquant ce choix**

**Rapport de l’Activité 7**<br>**Date :**		
**Interventions choisies**	**Groupe social que vous voulez cibler**	**Raison pour laquelle cibler ce groupe social**

Rapport de l’Activité 8<br>Date :	
**Le CSC choisira-t-il l’Option 1 ou l’Option 2 ?**	
**Nom de chaque quartier choisi pour les prochains six (6) mois**	**Nom de chaque intervention choisie pour les prochains six (6) mois**

Activité 10 : Plan de Réponse Rapide CSC
Date :

Pour exécuter votre Plan de Réponse Rapide CSC, vous devrez décider des interventions que vous voulez réaliser pour répondre à un cas de malaria et créer un plan pour :

- Phase intensive des activités sur une période de deux (2) semaines visant à localiser d'autres cas de malaria ;
- Phase supplémentaire des activités sur une période de trois (3) mois.

Ecrire ceci ci-dessous :

**Interventions choisies pour la phase intensive des activités de réponse rapide**	**Interventions choisies pour la phase de trois (3) mois des activités de réponse rapide**

Une fois terminée cette période d'intervention, incorporer ce quartier aux quartiers-cible pour vos interventions routinières sur une période d'un (1) an

**Activité 11 : Planification des Matériels et des Ressources**<br>**Date :**<br><br>Ce plan devra être mis à jour sur une base régulière				
		Demande (date)	***Approbation? (O/N)***	***Réception (date)***
**1. Matériel pédagogique**				
	Brochures			
	Flyers			
	Affiches			
**2. Fournitures de planification**				
	Livres			
	Panneaux d'affichage			
	Plumes			
**3. Matériel de mise en œuvre (si disponible)**	T-shirts			
	Badges			
	Kits scolaires			
	Equipements de nettoyage			
**4. Autres ressources**				
**5. Equipements spéciaux de contrôle de maladie (demandés par le MSPP au niveau local)**				
**6. Ressources communautaires et contributions**				
