# Supplementary material for: Evaluating a community engagement model for malaria elimination in Haiti: lessons from the community health council project (2019–2021)
Source: Malar J. 2023 Feb 9;22:47. doi: 10.1186/s12936-023-04471-z (PMC9910254; doi:10.1186/s12936-023-04471-z)
Supplement: Supplementary file 2 — Additional file 2. CHC M&E handbook English and French. [file 12936_2023_4471_MOESM2_ESM.zip › New folder (2)/CHC M&E Handbook_english.pdf]

2020

Community Health Councils in Haiti: A Monitoring, Evaluation and Learning Handbook

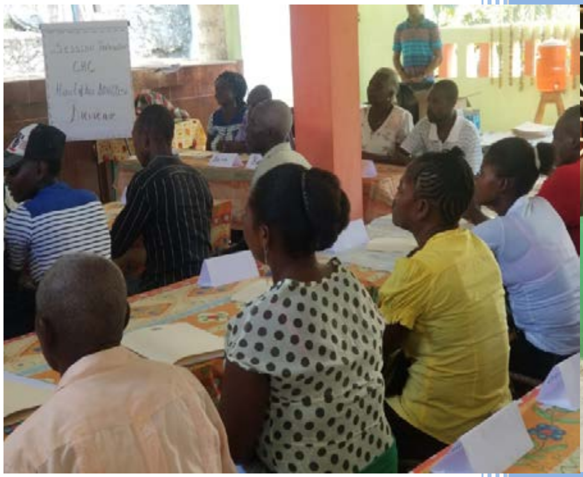

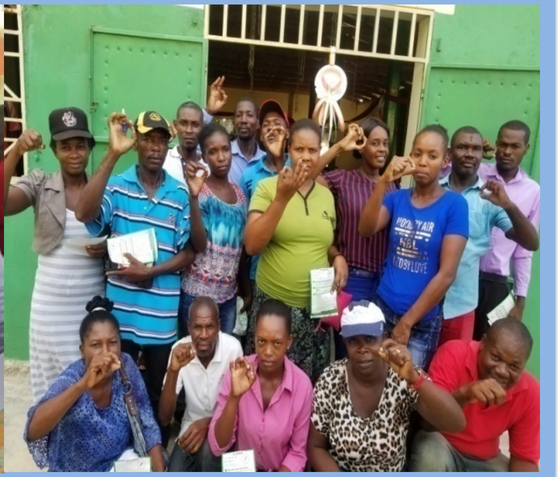

This handbook was developed to provide step-by-step guidance to staff from the Ministère de la Santé Publique et de la Population (MSPP) and other implementing partners who are directly involved in monitoring and evaluation (M&E) activities for malaria elimination. It provides detailed instructions for how to establish and run monitoring, evaluation and learning activities for Community Health Councils (CHCs). First developed in Grand Anse Department, CHCs are community action groups that aim to strengthen community health with the goal of eliminating malaria from Haiti. This handbook should be read alongside the CHC Implementation Manual.

Suggested citation:
Bardosh, K., Jean, L. Desir, L. Yoss, S., Nu, S., Poovey, B., Blount S. and Noland, G. (2020). *Community Health Councils in Haiti: A Monitoring, Evaluation and Learning Handbook.* The Carter Center: Atlanta.

Table of Contents

HOW TO USE THIS HANDBOOK 1

CHAPTER 1: WHAT IS COMMUNITY ENGAGEMENT AND WHY IS IT IMPORTANT? 2

CHAPTER 2: MONITORING, EVALUATION, AND LEARNING IN MALARIA PROGRAMS 5

CHAPTER 3: COMMUNITY HEALTH COUNCIL M&E FRAMEWORK 15

CHAPTER 4: THE CHC M&E TOOLBOX 20

INTRODUCTION 20
1) MONTHLY MONITORING REPORTS 21
2) SUPERVISION AND EVALUATION FORMS 23
3) RAPID QUALITATIVE ASSESSMENTS 24
4) COMMUNITY FEEDBACK 27
5) COMMUNITY-BASED KAP SURVEYS 29
6) ADAPTIVE LEARNING AND PARTICIPATORY ASSESSMENT WORKSHOPS 31

CHAPTER 5: THE CHC M&E WORKPLAN 33

CHAPTER 6: DATA ANALYSIS, REPORTING, AND PROGRAM LEARNING 38

APPENDIX 1: CHC ACTIVITY REPORTS 44

APPENDIX 2: SUPERVISOR FORMS 47

APPENDIX 3: MSPP AND IMPLEMENTING PARTNER ACTIVITY REPORT 53

APPENDIX 4: QUESTION BANK FOR THE RAPID QUALITATIVE ASSESSMENTS 54

APPENDIX 5: TIPS FOR QUALITATIVE INTERVIEWING 58

APPENDIX 6: RECORDING DATA IN THE RAPID QUALITATIVE ASSESSMENTS 60

APPENDIX 7: COMMUNITY FEEDBACK FORM 62

APPENDIX 8: QUESTIONS FOR A COMMUNITY-BASED KAP SURVEY 63

APPENDIX 9: ADAPTIVE LEARNING AND PARTICIPATORY ASSESSMENT WORKSHOP INSTRUCTIONS 66

How to use this handbook

This handbook is a step-by-step guide to monitoring, evaluation, and learning activities related to the use of community health councils (CHCs) for malaria elimination in Haiti.

It includes guidance for MSPP staff, CHC members, and other implementing partners in the planning, conducting, analyzing, reporting, and application of M&E data to improve the CHC initiative.

The handbook should be distributed to MSPP staff involved in CHC monitoring, evaluation, and learning activities. It should be read alongside the CHC Implementation Manual, which describes how to establish and operate CHCs.

The manual includes 6 Chapters and 9 Appendices.

Chapter 1 introduces why community engagement is important for malaria elimination.

Chapter 2 outlines the basic principles of monitoring, evaluation, and learning.

Chapter 3 describes the CHC program M&E framework including the logic model.

Chapter 4 provides step-by-step instructions for 6 M&E methodologies.

Chapter 5 discusses a number of key considerations for developing the M&E workplan.

Chapter 6 outlines key considerations for analysis, reporting, and learning.

The 9 Appendices provide necessary data collection forms, question banks, and detailed instructions for how to use the M&E tools outlined in this manual.

CHAPTER 1: What is community engagement and why is it important?

The global fight to eliminate malaria depends on the active participation of diverse groups of people. Studies have repeatedly shown that malaria interventions benefit from higher coverage and greater effectiveness when they involve local people in decision-making. This is true across the range of anti-malaria interventions – from case management to vector control to mass drug administration and surveillance. It is also true in areas where malaria transmission is low, as it currently is in much of Haiti.

Interventions not based on community engagement (CE) principles are more likely to encounter resistance, disengagement, or apathy at the community level; they also tend to overlook contextual details that are important to better planning and delivery. *CE can be defined as: the process of working collaboratively to achieve a set of common goals that improve health outcomes.* CE involves dialogue, trust, collaborative activities, feedback, and learning. There is no set "menu" for effective CE in malaria elimination; rather a set of principles should guide a process that heavily depends on listening to diverse voices and incorporating them into program plans.

When doing CE, public health officials face a paradox. If the program is too prescriptive, it risks encouraging top-down approaches that do not sufficiently involve community participation. In this case, we may say the program is "*participatory*" but, in reality, community and local voices are not really taken into account. On the other hand, all programs need pre-defined goals and objectives – especially those focused on one disease (in this case, the elimination of malaria) – and experts still need to provide guidance and protocols to ensure quality and accountability.

CE is a process that takes time. Relationships and trust need to be built at multiple stages of an effective CE plan. As with health promotion, the frequency of engagement activities is very important. People spend time on activities that they think are important, and determine their ideas of value based on a complex number of perceptions, beliefs, norms, and social realities. Considering that the risk of malaria in Haiti is relatively low,

it is not necessarily a major priority for local communities—particularly those in low transmission areas. In high transmission areas, its urgency is likely to decline as elimination is approached.

What are Community Health Councils (CHCs)?

A CHC is a voluntary organization run by local residents, focused on carrying out education and behavior change campaigns, assisting with community disease surveillance activities, helping to strengthen links between the community and medical professionals, and the mobilization of local residents in disease prevention. The primary goal of the CHC program is to empower individuals, families, and communities in Haiti to strengthen local action for health and social change in ways that directly assist in the elimination of malaria. The initial establishment of a CHC has a special emphasis on malaria prevention and control activities; although once CHCs are functional and have carried out activities for 6-12 months they are encouraged to engage in other health issues beyond malaria. The CHC approach was initially established in Grand Anse Department by The Carter Center, which leads community engagement for the Malaria Zero alliance[1], to support MSPP's malaria elimination efforts in Haiti.

Even in high transmission areas, malaria is only one of many problems people face, and it is usually not the most important one. Community enthusiasm and motivation for participation are particularly important, including intangible incentives such as recognition, the feeling of making a worthwhile contribution, and knowledge gained rather than by external financial incentives. CE may also require looking "outside the box" of malaria to also address other health delivery and disease problems simultaneously.

CE should work with and strengthen local capabilities, structures, and community leadership. This includes community members joining together to analyze the problem of

---

[1] Partners include the Ministry of Public Health and Population of Haiti, the Ministry of Public Health and Social Assistance of the Dominican Republic, the U.S. Centers for Disease Control and Prevention, the CDC Foundation, the Pan American Health Organization, The Carter Center, the Clinton Health Access Initiative, the London School of Hygiene & Tropical Medicine, and Tulane University School of Public Health and Tropical Medicine.

malaria transmission, prevention, and control and using that analysis to develop action plans to address them. They may also want to analyze other health problems and diseases as well. In this way, CE is a process of facilitating social and community change and addressing social norms, health delivery issues, and behavioral determinants. It also demands flexibility and adaptation.

For all of these reasons, monitoring, evaluation, and learning are very important parts of community engagement programs, including the Community Health Council (CHC) program in Haiti that is focused on malaria elimination.

This handbook should be read and used alongside the CHC Implementation Manual:

Bardosh, K., Jean, L. Desir, L. Yoss, S., Nu, S., Poovey, B., Blount, S., and Noland, G. (2020). *Community Health Councils (CHCs): An Implementation Manual for Community Engagement and Malaria Elimination in Haiti*. The Carter Center: Atlanta.

CHAPTER 2: Monitoring, Evaluation, and Learning in Malaria Programs

2.1. Monitoring and evaluation in the project cycle

Monitoring and evaluation (M&E) is a vital part of any effective health program. By tracking activities and community response, the M&E system facilitates accountability, learning, and adaptation. M&E helps to build the capacity of staff and community members in analyzing the delivery and social dimensions of the program. By doing so, M&E enhances problem solving and can be used to improve program quality and effectiveness when it is combined with learning activities that are integrated with the program planning process. Lastly, M&E data also plays an essential role in justifying resources and investments, and in helping plan for future activities and programs.

Community Engagement (CE) practice needs to be evidence-based. Frequently, health authorities conduct community activities and mobilize local groups in ways that are not as effective as they could be. Monitoring and Evaluating CE is important. The M&E activities described in this handbook will help malaria elimination staff and community members in Haiti better plan and implement anti-malaria interventions.

Figure 1: The Project Cycle

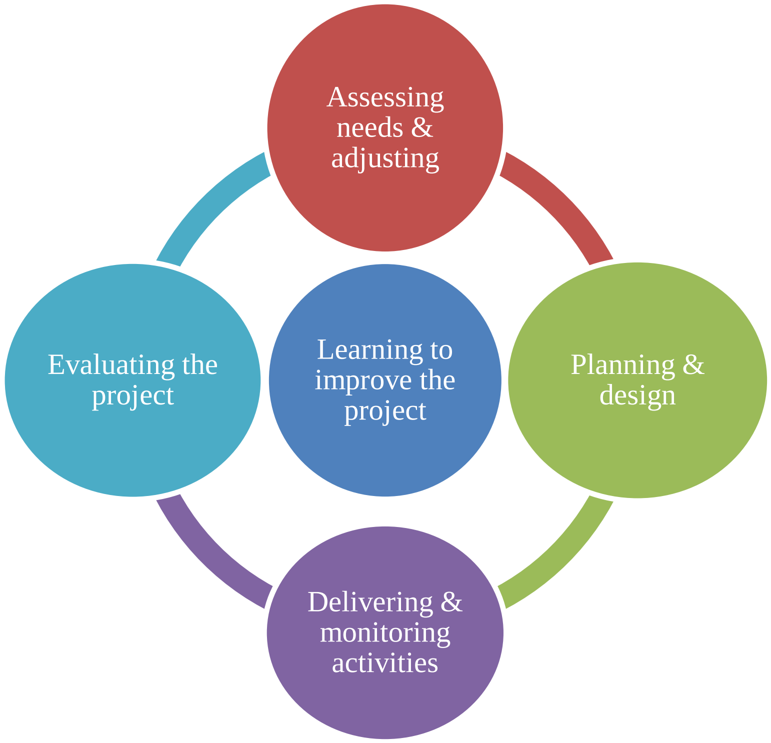

The feedback and learning generated by M&E data and analysis have the potential to dramatically improve health programs if M&E systems are designed wisely and thoughtfully, with sufficient resources. Unfortunately, in many programs M&E is treated as a secondary activity that is detached from planning and implementation; for example, focusing solely on quantitative data that summarizes how many people have been reached and how many activities have been preformed. This is an unfortunately limited view of the potential of M&E. The more expansive view is based on a shared commitment to collecting a wider range of data (quantitative and qualitative) and on using data and learning to inform planning and delivery. This process is illustrated above in **Figure 1.**

Monitoring and evaluation (M&E) are related activities. **Monitoring** is an ongoing activity that measures progress through routine data collection and helps inform real-time adjustments based on whether activities are being implemented faithfully and achieving their intended results. It involves collecting data to track progress in relation to the work-plan. The goal of monitoring is to correct and adjust – to make mid-course corrections and to fix problems as they arise. Monitoring is a form of feedback. In conducting monitoring, it is important to appreciate the importance of small implementation details and effects. Details matter. Monitoring allows the program to adjust the many details associated with implementation.

Evaluation is a more formal and comprehensive form of monitoring, typically done at midterm or at the end of a project. It is more rigorous and complex and focused on questions of effectiveness, relevance, and impact. Evaluation is used to make claims about the success of a program in meeting its desired outcomes, like knowledge and behavior change, or the reduction in malaria cases. The goal of evaluation is often to assess the effectiveness and value of an approach or policy and why it did or did not work. It is more programmatic and focused on documenting broader lessons learned, with a view of making claims about the overall approach that was taken.

M&E systems will allow a community engagement program to:

- **Improve activities** and plans by learning about what is working and what is not working
- **Improve the planning process** and the design of the program
- **Assess the results** of the program and whether it achieved its planned objectives
- **Determine the appropriateness** of program elements and activities
- **Make claims about effectiveness**
- **Demonstrate the value** of community engagement
- **Gain credibility** and support by showing results and impact

2.2. Introducing the logic model

M&E plans rely on a framework that outlines the ways in which a program is envisioned to work and how M&E activities should track and measure progress. This is typically outlined in a **logic model**, or logical framework, that articulates the logic of the project. A logic model is a management tool that helps clarify and review the activities and results of the project against its aims and objectives. The logic model also outlines the anticipated pathways and mechanisms for how activities will bring about change. See **Figure 2** here:

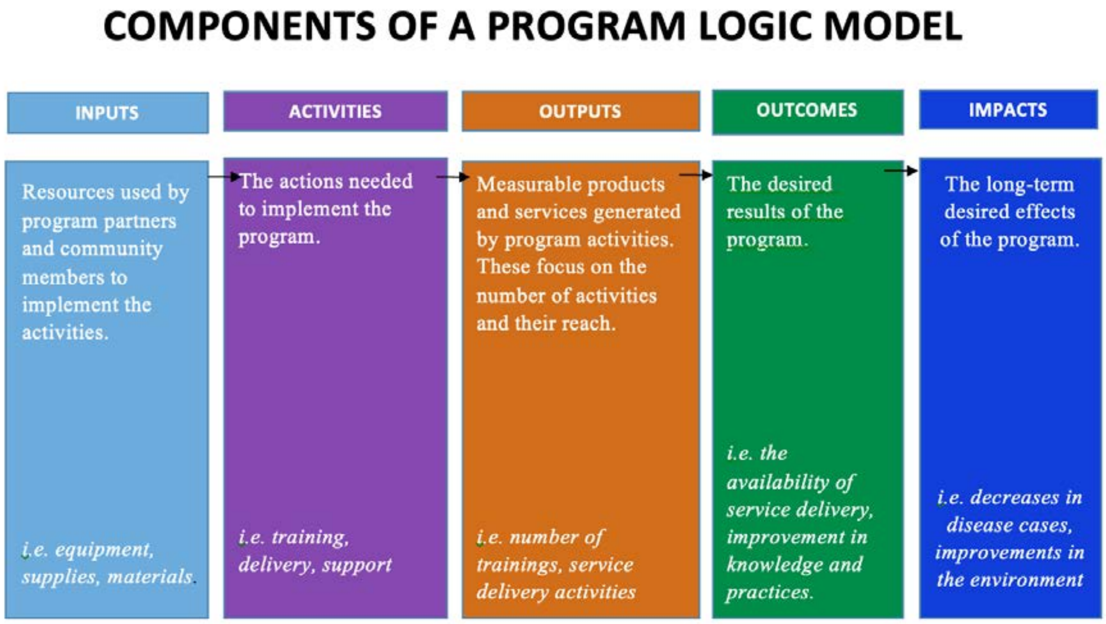

When designing an M&E system, it is important to ask:
"What do we need to know?" and "Who will use this information and for what purpose?"

2.3. Indicators

The inputs, outputs, activities, and outcomes described in logic models can be used as a template to develop **indicators**. Indicators define the focus of M&E data collection and analysis. It is the means by which success will be measured. While some indicators can be relatively straightforward and simple to measure, others are much more complex and challenging. This is especially true in CE programs when we are also interested in monitoring the process of change.

Indicators act much like a marker on the side of a road showing the distance traveled since leaving a destination. An example is provided below related to the cooking of rice. Indicators help to track a variable over time and include short, medium, and long-term measures for outputs and outcomes (see **Figure 3**).

**Cooking Rice**	
**Activity**	**Indicator**
Fire ready	Flames
Water boils	Bubbles and steam coming from the water
Rice is cooked well	The grain is soft, and the taste is good

A good indicator should closely measure the intended change with great specificity; in reality a set of indicators are often established to triangulate a desired change from multiple angles. This provides greater accuracy. **In general, indicators should not be modified once M&E activities begin.** However, modifications can occur if program objectives and approaches are changed, but the risks/benefits need to be considered.

Indicators can include quantitative (numeral) and qualitative (descriptive) variables. Although **quantitative indicators** tend to be used more widely, they have important

shortcomings for understanding CE programs. Tracking the number of activities that are implemented (for example, meetings or the distribution of drugs or bed-nets) is important to understand the delivery of the program. But they provide very limited insights into the *quality* of implementation and the effects of the activities on community members. CE interventions depend on the "soft" skills of persuasion, dialogue, and collective action that are inaccessible to quantitative indicators. For this reason, it is important that M&E frameworks consider including **qualitative indicators** that track factors associated with the *process* and *context* of community engagement.

M&E in community engagement (CE) programs

There is no "gold standard" M&E standard that can be used in all CE programs. The evidence on CE does not clearly indicate what specific indicators are associated with quality of process or outcomes. In fact, despite the acceptance that CE adds value to health programs, including for malaria elimination, important gaps remain in the science of *doing* CE. The evidence on what CE actions and practices are most effective—and their timing, frequency, and intensity (dosing) — is very context-specific. This makes evaluating CE challenging. There are no conclusive, straightforward standards. Rather, each CE program needs to develop its own strategy to meet project goals based on contextual factors and needs.

Indicators are used to track **inputs**, **outputs,** and **outcomes**. Outputs relate to the activities, services, events, and products of the project. By contrast, outcomes refer to what difference is made. Outcomes are the direct results, benefits, advantages, or disadvantages for individuals, families, groups, communities, organizations, or systems. Most projects find it easy to record and describe what activities are accomplished and the number of people served. The more challenging aspect of M&E relates to the outcomes and impact of a project: *what difference has been made? What changes have occurred due to the project?* These questions are tracked through outcome indicators. See **Figure 2** above for a definition of common M&E terms that we will use in the following chapters.

2.4. M&E in the project cycle

There are a few distinct M&E steps in the cycle of a typical program (**Figure 3**). The first involves a **formative assessment** phase (also known as a *needs assessment* or sometimes

simply called *exploratory research*). The formative evaluation phase is used to design and plan the project, as well as to review the program aims, objectives, and methodology prior to, or just after, the implementation of the project. For example, it can include qualitative research (focus group discussions, semi-structured interviews) on:

- Local perceptions and experiences of malaria;
- Sociocultural norms and practices relevant to malaria treatment, prevention, and control;
- The types of community-based organizations and groups operating in the area and their strengths and weaknesses;
- Existing health education material and channels of health communication;
- The acceptability of past and current anti-malaria activities, and of future possible interventions.

A formative evaluation allows staff an opportunity to explore perceptions, test the acceptability of the proposed intervention plans, and invite local community members to assist in defining priorities and activities. It can include research done before any activities have been implemented or it can be part of a small pilot project phase where some project activities are implemented and evaluated – for example, pre-testing malaria education poster or radio announcement with community members.

Figure 3: The steps involved in a typical M&E cycle

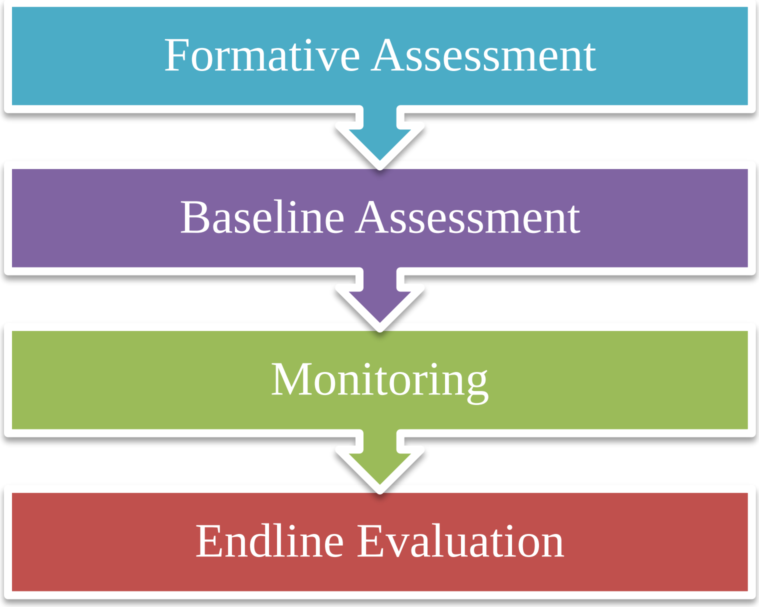

A second component of the M&E cycle is a **baseline assessment**. This should emerge from the formative research phase but may require additional data collection focused on a refined and focused set of outcome indicators that the CE intervention has selected to monitor over time. By providing a *before* and *after* picture grounded in data, a baseline assessment allows a program to make robust causative claims about change over time. Outcome indicators may include things like:

- Beneficiary knowledge
- Perceived risk
- Norms and attitudes
- Self-efficacy
- Behaviors and practices
- Epidemiological data
- Geographical distribution of services or environmental risk factors

Where a baseline evaluation is not possible or available, there are a number of research strategies that can provide insights into program-related changes. For example, beneficiaries and research participants can be asked:

1. <u>Retrospective questions:</u> what did you do or think before the project?
2. <u>Hypothetical questions:</u> if the project did not exist, what would be different right now?
3. <u>Change-based questions:</u> what has changed since the project? What has changed because of the project?

Similarly, if the ultimate goal of the evaluation is to measure the implementation process itself, robust baseline data may not be as important as when the aim is to strictly measure behavior changes before and after an intervention.

Baseline assessments usually focus heavily on data that are easily measured and quantifiable (but should, ideally, also make use of qualitative data). The baseline assessment should be revisited, using the same methodology, in the **endline evaluation**. The endline evaluation (often simply called "evaluation") refers to the final phase of data collection, where the inputs, indicators, outputs, and outcomes are examined in relation to the origin targets, and the successes and shortcomings of the program are determined.

During the program, as interventions are ongoing, program staff should focus on *monitoring* various indicators alongside program activities. **Monitoring** refers to the tracking of indicators between the baseline and endline evaluations. This typically includes quantitative data on the number of meetings, materials produced, populations reached, and people trained (quantitative data); but it should also include qualitative data on the process of implementation (what is going well and what challenges are being encountered?) and the acceptability, reactions, and level of participation and engagement of project beneficiaries and program staff to the implemented activities.

In order for M&E to have the most impact on community engagement (CE) programs, it is important to include participatory M&E methods and pay close attention to how data collection and analysis will be used, and by whom. A good way to think about this is to focus M&E on **social learning**: learning about a social context as you are actively engaged in that context. The *quality* of social learning is among the most important factors of making CE work and in using M&E data to improve implementation. Learning must take place at multiple levels as the program develops relationships of mutual trust with community members. Part of this should involve having community members collect and interpret data. It should also involve program staff providing data *back* to community members (for example, malaria epidemiological reports) in order to facilitate learning at the grassroots level. Data collection and the use of data should be a participatory process and not only a "top-down" exercise.

Learning should not only be confined to the "experts" who collect data in the field, analyze it in their offices, and distribute it back to the community. Communities need to be involved in the planning, collection, analysis, and reporting of M&E data.

A social learning approach is an important part of building a trust-based partnership where opportunities are found to iteratively adapt plans based on feedback from community members and field teams. This requires: open-mindedness, inclusivity, dialogue, transparency, and mutual respect. Knowledge is power and sharing knowledge increases trust and ownership of the activities.

Participatory monitoring is part of the social learning model and is an increasingly popular methodology in CE programs. This occurs during implementation, and is a key area for promoting community leadership, problem analysis, and social mobilization. It makes use of local skills and resources and facilitates group analysis. It also forces program staff to examine their assumptions about what progress looks like and gain a stronger appreciation of how local community members view the program aims and objectives. The program can use this information to adapt and modify its strategies and plans. This may include methods such as:

1. Participatory workshops
2. Self-evaluations and ranking exercises
3. Storytelling
4. Interpretation panels
5. Transect walks, focus groups and semi-structured interviews
6. Community and social mapping
7. Timeline and trend analysis
8. Seasonal calendars
9. Diaries and systematic narrative recording

This type of social learning model requires **a different set of skills** for M&E because it involves a facilitation process directed towards social change. This is challenging, as it requires a different set of "diplomatic" skills and expertise than is commonly available to public health and biomedical research teams. There are many **barriers to social learning** in a community engagement program. Although these are often blamed on a lack of resources, staff capacities, and/or political will, this is not always the case; poorly designed and implemented M&E systems are also a common driver. Typical weaknesses with M&E include: inappropriate methodologies, low-quality and inconsistently collected data, slow and inefficient analysis of the data, unsuitable dissemination and presentation of results, and a cultural reticence of using data to facilitate adaptation and change. A dedicated and rigorous M&E plan and field team, with a clear Standard Operating Procedure (SOP), is one of our best remedies for this.

A checklist for good M&E practice should include:

- ✓ Serve programmatic purposes, integrated with planning and implementation phases.
- ✓ Be participatory, involving program staff and community members in appropriate ways.
- ✓ Have specific, measurable, appropriate, and realistic methods and indicators.
- ✓ Be focused on generating new practical knowledge.
- ✓ Be accessible and understandable.
- ✓ Help empower project beneficiaries.
- ✓ Be adequately resourced.
- ✓ Consider the sociocultural and political context of the program.
- ✓ Address the theoretical assumptions of the project.
- ✓ Have an appropriate ethical framework in place, to protect confidentiality of individuals and safeguard against abuse or coercion.
- ✓ Be disseminated to community members and other stakeholders.
- ✓ Explore the weaknesses as well as the strengths of the project.
- ✓ Provide high-quality evidence for decision-makers and funders.

CHAPTER 3: Community Health Council M&E Framework

Introduction

Project M&E activities cannot measure everything. It is important to think carefully about what needs to be measured given the available budget and time restrictions of the program. To do so, M&E plans should rely on a M&E framework, which lists all the indicators for a program, data sources, how often indicators will be measured, and who is responsible for measuring them. This framework is used to guide M&E data collection, analysis, and reporting. It is important that you become very familiar with the M&E framework and the various indicators discussed in this manual. They form the core of the M&E plan.

Every M&E framework should be developed based on a program **theory of change** and/or **logic model**. A theory of change describes the *big picture* for how a given set of interventions is expected to lead to long-term change. It should capture the complexity of change within the wider social, cultural, economic, political, and environmental context of the program. In this way, it shows the different pathways to change, even if they are not related to the project. It asks: *"if we do X, then Y will change because…."* A theory of change is often presented as a flexible diagram with descriptive text. The CHC theory of change framework is outline in **Figure 4.**

Figure 4: CHC Theory of Change Framework

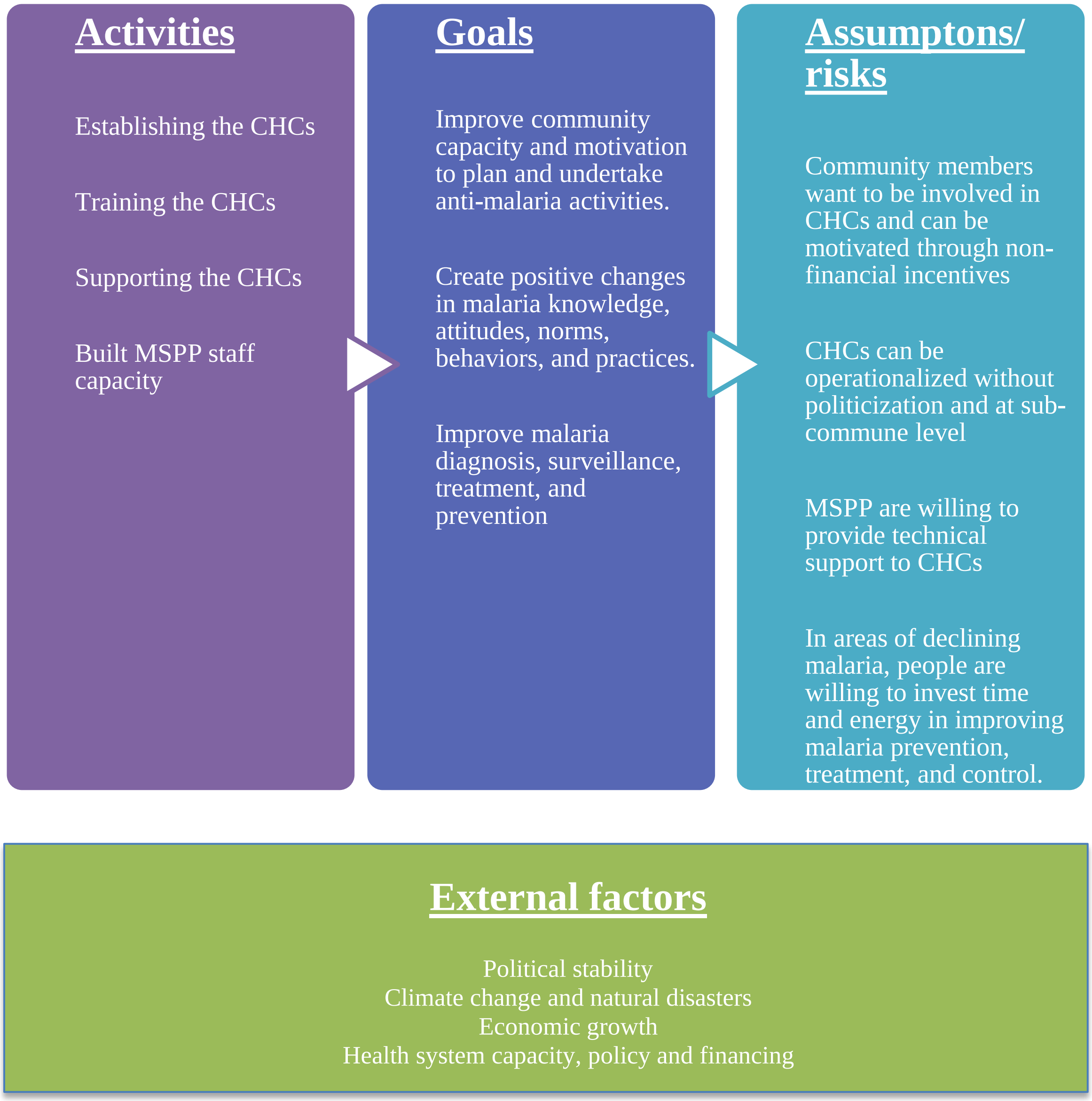

In contrast, the logic model illustrates how the change process will occur, from the programmatic (implementation) level. The logic model focuses only on the direct program components (inputs, activities, outputs, outcomes, and impacts) that are connected to change. The logic model for the CHC program is outlined in **Figure 5.**

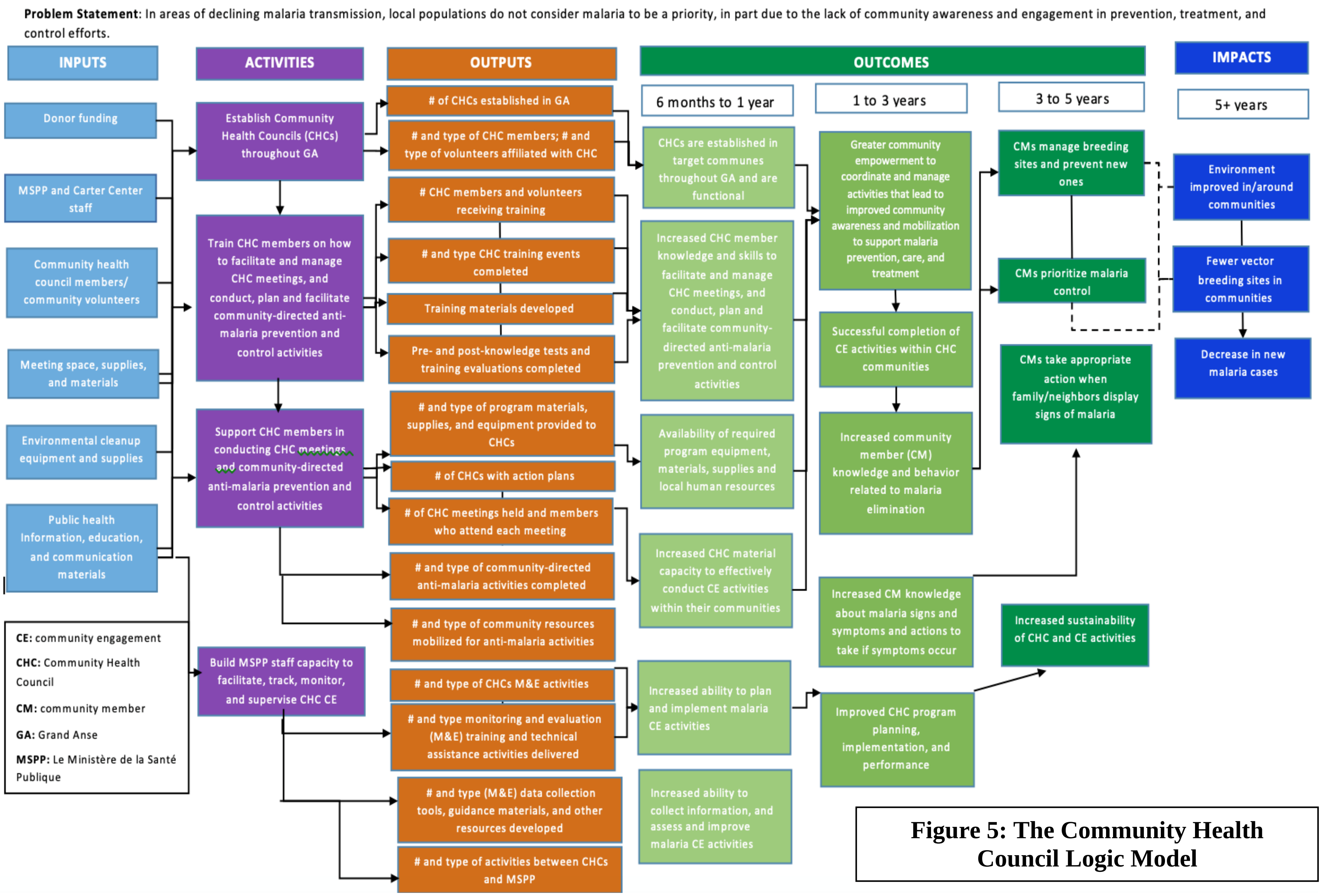

Figure 5: The Community Health Council Logic Model

The first part of the CHC logic model involves the **inputs** and **activities** of the program (see **Figure 6**). The activities of the CHC program involve:

1) ***Establishing*** the CHCs.

2) ***Training*** CHC members on how to plan, conduct, and facilitate CHC meetings and community-directed anti-malaria prevention and control activities in the various communities within their sub-commune.

3) ***Supporting*** CHC members in conducting CHC meetings and in community-directed anti-malaria prevention and control activities in the various communities within their sub-commune.

4) ***Building*** MSPP staff capacity to facilitate, track, monitor, and supervise CHC CE activities.

Figure 6: Inputs and activities of the CHC Logic Model

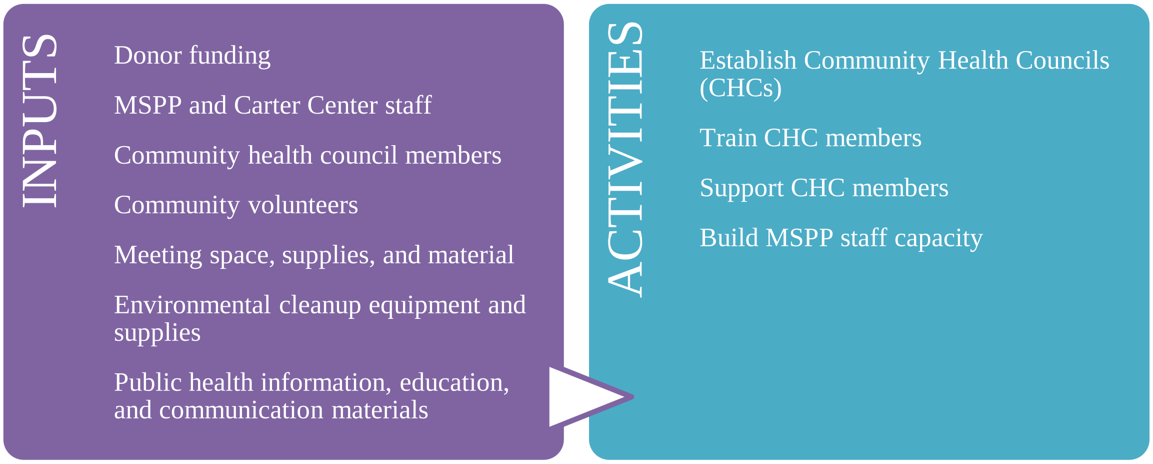

For each of the four activities listed above, there is a corresponding set of output and outcome indicators. In the table below we outline appropriate quantitative output indicators and list a set of outcome indicators. Depending on the methods that are used in the M&E activities, the outcome indicators can be assessed quantitatively and/or qualitatively. For example, the outcome indicator 3A (*Increased awareness about the importance of malaria control and prevention*) can be assessed either as a percentage increase (%) over time in a baseline and endline survey or through the use of qualitative data.

The CHC Activities: Key Program Indicators

Activity	Output indicators	Outcome indicators
1.0 Establish CHCs throughout GA	1.1. Number (#) of CHCs established against target<br>1.2. Number (#) of CHC members who participate in CE activities against target<br>1.3. Number (#) of community volunteers affiliated with the CHCs	1.a. Presence of functional CHCs throughout GA during program reporting period, with active volunteer network
2.0 Train CHC members on how to manage CHC meetings, and conduct, plan and facilitate community-directed anti-malaria prevention and control activities	2.1. Number (#) and type of CHC members and volunteers trained against target<br>2.2. Number (#) and type of CHC training events conducted against target<br>2.3. Training materials developed<br>2.4. Pre/post knowledge tests and training evaluations	2.a. CHC members with demonstrated improved knowledge post-training activities
3.0 Support CHC members in conducting CHC meetings, and community-directed anti-malaria prevention and control activities	3.1. Number (#) and type of materials, supplies, and equipment distributed to CHCs against target<br>3.2. Number (#) of CHCs with action plans<br>3.3. Number (#) of CHC meetings held annually, and % of members in attendance<br>3.4. Number (#) and type of community-directed anti-malaria activities completed against target<br>3.5. Number (#) and type of community resources mobilized for anti-malaria activities<br>3.6. Number (#) of health education materials distributed to CMs against target<br>3.7. Number (#) of CMs reached through anti-malaria activities, divided by type of activities	3.a. Increased awareness about the importance of malaria control and prevention<br>3.b. Increased awareness about malaria signs and symptoms and actions to take if symptoms occur<br>3.c. Positive opinions and experiences of the CHCs<br>3.d. Changes in behavior related to malaria risk, prevention and treatment<br>3.e. CHCs stories of change
4.0 Build MSPP staff capacity to track, monitor, and supervise CHC CE activities	4.1. Number (#) and type of CHC M&E activities<br>4.2. Number (#) and type of M&E guidance, tools and resources<br>4.3. Number (#) and type of M&E training and technical assistance activities completed against target<br>4.4. Number (#) and type of joint MSPP-CHC activities completed, divided by type	4.a. Improved capacity to track and report CE activities<br>4.b. Improved capacity to monitor and supervise CHC CE activities<br>4.c. Evidence of self-sustaining CHCs<br>4.d. Improved malaria KAP, as a result of CHC CE efforts

CHAPTER 4: The CHC M&E toolbox

Introduction

M&E is a form of applied research, and a fine balance needs to be obtained between rigor and practical usefulness in terms of design and methods. It is important that M&E activities are *fit-for-purpose*, meaning that they collect the minimum amount of information needed to generate meaningful knowledge about program goals and objectives. M&E activities should still measure unanticipated outcomes (things that were not anticipated to happen) and account for external factors. But if M&E becomes too academic and research-orientated, it can quickly become overly complicated, expensive and time-consuming. It is important to ask: *what insights will the M&E data create? Who can use these data? How will data improve the program?*

It is important that M&E includes the **triangulation** of different data collection methods. By including a diverse number of methods, we can approach problems from different angles and generate more meaningful, accurate knowledge. It is this diversity of techniques and methods that will make M&E a powerful tool for program decision-making and change. The methods need to focus on the most important information needed to evaluate project outputs and outcomes. They need to be realistic and consider time and financial and human resources.

The CE M&E plan will involve a set of methods and data collection tools to track the output and outcome indicators in the M&E framework. The data collection tools are listed below in the **M&E Tool Matrix**. The sampling framework and sampling strategies are discussed in each sub-section below where an SOP for each tool is also provided.

Figure 7: Monitoring and Evaluation Tool Matrix

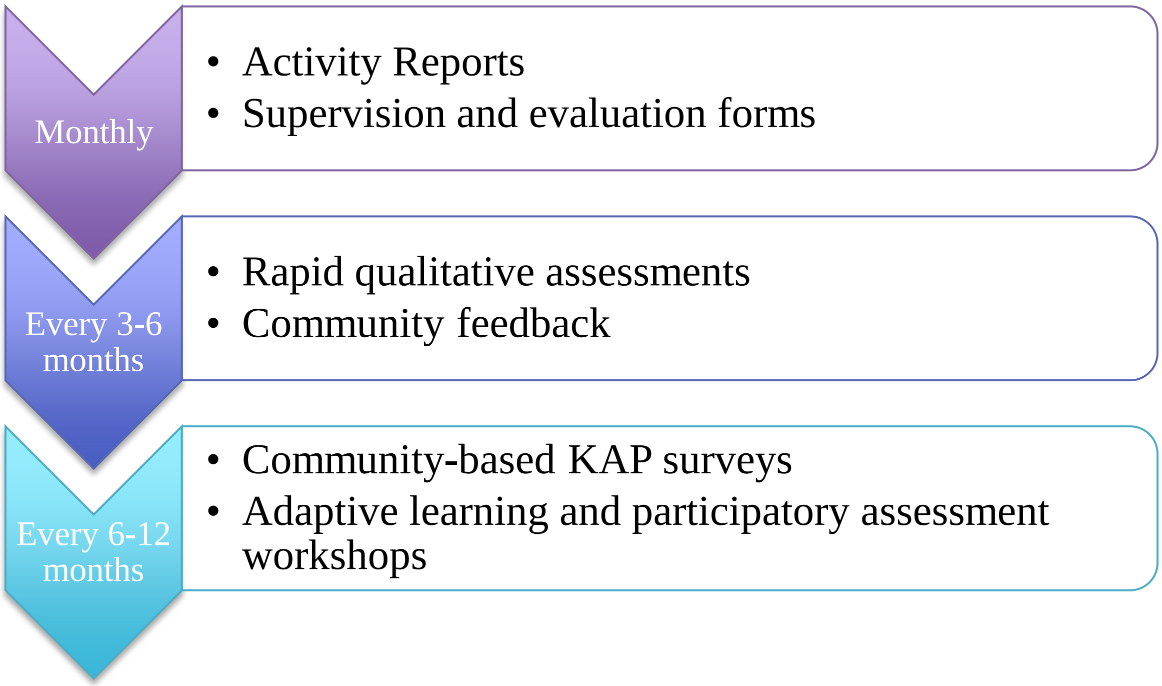

1) Monthly monitoring reports

The monthly monitoring reports will form the basis of the quantitative M&E data that will be collected and analyzed by the CHC program. This will include 1) CHC Activity Reports; and 2) MSPP/ Implementing Partner Activity Report.

Data recording should use the activity report forms in the appendix and can include:

- Retrieving hand-written reports filled in by the CHCs;
- Phone-calls with CHC members where the M&E team asks each CHC for the information on the Activity Reports;
- Electronic data collection via smartphones.

<u>CHC Activity Reports</u>

CHC Secretaries will submit CHC meeting data and community activity data to the M&E staff on a monthly basis. These will be submitted by mobile phone. There are two options for how to collect this data: 1) Submission via a smartphone to an online platform (such

as NEMO); 2) Verbal data collection via mobile phone, where the M&E staff will contact each CHC by mobile phone to verbally collect the information required for the reports. A combination of these two options may be required.

An M&E Officer will be responsible for collating and reviewing the data in order to provide a monthly summary to MSPP and CHC Program Managers.

During high-impact interventions (i.e. MDA, net distribution, IRS), the M&E team should be conducting rapid M&E activities and communicating findings on a daily and weekly basis to the MSPP implementation teams. This should be done by phone, a WhatsApp group, and email, and should include rapid summary of findings and recommendations.

The monthly CHC monitoring reports will include forms provided in the **Appendix 1**.

The CHC Activity Report will collect data on the following output indicators:

1.1. Number (#) of CHCs established against the target
1.2. Number (#) of CHC members who participate in CE activities against target
1.3. Number (#) of community volunteers affiliated with the CHCs

3.3. Number (#) of CHC meetings held annually, and % of members in attendance
3.4. Number (#) and type of community-directed anti-malaria activities completed against target
3.5. Number (#) and type of community resources mobilized for anti-malaria activities
3.6. Number (#) of health education materials distributed to CMs against target
3.7. Number (#) of CMs reached through anti-malaria activities, divided by type of activities

4.1. Number (#) and type of CHC M&E activities

<u>MSPP/ Implementing Partner Activity Report</u>

MSPP and Implementing partner staff involved in the CHC program at field level will submit a monthly activity report via email to MSPP and CHC Program Managers.

This will record the number and types of activities conducted, the challenges encountered, and the recommendations for future activities. The monthly MSPP/ Implementing Partner Activity Report will include forms provided in the **Appendix 2**.

As part of this monthly activity report, the CHC program will also collate malaria-specific data from MSPP. This will include the number of malaria cases and treatments by sub-commune location and the number of malaria rapid diagnostic tests (RDT) and microscopy tests performed, divided by +/-. These data are already collected as part of routine MSPP data collection. This data will allow program staff to plan for increases in malaria cases and, if needed, to activate the CHC rapid response plans.

The MSPP/ Implementing Partner Activity Report will collect data on the following output indicators:

2.1. Number (#) and type of CHC members and volunteers trained against target
2.2. Number (#) and type of CHC training events conducted against target
2.3. Training materials developed
2.4. Pre/post knowledge tests and training evaluations

3.1. Number (#) and type of materials, supplies, and equipment distributed to CHCs against target

4.2. Number (#) and type of M&E guidance, tools and resources
4.3. Number (#) and type of M&E training and technical assistance activities completed against target
4.4. Number (#) and type of joint MSPP-CHC activities completed, divided by type

2) Supervision and Evaluation Forms

As a tool to assist MSPP support staff in monitoring and supporting CHCs, two different supervision and evaluations forms should be part of routine M&E activities. These forms are provided in **Appendix 3** and should be used for program monitoring by MSPP staff. Data collection should be routine and the frequency and number of CHCs included in the monitoring framework should be determined by the available budget for routine monitoring activities. The key findings from the first form should be summarized in the Monthly MSPP/ Implementing Partner Activity Report.

The second form, which includes grades for each CHC, should be used at set intervals (for example, every 3-6 months) and used as the basis for the evaluation of CHC prizes. This data should be entered into an excel sheet for analysis.

3) Rapid Qualitative Assessments

Rapid qualitative assessments will form an important part of the M&E system that will be collected and analyzed by the CHC program. This will include qualitative semi-structured interviews, focus group discussions, and participant observations conducted by MSPP and implementing partner staff. It is important that staff who conduct the rapid qualitative assessments have appropriate training, skills, and prior experience in qualitative interviewing, data collection, and data analysis.

The rapid qualitative assessment should be conducted every 6 months to understand:

- CHC governance and management
- Strengths and weaknesses of anti-malaria field activities
- Community reactions and responses to CHC activities
- CHC motivation and incentives
- Capacity gaps and training needs
- The strengths and challenges of ongoing collaboration between CHCs and MSPP

The rapid qualitative assessment will collect data on all outcome indicators:

1.a. Presence of functional CHCs throughout GA during program reporting period, with active volunteer network
2.a. CHC members with demonstrated improved knowledge post-training activities

3.a. Increased awareness about the importance of malaria control and prevention
3.b. Increased awareness about malaria signs and symptoms and actions to take if symptoms occur
3.c. Positive opinions and experiences of the CHCs
3.d. Changes in behavior related to malaria risk, prevention and treatment
3.e. CHCs stories of change

4.a. Improved capacity to track and report CE activities
4.b. Improved capacity to monitor and supervise CHC CE activities
4.c. Evidence of self-sustaining CHCs
4.d. Improved malaria KAP, as a result of CHC CE efforts

Ideally, this should be done longitudinally through time in order to assess changes over time with the same CHCs.

A list of questions is provided in **Appendix 4.**

To create a **sample frame**, the M&E team should:

1) Make a list of all the CHCs;
2) Decide on the total number of CHCs that should be selected based on feasibility, staff capacity, and budget considerations;
3) Create a list of important 1) socio-cultural; 2) economic; 3) political and 4) geographical variations that are common to the total number of CHCs in the Department. This list should be as comprehensive as possible;
4) Make a list of key **output indicators** derived from the monthly CHC Activity Reports;
5) After completing the list, decide on the most important variables to consider for the sample frame. This should consider the variables that are most likely to influence the output and outcome indicators.
6) Group all of the CHCs according to the variable list. This grouping should produce a consensus of different "types" of CHCs that share common variables.
7) Select a certain number of CHCs to be followed longitudinally through time, based on this "common type" sample frame.
8) From this selected number, a smaller number of CHCs (3-5 CHCs per Department) should be selected for in-depth sentinel monitoring. In these sites, greater emphasis will be placed on collecting rapid assessment data directly from community members compared to the other selected CHC sites.

Therefore, the rapid qualitative assessment will include two different sample cohorts: a larger number of CHCs and a smaller number of CHCs to function as "in-depth sentinel monitoring sites." For example, we may want to include 10 CHCs in the Department of Grand Anse in the CHC sample frame and 4 of these CHCs in the in-depth sentinel monitoring. The only major difference between the two samples is that the in-depth sentinel sites should include qualitative research with a variety of community members,

conducted at the community level. During each rapid qualitative assessment, a sample frame for semi-structured interviews, focus groups, and participant observations should also be developed based on availability of staff and budget and logistical considerations. The methods and sample population of these two sample frames will consist of:

CHC sample frame	In-depth sentinel sites
Different CHC members in order to triangulate information;<br><br>Medical staff who have collaborated with the CHC, including MSPP support staff, community health workers, and local medics and nurses;	Different CHC members in order to triangulate information;<br><br>Medical staff who have collaborated with the CHC, including MSPP support staff, community health workers, and local medics and nurses;<br><br>A wide variety of different types of community members including community volunteers who work with CHC, community leaders and community-based organizations.

The exact number of interviews and focus groups will depend on the level of resources, time, and staff capacity available for the assessment.

For the **full CHC sample,** the assessment should ideally involve a field site visit to the sub-commune of each selected CHC. For ease of access, in some circumstances it may be necessary to convene select CHC members and MSPP staff at specific locations in order to conduct interviews and focus groups. In this circumstance, staff should consider a small reimbursement for travel costs and the provision of food/drinks. Additionally, it may be reasonable to conduct some, but not all, of these interviews through the use of phone-based interviews.

Activities for the **in-depth sentinel monitoring sites** should include visits to the locations where CHCs have targeted their activities in order to speak directly to

community members. Depending on feasibility, it is advisable to select at least 3 different locations to visit per sentinel monitoring site. Selection should account for socio-cultural and geographical variations common to the CHC catchment area. Some field visits should be planned to coincide with CHC meetings and outreach activities. This will help M&E staff hold interviews, focus groups, and observations with community volunteers, CHC members, community leaders, and MSPP supportive staff. However, it should be noted that this would increase the likelihood of *social desirability bias* (when people tell you what they think you want to hear, or emphasize positive things over criticisms, problems or negative perceptions and experiences). It is important that M&E staff are aware of the potential for social desirability bias and find ways to mitigate it by:

- Interviewing participants separately and not only in the presence of CHC members;
- Clearly explaining the purpose of the interview;
- Ensuring anonymity;
- Making participants feel comfortable by building rapport with them before asking difficult questions;
- Seeking clarifications and asking probing questions;

All interviews and focus groups should be both audio-recorded using a smartphone and conducted by two M&E staff; 1 staff member should be responsible for leading the discussion/interview while the second should take notes.

See **Appendix 5** for guidance on skills in qualitative interviewing and **Appendix 6** for guidance on data recording.

4) Community feedback

If the budget and staff capacity is available, it is advisable to establish a community feedback system as a complimentary part of the "rapid qualitative assessment." The goal of this community feedback system should be to facilitate adaptive learning in the field by following-up specific issues and problems identified in the rapid qualitative assessment. This should involve M&E and program staff from MSPP and implementing partners visiting the CHC at regular intervals, every 3 months, or as problems are

identified or additional support is required. During these visits, the community feedback team should visit with CHC members and then drive around the catchment area of the CHC with a few CHC representatives. During this "transect drive", M&E staff should take photographs, write down observations and reflections, and have conversations with CHC volunteers, community groups, and individual households. The focus of these conversations should be tailored to the particular issues and challenges that CHC is facing, as identified in the rapid qualitative assessment. After the transect drive, CHC program staff should convene a short meeting with as many CHC members as possible. During this meeting, program staff should:

1) Organize a problem-solving matrix using a flipchart with the following categories:

**Problems identified**		**Goal**	**Strategy**	**Tasks**	**Roles and responsibilities**
1)					
2)					
3)					
4)					

2) The list of major problems should be based on the results of the rapid qualitative assessment and/or monthly activity reports. These should have been agreed upon before the meeting.
3) Discuss the observations and findings of the community feedback obtained during the transect drive. If appropriate, add any additional problems or add greater details as appropriate.
4) Take field photographs and collect any photographs taken by CHC members of routine anti-malaria activities.
5) For each problem, CHC program staff should facilitate a group discussion in order to
    - Identify the goal;
    - Agree on the strategy to move from the problem to the goal;
    - Agree on the tasks required to achieve the strategy;
    - Agree on the roles and responsibilities of different individuals in each task.

6) At the end of the meeting, the CHC should keep the flipchart in order to assist with the implementation of the agreed activities. The M&E officer should take a photo of the flipchart and fill out the Community Feedback Form, provided in **Appendix 7.**
7) An excel spreadsheet should then be created to enter the data and to facilitate data analysis.

5) Community-based KAP surveys

Another important method that should be used as part of the quantitative M&E data collection are community-based knowledge, attitude, and practice (KAP) surveys. These should be organized every 6-12 months or can be organized at baseline and at the end of the project. These should be relatively short surveys that collect only the most essential household-level data on the outcome indicators listed below. Surveys are an important tool, if used appropriately, to explore changes over time in these key indicators.

A list of questions is provided in **Appendix 8.**

The community-based KAP survey will collect data on all outcome indicators:

3.a. Increased awareness about the importance of malaria control and prevention
3.b. Increased awareness about malaria signs and symptoms and actions to take if symptoms occur
3.c. Positive opinions and experiences of the CHCs
3.d. Changes in behavior related to malaria risk, prevention, and treatment

4.d. Improved malaria KAP, as a result of CHC CE efforts

If the budget and staff are available to conduct community-based KAP surveys, there are two options open to the M&E team.

1) *Involve CHC members directly in the survey data collection*. In this approach, MSPP and implementing partner staff should train CHC members and community volunteers in the design and use of a standardized survey instrument. This will allow CHCs to feel a sense of ownership over the results, although some form of remuneration and/or travel support should be considered and the use of CHC members may create social desirability bias in the results.
2) *Centrally deploy a team of data collectors from outside the CHC catchment area*. In this

approach, a trained team of data collectors is brought from outside the catchment area, which may reduce the possibility of social desirability bias but will increase the total cost of the survey.

Whatever approach you use, the field team will need to be trained on how to conduct the survey and the questionnaire will need to be pre-tested. This means that the questionnaire will be tested in the community with 5-10 participants and questions will be adapted if some questions are not clear or if additional questions should be added.

It is advisable to select the same CHCs for the community-based KAP survey as were selected for the rapid qualitative assessment; the sample strategy could include all selected CHC or only the in-depth sentinel monitoring sites. After an appropriate number of CHCs are selected, the sample strategy must also consider the number of locations to be sampled from within the catchment area of each CHC. At the local level, a final sampling consideration will involve the selection of participants. This can involve:

- Random sampling: Starting in the middle or outer periphery of a village and walking in a straight line, selecting every 5th household.
- If the survey is going to be repeated, it may be advisable to revisit the same participants in the future and repeat the survey longitudinally. In this case, a record of the survey participant should be maintained; for example, by marking their house location on a map, using GPS coordinates, and/or collecting phone numbers.
- A final option is to conduct surveys opportunistically at central points, such as markets, schools, and health centers. If this is done, it will be important to ensure that a representative sample of the population has been selected and that the sample has not been skewed to over-represent one specific social group.

If possible, M&E staff should consult a statistician who can advise on appropriate statistical sampling methods needed to generate robust statistical analysis on change over time in order to make causative claims. An excel spreadsheet should be created to enter the data and to facilitate data analysis.

6) Adaptive Learning and Participatory Assessment Workshops

As discussed at length in the CHC Implementation Manual, the CHC program must include strong participatory feedback mechanisms to ensure adaptive learning with CHC members and MSPP staff. To accomplish this, CHC program staff should organize formal workshops every 6-12 months. These will expand on the pedagogy used in the regular community feedback that, through the facilitated group discussion approach outlined above, aims to solve programmatic problems through group brainstorming and problem solving.

The number of CHCs, CHC, members and the frequency of these workshops will depend on the available budget and staff capacity. Ideally, they should be organized to correspond with the distribution of prizes, which is outlined in the Implementation Manual, and are an opportunity to evaluate and to adapt the Community Action Plan created by each CHC. Hence the objectives of these workshops can include:

- Promote adaptive learning;
- Provide a venue to distribute the CHC prizes;
- Evaluate and adapt Community Action Plans;
- Generate data for the formal monitoring and evaluation of the CHC program

Workshops can be organized with all CHCs and all CHC members or with a sub-set of CHCs (for example, those involved in the sentinel monitoring or community-based surveys). Workshops can also be organized by Department or by grouping several communes together. Travel and food costs should be considered for each delegate.

There should be at least 1 MSPP support staff for each CHC in attendance at the workshop. These support staff will help facilitate the group exercises and report back to the CHC M&E officer with appropriate notes and data.

In order to accomplish these objectives, the workshops will last 1-full day and be divided into 4 separate activities:

1) Program feedback to CHCs
2) Participatory assessment scoring
3) Generation of stories of change
4) Review of the Community Action Plan

The workshop will generate data on all of the outcome indicators listed above for the rapid qualitative assessment. In addition, it will be the only M&E techniques to provide data on outcome indicator *3.2: Number (#) of CHCs with action plans*.

Detailed instructions in how to facilitate the 1-day adaptive learning and participatory assessment workshop are provided in **Appendix 9**.

CHAPTER 5: The CHC M&E Workplan

Introduction

Implementing an effective M&E system requires developing and following an M&E workplan. The workplan should involve a number of major components, as shown in **Figure 7** below: 1) staffing, 2) schedules, 3) training, and 4) data collection. In this chapter, we discuss the important issues involved in each of these components and their role in CHC M&E activities. Details about data analysis and reporting are discussed in the next chapter.

Figure 8: Four components of the CHC M&E workplan

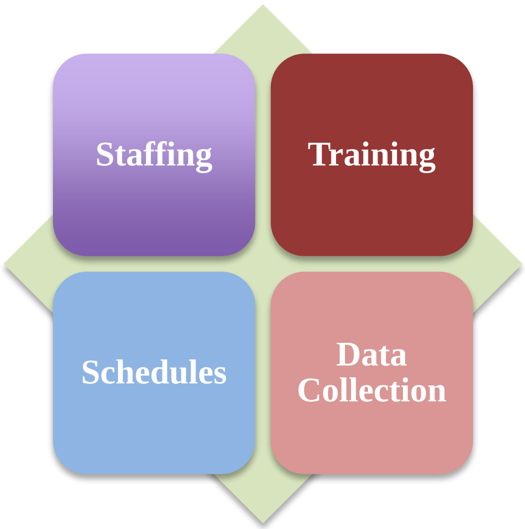

<u>Component 1: Staffing</u>

It is important to ensure that adequate staffing is provided to M&E activities. The exact composition of the M&E team will depend on the number and location of CHCs at any given time. In general, the staffing hierarchy should involve: 1) An M&E officer with a background in qualitative and quantitative research methods; 2) An M&E coordinator based in each Department; and 3) M&E assistants and field data collectors. These staff will work with the MSPP support staff in each commune and with each CHC, as described in the Implementation Manual.

Component 2: Training

Training in the CHC M&E plan should take place at multiple levels and should be repeated, as needed, on an annual basis.

First, an in-depth orientation needs to be provided to the M&E officer and M&E coordinators in all details of the CHC M&E handbook as well as a more extensive training in research methods, analysis, and reporting. Training in qualitative data analysis should be particularly emphasized.

Second, a detailed training workshop needs to take place with MSPP staff at the department and commune levels. This should be led by the M&E officer and should include detailed instructions for how MSPP support staff should train CHCs in the monthly Activity Reports.

Third, an orientation workshop should be organized for the CHCs and MSPP support staff. Two CHC members from each of the CHCs should be invited. Before this training, the CHC M&E officer should visit each CHC and discuss the CHC M&E plan.

Fourth, if the CHC program has sufficient resources, the M&E officer should develop a training plan for when and how to train field data collectors for the community-based KAP survey.

In all trainings, transport and per diems need to be included in the budget.

Component 3: Schedules

The activities of the CHC M&E team need to be carefully planned and this planning process will depend on the specific number and frequency of M&E data collection tools and the number of CHCs. The field schedule should be developed on a quarterly and annual basis but include monthly and weekly schedules.

The field schedule should include **four parts**, all with a strong emphasis on supervision and the clear division of labor. The first part should include training needs and plans; the second part should include a timeline for all field data collection activities; the third part should include a timeline for data analysis; and the fourth part should include a reporting schedule.

The **master field schedule** should be shared with appropriate partners and staff.

Figure 9: Four different schedules

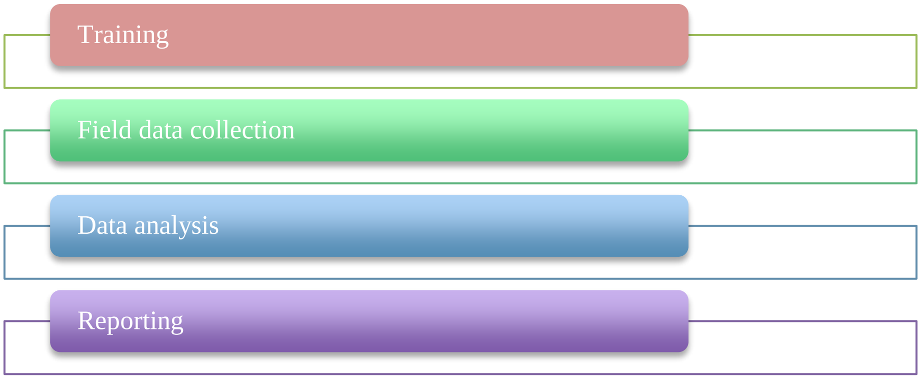

The quarterly and annual M&E workplan should describe the priority M&E activities and the roles and responsibilities of various individuals and groups. It should include the cost of each activity. And it should include a timeline for delivery of all outputs. The workplan is used for coordinating M&E activities and assessing progress of M&E implementation throughout the year. Plans should be adapted according to the field realities and needs of the CE program.

The budget should include the following elements:

- Staff salary costs
- Transport costs for field activities
- Transport costs for MSPP and field data collectors
- Infrastructure costs (computers, etc.))
- Supplies and materials
- Meeting venues, refreshments, and per diems
- Translation costs and printing
- Journal publication fees, if applicable

Component 4: Data collection

All data collection tools will need to be **field-tested**. An initial trial period needs to be planned, where modifications can be made to the tools themselves.

During data collection, M&E staff will need to follow acceptable **standards of research ethics**. This includes obtaining permission and informed consent. Standards and strategies for ethical engagement will be provided during the trainings. All data collectors will need to introduce the purpose of the data collection exercise to all participants and ask for consent. Because this is not a research study, but an M&E exercise, formal ethical approval, and formal consent may or may not be required – this needs to be confirmed. All data collection should be carried out with appropriate written approval from MSPP program managers.

Basic ethical standards for M&E data collection

Honesty: It is important that the M&E team does not lie or misrepresent things to community members.

Informed consent: People should, at all times, be told about the purpose of the data collection and be asked to participate. They should be informed that they can refuse, and that no consequences will occur if they refuse.

Confidentiality: All data collected from individuals must remain confidential and should not be disclosed publicly outside the M&E team, in a way that identifies a particular individual by name or in a way that their identity could be discerned. Pseudonyms should be used.

Cultural sensitivity: Data collection teams should abide by high standards of competence and professionalism and make every effort to communicate and behave in culturally sensitive and competent ways.

Reciprocity: Data collection teams should spend time answering participants' questions while in the field and should spent time providing feedback to community members on the results of the M&E data.

Lastly, the M&E team will need to think carefully about **recording** during the data collection process. The M&E team may want to use electronic data collection equipment

and software, hand-written notes, and/or audio recording. In each case, a short **SOP** should be developed for the recording strategy of each methodology.

CHAPTER 6: Data analysis, reporting, and program learning

Introduction

The M&E data cycle will involve a number of distinct steps, each of which will require attention. In most cases, these steps need to be repeated for each data collection method. The initial data collection phase is followed by four other steps; these include: **1) data entry and storage**; **2) data analysis**; **3) reporting**; and **4) program learning**. Tracking community engagement through a logical framework requires a systematic plan for data management, data analysis, and data use. Given the time, labor, and resources required to collect such data, it is imperative that the data is used for program learning and dissemination; otherwise, the M&E system is at risk of building excess capacity for information generation without an adequate mechanism for its practical use.

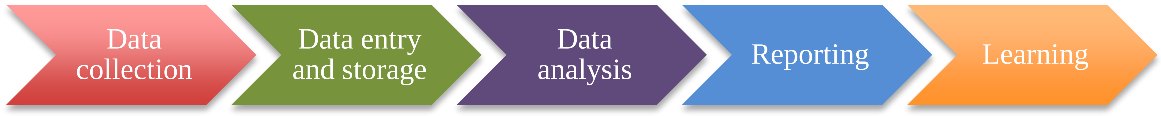

Figure 9: The M&E data process

Developing the M&E system will require unique skills and training to ensure rigor, accountability, and learning. Analysis of the Rapid Qualitative Assessments and the Adaptive Learning and Participatory Assessment Workshops will require social science skills not easily available within MSPP. Strengthening technical capacity for data analysis and reporting will be an important part of the overall contribution of CHC M&E activities.

Step 1: Data entry and storage

All data collection tools will require collection from the field and entry into a computer database. Data entry is a time-consuming task and adequate time and staff resources need to be devoted to it. Ideally, MSPP staff at the Department level can assist with most data entry, with support from the M&E team.

Data entry will involve developing multiple databases for each M&E methodology. A clear data quality check system should be developed, where every 20th line of quantitative data in Microsoft Excel is checked against the original data collection form for accuracy. Prior to analysis, the database needs to be cleaned. For qualitative data analysis, a software program (NVIVO/Atlas) may be used, or data can be analyzed using manual coding entered into Microsoft Word or Excel.

Data files should be saved in appropriately named folders with a code to allow easy access. The folder name should be the data collection tool (i.e. rapid qualitative assessment) and the file name should include the location and date.

All data should be uploaded to a shared cloud platform for easy access. The M&E officer should also backup all files on a daily basis onto an external hard-drive. Each week, the data from the external hard-drive should be saved onto a second external hard-drive that is stored under lock and key at the MSPP office. No one should have access to this second external hard-drive except the M&E officer and M&E assistant.

Four criteria for data quality

Accuracy – The data are accurate if they measure what they were intended to measure.

Timeliness – Data are timely when they are up to date and current and when the information is available and entered into the system on time.

Completeness – The data are complete when the results are complete – you must complete each tool in its entirety. Leaving data out weakens the outputs significantly and often means conclusions cannot be drawn.

Integrity – Data integrity is guaranteed when there are procedures or protocols that do not change according to the user or when or how the data is entered. This allows for consistent collection, measurement, and reporting of data. You need to be sure that you can trust the data entered.

During the data entry phase, the M&E officer should review the full dataset against accepted criteria for data quality and reflect on possible risks to bias and data quality that may have occurred. They should consider four criteria for data quality: accuracy, timeliness, completeness, and integrity.

Step 2: Data analysis

Data analysis involves both the analysis of individual data collection methods but also the collation of the various methods through triangulation. This M&E handbook has been designed to ensure that various forms of data are triangulated. Triangulation is where we use different data sources to approach the same issue in order to arrive at a richer, in-depth, and holistic understanding of social phenomena and program change. In this sense, the M&E officer will be tasked with comparing datasets to evaluate convergences and divergences.

The CHC M&E data analysis plan will involve analyzing qualitative, quantitative, and participatory data. MSPP staff at the Department level should preform the bulk of the data entry and analysis with support from the M&E officer and other staff. Training will need to be provided.

Step 3: M&E reporting

In general, there will be two types of reporting required for the M&E team. The first will include circulating the **Monthly MSPP/ Implementing Partner Activity Report.** This will occur on a monthly basis by email, from the M&E officer to the appropriate partners and MSPP staff. It may be advisable for an e-calendar to be established so that the team can review weekly schedules. Monthly calls should be arranged by the core M&E team to ensure that M&E activities are on track.

The second type of reporting will involve **quarterly M&E reports**. These should report all of the major activities undertaken that quarter, the results of all data collection, the implications for those results for the overall CE plan, and activities and the workplan for the next quarter. See a draft outline structure here:

Draft quarterly M&E report structure

Title page

- Narrative summary of progress (high point and low points) as compared to previous quarterly report.
- Describe any adjustments to workplan and justifications.

• **M&E Results**

- This is the main body of the report where you will present an update on successful and unsuccessful activities.
- Present progress against the targets you set at the beginning of the quarter.
- Present a summary of your M&E activities and present the key selected outputs.
- Under each graph note your interpretation for implementation.

• **Conclusions for Planning**

- Present conclusions drawn which led to the fulfillment of objectives.
- Present real difficulties and challenges, if any, and how you overcame them.

• **Next Steps**

- Explain how the CE team has used the information learned to adapt their work plan for the next quarter.
- Present your work plan for the next quarter.
- Present your M&E targets.

• **Links**

- Add links to photos.
- Add links to documents.

Step 4: Program learning

Arguably, learning is the most important part of the M&E process and should take place at multiple levels in the M&E system. Investing time and resources into developing and maintaining regular feedback loops, between the M&E team, MSPP, implementation partners, CHCs, and community members, will be key to facilitating this. Making M&E systems an effective mechanism for better program implementation will require regular communication between the M&E team and all other stakeholders. This will include formal, structured meetings and workshops, but also informal and casual conversations.

The goal of M&E data will be to initiate program learning at four levels.

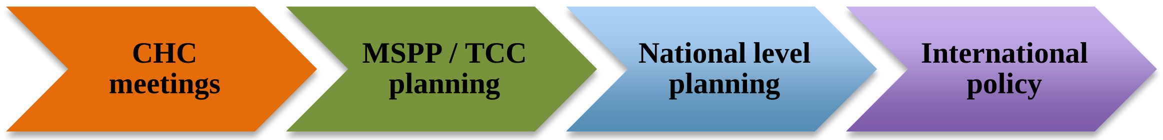

Figure 10: The four levels of program learning initiated by the CE M&E plan

Learning level 1: CHC meetings and action plans

As a community engagement program, it is of the utmost importance that M&E data are presented and shared with CHC members on a regular basis. This should occur as frequently as possible. Within each CHC, the CHC M&E officer will be tasked with presenting results back to the CHCs and, as appropriate, displaying this data in a public forum so that it is accessible to community members. Regularly sharing information about current malaria cases will be important for CHC planning and also will help foster a sense of shared responsibility and collaboration with MSPP. During routine support visits, MSPP staff should discuss previous data and ensure that action points have been addressed.

Learning level 2: MSPP/Carter Center planning at department level

The M&E officer will communicate regularly with the MSPP/partner implementation team. They will work collaboratively with these staff in developing the CHC work-plan and provide input into CHC strategies, their frequency, health message development, social mobilization approaches, incentive, and equipment inputs and training needs. They will provide regular updates on the results and implications of the M&E data. This should occur once per month.

Learning level 3: MSPP planning at national level

It is imperative that the M&E and CHC implementation teams present data back to the MSPP central office and other national partners. Ideally, this should include having MSPP staff from the central office directly involved in data collection, analysis and

reporting. Results should be shared through email updates on a regular basis and at appropriate national level meetings, preferably twice per year.

Learning level 4: International policy level

The goal of global malaria elimination continues to generate substantial political interest. The CHC model is a unique approach to the problem of engaging communities in malaria elimination, and there are very few rigorously evaluated community engagement approaches documented in the academic literature. Following the methodology outlined in this handbook, there should be an opportunity to present results at various international meetings and conferences, and in at least two academic research papers. Doing so will be important to building the evidence-base for community engagement in malaria elimination in Haiti but also in other countries. Staff should collate data into appropriate formats for high-level presentations and publications.

Appendix 1: CHC Activity Reports

Suivi de Réunion Bimensuelle

La date de la réunion __________________ (jj/mm/aaaa)

- Département Sanitaire ___________________ - Commune ______________________

- Section Communale ______________________ - Localité ________________________

- # de membres du conseil communautaire en total__________________

- # de membres présents à la réunion aujourd'hui ____________________

- Les noms et les rôles communautaires de tous les membres qui sont absents aujourd'hui:

Prénom	NOM	Rôle/Fonction/Titre

Résumez tout ce qui a été discuté à la réunion aujourd'hui:

Est-ce qu'il y a des problèmes ou des quéstions à rapporter?

Décrivez les quéstions / problémes :

Comment ces problèmes / questions ont-ils été résolus ou traités?

Résumez les décisions qui ont été prises à la réunion aujourd'hui:

Notez les points clés de la réunion aujourd'hui

Rapportage d’Activité de Sensibilisation

Date d'activité de sensibilisation ________________ (jj/mm/aaaa)

- Département Sanitaire _____________ - Commune ________________________

- Section Communale _______________ - Localité __________________________

- Lieu d’activité (une église, un marché, une église, une école, etc.) _____________________

- Les coordonnées GPS __________________________________

- Liste de membres du CHC qui ont participé:

Prénom	NOM	Rôle/Fonction/Titre

de personnes qui étaient touchées_________________

Choisissez l'activité de sensibilisation de la liste dessous. Cochez autant que nécessaires:

Faire de l'amélioration de l'environnement	
Sensibiliser la communauté sur l'aspect assainissement	
Efforts d'assainissement	
Sensibilisation au paludisme: réunions communautaires dans des installations publiques (école, église, etc.)	
Sensibilisation au paludisme: éduquer le public sur la transmission du paludisme	
Sensibilisation au paludisme: informer le public sur les mesures à prendre en cas de fièvre chez des parents ou des voisins	
Sensibilisation au paludisme: promouvoir le test et le traitement	
Sensibilisation au paludisme: informer le public sur le dépistage et le traitement gratuits	
Sensibilisation au paludisme: prévention du paludisme	

Détaillez l'activité de sensibilisation (par exemple: enlever l'eau stagnante):

Est-ce qu'il y a des problèmes ou des quéstions à rapporter?

Décrivez les quéstions / problémes.

Comment ces problèmes / questions ont-ils été résolus ou traités?

Appendix 2: Supervisor Forms

FORMULAIRE DE SUPERVISION ET D'EVALUATION COMITE DE SANTE

DATE: ………………………………………………………….

NOM DU COMITE: ……………………………………………………………………...

TYPE SUPERVISION : RENCONTRE ☐ **ACTIVITE** ☐ **EVALUATION** ☐

SUPERVISEUR NIVEAU : COMMUNAL ☐ **DEPARTEMENTAL** ☐

NOM/PRENOM SUPERVISEUR : 1.- ………………………………………………..

2.- ………………………………………………..

3.- ………………………………………………...

INFORMATIONS GENERALES

INFORMATION GENERALE SUR LE COMITE	
COMMUNE	
SECTION COMMUNAL	
LOCALITE	
LIEU	
POPULATION DE DESSERTE DU COMITE	
NOMBRE DE MEMBRES	

RENCONTRE MENSUELLE	
PRESENCE	# PRESENT ☐ # ABSENT ☐
LISTE MATERIELS DISPONIBLE ET CONNUE PAR TOUS LES MEMBRES	OUI ☐ NON ☐
VERIFICATION ENTREPOSAGE MATERIELS COMITE CHAQUE 3 MOIS	OUI ☐ NON ☐
ESPACE FIXE DE RENCONTRE MENSUELLE DISPONIBLE	OUI ☐ NON ☐<br>SI OUI……………………………………………………………………
SUJET PRINCIPAL DE LA RENCONTRE	……………………………………………………………<br>……………………………………………………………...<br>……………………………………………………………...
CAPACITE DU GROUPE	TRES BIEN ☐ BIEN ☐

A TRAITER LE SUJET A L'ORDRE DU JOUR	PASSABLE ☐ MAL ☐
	RECOMMANDATIONS :<br>1. …………………………………………………………… ……………………………………………………………<br>2. …………………………………………………………… ……………………………………………………………<br>3. …………………………………………………………… ……………………………………………………………
CALENDRIER DISPONIBLE	OUI ☐ NON ☐
PROBLEME DU COMITE	1. …………………………………………………………… ……………………………………………………………<br>2. …………………………………………………………… ……………………………………………………………
PROBLEME RENCONTRE PAR SUPERVISEUR	1. …………………………………………………………… ……………………………………………………………<br>2. …………………………………………………………… ……………………………………………………………
ACTIONS ENTREPRISES	1. …………………………………………………………… ……………………………………………………………<br>2. …………………………………………………………… ……………………………………………………………

**INITIATIVES DES SUPERVISEURS EN SUPPORT AU COMITE DE SANTE (PENDANT OU APRES)**
1.
2.
3.
4.

Signatures :

____

Coordonnateur du comité supervisé

Superviseur 1	Superviseur 2	Superviseur 3

FORMULAIRE DE SUPERVISION ET D'EVALUATION COMITE DE SANTE

DATE: ……………………………………………………………….

NOM DU COMITE: ………………………………………………………………………………...

TYPE SUPERVISION : RENCONTRE ☐ ACTIVITE ☐ EVALUATION ☐

SUPERVISEUR NIVEAU : COMMUNAL ☐ DEPARTEMENTAL ☐

NOM/PRENOM SUPERVISEURS : 1.- ………………………………………………………..

2.- ………………………………………………………..

3.- ………………………………………………………...

INFORMATIONS GENERALES

**INFORMATION GENERALE SUR LE COMITE**	
COMMUNE	
SECTION COMMUNAL	
LOCALITE	
LIEU	
POPULATION DE DESSERTE DU COMITE	
NOMBRE DE MEMBRES	

**GESTION ADMINISTRATIVE ET FINANCIERE**					
		**1**	**2**	**3**	**4**
	**Gestion matériels** : Compte rendu sur l'état des matériels chaque 3 mois.				
	**Inventaire du mobilier :** vérifier véracité dernier inventaire.				
	**Tenue de réunions chaque mois**: fréquence, procès-verbaux de réunions, suivi des décisions prises.				
	**Disponibilité des rapports de dépenses** : cahiers à jour, dépenses discutées et approuvées par les membres				
**SITUATION DU SIS**					
		**1**	**2**	**3**	**4**
	**Tenue cahier de rapport :** existence source primaire de données, propre, Cahier propre, cahier ordonné, etc. …				
	**Disponibilité calendrier d'activité :** existence calendrier mensuel, trimestriel, semestriel…				
	**Rapports mensuels :** à jour dans le cahier, vérification dernier rapport envoyé, rapports archivés…				
	**Situation des archives :** activités inscrites dans le calendrier, enregistrement de toutes les activités au niveau d'un cahier				
	**Existence d'un coin de discussion sur le rapport:** procès-verbal des rencontres mensuelles, …				
**GESTION DES RESSOURCES HUMAINES**					
		**1**	**2**	**3**	**4**
	**Administration**: connaissance des postes clés par les membres du comité, existence de moyens efficaces pour contacter tous les membres, liste téléphone contact des membres,				
	**Gestion des présences** : liste des absents disponibles pour toutes les interventions, discussions sur les absences dans au moins un procès-verbal des trois dernier mois (SOS)…				

	**Conditions de travail :** membre motivé à travers les ressources disponibles, équité, enthousiastes, …				
	**Respect mutuel :** Gestion de conflits, règle de fonctionnement du comité partagé				
	**Jour de célébration des résultats :** Présentation des résultats du comité aux leaders, petite séance récréative				
**GESTION DES RENCONTRES MENSUELLES**					
		**1**	**2**	**3**	**4**
	**Tenue rencontre mensuelle :** date fixe de rencontre, respect des dates au cours des 3 derniers mois…				
	**Procès-verbal des rencontres :** rapport de rencontre enregistré				
**GESTION DES ACTIVITES**					
		**1**	**2**	**3**	**4**
	**Evaluation des problèmes:** Liste des problèmes dans les différentes localités, intervention communautaire par ordre de priorité				
	**Efficacité des interventions** : Etablissement liste des causes des problèmes, arbre à problème, …				
	**Calendrier :** Calendrier en fonction des priorités, respect des calendriers (sauf non-respect documenté),				
	**Planification des activités :** Invitation, rencontre des leaders pour support, sensibilisation avant toute intervention d'assainissement				
	**Pérennité des interventions :** identification d'au moins deux actions durables menées par le comité, …				
	**Documentation correcte des interventions :** Description situation avant, description situation après, photos, vidéos (avant, pendant, après)				
	**Harmonisation /intégration :** recherche de partenariat avec d'autres intervenants				
**GESTION INTERRACTION INSTITUTION - COMMUNE -DEPARTEMENT**					
		**1**	**2**	**3**	**4**
	**Communication :** Communication avec les responsables (santé, mairie, …).				
	**Respect**				
**RECONNAISSANCE DU COMITE DANS LA COMMUNAUTE**					
		**1**	**2**	**3**	**4**
	**Comité connue :** connaissance existence du comité dans la zone (Ecole, Eglise, membre de la communauté)				
	**Rapports avec la communauté** : comite de gestion, réunion avec communauté, procès-verbaux des réunions, enregistrement des suggestions de la communauté				
	**Reconnaissance du comité :** Acceptation du comité dans la communauté,				
	**Inexistence de conflits :** neutralité du comité dans les interventions, non en conflit avec certains secteurs ou membres de la communauté				
	**Appréciation :** Communauté apprécie grandement les actions du comité				
	**Implication de la communauté** : Implication d'au moins 10 personnes de la communauté dans toutes les interventions d'assainissements, au moins 1 dans les activités de				

	sensibilisation comme autres intervenants				

Score final : ____________/___________ **Score Moyen / 100 :** _______________

RECAPITULATION DE LA VISITE

Points forts :

1- ____

2- ____

3- ____

4- ____

Points faibles :

1- ____

2- ____

3- ____

4- ____

Mesures prise sur place par les superviseurs :

1- ____

2- ____

3- ____

4- ____

Recommandations :

1- ____

2- ____

3- ____

4- ____

Signatures :

____

Responsable du comité supervisé

________________________ ________________________ ________________________

Superviseur 1 Superviseur 2 Superviseur 3

Appendix 3: MSPP and Implementing Partner Activity Report

This form should be filled out on a monthly basis and submitted by the CHC program manager, or equivalent, to the appropriate MSPP and implementing partner staff.

MSPP and Implementing Partner Activity Report

Month of reporting: ________________________________

Date of report: ____________________________________

*Staff member responsible for the report:*_________________

Describe the types of activities preformed, including the number of each and the location where they were preformed:

Describe the challenges you encountered and why you encountered them:

Describe recommendations for how the CHC program should address the challenges identified:

Suggest specific actions to follow-up, including who is responsible for them and the timeline for completion:

Malaria epidemiological data update:

Please ensure that you communicate with the Departmental MSPP malaria director and attach, to this monthly report, an epidemiological update that provides data on new malaria cases (including the location of cases), treatments, diagnostic tests (by RDT and microcopy) and other details, such as the implementation of interventions by the MSPP team.

Appendix 4: Question Bank for the Rapid Qualitative Assessments

The rapid qualitative assessment involves the use of semi-structured interviews and focus group discussions with CHC members, CHC volunteers, MSPP and other health staff, and a wide range of different community members and groups. Below is a question bank to assist M&E staff in their interviews and focus group discussions; this should be adapted based on the type of person being interviewed. The researcher may want to adjust the order of the questions, remove or add questions, and emphasize certain issues over others. For each question, researchers should probe with follow-up questions that expand upon *why* and *how* the participant thinks the way they do. Such probing is very important.

Section 1: Questions for CHC members and volunteers

1. *How have you been involved (roles and responsibilities) in the CHC?*
2. *In your experience, what are some of the positive things about the CHC?*
3. *In your experience, what are some of the weaknesses with the CHC?*
4. *What strengths have you seen with the management and coordination of the CHC?*
    - CHC meetings and coordination
    - Decision-making and planning
    - Involvement and coordination with MSPP
    - With monitoring and evaluation
5. *What weaknesses have you seen with the management and coordination of the CHC?*
    - CHC meetings and coordination
    - Decision-making and planning
    - Involvement and coordination with MSPP
    - With monitoring and evaluation
6. *How could the management and coordination of the CHC be improved?*
7. *What strengths have you seen with the activities and interventions of the CHC?*
    - Social mobilization
    - Environmental sanitation
    - Engagement with community groups
    - Education
8. *What weaknesses have you seen with the activities and interventions of the CHC?*
    - Social mobilization
    - Environmental sanitation
    - Engagement with community groups
    - Education
9. *How could activities and intervention of the CHC be improved?*

10. *What strengths and weaknesses have you seen with the capacity of the CHC?*
    - Do you think CHCs have sufficient motivation?
    - Do you think CHCs have sufficient knowledge and training
    - Do you think CHCs have sufficient guidance from MSPP?
11. *Does the CHC engage sufficiently with volunteers and other community members?*
12. *Has the CHC learned and adapted to challenges?*
13. *Do you think the CHC has had a major impact on malaria prevention and control?*
    - Why or why not?

Section 2: Questions for MSPP staff and medical workers

1. *How are you involved in malaria treatment and prevention?*
2. *How have you been involved (roles and responsibilities) in the CHC?*
3. *In your experience, what are some of the positive things about the CHC?*
    - In management and coordination
    - In activities and interventions
    - In motivation and capacity
4. *In your experience, what are some of the weaknesses with the CHC?*
    - In management and coordination
    - In activities and interventions
    - In motivation and capacity
5. *How have community members responded to the CHC?*
    - Positive things
    - Negative things
    - Areas for improvement
6. *Do you think the CHC has had a major impact on malaria prevention and control?*
    - Why or why not?
7. *How can MSPP better support the CHCs?*

Section 3: Questions for community members about the CHC

1. *Have you heard about the CHC?*
2. *What do you think about the activities of the CHC?*
3. *Have you participated in any CHC activities?*
    - Tell me about your experience.
    - What was good?
    - What was not good?
4. *What do most people think about the CHC?*
    - What good things do they say?
    - What not good things do they say?

5. *Do you think the CHC has helped reduce malaria?*
    - Why or why not?
6. *Do you think the CHC has increased people's knowledge of malaria?*
    - Why or why not?
7. *Do you think the CHC has had a positive impact on the prevention of malaria in your community?*
    - Why or why not?
8. *Do you think the CHC has had a positive impact on the diagnosis and treatment of malaria in your community?*
    - Why or why not?
9. *What problems or complaints about the CHC have you heard?*
10. *How do you think the CHC can be improved to be more effective and have more impact in your community?*
11. *Should malaria be more integrated with other health issues?*
    - What are the priority health issues for the population in your community?
    - How could these issues be better addressed through community groups and community action?
    - Can the CHC help? Should these issues be integrated with the CHCs?

Section 4: Questions for community members about malaria

1. *Do you think malaria is a problem in your community?*
2. *Can you tell me how malaria is spread?*
3. *What social factors are helping to spread malaria in your community?*
4. *When was the last time you heard messages about malaria?*
    a. Have you seen any malaria education material or heard any radio announcements in the last [INSERT] months?
        i. What did you think about them?
        ii. How could they have been improved?
    b. How could malaria information be better distributed in your community?
5. *Who do you consult if you suspect you might have malaria?*
6. *Do you have a community health worker in your neighborhood?*
    a. What do you consult them for?
    b. What good things do they do for people?
    c. What gaps exist in training or capacity for the community health workers?
7. *In the last [6-12] months, have you had a family member who suspected they might have malaria?*
    a. Can you tell me about the experience?
    b. Where did they seek help?
    c. Did they obtain malaria testing?
        i. If yes, did you pay for your malaria test?
        ii. Was it positive or negative?

d. Did they obtain any drugs for malaria?
e. How do you think the diagnosis and treatment of malaria could be improved in your neighborhood?

8. *Do you do anything in your household to prevent people from getting malaria?*
    a. Last night, did you sleep under a mosquito net?
        i. If not, why?
        ii. Did everyone sleep under a net?
        iii. Where did you get your nets?
    b. Do you work with your household members or other community members to address stagnant water sources?
        i. If yes, how?
        ii. How often?
9. *Do pregnant women or young children receive any special malaria prevention education or interventions in your neighborhood?*
10. *Do you think Hougans be more integrated with the malaria control effort?*
11. *Do you think it is possible to eliminate malaria from your Department?*
12. *What recommendations do you have for MSPP to improve malaria control in your community?*

If MSPP has implemented MDA or IRS in the area, additional questions on these interventions should be added to this list.

Appendix 5: Tips for qualitative interviewing

Good qualitative interviewing requires good conversation skills including:

- *Ask for consent:* Explain the project and reaffirm the person's informed consent.
- Keep key questions simple: *What? When? Who? Where? Why? How much/many?*
- Avoid leading questions
- Ask good "probes", also known as follow-up questions
- Focus on 1 to 3 major issues you want to discuss in any given interaction
- Build "rapport" to account for social desirability bias
- Use the language and categories of local people and avoid unnecessary biomedical terminology

Conduct a conversation with a purpose.

- Be flexible / conversational.
- Encourage anecdotes. Avoid too many generalizations.
- Don't control the conversation; accept pauses.
- Balance being both informal and formal socially.
- A few minutes of easygoing / casual chat here and there throughout the interview really helps.
- Follow the participants' clues.
- Be self-reflexive: pay attention to body language and what may be "hidden" or not disclosed by the participant in the interaction

Be familiar with the questions!

- Three types of questions: main question, follow-up questions and probes
- Don't look down at your notes and read questions: have a few specific reminders in the margins of your notebook
- Start the conversation off with light questions
- Move from the general/the societal to the personal/individual.
- Use local language and terms
- Avoid asking too many questions; rather, use indirect methods of conversational probing

Learn some strategies and be prepared

- Use hypothetical scenarios: "What would you do if…?"
- Ask for clarification, examples, and details
- Crosscheck: "I have heard…" or "I am finding that mostly men are…."
- Focus on personal stories
- Question strategy: "Please tell me about_____….tell me the whole story."
- Sometimes you want to ask the same question in different ways
- Ask dumb or naïve questions
- Make use of informal counting terms like "very few" / "some" / "most of the people"
- Make sure to differentiate between an individual's perceptions and practices about something and what they think others in the community think or do.
- Show "insider knowledge" to hint that you are ready for more detailed knowledge about a topic.

How should I take notes?

- Hold the book casually or have it in your pocket or bag. Don't hide your notes.
- Take out your notebook when the informant says something interesting.
    - Example: *"What you are saying is very interesting and important. I don't want to forget parts of it. I want to get it right. Do you mind if I take some notes while you are telling me these things?*
- Use your notes to clarify and ask questions

Appendix 6: Recording data in the rapid qualitative assessments

Recording should include hand-written notes and audio recording using smartphones. Each fieldworker should have two notebooks for: 1) for rough notes; reflective notes; and a methods summary; and 2) for expanded notes. *<u>Each field researcher should spend at least 1-1.5 hours each day completing reflective notes, expanded notes and the methods summary while they are in the field.</u>*

Rough notes

- We will primarily rely on hand-written "rough notes" using notebooks.
- Write down as many key words and phrases as you can while the informant is speaking.
- Retain the informants' exact words, as closely as you can. This is especially important for key phrases or creative sayings that the informant used to make an important point.
- For very important facts, you can ask: "wait a minute, this is very important for me to write down as you have just said it." Do this only for very important facts.
- You can read back your notes to the informant and ask them if you missed anything.
- After each discussion, or right before it ends, record (or ask) the general profile of these participant(s): gender, estimated age, social status/position.
- Immediately after the interview, go over the notes and add "remembered materials" that may have been missed while the informant was speaking (write up your field notes).

Reflective notes

- These notes are your personal diary of thoughts related to the fieldwork.
- You should note down important insights that you are gathering about the topic as well as hunches, hypotheses, and emerging follow-up questions you would like to bring up to the team in the group analysis.

Audio recording

- Audio recording should be used for key informant interviews and focus groups and only after consent is obtained from all participants. Audio recording should be used to add additional details to the notes when the field team creates "expanded notes"; the primary data recording method should be hand-written notes.

Expanded notes

- *<u>Once a day you need to expand your rough notes</u>*, moving from "rough notes" to half/full-sentence interview notes with descriptive details and observations.
    - Do not only give the main highlights; describe things in-depth.
    - Include some verbatim quotations (in quotation marks) and local language terms.

- Don't forget the details for each interview: Put the rough age and sex (and other relevant socio-demographic details, including social status/position) in parenthesis: [sex, age, other socio-demographic factors]
    - You will also grade each interview with a letter depending on the level of engagement shown by the participants and importance of the data: high (H), medium (M) or low (L). Use the letter H, M and L for this purpose.
- As you do this, reflect on how this new and interesting information fits into the other data you are collecting, and note any thoughts and follow-up questions in your *reflective notebook.*

Informed consent

- It is imperative that M&E staff introduce themselves and the purpose of the research activities. Appropriate verbal or written consent should be sought.
- Here is an example of a simple introductory strategy:
    - *Hello. I am a [researcher/health staff] working with [……] and we are asking people in the community about their thoughts and experiences regarding malaria and anti-malaria activities organized by MSPP, including the Community Health Councils (CHCs). We are asking people many different types of questions. The goal of our research is to understand how the malaria program can be improved. Is it okay if I ask you some research questions?*

Daily Activity Log

- You will be provided with a daily activity log. It should be filled in each day after your fieldwork and handed to the supervisor.

**Date:**			
**Location of research:**			
**Name of researcher:**			
**Method**	**Number**	**Socio-demographic characteristics of participant(s)[1]**	**Time spent on activity (hours per day)**
Key informant interview			
Focus group discussion			
Other____________			

[1] This should be entered based on rough age and gender. For the group discussion, a general description is fine; for example, a "group of young men."

Appendix 7: Community Feedback Form

Name of the CHC visited	
Date	
MSPP staff in attendance	
Implementation Partner staff in attendance	
# of CHC members in attendance	
Major findings from the community feedback	
Problems identified during the CHC meeting	
Strategies identified to move from the problem to the goal	
Agreed upon tasks required to achieve the strategy	
Issues to follow-up	

Appendix 8: Questions for a community-based KAP survey

It is advisable that community-based knowledge, attitude, and practice (KAP) surveys consist of relatively short questionnaires focused on key outcome indicators and tracking change over time. Below is a sample of questions that you may adapt for your use. Any questionnaire should be modified to fit program activities.

Section 1: Socio-demographic characteristics

1. Gender
2. Age
3. Level of education (defined as the final completed year of schooling)
4. Marital status
5. Occupation
6. Mobile phone ownership
7. How many people normally live physically in your house?
    - o How many adults [over 18]?
    - o How many children [from 6-18 years]?
    - o How many babies [5 and under]?
8. How many women are currently pregnant in your household?

Section 2: Awareness about malaria and the CHC

9. Have you heard about malaria?
10. When was the last time you heard messages about malaria?
11. Where did you last hear messages about malaria?
12. Have you heard about a local community group [name] focused on malaria control?
13. Have you participated in any activities organized by the local community malaria group?
    - o If yes, when?
    - o If yes, what activity were you involved in?
    - o If yes, did you have a positive, neutral, or negative experience participating with this group?
        - ▪ Explain your answer:____

14. What good things, if any, have you heard from other people about the local malaria group?
15. What bad things, if any, have you heard from other people about the local malaria group?

Section 3: Experiences and treatment of malaria

16. Have you had a family member who had malaria in the last [6-12] months?
17. What are the signs and symptoms of malaria?
    - Fever
    - Headaches
    - Chills
    - Nausea/vomiting
    - Body aches/ joint pain
    - Loss of consciousness
    - Jaundice
18. Have you had a fever in the last three months from [month] to [month]?
19. Did you seek help for your fever?
    - If yes, where?
20. Did you consult a community health worker?
21. What drugs did you receive?
22. Did you obtain malaria testing?
    - If yes, did you pay for your malaria test?
23. Were you positive for malaria?
24. How can you treat malaria?

Section 4: Malaria prevention

25. How is malaria spread? (Check all that applies)
    - Mosquito
    - Not sure
    - Dirty water
    - Other way: ____________________
26. How can you prevent malaria?
    - Mosquito nets:
    - Sleeping under a net,
    - Avoiding mosquito bites
    - Cutting grass around the house
    - Filling in puddles
    - Burning leaves,
    - Drugs / mass drug administration
    - Spraying a house with chemicals / IRS
27. Last night, did you sleep under a mosquito net?
    - If no, why not?
        - Net was used by others in the household
        - Net making them too hot
        - There were no mosquitoes around
        - The net was dirty
        - There was not enough space for the net

28. Do you own a mosquito net?
29. Where did you get your mosquito net?
30. How many mosquito nets do you have in your house?
31. Does everyone sleep under a mosquito net?

Section 5: MDA and IRS

If MSPP has implemented MDA or IRS in the area, additional questions on these interventions should be added here.

Appendix 9: Adaptive learning and participatory assessment workshop instructions

An adaptive learning and participatory assessment workshop should be organized every 6-12 months. There are a number of steps in organizing these workshops:

1) Pre-workshop preparations
2) Workshop facilitation
3) Reporting

Pre-workshop preparations

Organizing the CHC workshops will first require deciding on how often to organize the workshops and on the total number of CHCs and the number of members per CHC to invite. Ideally, workshops should be organized every 6-12 months to correspond with the distribution of prizes, as outlined in the Implementation Manual. This depends on the available budget and staff capacity. There are a few options for how to organize the participants:

- Invite a few (2-4) members of each CHCs to one large workshop;
- Invite a few (2-4) members of CHCs from only the sentinel monitoring sites to one large workshop;
- Organize a few workshops divided by Arrondissements in each Department. Either a few (2-4) or all CHC members could be invited.

It is important to plan a budget for the workshop that considers:

- Transport of all participants
- Food and drinks
- Paper, pens, flipcharts

It is important to coordinate the workshop with MSPP so that MSPP support staff for each CHC is in attendance at the workshop. These support staff will help facilitate the group exercises and report back to the CHC M&E officer with appropriate notes and data. It is important to decide on a clear SOP for the recording of the participatory assessment scoring and generation of stories of change. This should include notes,

flipcharts, and audio-recordings (which can be used to review the accuracy of the notes and flipcharts). At least 1 MSPP support staff should attend per CHC in attendance. Invitations should be formally sent to each CHC and MSPP support staff. The CHCs should be encouraged to decide, as a group, who should attend the workshop.

Before the workshop, it is important that all MSPP support staff read through the sections of the Implementation Manual that describe the planning for Community Action Plans and that they also familiarize themselves with this workshop protocol. CHCs members should come to the workshop with their Community Action Plans.

Workshop facilitation

The workshop should be a 1-day activity that begins at 9am and finishes around 5pm. An agenda should be provided for all participants. It should be divided into the following 5 activities:

Program feedback to CHCs	1 hour	Present findings from M&E data and provide malaria epidemiology update
Participatory assessment scoring	1 hour	Scoring 5 domains of the CHC program
Generation of stories of change	1 hour	Generating stories of change
Review Community Action Plans	3 hours	Update CHC Community Action Plans

1. Program feedback to CHCs

The workshop should begin by with brief presentations by the M&E team. Available data from the CHC program, including time trends on some of the output and outcome indicators, should be presented in a simple and accessible format. This should be followed by a brief presentation by the MSPP malaria coordinator, who should

summarize malaria trends. The malaria presentation should present the number of malaria cases and treatments by sub-commune location and the number of malaria rapid diagnostic tests (RDT) and microscopy tests performed, divided by +/-. The presentations should be followed by a question and answer period. The whole exercise should not last more than 1 hour total.

2. Participatory assessment scoring

The second part of the workshop should involve a scoring exercise. One MSPP support staff should convene each CHC; for this exercise, if possible, the MSPP support staff should not normally work with this particular CHC. The exercise should take 1 hour. The MSPP staff member should then explain the following information:

Explain that we are going to go through a series of 5 domains of the CHC program and we are going to score them. There are no right or wrong scores. This is a team exercise that we are using to explore what is going well and what needs improvement.

The MSPP support staff should arrange five flipchart pages on the table with the following headings:

- Leadership
- Planning and management
- Community involvement
- Partnership with MSPP
- Monitoring, evaluation, and learning

On each flipchart, the MSPP staff member should draw a circle adjacent to each member of the CHC who is present. This will be used by each individual CHC member to provide their own individual score. Put a large circle in the middle of the flipchart page, where the agreed upon group score will be presented. This should be done for each of the 5 flipchart pages.

Explain the scoring criteria and have them clearly written on the large flipchart for all CHC participants to see and reference. They are as follows:

- **Strong (5):** things have gone very well.
- **Strong but some improvement needed (4):** things have gone very well but some improvements are needed.
- **Average (3):** things have gone okay and there is room for improvement
- **Poor (2):** things have not gone very well and there is a lot of improvement needed.
- **Very badly (1):** things have gone very badly and we need to seriously change things

Explain how each of the 5 domains is going to be scored: We are now going to read the description of the domain first as a large group, meaning that it should be read and explained by senior program staff to the whole room. Questions should be taken to clarify any specific confusion. Handout sheets with the description of the domains should be provided to each group for reference. The groups should have 7 minutes to score each domain, at which point a bell or alarm should be rung and the senior staff should read the description of the next domain.

The description of each domain is provided below in Table 1.

It should be explained to the group that the MSPP staff member will be responsible for recording the reasons for why the group decided to score a particular domain the way it did.

At the end of the exercise, the MSPP staff member will collect the scores.

Table 1: Description of the 5 domains

This should be provided to each CHC for quick reference during the exercise

Domain	Description
1. Leadership	Consider the leadership of your CHC and how different members have interacted with each other, resolved conflicts, and contributed new ideas.
2. Planning and management	Consider how your CHC has been able to plan, adapt, and implement activities in the community.
3. Community involvement	Consider how well your CHC has involved the community.
4. Partnership with MSPP	Consider the level of partnership between your CHC and MSPP and the degree to which MSPP has supported your CHC activities.
5. Monitoring, evaluation and learning	Consider how well your CHC has discussed challenges and developed solutions to address them.

3. Generation of stories of change

Based upon the group discussion in the participatory scoring, CHC members should then transition, after a break, to the next activity. The Stories of Change exercise will consist of generating and documenting stories of positive change from CHC activities in the community and adaptive learning and management of the CHCs themselves. This will consist of CHC members, with the help of the MSPP support staff, brainstorming and reflecting on two questions:

1) "What are the most important changes that occurred in the community [since the last adaptive learning workshop]?"
2) "Why are these important for malaria elimination?"

The group may decide on as many important changes as possible; two to five (2-5) stories of change are ideal. The MSPP support staff will be responsible for documenting the stories of change by writing them down. Audio-recordings may also be used to ensure that all of the details are recorded.

4. Review Community Action Plans

The review of community action plans should follow the Stories of Change. This should involve each CHC re-visiting their Community Action Plans (CAPs). The first part of this exercise will involve the MSPP support staff facilitating a discussion with the CHC using the following discussion points:

1) *Does your CHC have a complete Community Action Plan?*
    a. *If not, what is missing? Why is it missing?*
    b. *If yes, what challenges has the CHC faced following the Community Action Plan?*

Depending on the answer to these questions, each CHC should be grouped with other CHCs in a similar stage of developing their Community Action Plan. MSPP support staff should be assigned to specific CHCs in order to facilitate CHCs to develop the particular areas of their Community Action Plan that is missing. If MSPP has any future plans for the implementation of any targeted interventions (such as IRS, MDA or LITNs distribution) or if there are any changes being made to the CHC program, then this should be discussed at this point in the workshop so that CHC Community Action Plans can be

updated appropriately.

The process of updating Community Action Plans should rely on the description of CAPs in the Implementation Manual. There are a series of Activities described in the manual, as noted here:

Table 2: List of 11 Activity Reports described in the Implementation Manual

Activity number	Title of Activity
Activity 1	Developing a vision of your CHC
Activity 2	Mapping malaria risk factors in your area
Activity 3	Develop a list of outstanding questions CHC members have regarding malaria
Activity 4	Discuss strengths and weaknesses of malaria approaches
Activity 5	Identify routine interventions
Activity 6	Identify where you want to implement your interventions
Activity 7	Identify who to target
Activity 8	Identify how often to implement your activities
Activity 9	Develop a 6-month activity calendar
Activity 10	CHC Rapid response plan
Activity 11	Material and supply plan

The focus in this workshop should be on updating Activities 8-11. This should include a focus on the neighborhood CHC groups, if relevant. Before the workshop, CHC members and MSPP support staff should have reviewed the details on how to create the Community Action Plans, from the Implementation Manual. All participants should come prepared, with copies of their Community Action Plans. It is up to MSPP and Implementation Partner staff to use this workshop to refine and update the CHC Community Action Plans. This part of the workshop should be divided into two 1.5 hours

sessions, for a total of 3 hours.

5. Provision of prizes

If the budget allows for the provision of prizes, as described in the Implementation Manual, then this should be done at the very end of the workshop before participants leave.
