# Supplementary material for: Evaluating a community engagement model for malaria elimination in Haiti: lessons from the community health council project (2019–2021)
Source: Malar J. 2023 Feb 9;22:47. doi: 10.1186/s12936-023-04471-z (PMC9910254; doi:10.1186/s12936-023-04471-z)
Supplement: Supplementary file 2 — Additional file 2. CHC M&E handbook English and French. [file 12936_2023_4471_MOESM2_ESM.zip › New folder (2)/CHC M&E Handbook_French.pdf]

2020

Conseils de Santé Communautaire en Haïti : Un Manuel d'Apprentissage, Suivi, et Evaluation

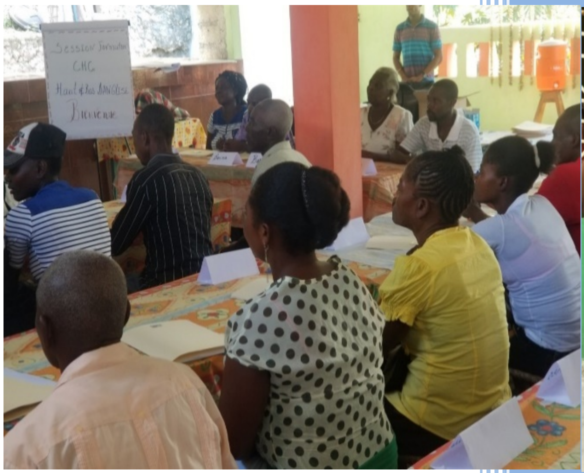

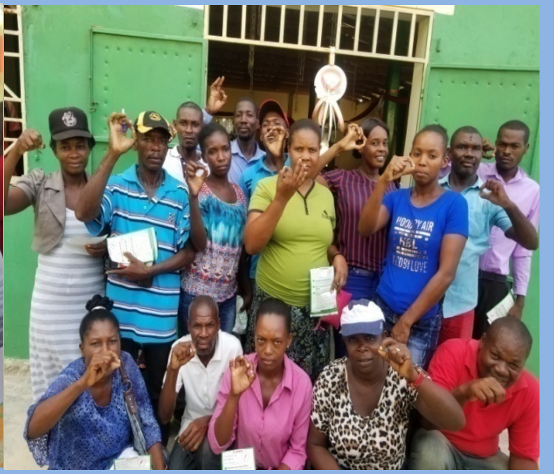

Ce manuel a été élaboré pour guider étape par étape le personnel du Ministère de la Santé Publique et de la Population (MSPP) et les autres partenaires d'exécution qui sont directement impliqués dans les activités de monitoring et d'évaluation (M&E) pour l'élimination de la malaria. Il fournit des directives détaillées sur la manière d'établir et de mener les activités de monitoring, d'évaluation et d'apprentissage pour les Conseils de Santé Communautaire (CSC). D'abord déployés dans le Département de la Grand Anse, les CSC sont des groupes d'action communautaire qui visent à renforcer la santé communautaire dans le but d'éliminer la malaria en Haïti. Ce manuel doit être lu en complémentarité avec le Manuel d'exécution des CSC. Ce manuel a été élaboré avec le soutien financier du Carter Center.

Citation suggérée:
Bardosh, K. Jean, L. Desir, L. Yoss, S, Nu, S. Poovey, B. Blount, S et Noland G. (2020). *Community Health Councils in Haiti: A Monitoring, Evaluation and Learning Handbook* [Les Conseils de Santé Communautaire en Haïti: Un Manuel d'Apprentissage, Suivi et Evaluation]. The Carter Center: Atlanta.

Table des Matières

COMMENT UTILISER CE MANUEL 1

CHAPITRE 1 : QU'EST-CE-QUE L'ENGAGEMENT COMMUNAUTAIRE ET POURQUOI EST-CE IMPORTANT ? 2

CHAPITRE 2 : MONITORING, EVALUATION, ET APPRENTISSAGE DANS LES PROGRAMMES DE LUTTE CONTRE LA MALARIA........ 5

CHAPITRE 3 : CADRE DE MONITORING ET D'EVALUATION POUR LES CONSEILS DE SANTE COMMUNAUTAIRE........ 16

CHAPITRE 4 : BOITE A OUTILS DE MONITORING ET D'EVALUATION DES CSC. 22

INTRODUCTION........ 22

1) RAPPORTS MENSUELS DE MONITORING........ 23

2) FORMULAIRE DE SUPERVISION ET D'EVALUATION 26

3) EVALUATIONS QUALITATIVES RAPIDES........ 26

4) REACTIONS DE LA COMMUNAUTE 30

5) ENQUETES BASEES DANS LA COMMUNAUTE SUR LES CONNAISSANCES, ATTITUDES ET PRATIQUES........ 32

6) ATELIERS D'APPRENTISSAGE ADAPTATIF ET D'EVALUATION PARTICIPATIVE.. 34

CHAPITRE 5 : LE PLAN DE TRAVAIL DE M&E DES CSC 36

CHAPITRE 6 : ANALYSE DE DONNEES, RAPPORTAGE ET APPRENTISSAGE DU PROGRAMME 41

ANNEXE 1: RAPPORTS DES ACTIVITES DU CSC 47

ANNEXE 2: SUPERVISOR FORMS 50

ANNEXE 3: RAPPORT DES ACTIVITES DU MSPP ET DU PARTENAIRE D'EXECUTION ... 55

ANNEXE 4: BANQUE DE QUESTIONS POUR LES EVALUATIONS QUALITATIVES RAPIDES 56

ANNEXE 5 : CONSEILS POUR LES ENTREVUES QUALITATIVES 59

ANNEXE 6 : ENREGISTREMENT DES DONNEES DANS LES EVALUATIONS QUALITATIVES RAPIDES 61

ANNEXE 7 : FORMULAIRE DE FEEDBACK COMMUNAUTAIRE 64

ANNEXE 8 : QUESTIONS POUR LES ENQUETES SUR LES CONNAISSANCES, LES ATTITUDES, ET LES PRATIQUES BASEES DANS LA COMMUNAUTE 65

ANNEXE 9 : DIRECTIVES POUR L'ATELIER SUR L'APPRENTISSAGE ADAPTATIF ET L'EVALUATION PARTICIPATIVE........ 68

Comment utiliser ce manuel

Ce manuel est un guide qui explique étape par étape le monitoring, l'évaluation, et les activités d'apprentissage pour utilisation par les Conseils de Santé Communautaire (CSC) pour l'élimination de la malaria en Haïti.

Il inclut des directives pour le personnel du MSPP, les membres du CSC et autres partenaires d'exécution dans la planification, la réalisation, l'analyse, le rapportage, et l'application des données de monitoring et d'évaluation pour améliorer l'initiative des CSC.

Le manuel doit être distribué au personnel du MSPP impliqué dans le monitoring, l'évaluation, et les activités d'apprentissage du CSC. Il doit être lu en accompagnement du manuel d'exécution du CSC qui décrit la façon d'établir et d'opérer un CSC.

Le manuel comporte six (6) chapitres et neuf (9) annexes.

Le Chapitre 1 présente les raisons pour lesquelles l'engagement communautaire est important pour l'élimination de la malaria.

Le Chapitre 2 souligne les principes de base du monitoring, de l'évaluation, et de l'apprentissage.

Le Chapitre 3 décrit le cadre de monitoring et d'évaluation (M &E) du programme CSC y compris le modèle logique.

Le Chapitre 4 fournit des instructions étape par étape pour six (6) méthodologies de monitoring et d'évaluation.

Le Chapitre 5 discute d'un nombre de considérations essentielles pour élaborer le plan de travail de monitoring et d'évaluation.

Le Chapitre 6 souligne les considérations principales pour l'analyse, le rapportage, et l'apprentissage.

Les neuf (9) Annexes fournissent les formulaires de collecte de données nécessaires, les banques de question et les instructions détaillées sur la façon d'utiliser les outils de monitoring et d'évaluation décrits dans ce manuel.

CHAPITRE 1 : Qu’est-ce-que l’engagement communautaire et pourquoi est-ce important ?

La lutte globale pour éliminer la malaria dépend de la participation active de divers groupes de personnes. Les études ont démontré de manière répétée que les interventions relatives à la malaria bénéficient d’une plus large couverture et d’une plus grande efficacité lorsque la population locale est impliquée dans les prises de décision. Ceci est vrai à travers toute la gamme des interventions anti-malariales – de la prise en charge des cas en passant par la lutte anti-vectorielle à l’administration massive de médicaments et à la surveillance. Ceci est aussi vrai dans les zones de faible transmission de la malaria comme c’est le cas actuellement dans la plupart des endroits en Haïti.

Les interventions qui ne sont pas basées sur les principes d’engagement communautaire (EC) sont plus aptes à confronter la résistance, le désengagement ou l’apathie au niveau communautaire ; elles ont aussi tendance à ne pas tenir compte des détails contextuels qui sont importants pour une meilleure planification et livraison. *L’engagement communautaire peut être défini comme étant : le processus de travailler en collaboration pour atteindre un ensemble d’objectifs communs qui améliorent les résultats au niveau sanitaire.* L’engagement communautaire implique le dialogue, la confiance, les activités collaboratives, le feedback, et l’apprentissage. Il n’existe pas de « menu » pour un engagement communautaire efficace dans l’élimination de la malaria ; mais plutôt un ensemble de principes qui doivent guider un processus qui dépend fortement de l’écoute de plusieurs voix et de leur incorporation dans les plans du programme.

Lorsqu’ils travaillent sur l’engagement communautaire, les responsables de la santé publique font face à un paradoxe. Si le programme est trop prescriptif, il risque d’encourager une approche du haut vers le bas qui n’implique pas suffisamment la participation de la communauté. Dans ce cas, nous pouvons dire que le programme est « *participatif* » mais, en réalité, la communauté et les voix locales ne sont pas vraiment prises en considération. D’autre part, tous les programmes requièrent des buts et objectifs prédéfinis - surtout ceux qui se concentrent sur une maladie (dans ce cas, l’élimination de la malaria) – et les experts doivent toujours fournir une orientation et des protocoles pour assurer la qualité et la reddition de comptes.

L’engagement communautaire est un processus qui prend du temps. Les relations et la confiance doivent se construire en plusieurs étapes d’un plan d’engagement communautaire efficace. Comme pour la promotion de la santé, la fréquence des activités d’engagement est très importante. Les gens passent du temps dans des activités qui selon eux sont importantes, et déterminent leurs idées de valeur en fonction d’un nombre complexe de perceptions, de croyances, de normes et de réalités sociales. Considérant que le risque de malaria en Haïti est relativement faible, ceci n’est pas forcément une priorité majeure pour les communautés locales – particulièrement celles dans les zones à faible transmission. Dans les zones à transmission élevée, l’urgence diminuera probablement au fur et à mesure que nous approchons de l’élimination.

Que sont les Conseils de Santé Communautaire (CSC) ?

Un CSC est une organisation bénévole dirigée par des résidents locaux, axée sur la réalisation de campagnes d’éducation et de changement de comportement, qui aide avec les activités de surveillance des maladies au niveau communautaire, le renforcement des liens entre la communauté et les professionnels de la médecine, et la mobilisation des résidents locaux dans la prévention des maladies. Le but principal du programme CSC est d’autonomiser les personnes, les familles et les communautés en Haïti pour renforcer les mesures locales pour la santé et le changement social de sorte à aider directement à l’élimination de la malaria. L’établissement initial d’une CSC met un accent particulier sur la prévention de la malaria et les activités de contrôle ; bien qu’une fois que les CSC deviennent fonctionnels et réalisent les activités pendant six (6) à douze (12) mois ils sont encouragés à s’engager dans d’autres problèmes de santé au-delà de la malaria. L’approche CSC a été établie initialement dans le Département de la Grand-Anse par le Carter Center, qui dirige l’engagement communautaire pour l’alliance Malaria Zéro[1], pour soutenir les efforts du MSPP pour l’élimination de la malaria en Haïti.

Même dans les zones à transmission élevée, la malaria n’est qu’un des nombreux problèmes confrontés par la population, et, en général, n’est pas le plus important. L’enthousiasme et la motivation communautaires pour la participation sont particulièrement importants, incluant les incitations intangibles telles que la reconnaissance, le sentiment d’apporter une contribution qui vaut la peine, et les connaissances acquises plutôt que par des incitations financières venant de

[1] Les partenaires incluent le Ministère de la Santé Publique et de la Population en Haïti, le Ministère de la Santé publique et de l’Assistance Sociale de la République Dominicaine, les Centres de Contrôle et de Prévention des Maladies des Etats-Unis, la Fondation CDC, L’Organisation Panaméricaine de la Santé, le Carter Center, la Clinton Health Access Initiative, l’Ecole d’Hygiène et de Médecine Tropicale de Londres, et l’Ecole de Santé Publique et de Médecine Tropicale de l’Université de Tulane

l'extérieur. L'engagement communautaire peut aussi exiger un regard « hors des sentiers battus » de la malaria pour aussi aborder simultanément des prestations de soins de santé et des problèmes de maladies autres.

L'engagement communautaire (EC) doit travailler avec et renforcer les capacités locales, les structures et le leadership communautaires. Ceci implique que les membres de la communauté se mettent ensemble pour analyser le problème de la transmission, de la prévention et du contrôle de la malaria et utiliser cette analyse pour élaborer des plans d'action en vue d'aborder ces problèmes. Ils peuvent aussi analyser d'autres problèmes de santé et de maladies. L'engagement communautaire est un processus de facilitation sociale et de changement communautaire en vue d'aborder les normes sociales, les questions de prestation de soins de santé et les déterminants comportementaux. Il exige aussi de la flexibilité et une capacité d'adaptation.

Pour toutes ces raisons, le monitoring, l'évaluation et l'apprentissage sont des parties très importantes des programmes d'engagement communautaire, dont le Programme de Conseils de Santé Communautaire (CSC) en Haïti qui se concentre sur l'élimination de la malaria.

Ce manuel doit être lu et utilisé en accompagnement le Manuel d'Exécution des CSC.

Bardosh, K., Jean, L., Desir, L., Yoss, S., Nu, S., Poovey, B., Blount, S., et Noland, G. (2020). Community Health Councils (CSC): *An Implementation Manual for Community Engagement and Malaria Elimination in Haiti* (Manuel de Mise en Oeuvre for l'Engagement Communautaire et l'Elimination de la Malaria en Haïti). The Carter Center: Atlanta.

CHAPITRE 2 : Monitoring, Evaluation, et Apprentissage dans les programmes de lutte contre la malaria

2.1. Monitoring et évaluation dans le cycle du projet

Le monitoring et l'évaluation (M&E) constituent une partie cruciale de n'importe quel programme de santé efficace. En retraçant les activités et la réponse des communautés, le système M&E facilite la reddition de comptes, l'apprentissage et l'adaptation. Le M&E aide à renforcer la capacité du personnel et des membres de la communauté en analysant la prestation et les dimensions sociales du programme. Ce faisant, M&E améliore la résolution des problèmes et peut être utilisé pour améliorer la qualité du programme et son efficacité, lorsque combiné avec des activités d'apprentissage intégrées dans le processus de planification du programme. Enfin, les données du M&E jouent aussi un rôle essentiel dans la justification des ressources et des investissements, et aide à planifier les activités et les programmes futurs.

La pratique d'engagement communautaire (EC) doit être basée sur des preuves. Très souvent, les autorités sanitaires font des activités communautaires et mobilisent des groupes locaux par des moyens qui ne sont pas aussi efficaces qu'ils pourraient l'être. Il est important de monitorer et d'évaluer l'engagement communautaire. Les activités de monitoring et d'évaluation décrites dans ce manuel aideront le personnel dévoué à l'élimination de la malaria et les membres de la communauté en Haïti à mieux planifier et exécuter les interventions contre la malaria.

Figure 1 : le cycle du projet

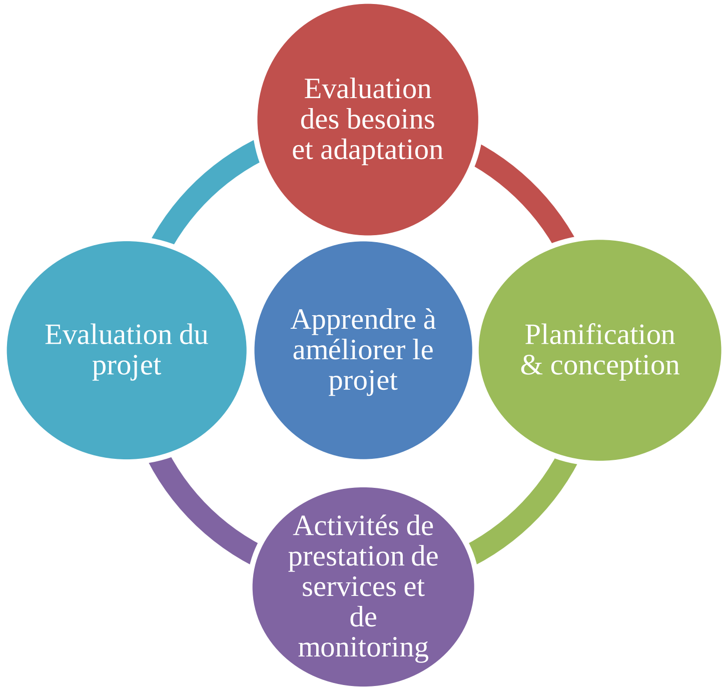

Le feedback et l'apprentissage générés par les données et l'analyse du monitoring et de l'évaluation ont la capacité d'améliorer dramatiquement les programmes de santé si ces systèmes sont conçus de manière avisée et réfléchie avec des ressources suffisantes. Malheureusement, dans beaucoup de programmes, le M&E est traité comme une activité secondaire, détachée de la planification et de l'exécution ; par exemple, strictement concentrée sur les données quantitatives qui résument le nombre de personnes qui ont été touchées et le nombre d'activités réalisées. Ceci est malheureusement une idée limitée du potentiel du monitoring et de l'évaluation. Le point de vue le plus large est basé sur un engagement partagé à collecter une plus grande gamme de données (quantitatives et qualitatives) et à utiliser ces données et cet apprentissage pour informer la planification et les prestations. Ce processus est illustré ci-dessus à la **Figure 1**.

Le monitoring et l'évaluation sont des activités liées entre elles. Le **monitoring** est une activité constante qui mesure les progrès à travers la collecte routinière de données et aide à fournir des informations pour des adaptations en temps réel à savoir si oui ou non les activités sont fidèlement exécutées et achèvent les résultats anticipés. Il implique la collecte de données pour retracer le progrès par rapport au plan de travail. Le but du monitoring est de corriger et d'adapter – de faire des corrections à mi-parcours et de corriger les problèmes au fur et à mesure qu'ils se présentent. Le monitoring est une forme de feedback. En faisant du monitoring, il est important d'évaluer l'importance des petits détails et des effets de l'exécution. Les détails sont importants. Le monitoring permet au programme d'ajuster les divers détails associés à l'exécution.

L'**évaluation** est une forme de monitoring plus formelle et plus générale qui se fait typiquement à mi-parcours ou à la fin d'un projet. Elle est plus rigoureuse et plus complexe et concentrée sur des questions d'efficience, de pertinence et d'impact. L'évaluation est utilisée pour prôner la réussite d'un programme dans l'atteinte des résultats désirés, comme les connaissances et le changement de comportement, ou la réduction des cas de malaria. Souvent, le but de l'évaluation est d'apprécier l'efficience et la valeur d'une approche ou d'une politique et la raison pour laquelle elles ont ou n'ont pas marché. Elle est plus programmatique et concentrée sur la documentation des plus grandes leçons apprises, en vue de prôner la raison pour laquelle l'approche générale a été prise.

Les systèmes M&E permettront à un programme d'engagement communautaire :

- **D'améliorer les activités** et les plans en apprenant ce qui marche et ce qui ne marche pas ;
- **D'améliorer le processus de planification** et de conception du programme ;
- **D'évaluer les résultats** du programme et si oui ou non il a atteint les objectifs prévus ;
- **De déterminer le bien-fondé** des éléments et des activités du programme ;
- **De prôner l'efficacité ;**
- **De démontrer la valeur** de l'engagement communautaire ;
- **D'acquérir une crédibilité** et un soutien en montrant les résultats et l'impact.

2.2. Présentation du modèle logique

Les plans M&E suivent un cadre qui souligne les façons d'envisager un programme qui marche et les moyens dont les activités M&E doivent suivre et mesurer les progrès. Ceci est typiquement décrit dans un **modèle logique** ou cadre logique, qui articule la logique du projet. Un modèle logique est un outil de management qui aide à clarifier et à réviser les activités et les résultats du projet par rapport à ses visées et objectifs. Le modèle logique souligne aussi les voies et mécanismes anticipés sur la façon dont les activités réaliseront le changement. Voir la **Figure 2** ci-après:

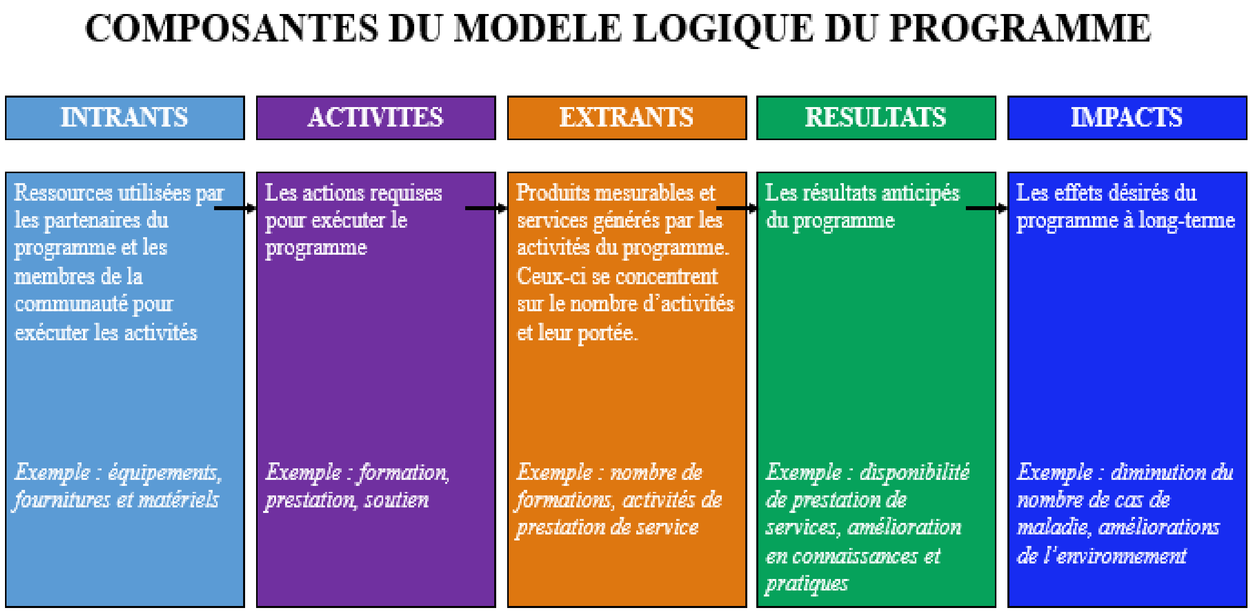

En conceptualisant un système M&E, il est important de demander :
« Que devons-nous savoir ? » et « qui utilisera ces informations et à quelle fin ? »

2.3. Indicateurs

Les intrants, les extrants, les activités et les résultats décrits dans les modèles logiques peuvent être utilisés comme modèles pour élaborer les **indicateurs**. Les indicateurs définissent le focus de la collecte de données et l'analyse du monitoring et e l'évaluation. C'est le moyen par lequel la réussite sera mesurée. Bien que certains indicateurs soient relativement clairs et simples à mesurer, d'autres sont beaucoup plus complexes et difficiles. Ceci est particulièrement vrai dans les programmes d'engagement communautaire où nous sommes aussi intéressés à monitorer le processus de changement.

Les indicateurs agissent comme les marqueurs au bord d'une route qui montrent la distance couverte du départ à l'arrivée. Un exemple est fourni ci-après lié à la cuisson du riz. Les indicateurs aident à retracer une variable dans le temps et incluent les mesures à court et à moyen-terme pour les extrants et les résultats (voir la **Figure 3**).

**Cuisson du riz**	
**Activité**	**Indicateur**
Feu prêt	Flammes
Eau qui bout	Bulles et vapeur à la surface de l'eau
Riz bien cuit	Le grain est tendre et a bon goût

Un bon indicateur devrait mesurer de près le changement envisagé avec une grande spécificité ; en réalité, un ensemble d'indicateurs est souvent établi pour trianguler un changement anticipé à partir de plusieurs angles. Ceci fournit une plus grande exactitude. **En général, les indicateurs ne doivent pas être modifiés une fois commencées les activités M&E.** Cependant, des modifications peuvent être apportées si les objectifs et les approches du programme sont changés, mais il faut considérer les risques/bénéfices.

Les indicateurs peuvent inclure des variables quantitatives (numériques) et qualitatives (descriptives). Bien que **les indicateurs quantitatifs** tendent à être plus largement utilisés, ils présentent des lacunes pour la compréhension des programmes d'engagement communautaire. Il est important de retracer le nombre d'activités exécutées (par exemple : les réunions ou la répartition des médicaments et des moustiquaires) pour comprendre la mise en œuvre du programme. Mais ils fournissent des perspectives très limitées sur la *qualité* de l'exécution et les effets des activités sur les membres de la communauté. Les interventions de l'engagement communautaire dépendent des compétences personnelles de persuasion, de dialogue et de l'action collective qui sont inaccessibles aux indicateurs quantitatifs. Pour cette raison, il est important que les cadres M&E considèrent l'inclusion d'**indicateurs qualitatifs** qui retracent les facteurs liés au *processus* et au *contexte* de l'engagement communautaire.

Le M&E dans les programmes d'engagement communautaire

Il n'existe pas de « référence absolue » ou de M&E standard qui peut être utilisé dans tous les programmes d'engagement communautaire. La preuve d'engagement communautaire n'indique pas clairement les indicateurs spécifiques liés à la qualité du processus et aux résultats. En fait, malgré l'acceptation que l'engagement communautaire ajoute de la valeur aux programmes de santé, incluant l'élimination de la malaria, des écarts considérables demeurent dans la science de l'engagement communautaire. Prouver là où les actions et pratiques de l'engagement communautaire sont les plus efficaces – leur synchronisation, fréquence et intensité (dosage) – est très spécifique au contexte. Ceci fait de l'évaluation de l'engagement communautaire un vrai défi. Il n'existe aucun standard probant et direct. Chaque programme d'engagement communautaire doit plutôt développer sa propre stratégie pour atteindre les objectifs du projet en fonction des facteurs contextuels et des besoins.

Les indicateurs sont utilisés pour retracer les **intrants, les extrants,** et **les résultats.** Les extrants se rapportent aux activités, aux services, aux évènements et aux produits du projet. En contraste, les résultats se réfèrent à la différence qui est faite. Les résultats sont l'aboutissement direct, les bénéfices, les avantages ou désavantages pour les personnes, les familles, les groupes, les communautés, les organisations ou les systèmes. La plupart des projets trouvent qu'il est facile d'enregistrer et de décrire les activités qui ont été réalisées et le nombre de personnes desservies. L'aspect du monitoring et de l'évaluation qui présente le plus de défis est lié aux résultats et à l'impact d'un projet : *quelle différence a été faite ? Quels changements se sont produits à cause*

du projet ? Ces questions sont retracées à travers les indicateurs de résultat. Voir la **Figure 2** ci-dessus pour une définition des termes communs de monitoring et d'évaluation que nous utiliserons dans les prochains chapitres.

2.4. Le Monitoring et l'Evaluation dans le cycle du projet.

Il y a peu d'étapes distinctes de monitoring et d'évaluation dans le cycle d'un programme typique (**Figure 3**). La première implique une phase **d'évaluation formative** (aussi dénommée *évaluation des besoins* ou parfois tout simplement *recherche exploratoire*). La phase d'évaluation formative est utilisée pour concevoir et planifier le projet, de même que pour réviser les buts, les objectifs et les méthodologies du programme avant ou juste après l'exécution du projet. Par exemple, elle peut inclure la recherche qualitative (discussions des groupes focus, entrevues semi-structurées) sur :

- Les perceptions et expériences locales de la malaria ;
- Les normes et pratiques socio-culturelles liées au traitement à la prévention et au contrôle de la malaria ;
- Les types d'organisation et de groupes communautaires qui opèrent dans la zone, leurs forces et leurs faiblesses ;
- Les matériels d'éducation sur la santé et les canaux existants de communication sur la santé ;
- L'acceptabilité des activités anti-malaria passées et actuelles et des interventions possibles à l'avenir.

Une évaluation formative donne au personnel une possibilité d'explorer les perceptions, de tester l'acceptabilité des plans d'intervention proposés, et d'inviter les membres de la communauté locale à aider dans la définition des priorités et des activités. Elle inclut la recherche faite avant l'exécution de n'importe quelle activité ou peut faire partie d'une phase de petits projets pilote où certaines activités du projet sont exécutées et évaluées – par exemple, pré-test d'une affiche ou d'une annonce radiodiffusée sur l'éducation au sujet de la malaria avec les membres de la communauté.

Figure 3 : les étapes que comportent un cycle typique de monitoring et d'évaluation

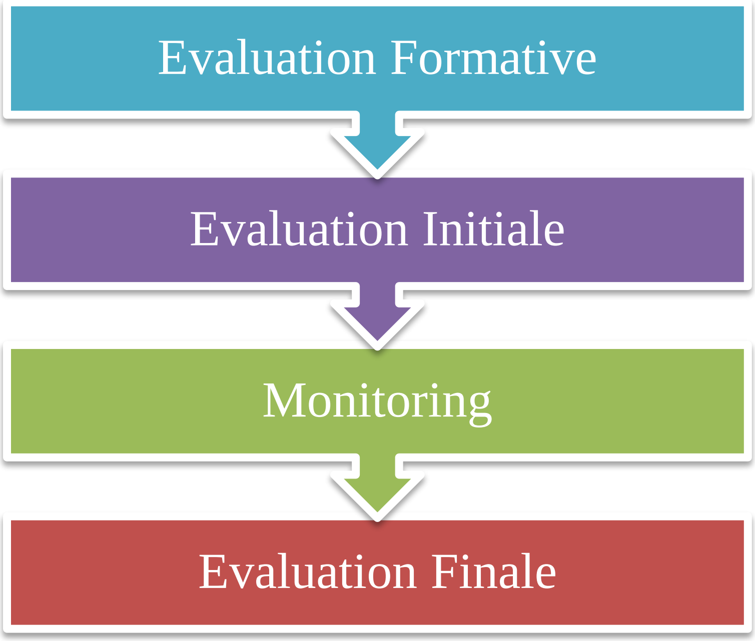

Une deuxième composante du cycle de monitoring et d'évaluation est **l'évaluation initiale.** Ceci doit émerger de la phase de recherche formative mais peut demander des collectes de données supplémentaires axées sur une série d'indicateurs de résultat raffinés et focalisés que l'intervention d'engagement communautaire a décidé de monitorer dans le temps. En fournissant une image *avant et après* basée sur les données, une évaluation initiale permet au programme de prôner des changements dans le temps. Les indicateurs de résultats peuvent inclure des choses comme :

- Connaissances des bénéficiaires ;
- Risques perçus ;
- Normes et attitudes ;
- Auto-efficience ;
- Comportements et pratiques ;
- Données épidémiologiques ;
- Distribution géographique des services ou des facteurs de risque environnementaux.

Lorsqu'une évaluation initiale n'est pas possible ou disponible, il y a un nombre de stratégies de recherche qui peuvent donner une perception sur les changements liés au programme. Par exemple : on peut demander aux bénéficiaires et aux participants à la recherche des:

1. Questions rétrospectives : qu'avez-vous fait ou pensé avant le projet ?
2. Questions hypothétiques : si le projet n'existait pas, qu'est-ce-qui serait différent aujourd'hui ?
3. Questions basées sur le changement : qu'est-ce-qui a changé depuis le projet ? Qu'est-ce-qui a changé à cause du projet ?

De même, si le but ultime de l'évaluation est de mesurer le processus d'exécution lui-même, des données initiales robustes pourraient ne pas être aussi importantes que lorsque le but est de mesurer seulement les changements de comportement avant et après une intervention.

D'habitude, les évaluations initiales se reposent lourdement sur les données qui sont facilement mesurées et quantifiables (mais idéalement, doivent aussi utiliser des données qualitatives). L'évaluation initiale doit être revisitée en utilisant la même méthodologie, dans **l'évaluation finale**. L'évaluation finale (souvent tout simplement « évaluation ») fait référence à la phase finale de collecte de données, où les intrants, les indicateurs, les extrants et les résultats sont examinés par rapport aux cibles d'origine et les réussites et les défaillances du programme sont déterminées. Durant le programme, alors que les interventions sont en cours, le personnel du programme doit se concentrer sur le monitoring de divers indicateurs à côté des activités du programme. Le **monitoring** fait référence au retracement des indicateurs entre les évaluations initiales et finales. Ceci inclut typiquement des données quantitatives sur le nombre de réunions, de matériels produits, de populations touchées et de personnes formées (données quantitatives) ; mais doit aussi inclure des données qualitatives sur le processus d'exécution (qu'est-ce-qui marche bien et quels défis se sont présentés ?) ainsi que l'acceptabilité, les réactions, le niveau de participation et l'engagement des bénéficiaires du projet et du personnel du programme par rapport aux activités exécutées.

Pour que le monitoring et l'évaluation aient le plus d'impact sur les programmes d'engagement communautaire, il est important d'inclure des méthodes de monitoring et d'évaluation

participatives et de prêter une attention particulière sur la façon d'utiliser la collecte et l'analyse des données et par qui. Une bonne façon de penser à cela est de concentrer le monitoring et l'évaluation sur l'apprentissage social : apprendre à connaître un contexte social tout en étant activement engagé dans ce contexte. La qualité de **l'apprentissage social** est un des facteurs les plus importants du travail d'engagement communautaire et l'utilisation des données de monitoring et d'évaluation pour améliorer l'exécution. L'apprentissage doit se faire à plusieurs niveaux alors que le programme développe des relations de confiance mutuelle avec les membres de la communauté. Ceci devrait en partie impliquer les membres de la communauté dans la collecte et l'interprétation des données (de même que le personnel du programme qui, à leur tour, fournit les données aux membres de la communauté, par exemple les rapports épidémiologiques sur la malaria), afin de faciliter l'apprentissage au niveau communautaire. La collecte et l'utilisation des données doit être un processus participatif et non pas un exercice du haut vers le bas.

L'apprentissage ne doit pas être limité aux « experts » qui collectent les données sur le terrain, les analysent dans leurs bureaux, et les redistribuent à la communauté. Les communautés doivent être impliquées dans la planification, la collecte, l'analyse et le rapportage des données de monitoring et d'évaluation.

L'approche d'apprentissage social est une partie importante pour développer des partenariats basés sur la confiance où des opportunités répétées sont trouvées pour adapter les plans basés sur le feedback des membres de la communauté et les équipes de terrain. Ceci exige : un esprit ouvert, l'inclusion, le dialogue, la transparence et le respect mutuel. Connaissance égale pouvoir et partager les connaissances augmente la confiance et l'appropriation des activités.

Le monitoring participatif fait partie du modèle d'apprentissage social, il s'agit d'une méthodologie de plus en plus populaire dans les programmes d'engagement communautaire. Ceci se passe pendant l'exécution et constitue un domaine clé pour promouvoir le leadership communautaire, l'analyse des problèmes et la mobilisation sociale. Il utilise les habilités et les ressources locales et facilite les analyses de groupe. Il force aussi le personnel du programme à examiner leurs hypothèses sur l'aspect du progrès et leur permet d'avoir une meilleure appréciation de la façon dont les membres de la communauté locale voient les buts et objectifs du programme.

Le programme peut utiliser ces informations pour adapter et modifier ses stratégies et ses plans. Ceci peut inclure des méthodes telles que :

1. Ateliers participatifs ;
2. Auto-évaluation et exercices de hiérarchisation ;
3. Raconter des histoires ;
4. Panels d’interprétation ;
5. Promenades d’étude, groupe focus et entrevues semi-structurées ;
6. Cartographie communautaire et sociale ;
7. Analyses des échéanciers et des tendances ;
8. Calendrier saisonnier ;
9. Journaux et enregistrements systématiques des récits.

Ce type de modèle d’apprentissage social requiert **des compétences différentes** pour le monitoring et l’évaluation parce qu’il implique un processus de facilitation axé sur le changement social. Ceci présente des défis car il exige des compétences « diplomatiques différentes » et une expertise communément disponible aux équipes de santé publique et de recherche biomédicale. Il y a beaucoup **d’obstacles à l’apprentissage social** dans un programme d’engagement communautaire. Bien que ces obstacles soient souvent rejetés dû à un manque de ressources, les capacités du personnel et/ou la volonté politique, tel n’est pas toujours le cas ; des systèmes de monitoring et d’apprentissage mal-conçus et exécutés sont aussi des erreurs communes. Les faiblesses typiques rencontrées dans le monitoring et l’évaluation incluent : des méthodologies inappropriées, des données de faible qualité et collectées de manière aléatoire, des analyses lentes et inefficientes des données, la mauvaise dissémination et présentation des résultats, et une réticence culturelle par rapport à l’utilisation des données pour faciliter l’adaptation et le changement. Un plan de monitoring et d’évaluation et une équipe de terrain dévoués et rigoureux avec un mode opératoire standardisé et clair constituent l’un des meilleurs recours pour ce genre de chose.

Une liste de vérification des bonnes pratiques de monitoring et d'évaluation doit:

- ✓ Servir à des fins programmatiques intégrées à des phases de planification et d'exécution ;
- ✓ Etre participatif, impliquant le personnel du programme et les membres de la communauté de manière appropriée ;
- ✓ Avoir des méthodes et des indicateurs spécifiques, mesurables, appropriés et réalistes ;
- ✓ Etre concentré sur la production de nouvelles connaissances pratiques ;
- ✓ Etre accessible et facile à comprendre ;
- ✓ Aider à augmenter les capacités des bénéficiaires du projet ;
- ✓ Avoir des ressources adéquates ;
- ✓ Considérer le contexte socio-culturel et politique du programme ;
- ✓ Aborder les suppositions théoriques du projet ;
- ✓ Mettre en place un cadre éthique approprié pour protéger la confidentialité des personnes et les protéger des abus ou de la coercition ;
- ✓ Etre disséminé aux membres de la communauté et autres parties prenantes ;
- ✓ Explorer les faiblesses et les forces du projet ;
- ✓ Fournir des preuves de haute qualité pour les décideurs et les bailleurs de fonds.

CHAPITRE 3 : Cadre de monitoring et d’évaluation pour les Conseils de Santé Communautaire

Introduction

Les activités de monitoring et d’évaluation du projet ne peuvent pas tout mesurer. Il est important de bien réfléchir sur ce qui doit être mesuré par rapport au budget disponible et aux restrictions de temps du programme. Pour ce faire, les plans de monitoring et d’évaluation doivent se reposer sur un cadre de monitoring et d’évaluation qui énumère tous les indicateurs d’un programme, les sources de données, la fréquence de mesurage des indicateurs, et la personne responsable de les mesurer. Ce cadre est utilisé pour guider la collecte de données, l’analyse et le rapportage du monitoring et de l’évaluation. Il est important de bien se familiariser avec le cadre de monitoring et d’évaluation et les divers indicateurs discutés dans ce manuel. Ils forment le noyau du Plan de Monitoring et d’Evaluation.

Chaque cadre de monitoring et d’évaluation doit être élaboré sur la base d’une **théorie de changement** et/ou d’un **modèle logique** du programme. Une théorie de changement décrit dans l’ensemble la façon dont une certaine série d’interventions peut mener à un changement à long terme. Elle doit capturer la complexité du changement dans le contexte élargi du programme au niveau social, culturel et économique, politique et environnemental. Ainsi, elle montre les différentes voies de changement, même si elles ne sont pas liées au projet. Elle suscite la question suivante : « *si nous faisons X alors Y changera parce que…* ». Une théorie de changement est souvent présentée comme un diagramme flexible avec des textes descriptifs. La théorie du cadre de changement des CSC est soulignée dans la **Figure 4.**

Figure 4 : La théorie du CSC du cadre du changement

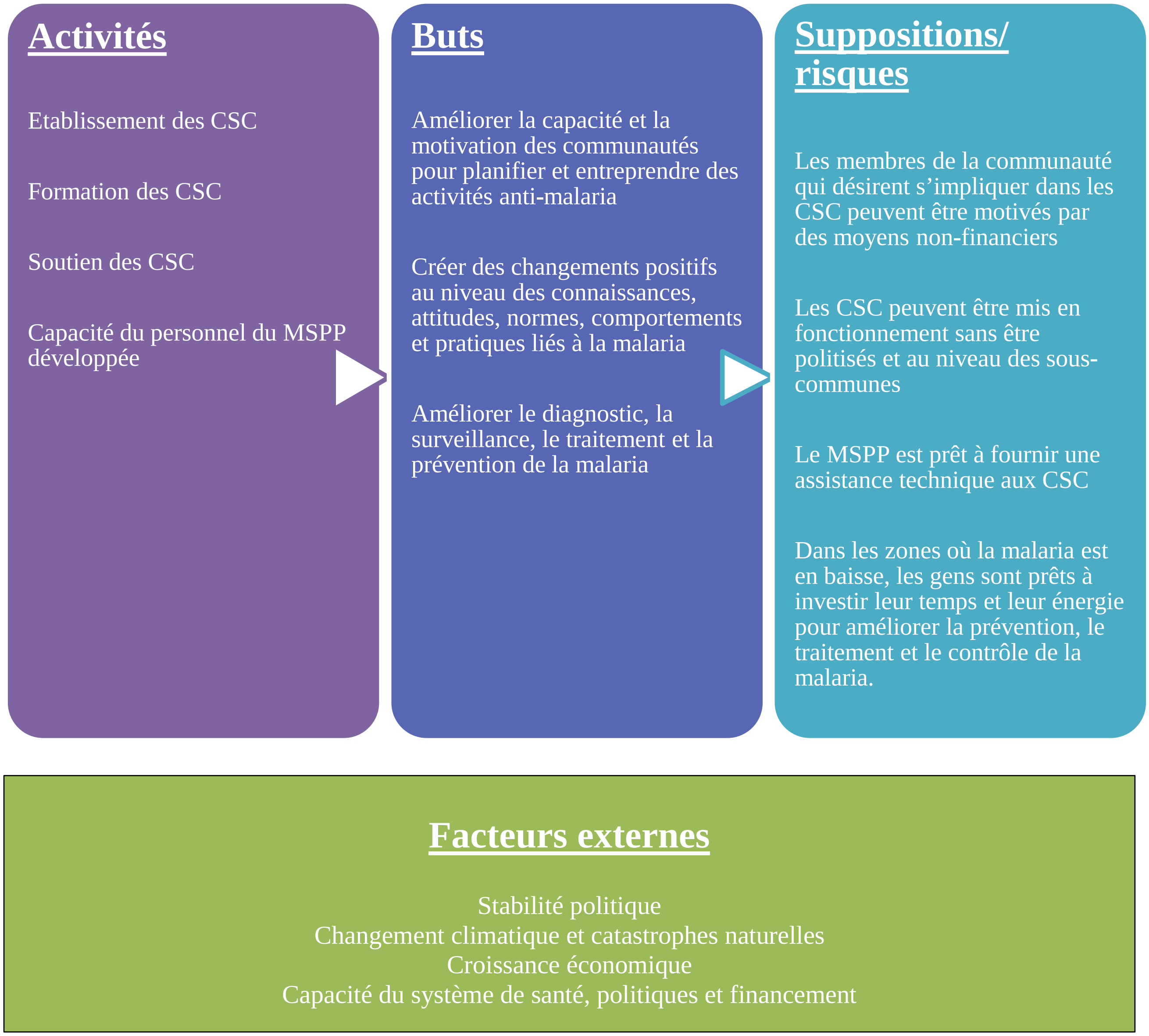

Par contraste, le modèle logique illustre la façon dont le processus de changement se fera, au niveau programmatique (exécution). Le modèle logique est seulement concentré sur les composantes directes du programme (intrants, activités, extrants, résultats et impacts) liées au changement. Le modèle logique pour le programme CSC est reflété à la **Figure 5**.

Figure 5 : Modèle logique du Conseil de Santé Communautaire

Problem Statement: In areas of declining malaria transmission, local populations do not consider malaria to be a priority, in part due to the lack of community awareness and engagement in prevention, treatment, and control efforts.

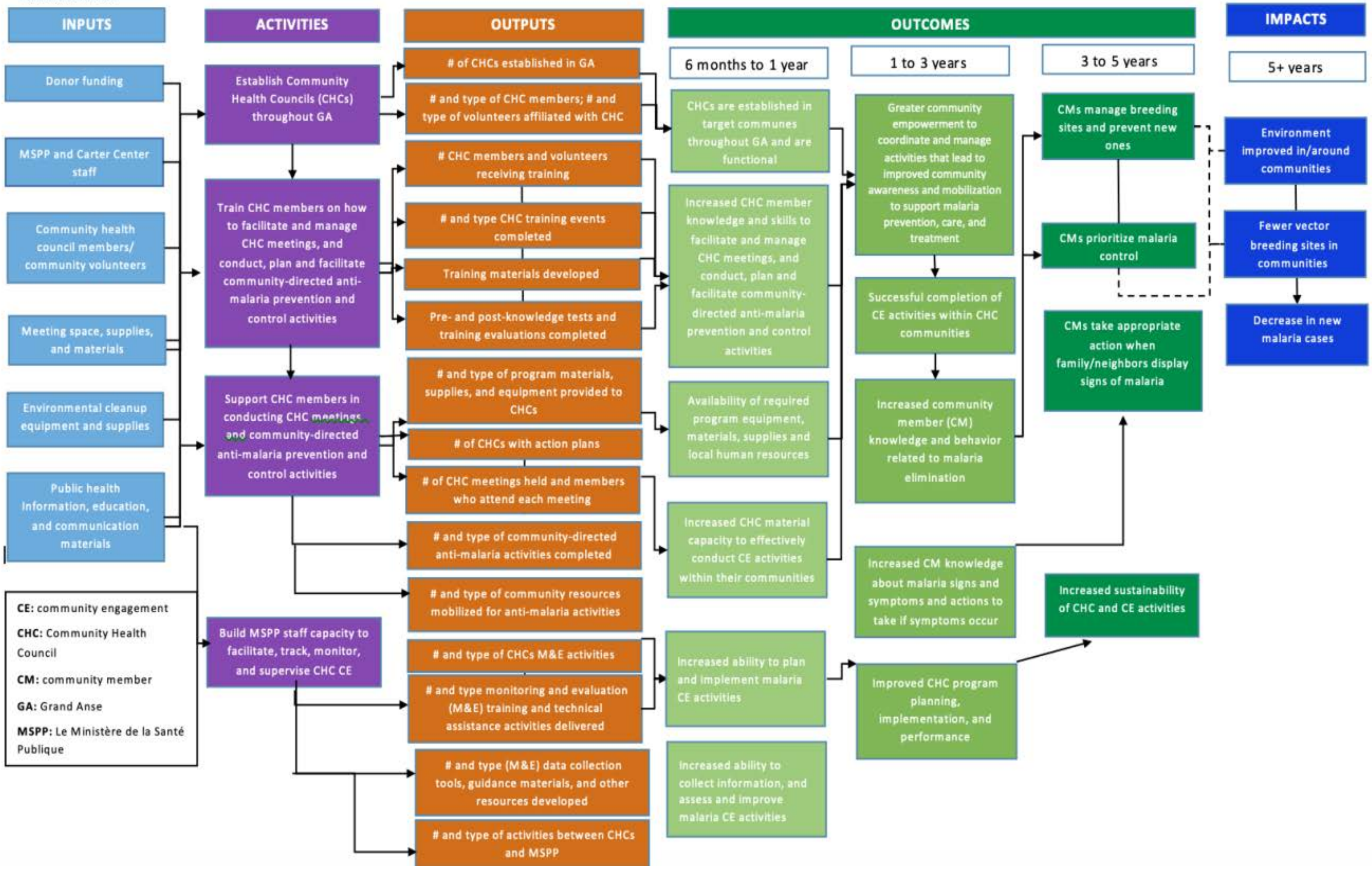

Enoncé du problème : dans les zones où il y a un déclin dans la transmission de la malaria, les populations locales ne considèrent pas la malaria comme étant une priorité due en partie au manque de sensibilisation communautaire et d'engagement dans les efforts de prévention, de traitement et de contrôle.

La première partie du modèle logique du CSC inclut les intrants et les activités du programme (voir la **Figure 6**). Les activités du programme CSC incluent :

1) ***Etablissement*** des CSC ;
2) ***Formation*** des membres des CSC sur la planification, la conduite et la facilitation des réunions CSC et des activités de prévention et de contrôle de la malaria dirigées par la communauté dans les diverses agglomérations et leurs sous-communes;
3) ***Soutien*** aux membres des CSC pour la tenue de leurs réunions et dans les activités de contrôle et de prévention de la malaria dirigées par les communautés dans leurs diverses agglomérations et leurs sous-communes.
4) ***Renforcement*** des capacités du personnel du MSPP pour faciliter, localiser, monitorer et superviser les activités d'engagement communautaire dans les CSC.

Figure 6 : Intrants et activités du Modèle Logique des CSC.

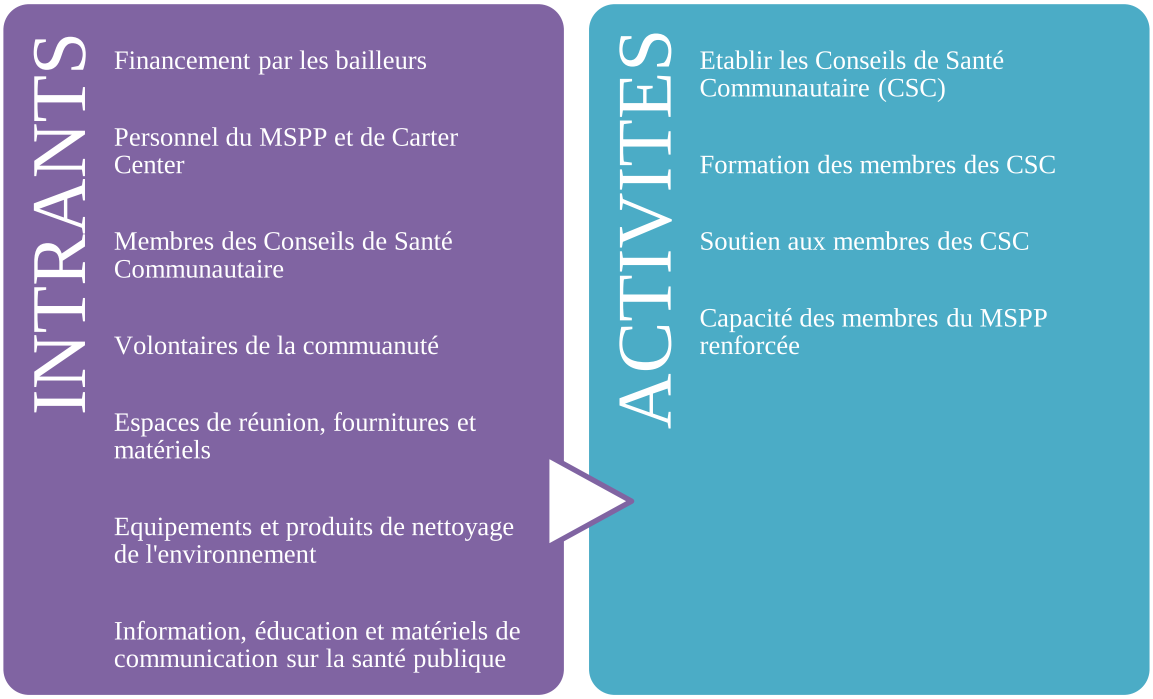

Pour chacune des quatre (4) activités énumérées ci-dessus, il y a une série correspondante d'indicateurs d'intrants et d'extrants. Dans le tableau ci-après nous soulignons les indicateurs d'extrants quantitatifs appropriés et énumérons une série d'indicateurs de résultats. Selon les méthodes utilisées dans les activités de monitoring et d'évaluation, les indicateurs de résultat peuvent être quantitativement et/ou qualitativement évalués, par exemple : l'indicateur de résultat 3A (*augmentation de la sensibilisation sur l'importance du contrôle et de la prévention de la malaria*) peut être évalué soit comme une augmentation du pourcentage (%) dans le temps dans une enquête initiale et finale ou par l'utilisation de données qualitatives.

Les Activités CSC: Indicateurs-Clé du Programme

Activité	Indicateurs d'extrants	Indicateurs de résultats
1.0 Etablir des CSC à travers toutes les zones géographiques	1.1. Nombre (#) des CSC établis par rapport à la cible<br>1.2. Nombre (#) de CSC qui participant aux activités d'engagement communautaire par rapport à la cible<br>1.3. Nombre (#) de volontaires communautaires affiliés aux CSC	1.a. Présence de CSC fonctionnels à travers la zone géographique durant la période de rapportage du programme avec un réseau de volontaires actifs
2.0 Former les membres des CSC sur la façon de gérer leurs réunions et mener, planifier et faciliter les activités de prévention et de contrôle de la malaria axées sur la communauté	2.1. Nombre (#) et types de membres des CSC et volontaires formés par rapport à la cible<br>2.2. Nombre (#) et types de formation en CSC réalisés par rapport à la cible<br>2.3. Matériels de formation élaborés<br>2.4. Tests de connaissances avant/après et évaluations des formations	2.a. Membres des CSC qui ont démontré des connaissances améliorées après les activités de formation
3.0 Soutenir les membres des CSC dans la réalisation de leurs réunions et leurs activités de prévention et de contrôle de la malaria axées sur la communauté	3.1. Nombre (#) et types de matériels de fournitures et d'équipements distribués aux CSC par rapport à la cible<br>3.2. Nombre (#) de CSC qui ont des plans d'action<br>3.3. Nombre(#) de réunions de CSC tenues annuellement et pourcentage (%) des membres présents<br>3.4. Nombre(#) et types d'activités anti-malaria réalisées par rapport à la cible<br>3.5. Nombre (#) et types de ressources communautaires mobilisées pour les activités anti-malaria<br>3.6. Nombre (#) de matériels éducatifs sur la santé distribués aux membres de la communauté par rapport à la cible<br>3.7. Nombre (#) de membres de la communauté touchés à travers les activités anti-malaria réparties par types d'activité	3.a. Plus grande sensibilisation sur l'importance du contrôle et de la prévention de la malaria<br>3.b. Plus grande sensibilisation sur les signes et symptômes de la malaria et mesures à prendre en cas de symptômes<br>3.c. Opinions et expériences positives des CSC<br>3.d. Changements de comportement liés au risques, à la prévention et au traitement de la malaria<br>3.e. Histoires de changements des CSC
4.0 Renforcer la capacité du personnel du MSPP pour retracer, monitorer et superviser les activités des CSC et de l'engagement communautaire	4.1. Nombre (#) et types d'activités de monitoring et d'évaluation des CSC<br>4.2. Nombre (#) et types d'orientation d'outils et de ressources de monitoring et d'évaluation<br>4.3. Nombre (#) et types d'activités et d'assistance technique en monitoring et évaluation réalisées par rapport à la cible<br>4.4. Nombre (#) et types d'activités conjointes MSPP-CSC réalisées, réparties par type.	4. a. Capacité améliorée à suivre et à rapporter les activités d'engagement communautaire<br>4.b. Capacité améliorée de monitorer et de superviser les activités des CSC et d'engagement communautaire<br>4.c. Preuve de CSC autonomes<br>4. d. Amélioration des connaissances, des attitudes et des pratiques liées à la malaria découlant des efforts des CSC et de l'engagement communautaire

CHAPITRE 4 : Boîte à outils de monitoring et d’évaluation des CSC.

Introduction

Le monitoring et l’évaluation constituent une forme de recherche appliquée et il faut obtenir un juste équilibre entre la rigueur et l’utilité pratique en termes de conception et de méthodes. Il est important que les activités de monitoring et d’évaluation soient faites sur mesure pour arriver aux fins désirées, c’est-à-dire qu’il faut collecter la quantité minimum d’informations requises pour produire des connaissances significatives pour les buts et objectifs du programme. Les activités de monitoring et d’évaluation doivent toujours mesurer les résultats non-anticipés (des choses qui arrivent auxquelles on ne s’attendait pas) et tenir compte des facteurs externes. Cependant, si le monitoring et l’évaluation deviennent trop académiques et orientés vers la recherche, ceci peut vite devenir beaucoup trop compliqué, cher et consommer beaucoup de temps. Il est important de se demander : *quelles perspectives vont créer les données du monitoring et de l’évaluation ? Qui peut utiliser ces données ? Comment les données peuvent-elles améliorer le programme ?*

Il est important que le monitoring et l’évaluation incluent la **triangulation** des différentes méthodes de collecte de donnée. En incluant un nombre de méthodes diverses, nous pouvons aborder les problèmes sous des angles différents et générer des connaissances plus utiles et plus précises. Cette diversité de techniques et de méthodes fait du M&E un outil puissant pour les prises de décision du programme et pour apporter le changement. Les méthodes doivent se concentrer sur les informations les plus importants nécessaires à l’évaluation des extrants et des résultats du programme. Elles doivent être réalistes et tenir compte du temps et des ressources financières et humaines.

Le plan de M&E par rapport à l’engagement communautaire inclura une série de méthodes et d’outils de collecte de données pour suivre les indicateurs d’extrants et de résultats dans le cadre du M&E. Les outils de collecte de données sont énumérés ci-après dans la **matrice d’outils M&E**. Le cadre d’échantillonnage et les stratégies d’échantillonnage sont discutés dans chacune des sous-sections ci-après où on trouve des procédures opératoires standard pour chaque outil.

Figure 7 : Matrice des outils de monitoring et d'évaluation

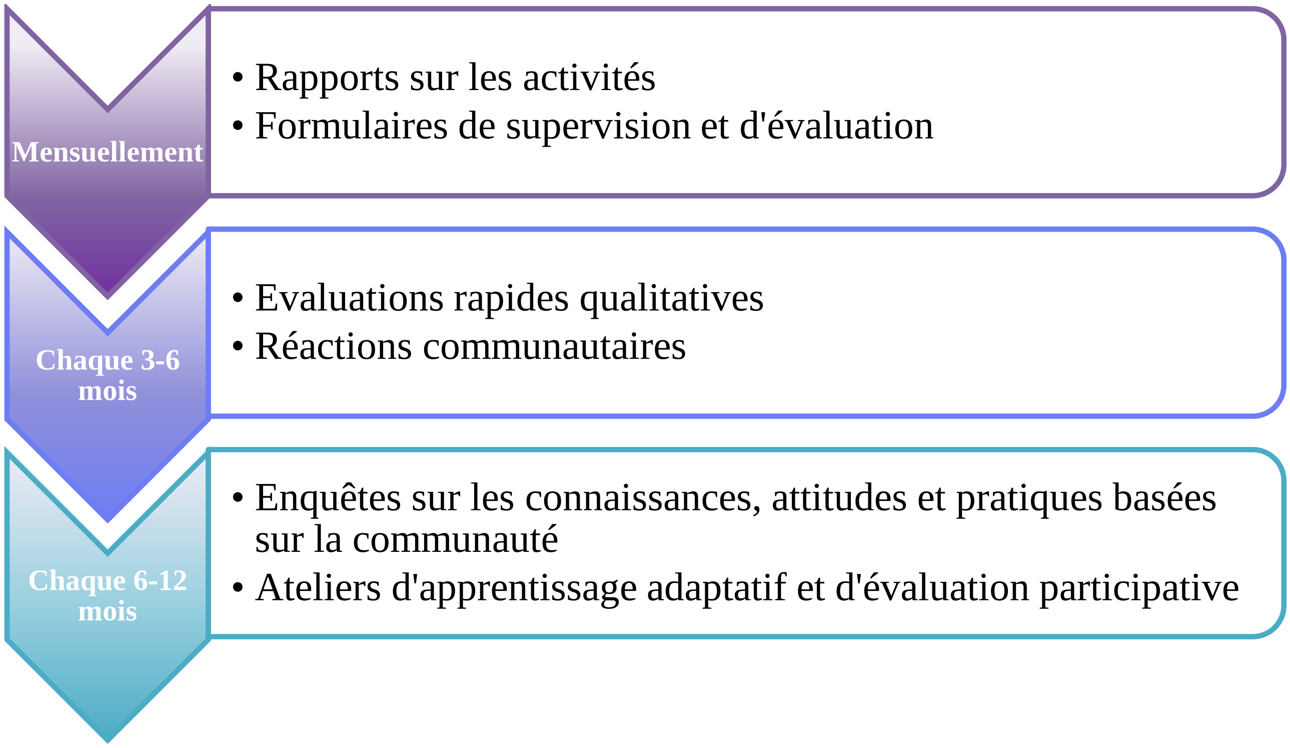

1) Rapports mensuels de monitoring

Les rapports mensuels de monitoring formeront la base des données quantitatives de M&E qui seront collectées et analysées par le programme CSC. Ceci inclura 1) les rapports d'activités des CSC et 2) le rapport sur les activités MSPP/partenaires d'exécution.

L'enregistrement des données doit se faire sur les formulaires de rapport d'activités en annexe et peuvent inclure :

- La récupération des rapports écrits à la main remplis par les CSC ;
- Les appels téléphoniques aux membres du CSC au cours desquels l'équipe de M&E demande à chacun des CSC des informations sur les rapports des activités ;
- La collecte de données électroniques par téléphone intelligent.

Rapports des Activités des CSC

Les secrétaires des CSC soumettront des données sur les réunions des CSC et sur les activités communautaires au personnel de M&E sur une base mensuelle. Ces données seront envoyées par téléphone mobile. Pour la collecte de ces données, il y a deux (2) options :

1) Envoi par téléphone intelligent à une plateforme en ligne (comme Nemo) ;
2) Collecte verbale de données par téléphone mobile où le personnel de M&E contacte chaque CSC par téléphone mobile pour verbalement collecter les informations requises pour les rapports. Il faudra peut-être une combinaison de ces deux (2) options.

Un officier de M&E sera responsable du collationnement et de la révision des données afin de fournir un résumé mensuel au MSPP et aux managers du programme CSC.

Lors des interventions de grand impact (ex : distribution massive de médicaments, distribution de moustiquaires et pulvérisations intra-domiciliaires), l'équipe de M&E doit faire des activités de M&E rapides et communiquer les conclusions sur une base quotidienne et hebdomadaire aux équipes d'exécution du MSPP. Ceci doit se faire par téléphone, par groupe WhatsApp et courriels, et inclure un bref résumé des conclusions et des recommandations.

Les rapports mensuels de monitoring des CSC doivent inclure les formulaires fournis à l'**Annexe 1**.

**Le rapport des activités du CSC doit collecter des données sur les indicateurs de résultats suivants :**<br><br>1.1. Nombre (#) de CSC établis par rapport à la cible<br>1.2. Nombre (#) de membres du CSC qui participant aux activités d'engagement communautaire par rapport à la cible<br>1.3. Nombre (#) de volontaires communautaires affiliés aux CSC<br><br>3.3. Nombre(#) de réunions de CSC tenues annuellement et pourcentage (%) des membres présents<br>3.4. Nombre(#) et types d'activités anti-malaria réalisées par rapport à la cible

3.5. Nombre (#) et types de ressources communautaires mobilisées pour les activités anti-malaria
3.6. Nombre (#) de matériels éducatifs sur la santé distribués aux membres de la communauté par rapport à la cible
3.7. Nombre (#) de membres de la communauté touchés à travers les activités anti-malaria réparties par types d'activité

4.1. Nombre (#) et types d'activités de monitoring et d'évaluation des CSC

Rapport des activités du MSPP/des partenaires d'exécution

Le personnel du MSPP et des partenaires d'exécution impliqués dans le programme CSC sur le terrain soumettront un rapport mensuel des activités par courriel au MSPP et aux managers du programme CSC.

Ceci enregistrera le nombre et les types d'activités réalisés, les défis rencontrés et les recommandations pour les activités futures. Le rapport mensuel des activités du MSPP/des partenaires d'exécution inclura les formulaires fournis à l'Annexe 2.

Dans ce rapport d'activités mensuel le programme CSC collationnera aussi les données spécifiques à la malaria du MSPP, dont le nombre de cas de malaria et de traitement par sous-commune et le nombre de tests de diagnostic rapides de la malaria (TDR) et des tests de microscopie effectués, divisés par +/-. Ces données sont déjà collectées de manière routinière dans la collecte de données du MSPP. Ces données permettront au personnel du programme de planifier pour les augmentations de cas de malaria et, si besoin est, d'activer les Plans de Réponse Rapide du CSC.

Le rapport des activités du MSPP/des partenaires d'exécution collectera des données sur les indicateurs de résultats suivants :

2.1. Nombre (#) et types de membres et volontaires des CSC formés par rapport à la cible
2.2. Nombre (#) et types de formation en CSC réalisée par rapport à la cible
2.3. Matériels de formation élaborés
2.4. Tests de connaissances avant/après et évaluations des formations

3.1. Nombre (#) et types de matériels, de fournitures et d'équipements distribués aux CSC par rapport à la cible

4.2. Nombre (#) et types d'orientation d'outils et de ressources de monitoring et d'évaluation de M&E
4.3. Nombre (#) et types de formations, d'activités d'assistance technique en monitoring et évaluation réalisées par rapport à la cible
4.4. Nombre (#) et types d'activités conjointes MSPP-CSC réalisées, réparties par type.

2) Formulaire de supervision et d'évaluation

Un outil destiné à aider le personnel de soutien du MSPP dans leur tâche de monitoring et de soutien aux CSC, deux (2) formulaires différents de supervision et d'évaluation doivent faire partie des activités routinières de M&E. Ces formulaires fournis à **l'Annexe 3** doivent être utilisés pour le monitoring du programme par le personnel du MSPP. La collecte de données doit être un travail de routine et la fréquence et le nombre de CSC inclus dans le cadre du monitoring seront déterminés par le budget disponible pour les activités de monitoring routinières. Les conclusions-clé du premier formulaire doivent être résumées dans le rapport mensuel des activités du MSPP/du partenaire d'exécution.

Le deuxième formulaire qui inclut des notes pour chaque CSC doit être utilisé à des intervalles fixes « par exemple, tous les trois (3) à six (6) mois » et utilisé comme base pour l'évaluation des prix à décerner aux CSC. Ces données doivent être saisies sur un tableur Excel pour analyse.

3) Evaluations qualitatives rapides

Les évaluations qualitatives rapides constituent une partie importante du système de monitoring et d'évaluation et seront collectées et analysées par le programme CSC. Ceci inclura des entrevues qualitatives semi-structurées, des discussions des groupes focus et des observations des participants réalisées par le personnel du MSPP et du partenaire d'exécution. Il est important que le personnel qui réalise les évaluations qualitatives rapides soit bien formé, compétent et aie une expérience préalable en entrevue qualitative, en collecte et analyse de données.

L'évaluation qualitative rapide doit se faire tous les six (6) mois pour permettre de comprendre :

- La gouvernance et la gestion des CSC ;
- Les forces et faiblesses des activités anti-malaria sur le terrain ;
- Les réactions et réponses de la communauté aux activités du CSC ;
- La motivation et l'incitation des CSC ;
- L'insuffisance des capacités et des besoins en formation ;
- Les forces et défis dans la collaboration continue entre les CSC et le MSPP.

L'évaluation qualitative rapide doit collecter des données sur tous les indicateurs de résultats :

1.a. Présence de CSC fonctionnels à travers la zone géographique durant la période de rapportage du programme avec un réseau de volontaires actifs.

2.a. Membres des CSC qui ont démontré des connaissances améliorées après les activités de formation.

3.a. Plus grande sensibilisation sur l'importance du contrôle et de la prévention de la malaria
3.b. Plus grande sensibilisation sur les signes et symptômes de la malaria et mesures à prendre en cas de symptômes
3.c. Opinions et expériences positives des CSC
3.d. Changements de comportement liés aux risques, à la prévention et au traitement de la malaria
3.e. Histoires de changements des CSC

4.a. Capacité améliorée de suivre et de rapporter les activités d'engagement communautaire
4. b. Capacité améliorée de monitorer et de superviser les activités des CSC et de l'engagement communautaire
4.c. Preuve de CSC autonomes
4. d. Amélioration des connaissances, des attitudes et des pratiques liées à la malaria découlant des efforts des CSC et de l'engagement communautaire

Idéalement, ceci doit se faire longitudinalement au fur et à mesure afin d'évaluer les changements dans le temps avec les mêmes CSC.

Une liste de questions est prévue à **l'Annexe 4.**

Pour créer un **cadre d'échantillonnage**, l'équipe de M&E doit :

1) Etablir une liste de tous les CSC ;

2) Décider du nombre total de CSC à choisir selon la faisabilité, la capacité du personnel et les considérations budgétaires ;
3) Créer une liste des organisations 1) socio-culturelles ; 2) économiques ; 3) politiques importantes et 4) des variations géographiques communes au nombre total de CSC dans le département. Cette liste doit être aussi complète que possible. ;
4) Faire une liste des **indicateurs de résultats importants** tirés des rapports mensuels des activités des CSC ;
5) Une fois la liste finalisée, décider des variables les plus importantes à considérer pour le cadre d'échantillonnage. Ceci doit tenir compte des variables les plus aptes à influencer les indicateurs des extrants et des résultats ;
6) Regrouper tous les CSC selon la liste des variables. Ce regroupement doit produire un consensus les différents « types » de CSC qui ont des variables en commun ;
7) Choisir un certain nombre de CSC à suivre longitudinalement dans le temps sur la base de ce cadre d'échantillonnage de « type commun » ;
8) De ce nombre de CSC choisis, il faut choisir un nombre moindre (trois (3) à cinq (5) CSC par département) pour surveillance sentinelle méticuleuse. Dans ces sites il faut placer plus d'emphase sur la collecte des données d'évaluation rapide des membres de la communauté en comparaison aux autres sites de CSC.

De ce fait, l'évaluation qualitative rapide inclura deux (2) cohortes d'échantillonnage différentes : un plus grand nombre de CSC et un plus petit nombre de CSC devant faire fonction de « site de surveillance sentinelle méticuleuse ». Par exemple, il faudra peut-être inclure dix (10) CSC du département de la Grand'Anse dans le cadre de l'échantillonnage et quatre (4) de ces CSC dans la surveillance sentinelle méticuleuse. La seule différence majeure entre les deux (2) échantillons est que les sites de surveillance sentinelle doivent inclure une recherche qualitative avec une variété de membres de la communauté et doivent être réalisés au niveau communautaire. Durant chaque évaluation qualitative rapide, un cadre d'échantillonnage pour les entrevues semi-structurées, les groupes focus et les observations des participants doit aussi être élaboré selon la disponibilité du personnel et les considérations budgétaires et logistiques.

Les méthodes et les populations-échantillons de ces deux (2) cadres d'échantillonnage seront consistés comme suit :

Cadre d’échantillonnage des CSC	Site sentinelle
Différents membres du CSC afin de trianguler les informations ;<br><br>Personnel médical ayant collaboré avec le CSC incluant le personnel de soutien du MSPP, les agents de santé communautaires et les infirmiers et infirmières locaux ;	Différents membres du CSC afin de trianguler les informations ;<br><br>Personnel médical ayant collaboré avec le CSC incluant le personnel de soutien du MSPP, les agents de santé communautaires et les infirmiers et infirmières locaux ;<br><br>Une large variété de différents types de membre de la communauté incluant les volontaires qui travaillent avec le CSC, les leaders communautaires et les organisations communautaires.

Le nombre exact d’entrevues et de groupes focus dépendra de la quantité de ressources, de temps et de la capacité du personnel disponible pour l’évaluation.

Pour **l’échantillonnage complet des CSC**, idéalement, l’évaluation doit inclure une visite de terrain de la sous-commune de chaque CSC choisi. Pour faciliter d’accès, dans certaines circonstances il serait peut-être nécessaire de convoquer des membres importants du CSC et du personnel du MSPP à des endroits spécifiques pour réaliser les entrevues et les groupes focus. Dans ce cas, le personnel doit considérer un petit remboursement pour les frais de déplacement, les repas et boissons. En outre, il serait raisonnable de réaliser certaines de ces entrevues par téléphone mais pas toutes.

Les activités pour les sites de **surveillance sentinelle méticuleuse** doivent inclure des visites aux localités où les activités des CSC ont été ciblées afin de parler directement aux membres de la communauté. Selon la faisabilité il est conseillé de choisir au moins trois (3) localités différentes à visiter par site de surveillance sentinelle. Le choix doit tenir compte des variations socio-culturelles et géographiques communes à la zone de captage du CSC. Certaines visites de terrain doivent être planifiées pour coïncider avec les réunions de CSC et les activités de sensibilisation. Ceci aidera le personnel de M&E de réaliser des entrevues, des groupes focus et des observations avec les volontaires communautaires, les membres du CSC, les leaders communautaires et le personnel de soutien du MSPP. Cependant, ceci peut augmenter la possibilité de *désirabilité*

sociale (lorsque les gens vous disent ce qu'ils pensent que vous voulez entendre, ou mettent l'accent sur les choses positives au lieu des critiques, des problèmes ou des perceptions et expériences négatives). Il est important que le personnel de M&E soit conscient du potentiel de désirabilité sociale et trouve des moyens de la mitiger en :

- Interviewant séparément les participants et pas seulement en présence des membres du CSC ;
- Expliquant clairement l'objet de l'entrevue ;
- Assurant l'anonymat ;
- Mettant les participants à l'aise en établissant un rapport avec eux avant de poser des questions difficiles ;
- Cherchant des clarifications et posant des questions exploratoires ;

Toutes les entrevues et groupes focus doivent être audio-enregistrées en utilisant un téléphone intelligent et aussi être réalisées par deux (2) membres du personnel de M&E ; un (1) des membres du personnel doit être responsable de mener la discussion/l'entrevue alors que le deuxième prend des notes.

Voir **l'Annexe 5** pour des directives sur les compétences nécessaires aux entrevues qualitatives et **l'Annexe 6** pour des directives sur l'enregistrement des données.

4) Réactions de la communauté

Si le budget et la capacité en personnel est disponible, il est conseillé d'établir un système de feedback communautaire pour compléter l'évaluation qualitative rapide. De plus, ce système de feedback communautaire serait de faciliter l'apprentissage adaptatif sur le terrain en faisant le suivi des questions et des problèmes spécifiques identifiés dans l'évaluation qualitative rapide. Ceci doit inclure le personnel de M&E et du programme du MSPP et des partenaires d'exécution qui visitent le CSC à des intervalles réguliers, tous les trois (3) mois, ou au fur et à mesure que les problèmes sont identifiés ou qu'une assistance supplémentaire est requise. Lors de ces visites, l'équipe de feedback communautaire doit visiter les membres du CSC et se promener autour de la zone de captage du CSC avec quelques représentants du CSC. Durant cette « promenade d'étude », le

personnel de M&E devrait prendre des photos, noter des observations et des réflexions et converser avec les volontaires du CSC, les groupes communautaires et les ménages individuels. Le focus de ces conversations doit être dirigé aux questions et défis particuliers que confronte le CSC comme identifié dans l'évaluation qualitative rapide. Après la promenade d'étude, le personnel du programme CSC doit convoquer une brève réunion avec autant de membres du CSC que possible. Durant cette réunion, le personnel du programme doit :

1) Organiser une matrice de solution de problème à l'aide de feuilles volantes dans les catégories suivantes :

**Problèmes identifiés**		**But**	**Stratégie**	**Tâches**	**Rôles et responsabilités**
1)					
2)					
3)					
4)					

2) La liste des problèmes majeurs doit être basée sur les résultats de l'évaluation qualitative rapide et/ou les rapports d'activités mensuelles, ceux-ci doivent être convenus avant la réunion.
3) Discuter des observations et des conclusions du feedback de la communauté obtenu lors de la promenade d'étude. S'il le faut, ajouter n'importe quel problème supplémentaire ou plus de détails si nécessaire.
4) Prendre des photos sur le terrain et collecter n'importe quelle photo prise par les membres du CSC des activités de routine anti-malaria.

5) Pour chaque problème, le personnel du programme CSC facilitera une discussion de groupe afin de :

   - Identifier le but ;
   - Convenir de la stratégie à suivre pour passer du problème au but ;
   - Convenir des tâches requises pour réaliser la stratégie ;
   - Convenir des rôles et responsabilités de différentes personnes pour chaque tâche ;

6) A la fin de la réunion, le CSC doit garder le tableau-papier pour aider à la mise en œuvre des activités convenues. L'officier de M&E doit prendre une photo du tableau-papier et remplir le Formulaire de Feedback de la Communauté fourni à **l'Annexe 7**.
7) Un tableur Excel doit alors être créé pour saisir les données et faciliter leur analyse.

5) Enquêtes basées dans la communauté sur les connaissances, attitudes et pratiques

Une autre méthode importante à utiliser dans le cadre de la collecte des données quantitatives de M&E sont les enquêtes basées dans la communauté sur les connaissances, les attitudes et les pratiques. Celles-ci doivent être organisées tous les six (6) à douze (12) mois ou initialement et à la fin du projet. Ces enquêtes doivent être relativement brèves et collectées seulement les données les plus essentielles au niveau des ménages sur les indicateurs de résultats énumérés ci-après. Les enquêtes constituent un outil important si elles sont utilisées comme il faut en vue d'explorer les changements dans le temps au niveau de ces indicateurs-clé.

Une liste de questions est fournie à **l'Annexe 8.**

**L'enquête basée dans la communauté sur les connaissances, les attitudes et les pratiques collectera des données sur tous les indicateurs de résultats :**<br><br>3.a. Plus grande sensibilisation sur l'importance du contrôle et de la prévention de la malaria<br>3.b. Plus grande sensibilisation sur les signes et symptômes de la malaria et mesures à prendre en cas de symptômes<br>3.c. Opinions et expériences positives des CSC<br>3.d. Changements de comportement liés aux risques, à la prévention et au traitement de la malaria<br>4. d. Amélioration des connaissances, des attitudes et des pratiques (CAP) liées à la malaria découlant des efforts des CSC et de l'engagement communautaire

Si le budget et le personnel sont disponibles pour mener les enquêtes CAP basées dans la communauté, deux (2) options sont ouvertes à l'équipe de M&E :

1) *Impliquer directement les membres des CSC dans la collecte de données de l'enquête.* Dans cette approche, le personnel du MSPP et du partenaire d'exécution doit former les membres du CSC et les volontaires communautaires dans la conception et l'utilisation d'un instrument d'enquête standardisé. Ceci permettra au CSC d'avoir un sens d'appropriation

par rapport aux résultats, bien qu'une sorte de rémunération et/ou des frais de déplacement doivent être considérés et l'utilisation des membres du CSC pourrait créer la désirabilité sociale dans les résultats.

2) *Déployer une équipe de collecteurs de données au niveau central en-dehors de la zone de captage du CSC.* Dans cette approche, on fait venir une équipe de collecteurs de données formés en-dehors de la zone de captage, ce qui peut réduire la possibilité de désirabilité sociale mais augmenter le coût total de l'enquête.

Quelle que soit l'approche utilisée, l'équipe de terrain devra être formée sur la façon de mener l'enquête et le questionnaire devra être pré-testé. Ce qui veut dire que le questionnaire sera testé dans la communauté avec cinq (5) à dix (10) participants et les questions adaptées si certaines ne sont pas claires ou s'il faut en ajouter d'autres.

Il est conseillé de choisir le même CSC pour l'enquête CAP que celui qui a été choisi pour l'évaluation qualitative rapide, la stratégie d'échantillonnage pourrait inclure tous les CSC choisis ou seulement les sites de surveillance sentinelle. Une fois qu'un nombre approprié de CSC aura été choisi, la stratégie d'échantillonnage doit aussi considérer le nombre d'endroits à échantillonner à l'intérieur de la zone de captage de chaque CSC. Au niveau local, une considération d'échantillonnage final impliquera le choix des participants. Ceci peut inclure :

- Echantillonnage aléatoire : à commencer par le milieu ou la périphérie d'un village et marcher en ligne droite en choisissant chaque 5ème ménage ;
- Si l'enquête doit être répétée, il est conseillé de revisiter les mêmes participants à l'avenir et refaire l'enquête en longitude. Dans ce cas, il faut maintenir un registre des participants à l'enquête ; par exemple, en marquant la localisation de leur maison sur une carte à l'aide de coordonnées GPS, et/ou en relevant leurs numéros de téléphone ;
- Une dernière option est de faire les enquêtes de manière opportuniste aux points centraux comme les marchés, les écoles et les centres de santé. Si ceci est fait, il est important d'assurer qu'un échantillon représentatif de la population a été choisi et que l'échantillon n'a été faussé pour surreprésenter un groupe social spécifique.

Si possible, le personnel de M&E devrait consulter un statisticien qui peut les conseiller sur les méthodes d'échantillonnage statistique appropriées nécessaires à générer des analyses statistiques robustes sur les changements dans le temps afin de faire des représentations causatives. Un tableur Excel doit être créé pour saisir les données et faciliter leur analyse.

6) Ateliers d'apprentissage adaptatif et d'évaluation participative

Comme largement discuté dans le Manuel d'Exécution des CSC, le programme doit inclure de forts mécanismes de feedback quant à la participation afin d'assurer l'apprentissage adaptatif avec les membres du CSC et le personnel du MSPP. Pour y arriver, le personnel du programme CSC doit donc organiser des ateliers formels tous les six (6) à douze (12) mois. Ces ateliers accroîtront la pédagogie utilisée dans le feedback communautaire régulier qui, à travers l'approche soulignée ci-dessus pour la facilitation des discussions de groupe vise à résoudre les problèmes programmatiques par le *brain-storming* et la résolution de problème en groupe.

Le nombre de CSC, les membres des CSC et la fréquence de ces ateliers dépendront du budget disponible et de la capacité du personnel. Idéalement, ils doivent être organisés pour coïncider avec la distribution des prix, ce qui est décrit dans le Manuel d'Exécution et donne une opportunité pour évaluer et adapter le Plan d'Action Communautaire créé par chaque CSC. Donc, les objectifs de ces ateliers peuvent inclure :

- La promotion de l'apprentissage adaptatif ;
- La détermination d'un site pour la distribution des prix CSC ;
- L'évaluation et l'adaptation des Plans d'Action Communautaire ;
- La génération de données pour le monitoring et l'évaluation formels du programme CSC.

Les ateliers peuvent être organisés avec tous les CSC et leurs membres ou avec une sous-série de CSC (par exemple : ceux qui sont impliqués dans la surveillance sentinelle ou les enquêtes basées dans la communauté). Les ateliers peuvent aussi être organisés par département ou en regroupant plusieurs communes. Les frais de déplacement et de repas doivent être considérés pour chaque délégué.

Il faut la présence d'au moins un (1) membre du personnel de soutien du MSPP pour chaque CSC à l'atelier. Ce personnel de soutien aidera à faciliter les exercices de groupe et faire leurs rapports à l'officier de M&E du CSC avec les notes et les données appropriées.

Pour atteindre ces objectifs, les ateliers dureront toute une (1) journée et seront répartis en quatre (4) activités séparées :

1) Feedback du programme aux CSC ;
2) Score de l'évaluation participative ;
3) Produire des histoires de changement ;
4) Réviser le Plan d'Action Communautaire.

L'atelier produira des données sur tous les indicateurs de résultats énumérés ci-dessus pour l'évaluation qualitative rapide. En outre, ce seront les seules techniques de M&E qui fourniront des données sur les indicateurs de résultats *3.2 : nombre (#) de CSC qui ont des plans d'action.*

Des instructions détaillées sur la façon de faciliter l'atelier d'apprentissage adaptatif et d'évaluation participative sont fournies à **l'Annexe 9**.

CHAPITRE 5 : Le plan de travail de M&E des CSC

Introduction

La mise en œuvre d'un système de M&E efficace demande le développement et le suivi d'un plan de travail de M&E. Ce plan de travail doit inclure un nombre de composantes majeures, comme reflété à la **Figure 7** ci-après : 1) pourvoi en personnel ; 2) calendriers ; 3) formation ; et 4) collecte de données. Dans ce chapitre, nous discutons des problèmes importants de chacune de ces composantes et leurs rôles dans les activités de M&E des CSC. Des détails concernant l'analyse des données et le rapportage sont discutés dans le prochain chapitre.

Figure 7 : les quatre (4) composantes du plan de travail M&E des CSC

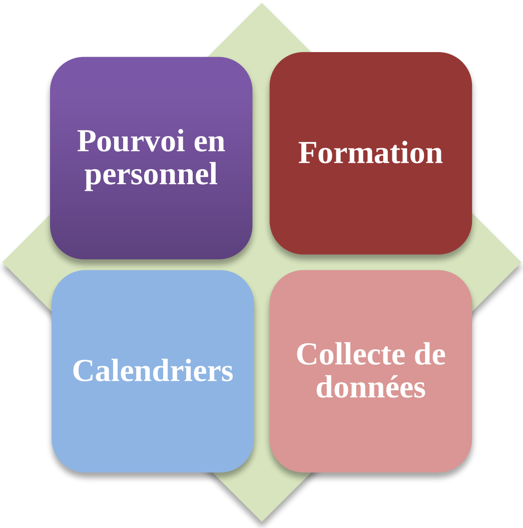

<u>Composante 1 : pourvoi en personnel</u>

Il est important d'assurer qu'un personnel adéquat soit prévu pour les activités de M&E. La composition exacte de l'équipe de M&E dépendra du nombre et de la localisation des CSC à tout moment. En général, la hiérarchie du personnel doit inclure : 1) un officier de M&E avec des antécédents en méthodes de recherche qualitative et quantitative ; 2) un coordonnateur de M&E basé dans chaque département ; et 3) des assistants de M&E et des collecteurs de données sur le terrain. Ce personnel travaillera avec l'équipe de soutien du MSPP dans chaque commune et avec chaque CSC comme décrit dans le Manuel d'Exécution.

Composante 2 : formation

Dans le plan de M&E des CSC, la formation doit se faire à plusieurs niveaux et doit se refaire, au besoin, sur une base annuelle.

D'abord, une orientation approfondie doit être donnée à l'officier de M&E et au coordonnateur de M&E avec tous les détails du Manuel de M&E de même qu'une formation plus poussée en méthodes de recherche, d'analyse et de rapportage. Il faut surtout mettre l'accent sur la formation en analyse de données qualitatives.

En deuxième lieu, un atelier de formation détaillé doit être offert au personnel du MSPP au niveau des départements et des communes. Cet atelier doit être animé par l'officier de M&E et inclure des instructions détaillées sur la façon dont le personnel de soutien du MSPP doit former les CSC sur les rapports d'activités mensuelles.

En troisième lieu, un atelier d'orientation doit être organisé pour les CSC et le personnel de soutien du MSPP. Deux (2) membres de chacun des CSC doivent être invités. Avant cette formation, l'officier de M&E doit visiter chaque CSC et discuter du plan de M&E.

Quatrièmement, si le programme de CSC a des ressources suffisantes, l'officier de M&E doit élaborer un plan de formation à savoir quand et où former les collecteurs de données sur le terrain pour l'enquête CAP basée dans la communauté.

Pour toutes les formations, le transport et les *per diem* doivent être inclus dans le budget.

Composante 3 : calendriers

Les activités de l'équipe de M&E des CSC doivent être soigneusement planifiées et ce processus dépendra du nombre et de la fréquence des outils de collecte de données de M&E et du nombre de CSC. Le calendrier de terrain doit être élaboré sur une base trimestrielle et annuelle et inclure des horaires mensuels et hebdomadaires.

Le calendrier de terrain doit inclure **quatre (4) parties** avec un fort accent sur la supervision et la répartition claire des travaux. La première partie doit inclure les besoins et les projets de formation ; la deuxième partie doit inclure une échéance pour toutes les activités de collecte de données sur le terrain ; la troisième partie doit inclure une échéance pour l'analyse des données ; et la quatrième partie doit inclure un horaire de rapportage. Le plan directeur du calendrier de terrain doit être partagé avec les partenaires et le personnel appropriés.

Figure 8 : quatre (4) calendriers différents

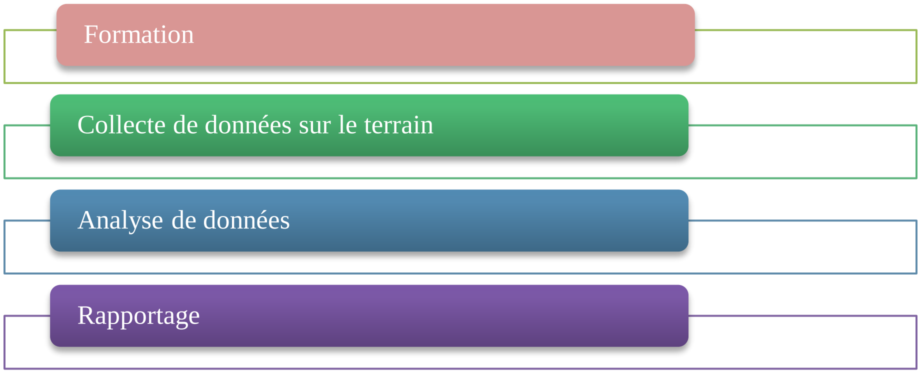

Le plan de travail trimestriel et annuel de M&E doit décrire les activités prioritaires de M&E et les rôles et responsabilités de personnes et de groupes divers. Il doit inclure le coût de chaque activité de même qu'une échéance pour la soumission de tous les résultats. Le plan de travail est utilisé pour coordonner les activités de M&E et évaluer le progrès de la mise en œuvre du M&E tout au long de l'année. Les plans doivent être adaptés selon les réalités du terrain et les besoins du programme d'engagement communautaire.

Le budget doit inclure les éléments suivants :

- Coût des salaires du personnel ;
- Frais de transport pour les activités de terrain ;
- Frais de transport pour les collecteurs de données du MSPP et ceux qui travaillent sur le terrain ;
- Coût des infrastructures (ordinateurs, etc.) ;
- Fournitures et matériels ;
- Sites des réunions, rafraîchissements et *per diem* ;

- Frais de traduction et d'impression ;
- Frais de publication dans les journaux le cas échéant ;

<u>Composante 4 : collecte de données</u>

Tous les outils de collecte de données doivent être **testés sur le terrain**. Une période initiale doit être planifiée pour apporter des modifications aux outils eux-mêmes s'il le faut.

Durant la collecte de données, le personnel de M&E doit suivre des **normes acceptables d'éthique dans la recherche**. Ceci inclut l'obtention de permission et le consentement éclairé. Les normes et stratégies pour l'engagement éthique seront fournies lors des formations. Tous les collecteurs de données devront présenter l'objet de l'exercice de collecte de données à tous les participants et demander leur consentement. Etant donné qu'il ne s'agit pas d'une étude de recherche mais d'un exercice en M&E, l'approbation éthique et le consentement formel peuvent être exigés ou ne pas l'être – ceci doit être confirmé. Toutes les collectes de données doivent se faire avec l'approbation par écrit des managers de programme du MSPP.

Normes d'éthiques de base pour la collecte de données de M&E

Honnêteté : il est important que l'équipe de M&E ne fasse pas usage de mensonges ou de représentations erronées des choses aux membres de la communauté.

Consentement éclairé : il faut à tout moment dire aux gens l'objet de la collecte de données et leur demander de participer. Il faut leur dire qu'ils peuvent refuser et que leur refus n'entraînera aucune conséquence.

Confidentialité : toutes les données collectées des personnes doivent rester confidentielles et ne pas être divulguées publiquement en-dehors de l'équipe de M&E de sorte à identifier une personne en particulier par son nom ou d'une façon qui permet de détecter son identité. Il faudrait utiliser des pseudonymes.

Sensibilité culturelle : Les équipes de collecte de données doivent se conformer à des standards de compétence et de professionnalisme très élevés et déployer tous leurs efforts pour communiquer et se comporter de manière culturellement sensible et compétente.

Réciprocité : les équipes de collecte de données doivent prendre le temps de répondre aux questions des participants sur le terrain et donner un feedback aux membres de la communauté sur les résultats des données de M&E.

En dernier lieu, l’équipe de M&E doit bien réfléchir à l’**enregistrement** durant le processus de collecte de données. L’équipe de M&E peut utiliser des équipements de collecte de données électroniques et des logiciels, des notes manuscrites et/ou des enregistrements audio. Dans chacun des cas, une brève **procédure opératoire standard** doit être élaborée pour la stratégie d’enregistrement de chaque méthodologie.

CHAPITRE 6 : Analyse de données, rapportage et apprentissage du programme

Introduction

Le cycle des données de M&E comporte un nombre d'étapes distinctes dont chacune requiert une attention particulière. Dans la plupart des cas, ces étapes doivent être répétées pour chacune des méthodes de collecte de données. La phase initiale de collecte de données est suivie de quatre (4) autres étapes : **1) saisie et sauvegarde des données ; 2) analyse des données ; 3) rapportage ; et 4) apprentissage du programme.** Suivre l'engagement communautaire par un cadre logique requiert un plan systématique pour la gestion des données, leur analyse et leur utilisation. Etant donné le temps, la main-d'œuvre et les ressources qu'il faut pour collecter ces données, il est impératif qu'elles soient utilisées pour l'apprentissage et la dissémination du programme ; autrement, le système de M&E risque de produire une capacité excédentaire pour la production d'informations sans mécanismes adéquats pour son utilisation pratique.

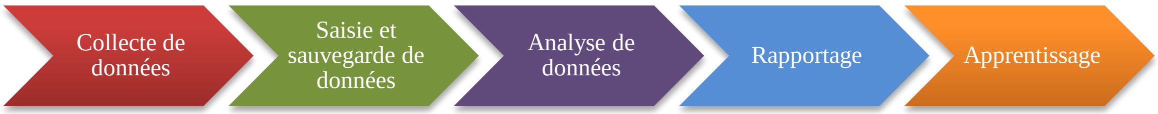

Figure 9 : le processus de collecte de données de M&E

L'élaboration du système de M&E exige des compétences et une formation spéciale pour assurer la rigueur, la reddition de comptes et l'apprentissage. L'analyse des Evaluations Qualitatives Rapides et les ateliers d'apprentissage adaptatif et d'évaluation participative exigeront des compétences en sciences sociales qui ne sont pas facilement disponibles au sein du MSPP. Renforcer la capacité technique pour l'analyse et le rapportage des données constitue une partie importante de la contribution générale des activités de M&E des CSC.

Etape 1 : saisie et sauvegarde des données

Tous les outils de collecte de données exigeront la collecte sur le terrain et la saisie dans une base de données informatisée. La saisie des données est une tâche qui consomme beaucoup de temps et il faut lui dévouer assez de temps et de ressources en personnel. Idéalement, le personnel du MSPP

au niveau départemental peut aider avec la plus grande partie de la saisie des données avec le soutien de l'équipe de M&E.

La saisie des données implique le développement de plusieurs bases de données pour chaque méthodologie de M&E, un système de vérification de la qualité des données doit être élaboré, selon lequel chaque 20ème ligne des données quantitatives sur Microsoft Excel est vérifiée contre le formulaire de collecte de données initial pour précision. Avant l'analyse, il faut nettoyer la base de données. Pour l'analyse des données qualitatives, un logiciel (NVIVO/Atlas) peut être utilisé ou alors les données peuvent être analysées au moyen d'un codage manuel saisi sur Microsoft Word ou Excel.

Les fichiers des données doivent être sauvegardés dans des dossiers bien identifiés avec un code pour permettre la facilité d'accès. Le nom du dossier doit être l'outil de collecte de données (ex : Evaluation Qualitative Rapide) et le nom du fichier doit inclure le lieu et la date.

Toutes les données doivent être téléchargées sur une plateforme Cloud partagée pour faciliter d'accès. L'officier de M&E doit aussi faire une copie de tous les fichiers sur un disque dur externe au quotidien. Chaque semaine, les données du disque dur externe doivent être sauvegardées sur un 2ème disque dur externe gardé sous clé au bureau du MSPP. Personne ne doit avoir accès à ce 2ème disque dur externe sauf l'officier de M&E et l'assistant de M&E.

Quatre (4) critères pour assurer la qualité des données

Précision - les données sont précises si elles mesurent ce qu'elles étaient destinées à mesurer.

Opportunité – les données sont opportunes lorsqu'elles sont actualisées et courantes et lorsque les informations sont disponibles et saisies dans le système à temps.

Complétude – les données sont complètes lorsque les résultats sont complets – chaque outil doit être finalisé dans son intégralité. L'omission des données affaiblit beaucoup les résultats et veut souvent dire que les conclusions ne peuvent pas être tirées.

Intégrité – L'intégrité des données est garantie lorsqu'il y a des procédures ou des protocoles qui ne changent pas selon l'usager ou le moment ou la façon dont les données sont saisies. Ceci permet une collecte constante, le mesurage et le rapportage des données. Il faut être certain que les données saisies sont fiables.

Durant la phase de saisie des données, l'officier de M&E doit réviser toute la série des données par rapport aux critères acceptés pour la qualité des données et refléter les risques possibles de distorsion et de qualité des données qui auraient pu se produire. Quatre (4) critères de qualité des données doivent être pris en compte : précision , opportunité, complétude et intégrité.

Etape 2 : Analyse des données

L'analyse des données implique l'analyse des méthodes individuelles de collecte de données et le collationnement des diverses méthodes par triangulation. Ce Manuel de M&E a été conçu pour assurer que les diverses formes de données soient triangulées. La triangulation est l'utilisation de différentes sources de données pour aborder le même problème pour arriver à une compréhension plus riche, plus approfondie et plus holistique des phénomènes sociaux et des changements du programme. En ce sens, l'officier de M&E aura la tâche de comparer les séries de données pour évaluer les convergences et les divergences.

Le plan d'analyse des données de M&E des CSC inclut l'analyse des données qualitatives, quantitatives et participatives. Le personnel du MSPP au niveau départemental doit exécuter la plus grande partie de la saisie et de l'analyse des données avec l'assistance de l'officier de M&E et d'autres membres du personnel. Il faudra leur donner une formation.

Etape 3 : Rapportage de M&E

En général, il faudra deux (2) types de rapportage pour l'équipe de M&E. Le premier sera de distribuer le **rapport mensuel des activités du MSPP/partenaire d'exécution.** Ceci se fera sur une base mensuelle par courriel de l'officier de M&E au partenaire impliqué et au personnel du MSPP. Il est conseillé d'établir un calendrier électronique pour permettre à l'équipe de réviser les agendas hebdomadaires. Des appels mensuels doivent être organisés par les principaux membres de l'équipe de M&E pour assurer que les activités de M&E sont sur la bonne voie.

Le deuxième type de rapportage sera **les rapports trimestriels de M&E.** Ceux-ci doivent rapporter toutes les activités principales entreprises pendant le trimestre, les résultats de toutes les

collectes de données, les implications de ces résultats pour l'ensemble du plan d'engagement communautaire de même que les activités et le plan de travail pour le prochain trimestre. Voir ci-après une ébauche de la structure.

Ebauche de la structure de rapports trimestriels de M&E

Page de garde

- Résumé narratif des progrès (points forts et points faibles) en comparaison aux rapports trimestriels précédents.
- Décrire n'importe quel ajustement au plan de travail et sa justification.
- **Résultat du M&E**
- Ceci est le corps principal du rapport où il faut présenter une mise à jour des activités réussies et non réussies.
- Présenter les progrès par rapport aux cibles établies au début du trimestre.
- Présenter un résumé des activités de M&E et les résultats principaux choisis.
- Sous chaque graphique, noter votre interprétation pour exécution.
- **Conclusion pour planification**
- Présenter les conclusions tirées qui ont mené à l'atteinte des objectifs.
- Présenter les difficultés et les défis réels, s'il en est, et comment vous les avez surmontés.
- **Prochaines étapes**
- Expliquer comment l'équipe d'engagement communautaire a utilisé les informations apprises pour adapter leur plan de travail pour le prochain trimestre.
- Présenter votre plan de travail pour le prochain trimestre.
- Présenter vos cibles de M&E.
- **Liens**
- Ajouter des liens aux photos.
- Ajouter des liens aux documents.

Etape 4 : Apprentissage du programme

Il ne fait pas de doute que l'apprentissage est la partie la plus importante du processus de M&E et doit se faire à des niveaux multiples dans le système de M&E. Investir du temps et des ressources dans le développement et le maintien des boucles de feedback régulier, entre l'équipe de M&E, le MSPP, les partenaires d'exécution, les CSC, et les membres de la communauté sont des choses essentielles pour la facilitation. Pour que les systèmes de M&E soient un mécanisme effectif pour une meilleure exécution du programme il faudra une communication régulière entre l'équipe de M&E et toutes les autres parties prenantes. Ceci inclura des réunions et des ateliers formels et structurés de même que des conversations informelles et ordinaires.

Le but des données de M&E sera d'initier l'apprentissage du programme à quatre (4) niveaux.

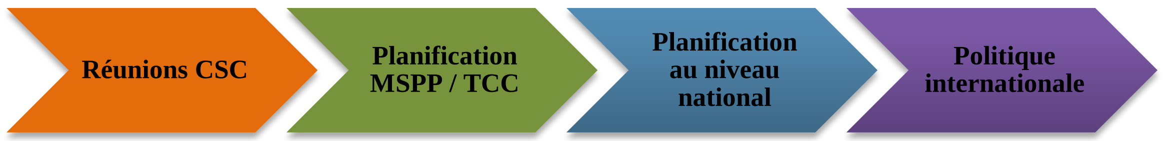

Figure 10 : les quatre (4) niveaux d'apprentissage du programme initié par le Plan de M&E d'engagement communautaire

Niveau d'apprentissage 1 : réunions des CSC et plans d'action

En tant que programme d'engagement communautaire, il est de la plus grande importance que les données de M&E soient présentées et partagées avec les membres des CSC sur une base régulière. Ceci doit se faire aussi souvent que possible. Au sein de chaque CSC, l'officier de M&E du CSC sera responsable de présenter les résultats au CSC et, au besoin, afficher ces données dans un forum public afin qu'elles soient accessibles aux membres de la communauté. Le partage régulier de ces données sur les actuels cas de malaria sera important pour la planification du CSC et aidera aussi à encourager un sens de responsabilité partagée et de collaboration avec le MSPP. Lors des visites de soutien de routine, le personnel du MSPP doit discuter des données précédentes et faire en sorte que tous les points d'action aient été abordés.

Niveau d'apprentissage 2 : Planification au niveau départemental par MSPP/Carter Center

L'officier de M&E communiquera régulièrement avec l'équipe de MSPP/partenaire d'exécution. Ils travailleront en collaboration pour élaborer le plan de travail du CSC et apporteront leur contribution aux stratégies du CSC, leur fréquence, l'élaboration de messages sanitaires, les approches de mobilisation sociale, les intrants en équipements et en besoins de formation. Ils donneront des actualisations régulières sur les résultats et les implications des données de M&E. Ceci doit se faire une (1) fois par mois.

Niveau d'apprentissage 3 : planification du MSPP au niveau national

Il est impératif que les équipes d'exécution de M&E et du CSC soumettent des données au bureau central du MSPP et aux autres partenaires nationaux. Idéalement, il faudrait que le personnel du MSPP du bureau central soit directement impliqué dans la collecte, l'analyse et le rapportage des

données. Les résultats doivent être partagés en envoyant les actualisations par courriel sur une base régulière et aux réunions nationales appropriées, préférablement deux (2) fois par an.

Niveau d’apprentissage 4 : Niveau de politique internationale

Le but de l’élimination mondiale de la malaria continue à susciter beaucoup d’intérêt politique. Le modèle CSC est une approche unique au problème d’engagement communautaire dans l’élimination de la malaria, et il y a peu d’approches d’engagement communautaire rigoureusement évaluées qui soient documentées dans la documentation académique. En suivant la méthodologie décrite dans ce manuel, il devrait y avoir une possibilité de soumettre les résultats à diverses réunions et conférences internationales et au moins dans deux (2) documents de recherche académique. Il est important de ce faire pour renforcer la base de preuves d’engagement communautaire pour l’élimination de la malaria en Haïti et aussi dans d’autres pays. Le personnel doit collationner les données dans des formats appropriés pour présentation et publication aux plus hauts niveaux.

Annexe 1: Rapports des activités du CSC

Suivi de Réunion Bimensuelle

La date de la réunion _________________ (jj/mm/aaaa)

- Département Sanitaire __________________ - Commune _____________________

- Section Communale _____________________ - Localité _______________________

- # de membres du conseil communautaire en total_________________

- # de membres présents à la réunion aujourd'hui ___________________

- Les noms et les rôles communautaires de tous les membres qui sont absents aujourd'hui:

Prénom	NOM	Rôle/Fonction/Titre

Résumez tout ce qui a été discuté à la réunion aujourd'hui:

Est-ce qu'il y a des problèmes ou des quéstions à rapporter?

Décrivez les quéstions / problémes :

Comment ces problèmes / questions ont-ils été résolus ou traités?

Résumez les décisions qui ont été prises à la réunion aujourd'hui:

Notez les points clés de la réunion aujourd'hui

Rapportage d’Activité de Sensibilisation

Date d'activité de sensibilisation _______________ (jj/mm/aaaa)

- Département Sanitaire ____________ - Commune ______________________

- Section Communale ______________ - Localité ________________________

- Lieu d’activité (une église, un marché, une église, une école, etc.) ___________________

- Les coordonnées GPS _______________________________

- Liste de membres du CHC qui ont participé:

Prénom	NOM	Rôle/Fonction/Titre

de personnes qui étaient touchées________________

Choisissez l'activité de sensibilisation de la liste dessous. Cochez autant que nécessaires:

Faire de l'amélioration de l'environnement	
Sensibiliser la communauté sur l'aspect assainissement	
Efforts d'assainissement	
Sensibilisation au paludisme: réunions communautaires dans des installations publiques (école, église, etc.)	
Sensibilisation au paludisme: éduquer le public sur la transmission du paludisme	
Sensibilisation au paludisme: informer le public sur les mesures à prendre en cas de fièvre chez des parents ou des voisins	
Sensibilisation au paludisme: promouvoir le Test et le Traitement	

Sensibilisation au paludisme: informer le public sur le dépistage et le traitement gratuits	
Sensibilisation au paludisme: prévention du paludisme	

Détaillez l'activité de sensibilisation (par exemple: enlever l'eau stagnante):

Est-ce qu'il y a des problèmes ou des quéstions à rapporter?

Décrivez les quéstions / problémes.

Comment ces problèmes / questions ont-ils été résolus ou traités?

Annexe 2: Supervisor Forms

FORMULAIRE DE SUPERVISION ET D'EVALUATION COMITE DE SANTE

DATE: …………………………………………………………….

NOM DU COMITE: ……………………………………………………………………………...

TYPE SUPERVISION : RENCONTRE ☐ **ACTIVITE** ☐ **EVALUATION** ☐

SUPERVISEUR NIVEAU : COMMUNAL ☐ **DEPARTEMENTAL** ☐

NOM/PRENOM SUPERVISEUR : 1.- ………………………………………………..

2.- ………………………………………………..

3.- ………………………………………………...

INFORMATIONS GENERALES

INFORMATION GENERALE SUR LE COMITE	
COMMUNE	
SECTION COMMUNAL	
LOCALITE	
LIEU	
POPULATION DE DESSERTE DU COMITE	
NOMBRE DE MEMBRES	

RENCONTRE MENSUELLE	
PRESENCE	# PRESENT ☐ # ABSENT ☐
LISTE MATERIELS DISPONIBLE ET CONNUE PAR TOUS LES MEMBRES	OUI ☐ NON ☐
VERIFICATION ENTREPOSAGE MATERIELS COMITE CHAQUE 3 MOIS	OUI ☐ NON ☐
ESPACE FIXE DE RENCONTRE MENSUELLE DISPONIBLE	OUI ☐ NON ☐<br>SI OUI……………………………………………………………………….
SUJET PRINCIPAL DE LA RENCONTRE	……………………………………………………………………<br>……………………………………………………………………...<br>……………………………………………………………………...
	TRES BIEN ☐ BIEN ☐<br>☐ ☐

CAPACITE DU GROUPE A TRAITER LE SUJET A L'ORDRE DU JOUR	PASSABLE MAL
	RECOMMANDATIONS :<br>1. ……………………………………………………………… ………………………………………………………………<br>2. ……………………………………………………………… ………………………………………………………………<br>3. ……………………………………………………………… ………………………………………………………………
CALENDRIER DISPONIBLE	OUI ☐ NON ☐
PROBLEME DU COMITE	1. ……………………………………………………………… ………………………………………………………………<br>2. ……………………………………………………………… ………………………………………………………………
PROBLEME RENCONTRE PAR SUPERVISEUR	1. ……………………………………………………………… ………………………………………………………………<br>2. ……………………………………………………………… ………………………………………………………………
ACTIONS ENTREPRISES	1. ……………………………………………………………… ………………………………………………………………<br>2. ……………………………………………………………… ………………………………………………………………

**INITIATIVES DES SUPERVISEURS EN SUPPORT AU COMITE DE SANTE (PENDANT OU APRES)**
1.
2.
3.
4.

Signatures :

____

Coordonnateur du comité supervisé

____________________ ____________________ ____________________

Superviseur 1 Superviseur 2 Superviseur 3

FORMULAIRE DE SUPERVISION ET D'EVALUATION COMITE DE SANTE

DATE: ……………………………………………………………….

NOM DU COMITE: ……………………………………………………………………………...

TYPE SUPERVISION : RENCONTRE ☐ **ACTIVITE** ☐ **EVALUATION** ☐

SUPERVISEUR NIVEAU : COMMUNAL ☐ **DEPARTEMENTAL** ☐

NOM/PRENOM SUPERVISEURS : 1.- ………………………………………………..

2.- ………………………………………………..

3.- ………………………………………………...

INFORMATIONS GENERALES

**INFORMATION GENERALE SUR LE COMITE**	
COMMUNE	
SECTION COMMUNAL	
LOCALITE	
LIEU	
POPULATION DE DESSERTE DU COMITE	
NOMBRE DE MEMBRES	

**GESTION ADMINISTRATIVE ET FINANCIERE**					
		**1**	**2**	**3**	**4**
	**Gestion matériels** : Compte rendu sur l'état des matériels chaque 3 mois.				
	**Inventaire du mobilier :** vérifier véracité dernier inventaire.				
	**Tenue de réunions chaque mois**: fréquence, procès-verbaux de réunions, suivi des décisions prises.				
	**Disponibilité des rapports de dépenses** : cahiers à jour, dépenses discutées et approuvées par les membres				
**SITUATION DU SIS**					
		**1**	**2**	**3**	**4**
	**Tenue cahier de rapport :** existence source primaire de données, propre, Cahier propre, cahier ordonné, etc. …				
	**Disponibilité calendrier d'activité :** existence calendrier mensuel, trimestriel, semestriel…				
	**Rapports mensuels :** à jour dans le cahier, vérification dernier rapport envoyé, rapports archivés…				
	**Situation des archives :** activités inscrites dans le calendrier, enregistrement de toutes les activités au niveau d'un cahier				
	**Existence d'un coin de discussion sur le rapport:** procès-verbal des rencontres mensuelles, …				
**GESTION DES RESSOURCES HUMAINES**					
		**1**	**2**	**3**	**4**
	**Administration**: connaissance des postes clés par les membres du comité, existence de moyens efficaces pour contacter tous les membres, liste téléphone contact des membres,				

	**Gestion des présences** : liste des absents disponibles pour toutes les interventions, discussions sur les absences dans au moins un procès-verbal des trois dernier mois (SOS)…				
	**Conditions de travail :** membre motivé à travers les ressources disponibles, équité, enthousiastes, …				
	**Respect mutuel :** Gestion de conflits, règle de fonctionnement du comité partagé				
	**Jour de célébration des résultats :** Présentation des résultats du comité aux leaders, petite séance récréative				
**GESTION DES RENCONTRES MENSUELLES**					
		**1**	**2**	**3**	**4**
	**Tenue rencontre mensuelle :** date fixe de rencontre, respect des dates au cours des 3 derniers mois…				
	**Procès-verbal des rencontres :** rapport de rencontre enregistré				
**GESTION DES ACTIVITES**					
		**1**	**2**	**3**	**4**
	**Evaluation des problèmes:** Liste des problèmes dans les différentes localités, intervention communautaire par ordre de priorité				
	**Efficacité des interventions** : Etablissement liste des causes des problèmes, arbre à problème, …				
	**Calendrier :** Calendrier en fonction des priorités, respect des calendriers (sauf non-respect documenté),				
	**Planification des activités :** Invitation, rencontre des leaders pour support, sensibilisation avant toute intervention d'assainissement				
	**Pérennité des interventions :** identification d'au moins deux actions durables menées par le comité, …				
	**Documentation correcte des interventions :** Description situation avant, description situation après, photos, vidéos (avant, pendant, après)				
	**Harmonisation /intégration :** recherche de partenariat avec d'autres intervenants				
**GESTION INTERRACTION INSTITUTION - COMMUNE -DEPARTEMENT**					
		**1**	**2**	**3**	**4**
	**Communication :** Communication avec les responsables (santé, mairie, …).				
	**Respect**				
**RECONNAISSANCE DU COMITE DANS LA COMMUNAUTE**					
		**1**	**2**	**3**	**4**
	**Comité connue :** connaissance existence du comité dans la zone (Ecole, Eglise, membre de la communauté)				
	**Rapports avec la communauté** : comite de gestion, réunion avec communauté, procès-verbaux des réunions, enregistrement des suggestions de la communauté				
	**Reconnaissance du comité :** Acceptation du comité dans la communauté,				
	**Inexistence de conflits :** neutralité du comité dans les interventions, non en conflit avec certains secteurs ou membres de la communauté				
	**Appréciation :** Communauté apprécie grandement les actions du comité				

	**Implication de la communauté** : Implication d'au moins 10 personnes de la communauté dans toutes les interventions d'assainissements, au moins 1 dans les activités de sensibilisation comme autres intervenants				

Score final : ____________/___________ **Score Moyen / 100 :** _______________

RECAPITULATION DE LA VISITE

Points forts :

1- ____________________
2- ____________________
3- ____________________
4- ____________________

Points faibles :

1- ____________________
2- ____________________
3- ____________________
4- ____________________

Mesures prise sur place par les superviseurs :

1- ____________________
2- ____________________
3- ____________________
4- ____________________

Recommandations :

1- ____________________
2- ____________________
3- ____________________
4- ____________________

Signatures :

____

Responsable du comité supervisé

________________________ ______________________ ____________________________

Superviseur 1 Superviseur 2 Superviseur 3

Annexe 3: Rapport des activités du MSPP et du partenaire d'exécution

Ce formulaire doit être rempli sur une base mensuelle et soumis par le manager du programme du CSC ou son homologue, au personnel approprié du MSPP et du partenaire d'exécution

Rapport des activités du MSPP et du partenaire d'exécution

Mois du rapportage : ______________________________

Date du rapport : _______________________________

Membre du personnel responsable du rapport : ____________________

Décrire les types d'activités exécutés, incluant le nombre de chacune d'entre elles et l'endroit où elles ont été exécutées :

Décrire les défis que vous avez rencontrés et pourquoi vous les avez rencontrés :

Décrire les recommandations sur la façon dont le programme CSC devrait aborder les défis identifiés :

Suggérer des actions spécifiques à suivre, incluant la personne responsable et le délai d'exécution :

Actualisation des données sur la malaria épidémiologique :

Veuillez faire en sorte de communiquer avec le Directeur Départemental de la malaria du MSPP et joindre à ce rapport mensuel une mise à jour épidémiologique qui fournit des données sur les nouveaux cas de malaria (incluant la localisation des cas), les traitements, les tests de diagnostic (par RDT et microscopie) et autres détails, comme l'exécution des interventions par l'équipe du MSPP.

Annexe 4: Banque de Questions pour les Evaluations Qualitatives Rapides

L'Evaluation Qualitative Rapide inclut des entrevues semi-structurées et des discussions en groupe focus avec les membres des CSC, les volontaires des CSC, le MSPP et autre personnel sanitaire, de même qu'une grande gamme de différents membres et groupes communautaires. Veuillez trouver ci-après une banque de questions pour aider le personnel de M&E dans leurs entrevues et leurs discussions en groupe focus ; ceci doit être adapté selon le type de personne interviewée. Le chercheur peut vouloir ajuster l'ordre des questions, enlever ou ajouter des questions, et mettre l'accent sur certains problèmes par rapport à d'autres. Pour chacune des questions, les chercheurs doivent approfondir avec des questions de suivi pour mieux comprendre *pourquoi et comment* le participant pense d'une certaine façon, ceci est très important.

Section 1 : Questions pour les membres et les volontaires des CSC

1. *Quelle a été votre implication (rôles et responsabilités) au sein du CSC ?*
2. *D'après votre expérience, quelles sont certaines choses positives apportées par le CSC ?*
3. *D'après votre expérience, quelles sont certaines des faiblesses des CSC ?*
4. *Quelles sont les forces que vous avez notées dans la gestion et la coordination du CSC ?*
   a. Réunion et coordination du CSC ;
   b. Prise de décision et planification ;
   c. Implication et coordination avec le MSPP ;
   d. Avec le monitoring et l'évaluation.
5. *Quelles sont les faiblesses que vous avez notées au niveau du management et de la coordination du CSC ?*
   a. Réunion et coordination du CSC ;
   b. Prise de décision et planification ;
   c. Implication et coordination avec le MSPP ;
   d. Avec le monitoring et l'évaluation.
6. *Comment pourrait-on améliorer la gestion et la coordination des CSC ?*
7. *Quelles forces avez-vous observé au niveau des activités et des interventions du CSC ?*
   a. Mobilisation sociale ;
   b. Assainissement de l'environnement ;
   c. Engagement avec les groupes communautaires ;
   d. Education.
8. *Quelles sont les faiblesses que vous avez notées au niveau des activités et des interventions du CSC ?*
   a. Mobilisation sociale ;
   b. Assainissement de l'environnement ;
   c. Engagement avec les groupes communautaires ;
   d. Education.
9. *Comment pourrait-on améliorer les activités d'intervention des CSC ?*
10. *Quelles sont les forces et les faiblesses que vous avez notées au niveau de la capacité du CSC ?*
    a. Pensez-vous que les CSC sont suffisamment motivés ?
    b. Pensez-vous que les CSC ont suffisamment de connaissances et de formation ?
    c. Pensez-vous que les CSC ont suffisamment de directives du MSPP ?
11. *Est-ce-que le CSC s'engage suffisamment avec les volontaires et les autres membres de la communauté ?*

12. *Le CSC a-t-il appris des défis rencontrés et s'est adapté en conséquence ?*
13. *Pensez-vous que le CSC a eu un impact majeur sur la prévention et le contrôle de la malaria ?*
    a. Pourquoi ou pourquoi pas ?

Section 2 : Questions pour le personnel du MSPP et les agents de la santé

1. *Comment êtes-vous impliqué dans le traitement et la prévention de la malaria ?*
2. *Comment avez-vous été impliqué (rôles et responsabilités au niveau du CSC) ?*
3. *D'après votre expérience, quelles sont certaines des choses positives du CSC ?*
    a. Au niveau de la gestion et de la coordination ;
    b. Au niveau des activités et des interventions ;
    c. Au niveau de la motivation et de la capacité ;
4. *D'après votre expérience, quelles sont certaines des faiblesses du CSC ?*
    a. Au niveau de la gestion et de la coordination ;
    b. Au niveau des activités et des interventions ;
    c. Au niveau de la motivation et de la capacité ;
5. *Comment les membres de la communauté ont-ils réagi au CSC ?*
    a. Les choses positives ;
    b. Les choses négatives ;
    c. Les domaines d'amélioration.
6. *Pensez-vous que le CSC a eu un impact majeur sur la prévention et le contrôle de la malaria ?*
    a. Pourquoi ou pourquoi pas ?
7. *Comment le MSPP peut-il mieux supporter les CSC ?*

Section 3 : Questions pour les membres de la communauté au sujet du CSC

1. *Avez-vous entendu parler du CSC ?*
2. *Que pensez-vous des activités du CSC ?*
3. *Avez-vous participé aux activités du CSC ?*
    a. Parlez-moi de votre expérience ;
    b. Qu'est-ce-qui était bien ?
    c. Qu'est-ce-qui n'était pas bien ?
4. *Que pensent la plupart des gens du CSC ?*
    a. Que disent-ils de bon ?
    b. Que disent-ils de pas bon ?
5. *Pensez-vous que le CSC a aidé à réduire la malaria »*
    a. Pourquoi ou pourquoi pas ?
6. *Pensez-vous que le CSC a augmenté la connaissance des gens sur la malaria ?Pourquoi ou pourquoi pas ?*
7. *Pensez-vous que le CSC a eu un impact positif sur le diagnostic et le traitement de la malaria dans votre communauté ?*
    a. Pourquoi ou pourquoi pas ?

8. Quels sont les problèmes ou les doléances que vous avez entendus au sujet du CSC ?

9. Comment pensez-vous que le CSC peut être amélioré pour être plus efficace et avoir plus d'impact dans votre communauté ?

10. La malaria doit-elle être plus intégrée à d'autres problèmes de santé ?

a. Quels sont les problèmes de santé prioritaires pour la population de votre communauté ?

b. Comment ces problèmes peuvent-ils être mieux abordés à travers les groupes communautaires et les actions communautaires ?

c. Le CSC peut-il aider ? Ces problèmes doivent-ils être intégrés aux CSC ?

Section 4 : Questions pour les membres de la communauté sur la malaria

1. *Pensez-vous que la malaria est un problème dans votre communauté ?*
2. *Pouvez-vous me dire comment se propage la malaria ?*
3. *Quels sont les facteurs sociaux qui aident à propager la malaria dans votre communauté ?*
4. *Quelle est la dernière fois que vous avez entendu des messages au sujet de la malaria ?*
   a. *Avez-vous vu des matériels d'éducation sur la malaria ou entendu des annonces à la radio au cours des [INSERT] mois ?*
      i. *Qu'avez-vous pensé de ces choses ?*
      ii. *Comment auraient-elles pu être améliorées ?*
   b. *Comment les informations sur la malaria peuvent-elles être mieux distribuées dans votre communauté ?*
5. *Qui consultez-vous si vous soupçonnez que vous avez la malaria ?*
6. *Avez-vous un agent de santé communautaire dans votre quartier ?*
   a. *Pour quoi les consultez-vous ?*
   b. *Que font-ils de bon pour les gens ?*
   c. *Quelles sont les failles qui existent dans la formation ou la capacité des agents de santé communautaires ?*
7. *Au cours des six [6] à douze [12] derniers mois un membre de votre famille a-t-il pensé qu'il pourrait avoir la malaria ?*
   a. *Parlez-moi de votre expérience ?*
   b. *Où ont-ils cherché de l'aide ?*
   c. *Ont-ils été testé pour la malaria ?*
      i. *Si oui, avez-vous payé pour le test de la malaria ?*
      ii. *Le résultat était-il positif ou négatif ?*
   d. *Ont-ils obtenu des médicaments pour la malaria ?*
   e. *Comment pensez-vous que le diagnostic et le traitement de la malaria peuvent être améliorés dans votre quartier ?*
8. *Que faites-vous dans votre ménage pour empêcher que les gens n'attrapent la malaria ?*
   a. *Hier soir avez-vous dormi sous un moustiquaire ?*
      i. *Si non, pourquoi ?*
      ii. *Tout le monde a-t-il dormi sous un moustiquaire ?*
      iii. *Où avez-vous obtenu vos moustiquaires ?*
   b. *Travaillez-vous avec les membres de votre famille ou autres membres de la communauté pour éliminer les sources d'eau stagnante ?*
      i. *Si oui, comment ?*
      ii. *Avec quelle fréquence ?*
9. *Dans votre quartier, est-ce-que les femmes enceintes ou les enfants reçoivent une éducation ou des interventions spéciales sur la prévention de la malaria dans votre quartier ?*
10. *Pensez-vous que les Hougans doivent être plus intégrés dans l'effort de contrôle de la malaria ?*
11. *Pensez-vous qu'il est possible d'éliminer la malaria dans votre département ?*
12. *Quelles recommandations avez-vous pour le MSPP pour améliorer le contrôle de la malaria dans votre communauté ?*

Si le MSPP a exécuté une distribution massive de médicaments ou la pulvérisation intra-domiciliaire dans la zone, des questions supplémentaires sur ces interventions doivent être ajoutées à cette liste.

Annexe 5 : Conseils pour les entrevues qualitatives

Pour faire de bonnes interviews qualitatives, il faut avoir de bonnes compétences sociales dont :

- *Demander le consentement :* expliquer le projet et réaffirmer le consentement informé de la personne ;
- *Garder les questions essentielles simples : Quoi ? Quand ? Qui ? Où ? Pourquoi ? quelle quantité ?*
- *Eviter les questions suggestives ;*
- *Poser de bonnes questions d'« approfondissement » que l'on appelle aussi des questions de suivi ;*
- *Concentrez-vous sur une (1) à trois (3) choses majeures que vous désirez discuter dans n'importe quelle interaction ;*
- *Etablir un « rapport » pour tenir compte de la désirabilité sociale ;*
- *Utiliser la langue et les catégories des personnes locales et éviter n'importe quelle terminologie biomédicale qui n'est pas nécessaire.*

Mener une conversation avec un objectif :

- Soyez flexible/conversationnel;
- Encourager les anecdotes. Eviter trop de généralisations ;
- Ne pas dominer la conversation ;
- Accepter les pauses ;
- Faire l'équilibre entre le formel et l'informel ;
- Quelques minutes de causerie amicale/informelle ici et là pendant toute l'entrevue peuvent vraiment aider ;
- Suivre les indices des participants ;
- Soyez attentif : prêtez attention au langage corporel et ce qui pourrait être « caché » ou qui ne serait pas dévoilé par le participant pendant l'interaction.

Connaissez bien vos questions :

- Trois (3) types de question : question principale, question de suivi et d'approfondissement ;
- Ne regardez pas vos notes pour lire les questions : notez quelques rappels spécifiques dans la marge de votre cahier de notes ;
- Commencez la conversation avec des questions légères ;
- Passez du général/au sociétal/au personnel/à l'individuel ;
- Utilisez la langue et la terminologie locale ;

- Evitez de poser trop de questions ;
- Utilisez plutôt des méthodes indirectes pour obtenir des réponses au niveau de la conversation ;

Apprenez des stratégies et soyez préparé :

- Utilisez des scénarios hypothétiques : « que feriez-vous si… ? » ;
- Demandez des éclaircissements, des exemples et des détails ;
- Contrevérifiez : « j'ai entendu dire… ? » ou « je découvre que les hommes pour la plupart… » ;
- Concentrez-vous sur les histoires personnelles ;
- Stratégie de questionnement : « parlez-moi de __... racontez-moi toute l'histoire. » ;
- Parfois il serait bon de reformuler la même question ;
- Posez des questions bêtes ou naïves ;
- Utilisez des termes de comptage informel comme « très peu » / « certains » / « la plupart des gens » ;
- Faîtes en sorte de différencier entre les perceptions et les pratiques d'une personne par rapport à quelque chose et ce qu'elle pense que d'autres membres de la communauté pensent ou font ;
- Montrez que vous avez des « informations privilégiées » pour suggérer que vous êtes prêts à obtenir des connaissances détaillées sur un sujet ;

Comment dois-je prendre des notes :

- Tenez le livre avec nonchalance ou mettez-le dans votre poche ou dans votre sac. Ne cachez pas vos notes ;
- Sortez votre cahier de notes lorsque la personne dit quelque chose d'intéressant.
    - Exemple : « *ce que vous dîtes est très intéressant et très important. Je ne veux pas oublier certaines parties de ce que vous dîtes. Je veux bien comprendre. Est-ce-que cela vous dérange si je prends quelques notes pendant que vous me parlez de ces choses ? » ;*
- Utilisez vos notes pour clarifier et poser des questions.

Annexe 6 : Enregistrement des données dans les Evaluations Qualitatives Rapides

L'enregistrement doit inclure des notes manuscrites et des enregistrements audio à l'aide de téléphones intelligents. Chaque travailleur de terrain doit avoir deux (2) cahiers de notes pour : 1) des notes rudimentaires, des notes de réflexion, et un résumé des méthodes ; et 2) pour des notes plus longues. *Chaque chercheur de terrain doit passer au moins une (1) heure à une heure et demie chaque jour à finaliser leurs notes de réflexion, leurs notes extensives et le résumé des méthodes pendant qu'il est sur le terrain.*

Notes rudimentaires

- Nous nous fierons principalement aux notes manuscrites « rudimentaires » en utilisant les cahiers de notes ;
- Ecrire autant de mots et de phrases-clés que vous pouvez pendant que la personne parle ;
- Retenez les mots exacts de la personne aussi fidèlement que possible. Ceci est particulièrement important pour les phrases-clés ou les phrases innovatrices que la personne utilise pour faire passer un point important ;
- Pour les faits très importants, vous pouvez dire : « attendez une minute, il est très important que j'écrive ceci exactement comme vous venez de le dire. » Faîtes-le seulement pour les faits très importants ;
- Vous pouvez donner lecture de vos notes à la personne et leur demander si vous avez raté quelque chose ;
- Après chaque discussion ou tout de suite avant qu'elle finisse, enregistrez (ou demandez) le profil général du/des participants : genre, âge approximatif, statut/position sociale ;
- Immédiatement après l'entrevue, repassez sur vos notes et ajoutez « matériels remémorés » que vous auriez pu manquer pendant que la personne parlait (rédigez vos notes de terrain).

Notes de réflexion.

- Ces notes constituent votre journal personnel de vos pensées relatives à votre travail sur le terrain ;
- Vous devez noter des perceptions importantes que vous ressentez sur le sujet de même que des pressentiments, des hypothèses, et des questions de suivi émergentes que vous aimeriez soulever avec l'équipe lors de l'analyse de groupe.

Enregistrements audio

- Les enregistrements audio doivent être utilisés dans les entrevues avec les informateurs-clés et les groupes focus mais seulement après avoir obtenu le consentement de tous les participants. Les enregistrements audio doivent être utilisés pour ajouter des détails supplémentaires aux notes lorsque l'équipe de terrain rédige « les notes extensives », la première méthode d'enregistrement de données doit être les notes manuscrites.

Notes extensives

- *Une fois par jour, vous devez ajouter à vos notes rudimentaires, passer des « notes rudimentaires » à des demi-phrases ou des phrases complètes, des notes que vous avez prises durant l'entrevue avec des détails descriptifs et des observations.*
    - Ne pas noter seulement les points saillants, mais plutôt décrire les choses en profondeur ;
    - Inclure certaines citations mot-à-mot (entre guillemets) avec des termes locaux ;
    - N'oubliez pas les détails pour chaque entrevue : écrire l'âge approximatif et le genre (ainsi que d'autres détails sociodémographiques pertinents, incluant le statut/la position sociale) entre parenthèses : [sexe, âge, autres facteurs sociodémographiques] ;
    - Vous donnerez aussi une note à chaque entrevue au moyen d'une lettre selon le niveau d'engagement démontré par les participants et l'importance des données : très bien (B), assez bien (AB) ou faible (F). Utilisez les lettres B, AB et F à cette fin.
- En faisant ceci, réfléchissez sur la façon dont ces informations nouvelles et intéressantes s'inscrivent dans les autres données que vous collectez et notez n'importe quelles idées et questions de suivi dans votre *cahier de notes de réflexion*.

Consentement informé

- Il est impératif que le personnel de M&E se présente et explique l'objectif des activités de recherche. Il faut chercher à obtenir un consentement verbal ou écrit approprié ;
- Suit un exemple d'une stratégie de présentation simple :
    - *Bonjour. Je suis un [chercheur/agent de santé]. Je travaille avec […] et nous demandons aux gens de la communauté ce qu'ils pensent et leurs expériences par rapport à la malaria et aux activités anti-malaria organisées par le MSPP, incluant les Conseils de Santé Communautaire (CSC). Nous posons plusieurs types de questions différentes aux gens. Le but de notre recherche est de comprendre comment améliorer le programme de la malaria. Est-ce-que ça vous dérange si je vous pose certaines questions de recherche ?*

Journal des activités quotidiennes

- On vous donnera un journal d'activités quotidiennes à remplir chaque jour après votre travail sur le terrain et à remettre à votre superviseur.

**Date :**			
**Lieu de la recherche :**			
**Nom du chercheur :**			
**Méthode**	**Nombre**	**Caractéristiques socio-démographiques de(s) participant(s)[1]**	**Temps total de l'activité (heures par jour)**
Entrevue avec l'informateur-clé :			
Discussion de groupe			
Autre ________________			

[1] Ceci doit être saisi en fonction de l'âge et du sexe approximatifs. Pour la discussion de groupe, une description générale convient; pour exemple, un «groupe de jeunes hommes».

Annexe 7 : Formulaire de feedback communautaire

Nom du CSC visité	
Date	
Personnel MSPP présent	
Personnel du partenaire d'exécution présent	
# de membres du CSC présents	
Conclusions majeures à partir du feedback communautaire	
Problèmes identifiés durant la réunion du CSC	
Stratégies identifiées pour avancer du problème vers le but	
Tâches convenues prévues pour réaliser la stratégie	
Problèmes à suivre	

Annexe 8 : Questions pour les enquêtes sur les connaissances, les attitudes, et les pratiques basées dans la communauté

Il est conseillé que les enquêtes basées dans la communauté sur la connaissance, l'attitude et les pratiques (CAP) consistent en questionnaires relativement brefs axés sur les indicateurs-clés des résultats et à retracer le changement dans le temps. Ci-après, un échantillon des questions que vous pouvez adapter pour votre utilisation. Tout questionnaire devrait être modifié pour s'adapter aux activités du programme.

Section 1 : caractéristiques sociodémographiques

1. Genre
2. Age
3. Niveau d'éducation (défini comme étant la dernière année de scolarité achevée)
4. Etat matrimonial
5. Occupation
6. Possession de téléphone portable
7. Combien de personnes vivent normalement dans votre maison physiquement ?
    - Combien d'adultes [de plus de 18 ans] ?
    - Combien d'enfants [de 6 à 18 ans] ?
    - Combien de bébés [de 5 ans et moins] ?
8. Combien de femmes sont enceintes actuellement dans votre ménage ?

Section 2 : Sensibilisation sur la malaria et le CSC

9. Avez-vous entendu parler de la malaria ?
10. Quelle a été la dernière fois que vous avez entendu des messages sur la malaria ?
11. Où avez-vous entendu des messages sur la malaria pour la dernière fois ?
12. Avez-vous entendu parler d'un groupe communautaire local [nom] focalisé sur le contrôle de la malaria ?
13. Avez-vous participé à des activités organisées par le groupe communautaire local sur la malaria ?
    - Si oui, quand ?

- Si oui, dans quelle activité étiez-vous impliqué ?
- Si oui, avez-vous eu une expérience positive, neutre ou négative en participant à ce groupe ?
  - Expliquez votre réponse : ____

14. Quelles sont les bonnes choses, s'il y en a, que vous avez entendu d'autres gens par rapport au groupe local sur la malaria ?
15. Quelles sont les mauvaises choses, s'il y en a, que vous avez entendu d'autres gens par rapport au groupe local sur la malaria ?

Section 3 : Expériences et traitement de la malaria

16. Avez-vous un membre de la famille qui a eu la malaria lors des [6-12] derniers mois ?
17. Quels sont les signes et symptômes de la malaria ?
    - Fièvre
    - Maux de tête
    - Frissons
    - Nausée/vomissement
    - Courbatures/douleurs aux articulations
    - Perte de connaissance
    - Jaunisse
18. Avez-vous eu de la fièvre lors des trois (3) derniers mois de [mois] à [mois] ?
19. Avez-vous cherché de l'aide pour votre fièvre ?
    - Si oui, où ?
20. Avez-vous consulté un agent de santé communautaire ?
21. Quels médicaments avez-vous reçus ?
22. Avez-vous subi un test de dépistage de la malaria ?
    - Si oui, avez-vous payé pour votre test de dépistage de la malaria ?
23. Votre test était-il positif ?
24. Comment pouvez-vous traiter la malaria ?

Section 4 : Prévention de la malaria

25. Comment se propage la malaria ? (Cochez tout ce qui s'applique)
    - Moustique
    - Incertain
    - Eau sale
    - Autre façon : ________________
26. Comment pouvez-vous prévenir la malaria ?
    - Moustiquaires
    - Dormir sous une moustiquaire
    - Eviter les piqûres de moustiques
    - Couper les herbes autour de la maison
    - Flaques d'eau
    - Brûler des feuilles
    - Médicaments/distribution massive de médicaments
    - Pulvérisation intra-domiciliaire à l'aide de produits chimiques

27. Hier soir, avez-vous dormi sous une moustiquaire ?
    - Si non, pourquoi pas ?
        - D'autres personnes dans le ménage utilisaient la moustiquaire
        - Il fait trop chaud sous la moustiquaire
        - Il n'y avait pas de moustiques
        - La moustiquaire était sale
        - Il n'y avait pas assez de place pour la moustiquaire
28. Possédez-vous une moustiquaire ?
29. Où avez-vous trouvé votre moustiquaire
30. Combien de moustiquaires avez-vous chez vous ?
31. Est-ce-que tout le monde dort sous une moustiquaire ?

Section 5 : Distribution Massive de Médicaments et Pulvérisation Intra-Domiciliaire

Si le MSPP a exécuté une distribution massive de médicaments ou une pulvérisation intra-domiciliaire dans la zone, des questions supplémentaires doivent être ajoutées ici pour ces interventions.

Annexe 9 : Directives pour l'atelier sur l'apprentissage adaptatif et l'évaluation participative

Un atelier d'apprentissage adaptatif et d'évaluation participative doit être organisé tous les six (6) à douze (12) mois. Il y a plusieurs étapes pour l'organisation de ces ateliers :

1) Préparation avant l'atelier ;
2) Facilitation de l'atelier ;
3) Rapportage.

Préparation avant l'atelier

L'organisation des ateliers CSC exige d'abord une décision sur la fréquence à laquelle il faut tenir les ateliers, le nombre total de CSC et le nombre de membres par CSC qu'il faut inviter. Idéalement, les ateliers devraient être organisés tous les six (6) à douze (12) mois pour correspondre avec la distribution des prix comme souligné dans le Manuel d'Exécution. Ceci dépend du budget disponible et de la capacité du personnel. Il y a quelques options sur la façon d'organiser les participants :

- Inviter quelques (2 à 4) membres de chaque CSC à un (1) seul grand atelier ;
- Inviter quelques (2 à 4) membres des CSC seulement des sites sentinelles de monitoring à un (1) seul grand atelier ;
- Organiser quelques ateliers répartis par arrondissement dans chaque département. On peut inviter quelques membres (2 à 4) ou tous les membres des CSC.

Il est important de planifier un budget pour l'atelier qui tient compte :

- Du transport de tous les participants ;
- De la nourriture et boissons ;
- Papiers, plumes et tableaux-papier.

Il est important de coordonner l'atelier avec le MSPP pour permettre au personnel du soutien du MSPP pour que chaque CSC participe à l'atelier. Ce personnel de soutien aidera à faciliter les exercices de groupe et fera son rapport à l'officier CSC de M&E avec des notes convenables et

des données. Il est important de décider d'une procédure opératoire standard claire pour l'enregistrement du score d'évaluation participative et la production d'histoires de changement. Ceci doit inclure les notes, les tableaux-papier et les enregistrements audio (qui peuvent être utilisés pour vérifier l'exactitude des notes et des tableaux-papier). Au moins un (1) membre du personnel de soutien du MSPP doit être présent pour chaque CSC présent. Des invitations formelles doivent être envoyées à chaque CSC et à chaque membre du personnel de soutien du MSPP. Les CSC doivent être encouragés à décider en groupe des personnes qui doivent assister à l'atelier.

Avant l'atelier, il est important que tout le personnel de soutien du MSPP lisent les sections du Manuel d'Exécution qui décrivent la planification des Plans d'Action Communautaire et aussi se familiariser avec le protocole de cet atelier. Les membres des CSC doivent venir à l'atelier munis de leur Plan d'Action Communautaire.

Facilitation de l'atelier

L'atelier doit être une activité d'une (1) journée qui commence à 9 :00 AM et qui prend fin à 7 :00 PM. Un ordre du jour doit être fourni à tous les participants et être réparti en cinq (5) activités comme suit :

Feedback du programme aux CSC	1 heure	Présenter les conclusions des données de M&E et donner une actualisation de l'épidémiologie de la malaria
Scoring de l'évaluation participative	1 heure	Donner des scores à cinq (5) domaines du programme CSC
Production des histoires de changement	1 heure	Produire des histoires de changement
Révision des Plans d'Action Communautaire	3 heures	Actualiser les Plans d'Action Communautaire des CSC

1. *Feedback du programme aux CSC*

L'atelier doit commencer avec de brèves présentations par l'équipe de M&E. Les données disponibles du programme de CSC incluant les tendances chronologiques sur certains des indicateurs d'extrants et de résultats. Tout ceci doit être présenté dans un format simple et

accessible. Ceci doit être suivi par une brève présentation du coordonnateur de la malaria du MSPP qui doit résumer les tendances de la malaria. La présentation de la malaria doit soumettre le nombre de cas et de traitements de la malaria par sous-commune et le nombre de tests de Diagnostic Rapide de la malaria et de test microscopiques effectués, divisés par +/- . Les présentations doivent être suivies par une période question-réponse. Tout l'exercice doit durer une (1) heure au total sans plus.

2. *Scoring de l'évaluation participative*

La deuxième partie de l'atelier doit inclure un exercice de scoring. Si possible, un (1) membre du personnel de soutien du MSPP doit convoquer chaque CSC et ce membre ne doit pas normalement travailler avec ce CSC en particulier. L'exercice devrait durer une (1) heure. Ensuite, le membre du personnel du MSPP doit donner les informations suivantes :

Expliquer que nous allons passer par une série de cinq (5) domaines du programme CSC et leur donner un score. Il n'y a pas de bon ou de mauvais score, il s'agit d'un exercice d'équipe que nous utilisons pour expliquer ce qui marche bien et ce qui a besoin d'amélioration.

Le personnel de soutien du MSPP doit prévoir cinq (5) pages de feuilles volantes sur la table avec les rubriques suivantes :

- Leadership ;
- Planification et gestion ;
- Implication communautaire ;
- Partenariat avec le MSPP ;
- Monitoring, évaluation et apprentissage.

Sur chaque feuille volante, le membre du personnel du MSPP doit tracer un cercle à côté de chaque membre du CSC présent. Ceci sera utilisé par chaque membre individuel du CSC pour noter leur propre score. Dessiner un grand cercle au milieu de la feuille volante où le score convenu par le groupe sera reflété. Ceci doit être fait pour chacune des cinq (5) feuilles volantes. Expliquer les

critères du scoring et les écrire clairement sur le tableau-papier pour permettre à tous les participants du CSC de voir et de s'y référer. Les critères sont les suivantes :

- **Fort (5)** les choses qui ont très bien marché ;
- **Fort mais requiert une certaine amélioration (4)** : les choses qui ont bien marché mais qui méritent des améliorations ;
- **Moyen (3) :** des choses qui sont bonnes mais doivent être améliorées ;
- **Faible (2) :** les choses n'ont pas trop bien marché et méritent beaucoup d'amélioration ;
- **Très mal (1) :** les choses ont très mal marché et où il faut des changements sérieux.

Expliquer comment chacun des cinq (5) domaines doit être évalué. Maintenant nous allons lire la description du domaine d'abord en groupe c'est-à-dire il doit être lu et expliqué par le personnel sénior du programme pour toute la salle. Des questions doivent être prises pour clarifier n'importe quelle confusion spécifique. Des feuilles avec la description des domaines doivent être distribuées à chaque groupe pour référence. Les groupes doivent avoir sept (7) minutes pour donner un score à chaque domaine, à ce moment-là, une cloche ou une alarme doit être sonnée et le personnel sénior lire la description du prochain domaine.

La description de chaque domaine est fournie ci-après au Tableau 1. Il faut expliquer au groupe que le membre du personnel du MSPP sera responsable d'enregistrer les raisons pour lesquelles le groupe a décidé de donner un score à un domaine en particulier.

A la fin de l'exercice, le membre du personnel du MSPP ramassera les scores.

Tableau 1 : description des cinq (5) domaines

Ceci doit être donné à chaque CSC pour référence rapide durant l'exercice.

**Domaine**	**Description**
1. Leadership	Considérer le leadership de votre CSC et la façon dont les différents membres ont interagi les uns avec les autres, résolu des conflits et contribué des idées nouvelles
2. Planification et gestion	Considérer la façon dont votre CSC a pu planifier, adapter et exécuter les activités dans la communauté
3. Implication communautaire	Considérer la façon dont votre CSC a réussi à impliquer les membres de la communauté
4. Partenariat avec le MSPP	Considérer le niveau de partenariat entre votre CSC et le MSPP et le degré de soutien apporté par le MSPP à vos activités de CSC.

5. Monitoring, évaluation et apprentissage	Considérer la façon dont votre CSC a réussi à discuter des défis et élaborer des solutions pour les aborder

3. Production des histoires de changement

Basé sur les discussions de groupe de scoring participatif, les membres des CSC doivent alors passer à une prochaine activité après une pause. L'exercice des histoires de changement consistera à produire et à documenter des histoires de changement positif des activités du CSC dans la communauté et l'apprentissage adaptatif et la gestion des CSC eux-mêmes. Ceci sera constitué par les membres du CSC avec l'aide du personnel de soutien du MSPP qui feront du *brainstorming* et réfléchirons sur deux (2) questions :

1) « Quels sont les changements les plus importants qui ont eu lieu dans la communauté [depuis le dernier atelier d'apprentissage adaptatif] ? »
2) « Pourquoi ces changements sont-ils importants pour l'élimination de la malaria ? »

Le groupe peut décider d'autant de changements importants que possible ; l'idéal est d'avoir deux (2) à cinq (5) histoires de changement. Le personnel de soutien du MSPP sera responsable de documenter les histoires de changement en les écrivant. Les enregistrements audio peuvent aussi être utilisés pour assurer que tous les détails soient enregistrés.

4. Révision des Plans d'Action Communautaire

La révision des Plans d'Action Communautaire doit suivre les histoires de changement. Ceci demande que chaque CSC révise son Plan d'Action Communautaire. La première partie de cet exercice inclura la facilitation d'une discussion par le personnel de soutien du MSPP avec le CSC sur les points de discussion suivants :

1) *Votre CSC a-t-il un Plan d'Action Communautaire complet ?*
   a. *Sinon, qu'est-ce-qui manque et pourquoi ?*
   b. *Si oui, quels sont les défis confrontés par le CSC suite au Plan d'Action Communautaire ?*

Selon la réponse à ces questions, chaque CSC doit être mis en groupe avec d'autres CSC qui sont à une étape similaire d'élaboration de leur Plan d'Action Communautaire. Un (1) membre du personnel de soutien du MSPP doit être affecté à des CSC spécifiques afin de faciliter l'élaboration par les CSC de certaines parties spécifiques de leur Plan d'Action Communautaire qui manquent. Si le MSPP a des plans futurs pour l'exécution de n'importe quelles interventions ciblées (comme la pulvérisation intra-domiciliaire, la distribution massive de médicaments, la distribution de moustiquaires imprégnés) ou s'il y a des changements qui se font au niveau du programme CSC alors, à ce moment-là, ceci doit être discuté dans l'atelier pour permettre l'actualisation convenable des Plans d'Action Communautaire des CSC.

Ce processus d'actualisation des Plans d'Action Communautaire doit se reposer sur la description des CAP dans le Manuel d'Exécution. Il y a une série d'activités décrites dans le Manuel comme noté ci-après :

Tableau 2 : Liste des onze (1) rapports d'activité décrits dans le Manuel d'Exécution

**Activité No.**	**Titre de l'activité**
Activité 1	Elaboration d'une vision pour votre CSC
Activité 2	Cartographie des facteurs de risque de malaria dans votre zone
Activité 3	Elaboration d'une liste de questions en suspens qu'ont les membres du CSC concernant la malaria
Activité 4	Discussion des forces et faiblesses des approches à la malaria
Activité 5	Identification des interventions routinières
Activité 6	Identification des lieux où vous voulez exécuter vos interventions
Activité 7	Identification des personnes à cibler
Activité 8	Identification de la fréquence d'exécution de vos activités
Activité 9	Elaboration d'un calendrier d'activités de six (6) mois
Activité 10	Plan de Réponse Rapide du CSC
Activité 11	Plan des Matériels et des Ressources

Le focus de cet atelier doit être sur l'actualisation des activités 8 à 11 qui doit inclure une concentration sur les groupes CSC de quartier si ceci est pertinent. Avant l'atelier, les membres des CSC et le personnel de soutien du MSPP doivent avoir revu les détails sur la façon d'élaborer les Plans d'Action Communautaire à partir du Manuel d'Exécution. Tous les participants doivent se préparer avant de venir et être munis de copies de leur Plan d'Action Communautaire. Les membres du personnel du MSPP et du partenaire d'exécution peuvent décider d'utiliser cet atelier pour raffiner et actualiser les Plans d'Action Communautaire des CSC. Cette partie de l'atelier doit être répartie en deux (2) sessions d'une heure-et-demi chacune pour un total de trois (3) heures.

5. *Distribution des prix*

Si le budget permet d'avoir une distribution de prix comme décrit dans le Manuel d'Exécution, ceci devra se faire tout à la fin de l'atelier avant le départ des participants.
